# Analysis of network public opinion on COVID-19 epidemic based on the WSR theory

**DOI:** 10.3389/fpubh.2022.1104031

**Published:** 2023-01-13

**Authors:** Kun Yang, Junqi Zhu, Li Yang, Yu Lin, Xin Huang, YunPeng Li

**Affiliations:** School of Economics and Management, Anhui University of Science and Technology, Huainan, Anhui, China

**Keywords:** network public opinion, grounded theory, WSR methodology, COVID-19, qualitative analysis

## Abstract

**Objective:**

To obtain the influencing factors of public opinion reactions and to construct a basic framework of the factors causing the occurrence of online public opinion in the epidemic area.

**Methods:**

The hot news comments on microblogs during the epidemic in Shanghai were collected and analyzed with qualitative analysis, grounded theory, and the “Wuli-Shili-Renli” (WSR) methodology as an auxiliary method.

**Results:**

(1) Three core categories of the Wuli system, the Shili system, and the Renli system, 15 main categories, and 86 categories that influence the development of network public opinion are obtained. (2) WSR Elements Framework Of Network Public Opinion (WSR-EFONPO) is established. (3) The WSR-EFONPO is explained.

**Conclusion:**

The framework of factors for the occurrence of network public opinion is proposed, and the development process of network public opinion under COVID-19 is sorted out, which is of great theoretical value in guiding the public in the epidemic area to form reasonable behavior.

## 1. Introduction

As a significant public health emergency, the outbreak of COVID-19 in early 2020 has received widespread public attention due to its high infectiousness (1), rapid transmission speed, and difficulty in prevention. Public discussion of the epidemic has been constantly emerging and fermenting, and the spread of COVID-19 has attracted the public's attention. Due to the infectious nature of the outbreak, the government has introduced quarantine measures to control the spread of the disease (2), which has led to online platforms becoming the main channel for people to obtain information about the COVID-19 outbreak (3). Online platforms allow members of the public to closely track epidemic-related information, make personal comments, and express personal emotions about such information (4). The process includes a discussion of online user reviews and a response to offline user reviews, forming the “dynamic public opinion” mode of social networking for online and offline users (5). The public in this model will make the changes in network public opinion triggered by the epidemic more complicated, resulting in the following situations: rumors (6), extreme statements of uncontrollable emotions (7), incessant public opinion (8), blurring of the boundary between the content and form of official news and network public opinion (9), and increasing difficulties for individuals to obtain objective information (10, 11), which may cause a series of negative problems such as excessive personal psychological stress, emotional anxiety, depression, and suicide (12, 13). This may even lead to a social crisis from “online commotion” to an “offline party” (14). When COVID-19 cannot be eliminated in the short term, it is easier for the public to suffer from excessive and sustained stress in the COVID-19 environment in the long term, leading to a deterioration in their physical and mental health (15). It is therefore crucial to investigate the influencing factors and item framework of public opinion response behavior during the COVID-19 epidemic for public behavior intervention.

Currently, in the field of public health, the mechanism of transmission of public opinion on the Internet is primarily studied from the following three dimensions: “hermeneutics,” “psychology,” and “governance.” Hermeneutics focuses on the study of public opinion in the areas of early warning (16), cycle and division of fields (17, 18), characteristics of stages of occurrence (19), and content identification (20), in order to explore the characteristics and evolution of public opinion dissemination (5, 21). The psychological dimension focuses on Internet users in the context of the public health emergency in terms of the psychological-emotional impact on the spread of Internet public opinion (22, 23), infectious diseases, or acute social unrest, which will cause people to become nervous, anxious, fearful, depressed, and adverse (24). The unease would further increase the events related to seeking attention (25). Anxiety, fear, and other negative emotions will accelerate the spread of public opinion (20). Studies have shown that adverse mental health effects have become a serious global public health issue during the COVID-19 pandemic and that mental health issues are more prevalent in China (26, 27). Governance studies the management of network public opinion committed to overseeing and mitigating the trend of public opinion formation (28). Research from the media, the public, and the government finds that the operation of critical points of information diffusion can effectively control the direction of public opinion development (29). Regarding rumors, seizing the golden period and dealing with them can quell the mass outbreak of public opinion (30); timely response and governance of social governance events can potentially improve the effectiveness of public opinion governance (31). Through a combination of the literature above, the hermeneutic dimension is found to carry forward the analysis of the evolution of existing content and features but does not have a link to the source or influencing factors of public opinion. Research on the psychological dimension focuses on the emotions of netizens but lacks the objective elements that produce emotion and the expanded connection of actual behavior following an emotional change. While the governance dimension places importance on the influence of each link on public opinion, the focus is more on the governance of public opinion in particular circumstances and less on the general system of public opinion processing frameworks.

For this reason, this study introduces Wuli-Renli-Shili (WSR) methodology and grounded theory to establish the framework of the elements in the field of public health opinion. The objective factors affecting the development of network public opinion are explored in the “Wuli” dimension, and what material conditions will lead to the generation and fermentation of network public opinion are clarified to expand the research boundary in the field of public health. Analyzing the interaction between people and society in different environments from the “Renli” dimension provides better experimental materials for the study of online public opinion in the fields of “hermeneutics,” “psychology,” and “governance.” In the “Shili” dimension, the unknown behavior laws of the public, the government, the media, the community, and other fields in the social life and natural state under the background of network public opinion are explored to improve the theoretical richness of online public opinion research. The application of grounded theory ensures the authenticity of data extraction and classification, and using the WSR method provides the rigor of logical rise. It is complete, scientific, and reliable, combining the two methods to construct the framework of the elements in the field of public health opinion.

## 2. Methodology

### 2.1. Methods

Zhao and Gu (32) proposed the WSR methodology in the 1990s. They analyze the research object from the perspectives of physics, principles, and human principles and then obtain the material goal and a reasonable mechanistic model of the object. This method, which originates from the research achievements of Qian et al. (33) and other scholars, emphasizes the practice of the system as a material world, a combination of systems, and a dynamic unification of people (34). That is, the analysis of practical activity considers the general laws of material movement and technical action, the ways and laws of intervention and implementation of management by managers of things in the mechanism, and the relevant subjects that determine human collaboration (35, 36). This method holds that there is a kind of hybrid system composed of the natural system, the social system, and some systems involving human society and nature. For the cognition and modeling of this system, “Wuli” must expect to eliminate the subjective factors of human beings and reflect the objective law. “Renli” necessarily expects to eliminate the material factor to reflect the law of man and society; based on “Wuli” and “Renli,” “Shili” must reflect the operation law of a certain kind of “system of interaction between the social system and the natural system.” The specific practice methods and processes of the WSR methodology should be flexibly changed according to the field of practice and the object of investigation. The general working process is as follows: (1) understanding intention; (2) setting goals; (3) investigation and analysis; (4) construction strategy; (5) selection of programs; (6) coordinating the relationship; and (7) realize the idea. The WSR methodology has been widely applied in the areas of energy and sustainability (37), climate catastrophe (38), and the development of software projects (39). Due to its systematic characteristics, it is particularly prominent in the field of risk research, which needs multi-agent collaboration. For example, the WSR framework is used for risk control in the area of nuclear security (40), cost risks in the field of construction (41), and security risks in the field of enterprise management and control (42). However, it is rarely used in the area of public health network opinion, and there are many topics involved in this area. Improper handling of network public opinion is more likely to cause a risk crisis, which is in line with the nature of WSR's theoretical research. This methodology can be used to study the field of public health networks and public opinion. Considering that WSR methodology is rarely studied in the field of network public opinion and that it is difficult to establish a research framework in the initial stage, grounded theory is introduced in order to conduct ancillary research. In addition, grounded theory and WSR methodology have certain correlations (43), so it is reasonable to use them to study network public opinion.

In 1967, Glazer and Strauss proposed a grounded theory approach. It is a qualitative research method that adopts a “bottom-up” analysis of research objects to explore the nature of things (34, 44), with a focus on the creation of a theoretical framework or inductive analysis of data (45). The research stages of grounded theory are as follows: (1) open coding stage, researchers are required to keep an open mind, try their best to “suspend” personal “prejudices” and “stereotypes” within the research community, and code all the data according to their status. It is an operational process of breaking up collected information, giving it concepts, and then reassembling it in new ways. The purpose of coding is to discover conceptual categories from the data, name the categories, determine the attributes and dimensions of the categories, and then name and categorize the phenomena under study. (2) The main task of axial coding is to find and establish various connections between concept categories to show the organic association between various parts of the data. These connections can be causal, temporal, semantic, situational, similar, and other different relationships. Researchers only conduct in-depth analysis on one category at a time, looking for correlations around this category, which is called the “axis.” (3) Selective coding refers to selecting a “core category” after systematically analyzing all of the discovered concept categories. The subcategory coding is continuously focused on those codes related to the core category. The core code must be repeatedly proven to be dominant in comparison with other codes and able to include the most research results within a relatively broad theoretical scope. (4) The saturation test solves the step of when to stop collecting data. When no new research content can be found during the analysis of the collected data, the theoretical saturation is reached, and the data collection can be stopped at this time. Theory formation relies on a large number of data analyses. This places higher demands on the researchers' ability to make objective judgments and their research experience, and there may be subjective bias in the research process (46). To reduce the subjective influence to a certain extent, other theories can be used for guidance. The combination of WSR methodology and grounded theory used in this study can reduce root-orientation errors caused by missing data and lack of experience and improve the attribution of various Wuli-Shili-Renli (47). Not only can it clarify the focus and objectivity of grounded theory, but it also extends the contribution of WSR methodology to the realm of network public opinion, particularly in exploring network public opinion in COVID-19's isolated areas. Since there are few theoretical frameworks related to this type of research, grounded theory can be used for the research (48).

This study collects public opinion comment data related to the epidemic on the Weibo network platform, conducts grounded analysis, and combines WSR methodology to summarize the transmission and public response factors of network public opinion under the epidemic form in order to construct the framework for the epidemic public opinion research system. Through qualitative research into the conceptualization and categorization of user feedback, taken together with the research findings and the reality, the framework for the network public opinion response mechanism of COVID-19 under WSR is concluded.

### 2.2. Research process

**Figure 1:**
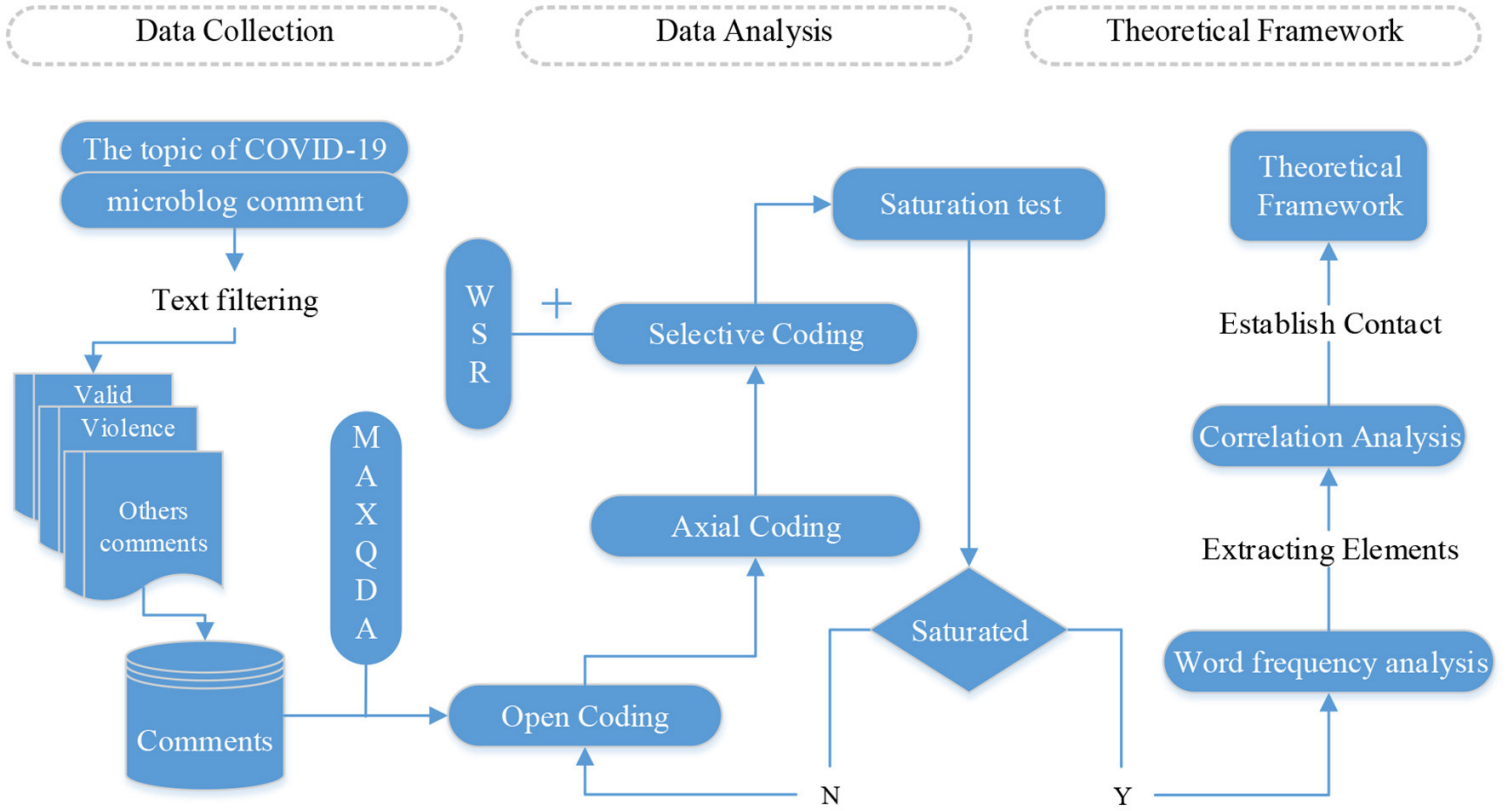
Method flowchart.

This study takes user feedback on epidemic-related events under the Weibo network platform as our research material, including online commentary from relevant bloggers and public opinion event experts. Microblogs are mass platforms for exchanging, sharing, and disseminating information based on user relationships (29). The system has the characteristics of rapid updating of comments, open communication content, and timely updating of information. Taking Weibo as a platform for data collection allows us to understand better relevant remarks from different ages, audiences, and circles and to capture the values and ideas users want to convey. First, this study used grounded theory to analyze the comments, combined with the WSR methodology, applied MAXQDA to conduct a grounded analysis of the elicited data, and obtained relevant categories in the open coding phase. In the main axis coding stage, categories are classified and logically inferred to the core category. The selective coding stage combines WSR methodology to show the correlation between basic categories. Finally, saturation testing was performed, and data were collected if the saturation testing standard was not met. Based on data saturation, a word frequency analysis of the categorization coding was conducted to identify elements of the theoretical framework for the public's response to the outbreak. A correlation analysis of individual categorization coding was performed to clarify the relationship between items according to the WSR methodology, and a grounded theory of the mechanical framework of the network public opinion response to COVID-19 as part of the WSR was obtained. The method flowchart is shown in Figure 1.

### 2.3. Data collection

**Figure 2:**
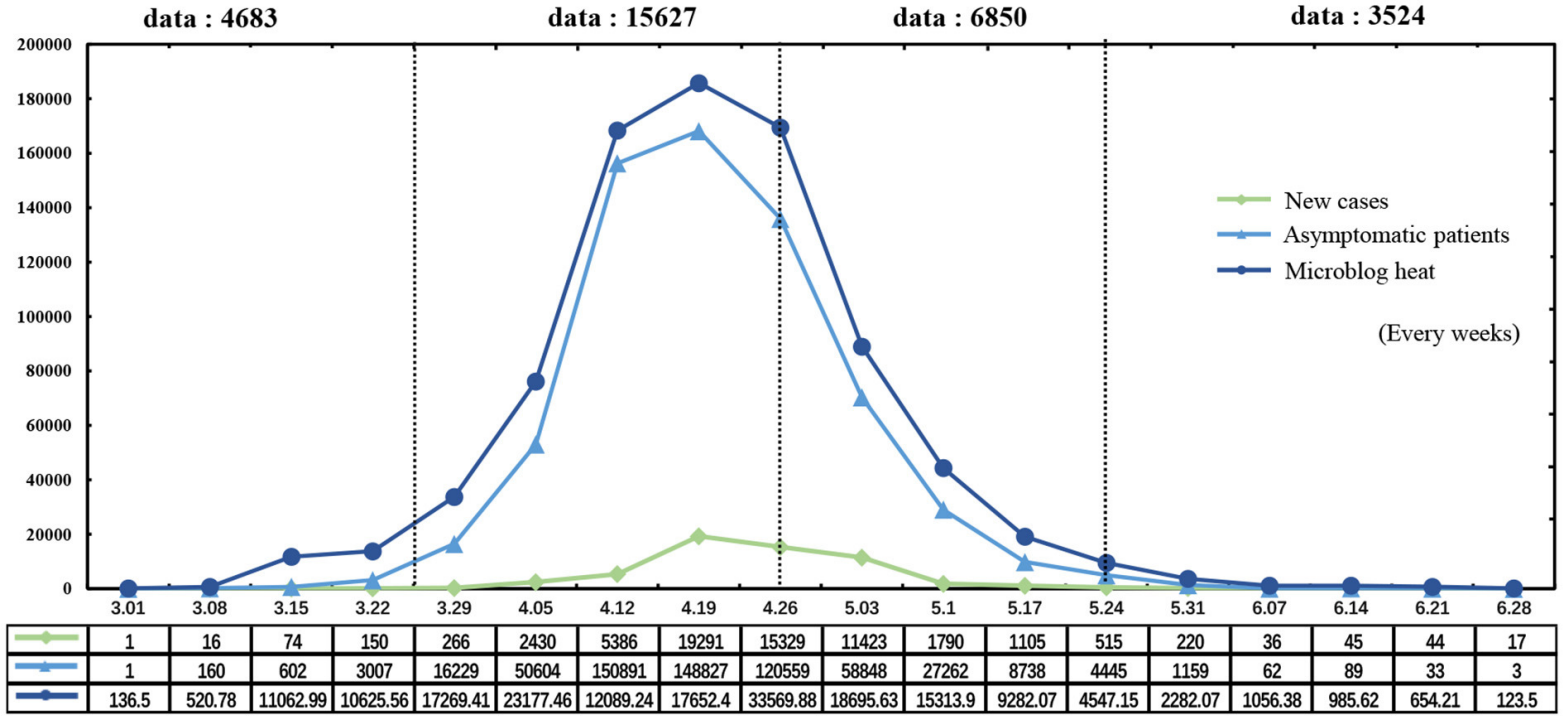
Data collection chart.

The data collection phase used the Web Scraper plug-in under the Chrome website, and the place of the epidemic was used as the keywords, such as “Shanghai epidemic” and “Shanghai epidemic conference.” The hot topics were chosen as the data source through the chain expansion of users' online comments. Data were selected by week and focused from 1 March 2022 to 28 June 2022. Figure 2 shows how the popularity trend of microblogs was displayed as a function of the number of local COVID-19 cases and local asymptomatic individuals in Shanghai (Data on the number of patients with confirmed COVID-19 and asymptomatic patients were gathered from the Shanghai Health Commission, and popularity data for Weibo were collected from the attention index of Weibo popularity, and popularity was displayed as actual popularity/100). Videos of outbreak events during the corresponding period and user comments under posts were obtained to investigate public sentiment, attitudes, and opinions. The total number of comments captured was 30,684. MAXQDA software was used for the qualitative analysis; concepts were extracted from the comments, and concepts were summarized and sorted to carry out the grounded theory coding process at three levels. The criteria for data collection were as follows: first, the comments were substantial and belonged to the scope of public opinion; second, there was no reactionary, obscene, or malicious information; and third, the content of the review has no repetition or other features. Finally, 6,579 original comments data in Supplementary Table 1 were obtained. The 6,379 comments data were selected for root-code analysis, with the remaining 200 comments serving as test data to detect theoretical saturation.

## 3. Data analysis

In this study, the network public opinion related to the epidemic is taken as the research object, microblog comments are taken as the research basis, and the data of the following four stages are analyzed by grounded theory. First, open coding, conceptualizing, and categorizing the textual content of the original data. Second, axis coding, deepening, and summarizing the category formed in the first stage forms the main category. Third, in selective coding, the relationship between the categories is split, and the core category is included in the core category of the WSR dimension. Fourth, if not saturated, the theory saturation test continues to collect data and cycles through stages of operation. If data saturation is reached, proceed to the next stage. Fifth, word frequency analysis of category coding is performed to obtain the necessary components of theory building. Finally, to form the framework of network public opinion response behavior during the pandemic, this study introduced correlation analysis from Vosviewer software to establish the relationship between codes.

### 3.1. Open coding

Transforming raw data into representative codes requires conceptual analysis of the raw data, concept definition, content summarization, and other operations, all of which must be accomplished in the open coding phase. There is no researcher bias when conducting coding research, allowing initial concepts in the raw data to emerge naturally. Thus, the coding results are formed in order to avoid the influence of subjective reflection and end up with coding results that are objective, free of bias, and close to the meaning of the data itself. Otherwise, there will be a large gap between the final grounded theory obtained and the realistic results (49). In this study, open coding is based on relevant standards. The raw data of user comments and online reviews on epidemic-related matters is generalized, summarized, and merged without subjective thinking in the data coding process. The analysis results are related to “epidemic,” “material,” “safety,” “people,” etc. The events around the center of the epidemic get more attention from users. Through the distillation of comments and repeated analysis, a total of 6,579 comments and texts were coded. There were 1,243 conceptualization results and 86 category results in the corresponding category column in **Table 2**; the details are in Supplementary Table 2; part of the conceptualization process is shown in Table 1, and all of the category results are shown in Table 2.

**Table 1:** Partial categorization process.

**Category**	**Categorization**	**Comment**
Anti-epidemic measures	Epidemic control, personnel gathering, etc.	As a closed community, Shanghai Jing'an District, Haifeng Road, Lane 100 Jurong District has been unmanaged, and even people run drugs. Positive does not pull away; all kinds of people in the community gathered, not wearing masks, chatting, or smoking
Medical resources	Seek medical attention, medical help, etc.	Even if you are infected with the new crown pneumonia, the people who buy antipyretic drugs, cold medicine emergency ah, even 120 can not go
Material distribution	Food price fluctuation, material distribution, etc.	We are living this life now! People are spending money to buy high prices; many are terrible dishes, and even nasty dishes have to buy high prices
Information resources	Hardware technology, digital technology, etc.	Nucleic acid do it; the great white said the system crashed, let everyone go back temporarily
Special populations	Special groups, objects in need of help, etc.	Shanghai stipulates the flow of maternal visits during the epidemic, and just a statement will not work; there must be coordination
Security events	Inequality in epidemic prevention, safety incidents, etc.	Stars have the privilege of being able to openly dine in and apologize for not even one, while many ordinary people cannot eat now

**Table 2:** Axis code table.

**Main category**	**Meaning**	**Corresponding category**
Government behavior	The way the government dealt with the epidemic affected the direction of public opinion.	Government control Regulatory authorities Legal constraints Government credibility Prevention and control measures Departmental linkage Policy realization information notification
Media behavior	Media influences people's attitudes, and as a place for emotional fermentation, it speeds up the spread of public opinion.	Rumor elimination Delete hotspot Retweet Respond Ignore
Demand-side behavior	The differences in demand cognition, preference, and urgency of required services on the demand side during the epidemic event affect the spread of network public opinion on the epidemic event.	Demander awareness Non-urgent needs Daily needs Urgent needs Objective remarks Positive remarks Negative remarks
Solution-side behavior	The quality and behavior of the problem solver is the response to the epidemic event service, which affects the actual perceived effect of the demand side, and then affects the public opinion dissemination of the epidemic event.	Understanding willingness Incident resolution time The way to solve the incident Incident feedback Security guarantee
Community party behavior	As the first line of handling epidemic events, the community side affects the actual perception of the public and is easy to generate the public opinion center of epidemic events.	Anti-epidemic measures Rescue services Emergency response Community action Internal and external coordination information exchange Material distribution
Operation mechanism	Since this is the norm of each subject's activities, the actual operating effect of the operating mechanism is also affected by the perception of the information disseminators.	Detection rules Isolation rules Protection rules Payment rules Rescue rules
Coordination mechanism	It coordinates various organizations to reach consensus, promotes diversified coordination, defends the epidemic and cleanses public opinion.	Time loss Coordination behavior Emotional attrition Stakeholder Fee payment
Management mechanism	Improve the management mechanism of the epidemic event processing platform, and reduce the aggregation of negative comments by improving the management methods.	Service attitude Management model Supervision strength Epidemic propaganda Public services Emergency planning Medical protection
Responsibility mechanism	The responsibility of the epidemic event should be divided, and the roles of service providers, communities, governments, and the public should be established in handling the event.	Inconsistency between authority and responsibilities Responsibility shirked Complaint handling Notification of punishment Responsibility Implementation of the solution Reward publicity
Communication mechanism	Improve the communication channels between the public and service providers, ensure the integrity of information transmission, and avoid the bias of public opinion caused by false information and lack of information.	Information dissemination Individual communication Information uploading Information linkage Information discovery Information feedback Information identification
Event properties	The occurrence of the epidemic is the source of public opinion dissemination, and the attributes presented by the event trigger the scope of public opinion dissemination.	Rescue incidents Security events Health events Epidemic prevention events False events
Material resources	The occurrence of network public opinion during the epidemic event manifests the contradiction of material distribution, which is the material basis of public opinion fermentation.	Medical resources Life resources Security resources Information resources Human resources
Epidemic status	The number of confirmed cases of the epidemic is the main event factor leading to the spread of public opinion on the epidemic event, which intensifies the direction of the evolution and development of public opinion on the epidemic event.	Number of infections Number of asymptomatic people Number of deaths Growth scale
Individual perception	The risk of events and the impact degree of individual life presented under the epidemic events and the public opinion on the actual life perception of users has increased or decreased.	Safety perception Infection risk perception Perceived risk of death Perception of experience Outcome perception
Crowd environment	Under the isolation of the epidemic, the population's environment affects the personal life experience and the emotional development direction of the network public opinion.	Occupational characteristics Community relations Family characteristics Special populations

### 3.2. Axis coding

Axis coding, the categorization results obtained through open coding, is further discussed, analyzed, and summarized; the logical relationship between the open-ended results is explored; and the main category is obtained through axis coding. First, the nature and level of the initial category are determined, and then the logical relationships among the different categories are established through a continuous comparison between the initial categories. The specific steps are defined as follows. (1) The initial 86 categories were analyzed, and initial categories with similar meanings were categorized. (2) According to the “Wuli-Shili-Renli” perspective, the subcategory is collected into the main category at a higher level. (3) The significant categories obtained were compared with relevant government research standards, the media, and the community in the epidemic research literature and modified and refined to ultimately form 15 main categories, as shown in Table 2.

### 3.3. Selective coding

**Figure 3:**
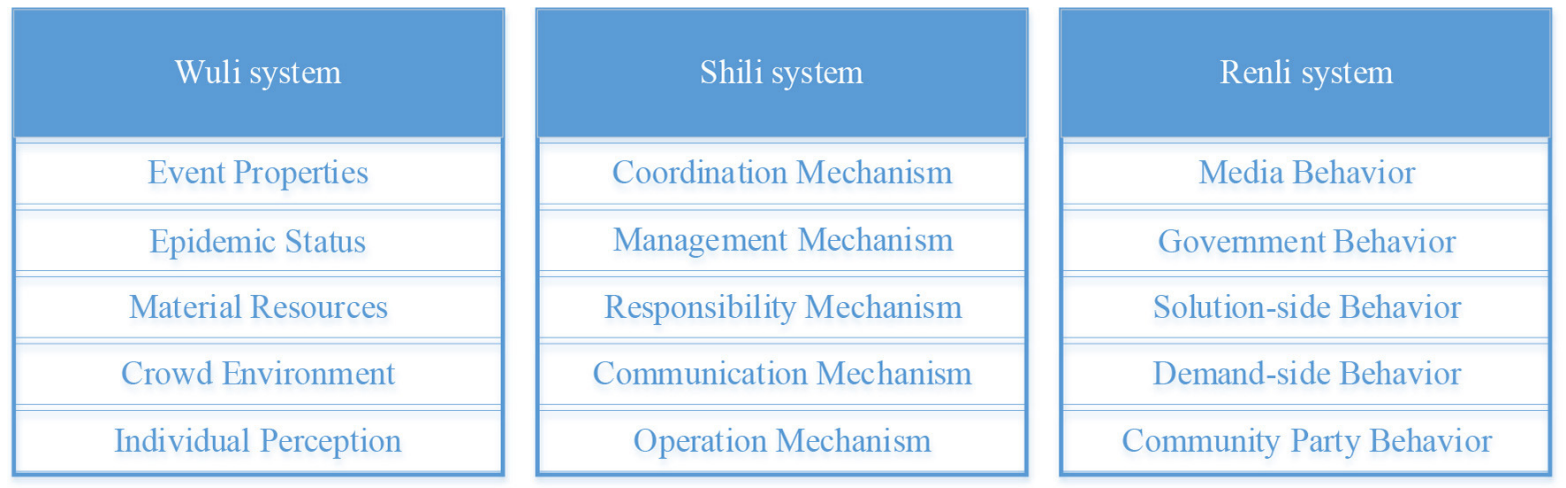
Core category.

“Selective coding builds on axial coding, within established categories, by clarifying storylines to tease apart and uncover core categories that are both comprehensive and dominant, and organically relate basic categories to other categories, and then construct a new theory (50).” A bottom-up summary of the original material is based on facts and experience. However, theory directly forms a sizeable logical jump from the data, and there is a disconnect between theory and experiment. To ensure the effectiveness of the construction of the grounded theory structure, the grounded theory and WSR methodology are combined to ensure a solid research foundation and a rigorous logical relationship. It makes the connection between specific data and various factors traceable, and these factors can be located in a specific Wuli-Shili-Renli dimension. In the core category coding stage, the core category is categorized within the framework of “Wuli-Shili-Renli” analysis, which can highly theorize interpretive data and extract the core categories of Wuli-Shili-Renli dimensions affecting public opinion communication. As shown in Figure 3, the objective objects of the study of the Wuli dimension are the objectively existing material carriers and the environmental risks during the COVID-19 pandemic development, including significant categories of event properties, epidemic status, material resources, a crowded environment, and individual perception. Operational coordination mechanisms, management mechanisms, responsibility mechanisms, communication mechanisms, and operation mechanisms are categorized in the Shili dimension, and media behavior, solution-side behavior, government behavior, demand-side behavior, and community party behavior are categorized in the Renli dimension. The ecosystem affected by COVID-19 network public opinion comprises the three essential category factors, and the typical relationship structure between them is given in Table 3.

**Table 3:** Typical relationship structure.

**Typical relationship structure**	**Relationship structure connotation**
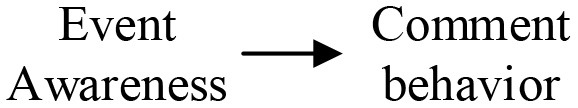	The perception of hot events affects the public's online speech and communication
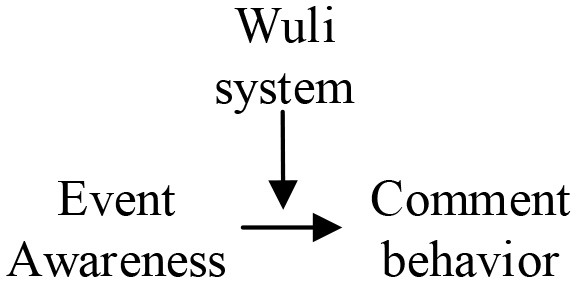	The Wuli factor is the influence of the surrounding environment that the public can directly contact public opinion. Event properties, material resources, epidemic status, individual perception, and crowd environment constantly affect public life, change public cognition and influence online comments on events
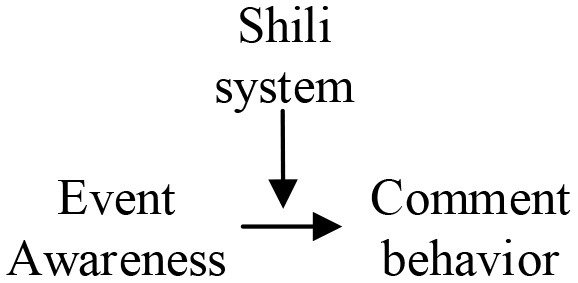	The Shili factors affect the development direction of public opinion communication. coordination, management, responsibility operation, and communication mechanisms can influence public opinion development trends. Meanwhile, the cognitive degree of media factors affects public comment behavior's positive and negative tendencies
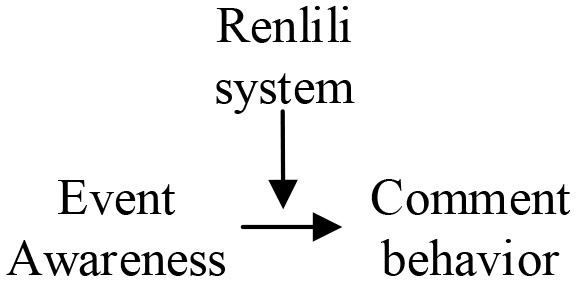	The Renli factor is the top-level design. The behaviors of the government, the media, the demand-side, the solution-side, and the community party are coordinated in multiple ways, which jointly affect public cognition, reduce radical and provocative comments and establish a sound public opinion ecology

### 3.4. Saturation test

Using the original data as the test object, the theoretical saturation test verifies the reliability of the coding results of the grounded analysis. In this study, 200 comments data were tested for theoretical saturation according to the same criteria, and multiple researchers were also used to code and verify them to ensure the rigor of the test. This study presents five comments from 200 test data:

Comment 1: “I do not understand. You can send two police officers but cannot send professional personnel to do a nucleic acid test. Is it a crime to be a citizen? The commentary focuses on prevention and control measures, which belong to the main category of government agencies, namely policy exchange. It can also be considered the core category of material resources of the Wuli system, namely human resources.”
Comment 2: “Shanghai strictly controls the ‘three zones.’ I know that even the zones are limited to this area. How can they get out of Shanghai? It was impossible to get to the station; public transport was suspended, no taxis, and cars could not travel without a pass. The comment focuses on the city's traffic problems, which fall under the category of government agencies, namely government regulation. It can also belong to the core category of coordination mechanism of the Shili system, namely information linkage.”
Comment 3: “Who knows if the WeChat video account is not allowed to post the actual situation of the epidemic in Shanghai? I do not see it. This comment focuses on the real situation of the epidemic and belongs to the main category of media behavior, namely Respond.”
Comment 4: “When the epidemic falls on others, one's positive energy becomes a ‘concentration camp’ when it falls on one's head, and the positive energy becomes created by the media. The logic of these people is really. This comment focuses on the voice of comments, which belongs to the main category of media behavior, namely rumor elimination.”
Comment 5: “There are also a group of people in Shanghai who are either going to milk tea shops or going out to drink coffee, which is the aroma of coffee in epidemic prevention and control. This comment focuses on the implementation degree of epidemic prevention in the community, which belongs to the main category of community party behavior, namely Anti-epidemic measures.”

Theoretical saturation test results show that the test data are consistent with the grounded analysis above. The categories of network public opinion influencers in epidemic areas discussed in this study have been quite rich, and no new category information has been discovered in the testing process. As a result, the category of influencers of network public opinion about this event has reached saturation.

### 3.5. Word frequency analysis

To obtain the item composition of the research framework, this study performs a radar display of coded categorical word frequencies based on the determination of theoretical saturation. Specifically, the operation process proceeds as follows. First, saturated category coding is compared to the original data. Second, the frequency of category coding is analyzed. Finally, the radar distribution map displays the word frequency of the category encodings from the Wuli-Shili-Renli.

**Figure 4:**
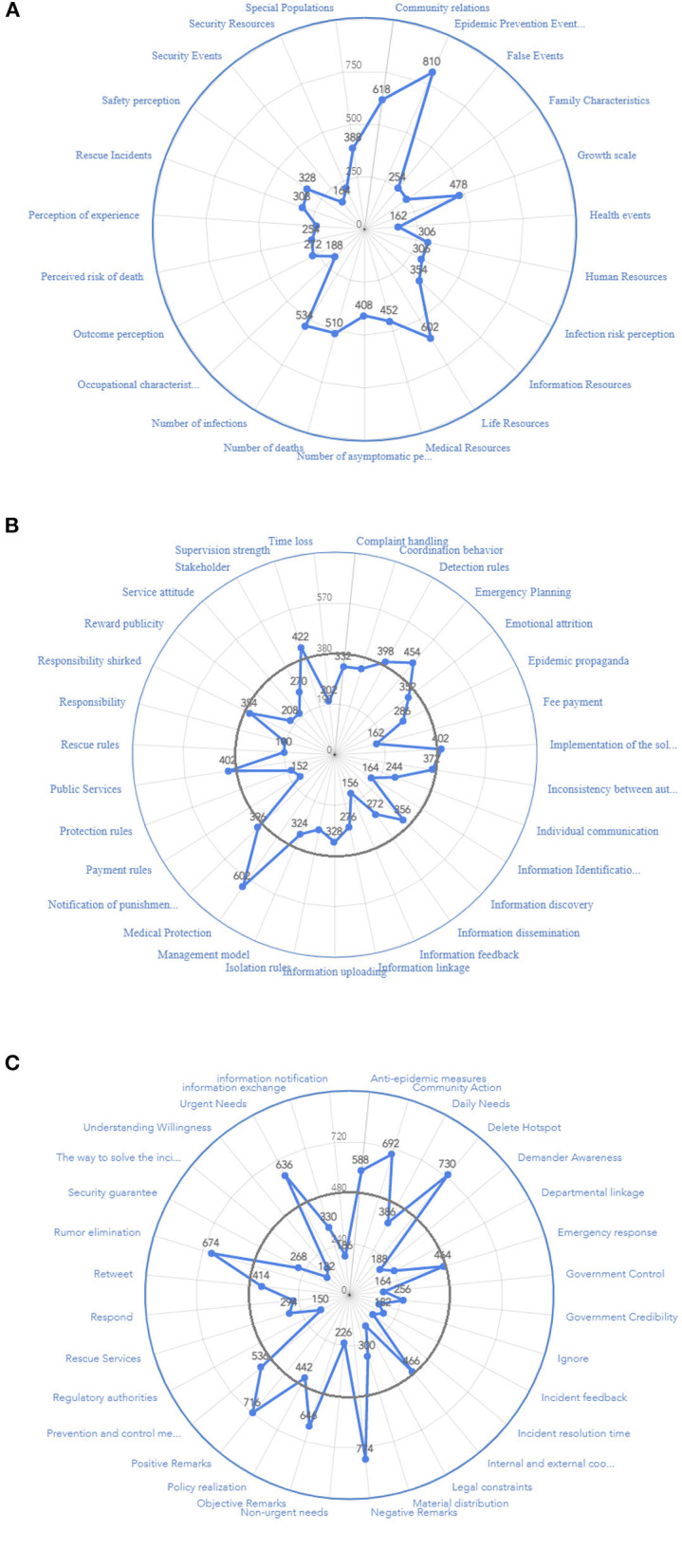
Word frequency analysis. **(A)** Wuli, **(B)** Shili, and **(C)** Renli.

In the word frequency distribution diagram, Figure 4A, under the main category of the Wuli system, material resources, epidemic status, and individual perception. The frequency of occurrence of the subcategories is similar, and the frequency distribution is 100–500. Moreover, there are no prominent rare subcategories. It can be considered that material resources, epidemic status, and individual perception can compose frame elements. The codes in the categories of event properties and crowd environment, epidemic prevention events, and community relations appeared 618 and 810 times. It can be considered that event properties and crowd environment play more critical roles in the composition of framework elements. In Figure 4B, under operation mechanism, coordination mechanism, management mechanism, responsibility mechanism, communication mechanism, the frequency of each communication mechanism subcategory is around 500. Only medical protection belonging to management mechanism has a slightly higher frequency, but the frequency gap is small. Therefore, it can be considered that the system's main categories can also constitute the framework in Figure 4C. Rumor elimination, urgent needs, internal and external coordination, and other codes frequently appear in the Renli system, which is distributed around 700. Therefore, the Renli system's main categories can be government behavior, media behavior, demand-side behavior, solution-side behavior, and community party behavior. These five main categories as framework elements have sufficient conditions and are highly important. This study concludes that the main categories established by grounded theory have a solid logical basis and a sufficient realistic foundation as elements of the COVID-19 network public opinion response framework, which can be used to set up the COVID-19 framework for network public opinion response.

### 3.6. Correlation analysis

While the above analysis of word frequency has clarified the framework's elements, the relationship between the elements needs to be further explored. To explore the correlation between these elements, considering that some of the collected comments have a wide range of meanings, and that a single code can reflect the main views of the reviewers, there needs to be a more comprehensive understanding of the reviewers' views. Then the subcategories formed by open coding are taken as the standard to expand the content of the comments and form a word list. The keyword analysis method of VOSviewer is used to show their relevance. This operation can comprehensively understand the reviewers' opinions and establish the connection between relevant elements. The specific operation is defined as follows. (1) The content of the comments is analyzed in order to determine whether it is extensible. (2) The comments content that is not expansible, that is, the comments that can be summarized entirely by a single subclass, is not included in the research scope. (3) Comments with extensibility can be expanded according to the standard to form a word list and visualized by VOSviewer. For example, the content of the text is “the nucleic acid is doing, the medical staff said the system crashed, let everybody go back temporarily.” The subcategory formed by open coding allows for the generation of the “information resources, emergency planning” keyword table. “Shanghai has stipulated the flow of medical treatment for the mother during the pandemic. One statement is not enough, and coordination is needed.” Can produce “Special Populations, governmental control, internal, and external coordination” table of keywords. Furthermore, the single keyword “positive remarks” generated by the textual content “Cheer up Shanghai” is not included in the content of the analysis. Using the above research on the Wuli system, Shili system, and Renli system under the main category of related relationships, the relationship between elements of each other is clarified and the relationships between the basic categories are summarized.

**Figure 5:**
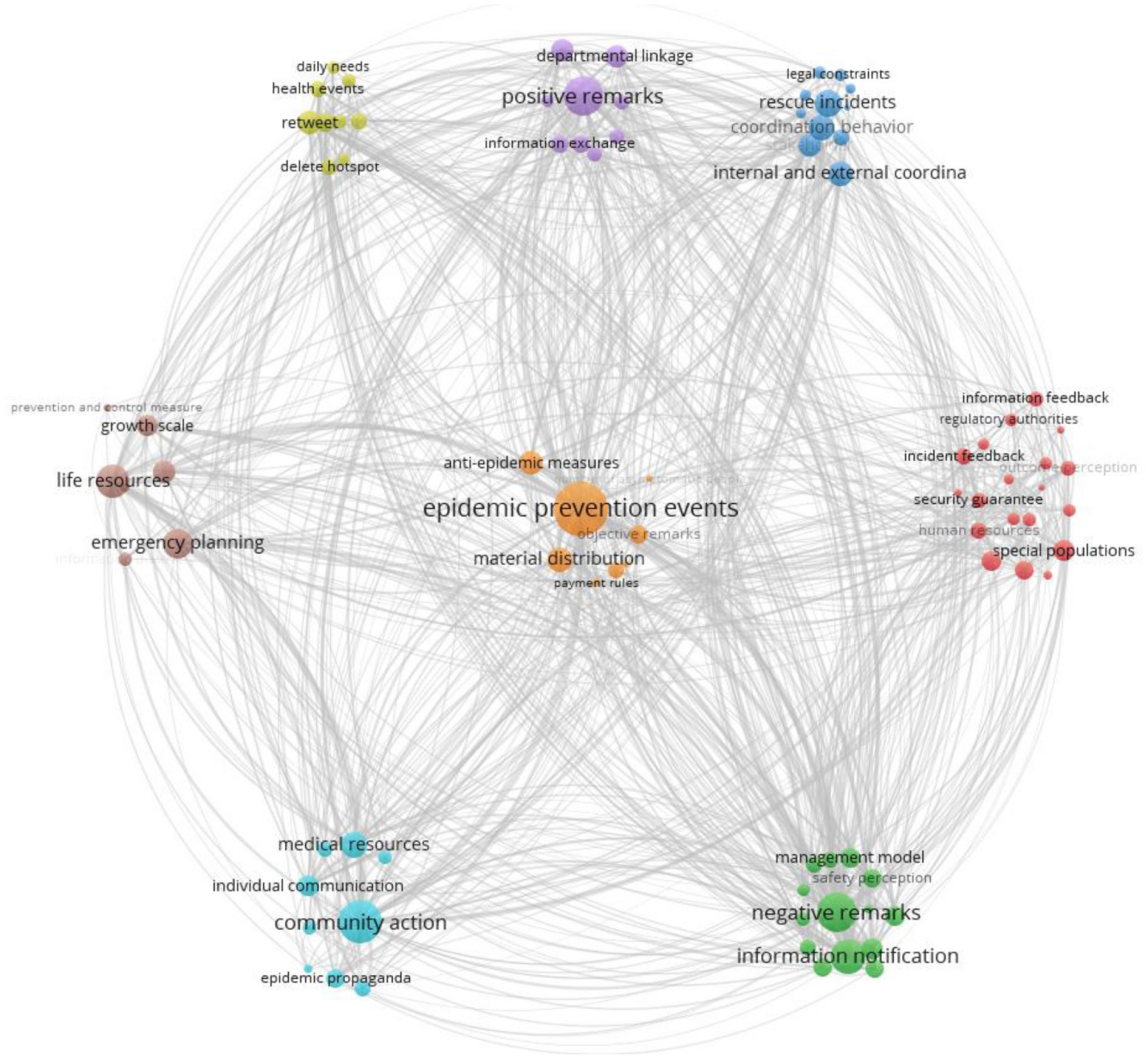
Category correlation diagram.

Through the establishment of the text data keyword table, the data were imported into VOSviewer for correlation analysis of the existing 86 sub-categories, and eight clusters of relations were generated, as shown in Figure 5, in which all categories under Wuli-Shili-Renli systems were reflected in each cluster to clarify the relationship between the major categories and further reflect the path relationship between the core categories formed by grounded theory.

**Figure 6:**
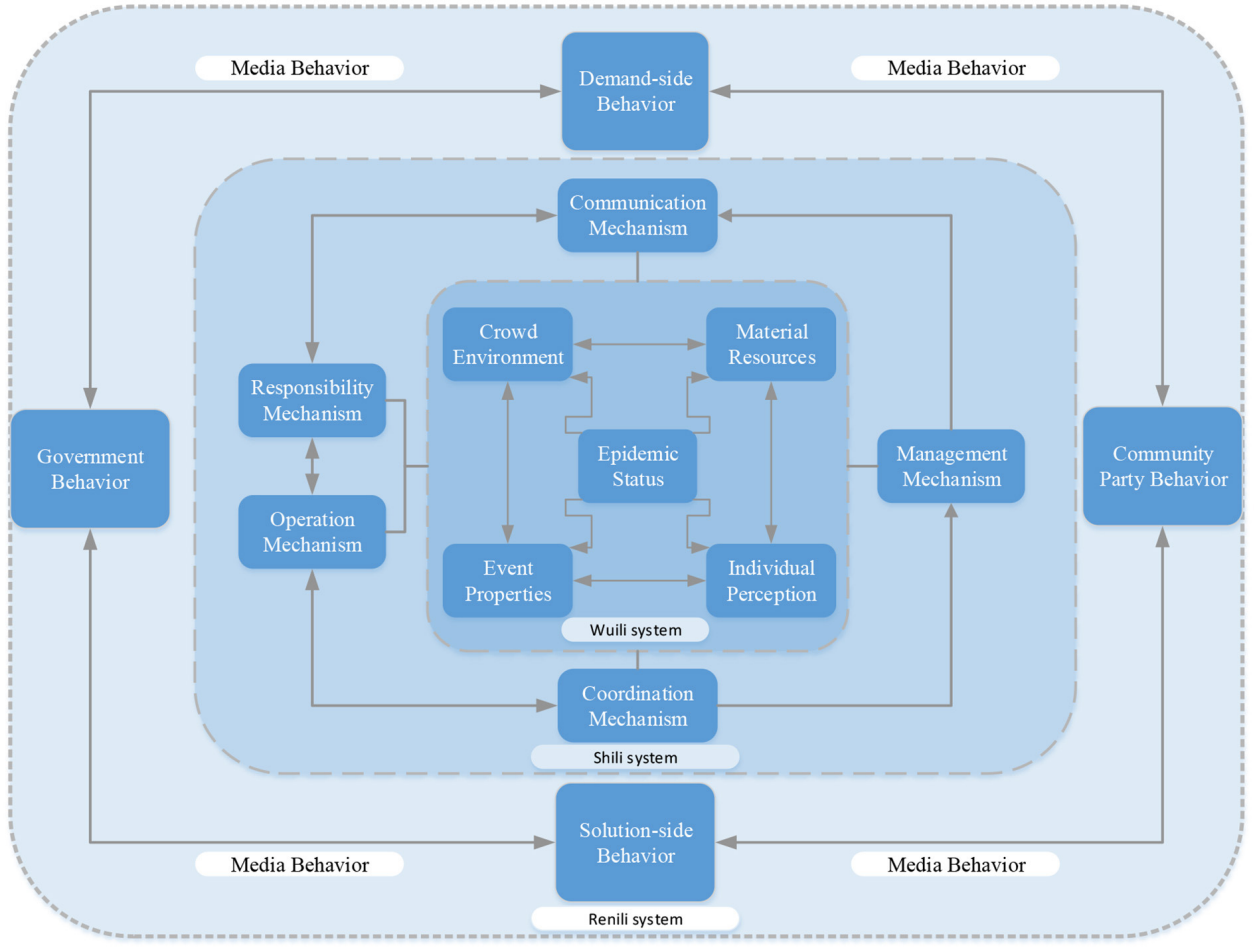
WSR Elements Framework Of Network Public Opinion (WSR-EFONPO).

This study attempts to establish the framework from Wuli to Shili and Renli, as shown in Figure 5. In the core category dimension of the Wuli system, Wuli categories such as “epidemic prevention events,” “life resources,” and “rescue incidents” are closely related to each other. Categories such as “positive remarks” and “negative remarks” have also attracted much attention. The above categories are not only associated with Shili categories, such as “internal and external coordination,” “information feedback,” “delete hotspot,” but also with “regulatory authorities authority,” “management mode,” “information exchange,” and other Renli categories. Wuli category is related to the Shili category and the Renli category, forming a diffusion trend from the Wuli system to the outside (51). In the core category of the Shili system, “department linkage,” “information notification,” and other factors have been the focus of Internet users (52). The category of the shili system is closely related to the Wuli system. However, it also connects to categories in the Renli system, such as “community behavior,” “government credibility,” and other categories. Therefore, it can be considered that the Wuli system connects the preceding and the next in the whole framework and plays the role of buffering and connecting the system (53). In addition, “community action,” which is included in the core category of community behavior, is gradually affecting the behaviors of the government, media, demand-side, and solution-side, resulting in a diversified link to the community party as Renli's center of public opinion (54). Thus, the Wuli factor can effectively be thought of as the influence of the surrounding environment that the public can directly communicate with public opinion, the Shli factor affects the direction of communication and the development of public opinion, and the Renli factor is the intermediary link. Multiparty collaboration jointly influences public cognition, giving rise to the relational structure shown in Figure 6 and the item framework diagram for public opinion occurrence.

### 3.7. WSR framework for network public opinion on COVID-19

In light of the above discussion, this study takes the main categories belonging to the core categories of Wuli-Shili-Renli as the components of the grounded theory framework, including the following:

Wuli: communication mechanism, event properties, material resources, epidemic status, individual perception, crowd environment.
Shili: operation mechanism, coordination mechanism, management mechanism, responsibility mechanism, communication mechanism.
Renli: government behavior, media behavior, demand-side behavior, solution-side behavior, community party behavior.

Since events are spread through the network, the primary category of media behavior in the Renli system is the background element. In the connection between the Renli dimension and the Shli dimension, government behavior is more closely related to operation mechanism and responsibility mechanism. Demand-side behavior, solution-side behavior, and community party behavior are closely related to the communication, coordination, and management mechanism, so the design, as shown in Figure 6, is produced. The design form is a whole in the connection between the Shili dimension and the Renli dimension because the number of codes is similar.

To sum up, this element framework reflects the emergence of public opinion from the Wuli system, coordinated through the Shili system, transmitted to the Renil system, and returned to the Wuli system through multiple coordination. These are related and affect one another, forming the WSR Elements Framework Of Network Public Opinion (WSR-EFONPO) (Figure 6).

## 4. Discussion

This study constructs WSR-EFONPO in the context of the regional epidemic. From the point of view of the logic of generation, the public formed a state of interaction through the “Wuli-Shili-Renli” system, but its internal mechanism needs further analysis, which will be discussed in the following sections.

### 4.1. Individual formation reaction process of the WSR system

The Wuli factor is an objective existence that is not subject to the will of man, and it is the basis for Shili and Renli to play a role in the network public opinion dissemination of regional events. In this study, Wuli factors are thought to underlie individual responses. In the Wuli system, direct impacts on individual life include communication mechanisms, event properties, material resources, epidemic status, individual perception, and crowd environment. Moreover, because of the restriction of isolation measures, people's ability to secure resources and deal with crises is significantly weakened, which results in the increased influence of Wuli factors on individual lives (55). In this way, objective conditions for individual network discourse are generated, and then the logical basis for the occurrence of public opinions online is formed. Such as “a long-term lack of biological resources in the community, the network to seek material assistance” is to follow the individual life resources of the Wuli system due to the isolation of the epidemic cannot be obtained in time, i.e., the logical path to seek help on the network.

As a mechanistic link of network public opinion diffusion and development, the Shili factor emphasizes the management of organizations and the construction of institutions and believes that individuals stimulated by the Wuli factor generate demands through network expression to obtain help from organizations or institutions. Operation mechanism, coordination mechanism, management mechanism, and responsibility mechanism always impact the interaction between individual needs and demand solutions. Because the Shili factor is the logical and practical norm of event resolution, the ability of the crowd to understand and cooperate with it in the problem-solving process is a test of the level of design of the material framework (56). Whether or not individuals agree with the Shili factor in the interaction process can be seen as the critical link of individual discourse in forming network public opinion. The Shili factor of individual identification may help resolve the demand, i.e., individual comments may produce more objective comments in the formation of public opinion online without causing the diffusion of public opinion. Individuals negate the Shili factor to resolve demand, i.e., individual comments are more likely to attract attention in the network's public opinion, resulting in public opinion propagation.

Humans play an important role in practical activities, and Renli is an essential criterion for practice that emphasizes how to guide multiple relationships between individuals and groups. If the individual fails to address objective needs, the individual role will be transformed into the demand-side role, and the organization will help solve the problem. In this process, the individual needs are conveyed to the Renli system through the Shili system and finally realized. Under the roles of the government side, community party, demand-side, and solution-side of the Renli system, demand generation and solution are completed, a complete understanding of individual events is formed, and a complete framework for the occurrence of public opinion is formed. In epidemic isolation, prevention, and control, all kinds of life and production activities need people to carry out production activities. Links between governments, communities, and individuals are critical during this period, as they involve complex information transmission, coordination control, safety, and security. In addition, the Renli factor is a behavioral guide that guides the interaction between individuals and groups. In the end, the satisfaction degree of the whole activity process is reflected in the problem-solving situation of the demander, and the network identity behavior of the individual concerned is produced by a similar network sense of the event, which is manifested as network vocalization.

### 4.2. Individual differential responses under the WSR system

Individual differential response behavior originates from the Wuli system. It is essential for the people who live in the community whether the community can provide adequate basic living resources. Adequate rumors refuting for nucleic acid testing and material distribution. Sufficient medical resources to help the sick group and unobstructed information resources to communicate individuals' needs will impact individuals' behavioral responses (57). The following incidents occurred in communities where supplies were relatively scarce, resulting in individual emotional reactions and changes in network public opinion. For example, “nucleic acid testing was not timely, and positive cases were concentrated in the community,” “supplies were not procured properly, and community supplies piled up and rotted,” “Children had a fever and could not buy antipyretic drugs,” “elderly people lacked medication for heart disease, which led to death,” and “120 was called for resuscitation and was not allowed to enter the community, missing the best time for resuscitation.” In communities with sufficient material resources, individual perceptions, and the crowd environment positively impact individuals. Sick people are rescued in time, and emergencies are solved in time. Moreover, some communities even become “cigarette communities” and “Coke communities,” using cigarettes and Coke to some communities have become “cigarette communities” and “Coke communities” where cigarettes and Coke are exchanged for supplies. This results in a harmonious overall community environment and, subsequently, a more positive online presence. However, in communities with general material resources, especially in rescue and epidemic prevention incidents, individuals are more concerned about material resources and focus on solving the problem, such as “when will the food be distributed? There is not enough food in the house.” “There is a gas leak in the house. How long do I have to wait for the repairman?” At this time, the inability to effectively respond to the supply of material resources will create conditions for the fermentation of community members' online opinions. More users who get effective responses will still maintain an optimistic attitude and wait for the problem to be solved.

Individualized differential response behavior stems from the Shili system mechanism. The Shili factor, which involves organizational management and institution building, is involved in the event processing stage after demand formation, and untimely processing or unsatisfactory results can cause fluctuations in public opinion. In the case of the temporary treatment center, for example, although individual needs were met, the following situations caused the loss of individual lives and property, leading to confusion in public opinion. For example, coordination at the matter level was not timely, management mechanisms were unclear, and responsibilities were not clearly defined. In a community elderly rescue incident, communication mechanism problems such as information propaganda, information communication, and information discrimination caused the situation of elderly prevention to get out of control, which in turn triggered a hot debate on the Internet. Some comments slammed the community Wuli system as inadequate, while others decried the Renli system as uncoordinated. These comments illustrate how problems at the Shili level can be transferred to organizations or individuals, focusing the problem on the Wuli and Renli systems. Individualized differential response behavior originates from the Renli system.

The relationship between the individual and the group is a problem to be considered at the Renli level. The individual needs caused by the epidemic quarantine require more coordination among multiple subjects to complete, starting with objective material at the Wuli level, passing through the Shili level, and finally intersecting at the Renli level through multiple subjects. For example, the quarantine policy in Shanghai, where the closure of the city was not implemented at the beginning, led to the rapid development of the scale of the epidemic, which eventually made the public question the behavior of the government side. In a district, the elderly did not wear masks and gathered in groups, resulting in multiple diagnoses and making community epidemic prevention chaotic. Netizens began to question the behavior of the community side. Similarly, the Shanghai Weibo hot search, taken down within half an hour, made Internet users question the media for covering up the truth. It can be seen that the needs and conflicts at the Wuli level and Shili level will be transferred to the Renli level because in the process of demand generation and solution, individuals turn into demand parties through their needs. Organizations or groups have the resources to become solution parties. Through coordination between government parties and community parties, the response to demand parties and the arrangement of solutions will affect the feelings of each subject and the direction of network public opinion at any time.

### 4.3. The collective reaction of the public under the WSR system

Network public opinion is a group reaction generated by the role of online media. Individuals express their personal opinions and feelings through online platforms, which trigger the attention of more individuals and induce group public opinion. After individuals have formed their awareness through the “Wuli-Shili-Renli” system, they express their views and opinions on the network platform, and the network users resonate with them, gradually converging into an opinion group with representative views. In addition, individual stressors in the epidemic area will pay more attention to online events, which undoubtedly intensifies Internet users' perception of events in time and space. The result is that individual speech is transformed into an ever-changing network public opinion.

In the areas where the epidemic occurred, certain events triggered public reactions that often originated as single-factor problems at the Wuli-Shili-Renli levels and eventually developed into systemic events at the “Wuli-Shili-Renli” level. For example, in a community where supplies are not distributed in time and rotten vegetables appear, the influence of the Wuli factor does not cause public opinion to ferment. However, the lack of effective control over the distribution of supplies and vegetables between the Shili and Renli systems leads to a situation that gets out of hand and eventually creates an internet hotspot. In other words, whether from the dimension of Wuli-Shili-Renli, the residents isolated by the epidemic could feel the emotional resonance and voice out on the Internet, forming a group response. “How are the staff assigned? How are they managed!” and “How much longer do we have to live like this.” These responses have profoundly affected the climate of public opinion in the region. Similarly, after the completion of the establishment of the square cabin hospital, with the Wuli factor and the Shili factor going smoothly, the unreasonable handling of the Renli factor once again triggered a hot debate on the Internet. Not only that, in a medical aid case where both the Wuli factor and Renli factor went smoothly, 120 ambulances could not enter the district in time because of the untimely handling of the Shili factor, which also triggered network public opinion.

In addition, from the coding results, among the leading positions generating network public opinion, the focus of Internet users' attention in Figure 5 is firstly reflected in the status of the epidemic, community action, and urban epidemic prevention, echoing the results obtained in Figure 4. In particular, in the public's opinion on positions related to epidemic prevention, positive and negative statements emerged, and conspiracy theorists emerged. Once the WSR system had problems with such events, the degree of online fermentation spread further, leading to online chaos, as shown in Figure 5. This shows that community action has a more significant impact on public life in epidemic prevention and control. Information notification in the Shili system is also a concern for netizens, which shows that information notification and communication, such as epidemic prevention information, safety information, help information, and security information, are essential. The acquisition and communication of information significantly impact personal life and are crucial to regional epidemic prevention and control. The public is more concerned about the status of epidemic prevention and living resources. It can be seen that any of the dimensions of WSR is crucial in the governance of network public opinion. Each element has a profound influence on the direction of public opinion development. It can be concluded that the analysis of network public opinion through the public opinion framework established by WSR has specific theoretical and practical values.

## 5. Conclusion

This study discusses the factors that influence the generation and spread of network public opinion on regional hotspot events from the three dimensions of Wuli-Shili-Renli. This study obtains three core categories, 15 main categories, and 86 categories through grounded analysis. Moreover, the WSR Elements Framework Of Network Public Opinion is established. The mechanism of WSR-EFONPO is analyzed from the following perspectives: the process of individual formation reaction, individual differentiation reaction, and the collective reaction of the public.

In WSR-EFONPO, first, through the analysis of the individual formation reaction process of the WSR system, it is found that an objective material basis is the fundamental reason for the occurrence of public opinion caused by hot events. The Wuli factor, as the cause of public opinion, is rooted in the fact that material resources are the necessary basis for individual existence. The abundance of material resources directly affects individual life. The epidemic will undoubtedly increase the individual's perception of the environment. The local crowd environment will cause an asymmetry in information acquisition, and hot events will become a centralized platform for information aggregation. Therefore, it is necessary to objectively help the public solve problems, significantly improve the coordination of material resources, stabilize the development of the epidemic situation, let the public obtain complete information, and realize the healthy development of the network ecology.

Second, in the analysis of the individual differentiated responses under the WSR system, it is found that the mechanism is an important driving force for the fermentation of network public opinion on hot events. The Shili factor promotes the development of network public opinion; communicators' cognition and use of management mechanisms and rules affect the development trend of public opinion. The guidance of public opinion should focus on the solution of individual needs, the design of organizational mechanisms and the division of responsibilities and cooperation, and other aspects. Based on the objective events, it is more important to strengthen the communication, coordination, and management guidance system of the Shili system. The management system can be improved in terms of top-level design and theoretical guidance, and the use of artificial intelligence and big data to realize the monitoring of the network. The Shili system is conducive to better control of the development trend of public opinion.

Finally, in the analysis of the collective reaction of the public under the WSR system, it is found that multi-subject collaboration is a long-term strategy for public opinion governance. The participation of multiple subjects affects the effectiveness of governance and the guidance of network public opinion. Through the Renli factor, it is found that individuals or organizations caused by epidemic isolation may play the role of demand-side and solution-side at any time. The solution of demand requires the coordination and interaction of government, media, and community, which affects the fermentation degree of events and the difficulty of public opinion governance in the process of coordination and interaction. Therefore, it is necessary to mobilize all kinds of forces to guide and monitor the trend and direction of public opinion. It is necessary to pay attention to the joint efforts of the government, the media, and society to activate the relevant subjects to participate in the settlement of the event and the governance of public opinion, and form a joint force in many aspects.

## 6. Contribution and limitations

### 6.1. Contribution

The theoretical contribution of this study is mainly reflected in the following aspects: (1) The combination of WSR methodology and grounded theory makes up for the defects of high dimensionality, poor logic, and weak correlation among dimensions in the grounded theory analysis of text content. The three core categories of Wuli-Shili-Renli and the main categories based on these three dimensions are designed to construct the public occurrence mechanism model of network public opinion in regional epidemic hot events. The model combines the cross advantages of systems science, management, humanities, and behavioral sciences. In the application process, grounded theory is more logical and scientific, and the WSR methodology has also been extended in practice. This combination of grounded theory and the WSR method can provide a reference for more research. (2) WSR method analyzes the macro-perspective and micro-factors of public opinion from three dimensions, which not only provides a systematic framework and logical context for the construction of the public opinion mechanism model but also provides a direction for the development and improvement of the model.

### 6.2. Limitations

The shortcomings of this study are reflected in the following aspects: First, the number of comment texts used in the manual analysis is not large enough, and only user comments from microblog data are used. In the future, multiple heterogeneous datasets can be collected for further research and verification. Second, the number of 'hot events' used is small, and the text gathers network public opinion cases such as hot events and related events in epidemic areas. Further research can collect more cases for research and enrich the research content. Third, this study adopts grounded theory, a qualitative research method. Some conclusions have empirical components. Subsequent research can use a combination of qualitative and quantitative methods, from qualitative analysis to quantitative testing, for comprehensive verification so that the conclusions are universal and scientific.

## Data availability statement

The original contributions presented in the study are included in the article/Supplementary material, further inquiries can be directed to the corresponding author.

## Author contributions

KY and JZ: conceptualization, methodology, formal analysis, and writing—original draft. LY: supervision, funding acquisition, and writing—review and editing. YLin: data curation. XH: software. YLi: investigation. All authors contributed to the article and approved the submitted version.
