# Supplementary material for: Analysis of network public opinion on COVID-19 epidemic based on the WSR theory
Source: Front Public Health. 2023 Jan 13;10:1104031. doi: 10.3389/fpubh.2022.1104031 (PMC9880161; doi:10.3389/fpubh.2022.1104031)
Supplement: Supplementary file 1 [file Table_1.DOCX]

| 1. 评论内容 |
| --- |
| 1. 上海最近确实做得不好，但是上海还是中国防疫做得最好的地区，不服的自己跳出来把入境上海的香港人接过去 |
| 1. 上海从来没自夸过吧？我觉得至少到目前2年多很不错。外防输入压力最大的城市，没有之一。 |
| 1. 上海就算沦陷了，也还是上海。 |
| 1. 不过真的觉得上海还是挺好的，没疫情的小区照常生活，群众的素质很高很配合，没有完美城市，但可以打高分。 |
| 1. 难免都有百漏一疏的情况，我们都是不完美，只要积极面对就可以！我爱上海 |
| 1. 确实上海这次令人失望，不理解为何这么严重了，还不停课，早就应该像两年前一样停课了，这次远比两年前严重的多 |
| 1. 前一阵吹上海吹的过头了 |
| 1. 上海人有意思，外地发生疫情骂得最凶，洋洋得意说自己多了不起，发生在自己头上，好吧，百密终有一疏，可以原谅 |
| 1. 还一个劲的无症状，无症状。。 |
| 1. 评论里没必要踩上海捧深圳，我是深圳人，但是我依然认为真正的国际化大都市，只有上海，深圳还真比不了。 |
| 1. 对于一线城市，如何在奥密克戎这种超强传播率的病毒下，以最小代价，提高与调整精准防控的措施，来保证城市的正常运行才是最重要的，病毒不断升级变异，肯定会不断出现问题，遇到问题能及时解决问题，探索出最合适的防控措施才是最大的收获，城市的体量，经济地位决定了防控手段的不同 |
| 1. 锦江集团S运作 |
| 1. 小学生才不开心呢我在隔离点我隔壁的小学生每天被核酸都哭的像杀猪 |
| 1. 质疑很正常，我身处其中，周边很多小区封控，也有很多学校开始网课，同时也有很多人还在正常工作生活。全国很多地方可能也都是这样，防疫模式有最佳吗？好或不好自然有生活在这里的人评判。最字就不必去用了吧。不论如何，我深爱自己的家乡，也愿意相信她与她并肩作战。 |
| 1. 上海已经开始征用有长租合同的公寓了，中午通知凌晨收拾完全被赶出来坐标徐汇区天钥桥路909号逗号公寓 |
| 1. 那么多人口，承包了大部分境外旅客，一有疫情层层筛查，流调工作做得有效精准，没有一刀切，没有造成恐慌和混乱，好不好从来不自夸，大家都看得到 |
| 1. 这种时候又是永无止境的地域攻击，身在上海我从来没自夸过，用不着一个人代表全部。我现在被封在里面，快递两天了都没拿到，深刻感受到疫情下暴露的短板 |
| 1. 精准防控没有错、而且是行之有效的，但问题出在某些人不识大局的愚蠢与贪婪。 |
| 1. 学生还是太天真¡评论配图 |
| 1. 我只知道这两年云南真的太难了 |
| 1. 说实话，上海的高帽子可是新华社这些央媒硬扣的，大家可以看下每条下面都有上海市民喊不要再宣传了，上海只想低调抗疫，每个城市社会环境都不一样，上海也从来没有叫大家学我们。现在扛不住外溢了反而拼命往上海身上泼脏水。 |
| 1. 上海好多条线，华亭宾馆的那条肯定是人祸，事后疫情平复相关人员一定会被处理吧，关键现在还有好几条线的源头没找到，就代表潜在风险还是比较大的，上海加油，一切都会好的 |
| 1. 香港到处都是新冠接手的是上海；全国其他呀，不服的把请把香港回来的都接过去 |
| 1. 西安这次疫情就是上海飞西安航班带过来了四个，然后一下次传开了 |
| 1. 今天网上辟谣了，上海疫情和香港、和华亭之间的关联 |
| 1. 我大学同学今天凌晨发了个圈，封小区封楼，做核酸。。。。真不容易啊。成都都才清零，希望其他地区早日回归正常生活 |
| 1. 你的消息被证实了，华亭宾馆 |
| 1. 我们市两个确诊的无症状，都是上海回来的 |
| 1. 还真有尼玛这样的奴才还夸深圳的//@曹2小B:深圳的香港通关入境人数是上海的多少倍，还有偷渡过来的，风险不比上海高吗？为啥深圳疫情也很严重，但很少有人喷深圳？因为上海那些所谓的精准防控，深圳一项不落的都在做，只不过没有宣传，政府没有，老百姓也没有。出现疫情也没有死撑着精准防 |
| 1. @bobi26你一个上海硬盘三和，住里弄天天倒手提马桶的货，在这里说尼玛呢 |
| 1. 讲个笑话，上海从没自夸过防控做得好呢。除了迪士尼烟花下的核酸、全国最小封控区奶茶店、精准防控巴拉巴拉…… |
| 1. 这波装逼装死了 |
| 1. 上海这次确实出现了纰漏，确实该检讨，但上海一定会度过这次难关的。上海这次严重了，这个时候可以说上海这次做了如何不好，但不用跳出来说上海是不是优等生的问题。只有酸优等生的人，才会等优等生出错了马上跳出来说。 |
| 1. 评论底下地区上海旁友和外地旁友扭打在了一起，个么作为在外地的上海人，我只希望阿拉上海学雷锋做好事的事体可以少做做，摆亏劲扎台型，没意思的。背负太多，又不给你发奖金 |
| 1. 上海为什么要自己跳出来把香港人接过来？好像有什么大病……华亭宾馆…这地方真是我童年阴影 |
| 1. 大家对上海敌意怎么这么大？？？？？？？？ |
| 1. 搞了二年多，疫苗大规模接种，现在弄了一堆无症状，防控难度更加大。再不调整防疫政策，可以考虑港澳地区财政转移支付了。 |
| 1. 下老师还不忘记调侃这帮神兽！ |
| 1. 我是在上海的打工人哈，2个娃娃分别是幼儿园和小学，今天全部在学校做核酸，普筛，办公室园区今天也在全部核酸检测，其他生活方面目前不受影响，希望娃娃们可以继续上学 |
| 1. 我还真没见过上海的学生夸上海的 |
| 1. 上海永远是上海人以及热卖上海的人们的太阳！ |
| 1. 哎，如果有证据印证，确实是zf某环节的过失。但是功大于过这是不争的事实，你可以加点点人文关心，你最后那段话，讽刺的很恶心！真希望你所在的地区可以做的更好！ |
| 1. 「没有哪个偶像是光，只是其他人把他当成偶像而已 |
| 1. 问就是精准防控 |
| 1. 评论里有些人的逻辑和言语让我更希望网暴尽快专项立法吧 |
| 1. 上海加油！ |
| 1. 都是网红惹的祸！ |
| 1. 说一句上海加油真是难为你们啦 |
| 1. 我觉得之前精准可控是没有错的但是精准可控只对人与人传播有效在上海大数据完备的情况能追到最小影响的控制但是面对这次环境污染尤其是疏忽导致的处置滞后才会大面积开花 |
| 1. 确实上海肩上的担子太重了，入境的压力，流动人口密度，时至今日各种质疑，一有问题就各种仇恨各种讥讽，做着最吃力不讨好的事，却受着最重的指责。 |
| 1. 偶像包袱 |
| 1. 这种上海人真的笑死。前几天我还说，把香港放上海旁边试试。结果才来了几个香港人就受不鸟啦😅😅😅//@数学汤家龙_:不好意思，境外输入最大的是深圳。其次，上海没自夸过这句话我是不敢恭维，微博上海人自夸可太行了，自夸精准防控，嘲笑别的城市全民核酸，嘲笑封城。还控评。这些没有之一。 |
| 1. 不是漏掉，是避而不说吧 |
| 1. 这就好比一直是优等生，次次得90分，一次得了80分，全部都在看笑话 |
| 1. 上海挺好的呀，在其他地方的人看来有多严重多严重。但是没有影响大部分人的工作生活，该怎样还是怎样很便利。 |
| 1. 香港这一点存疑？因为身边有朋友咨询过海关的工作人员，海关人员明确表示是假 |
| 1. 转发微博 |
| 1. 我终于在上海要核酸检测了 |
| 1. 上海人还来西安了 |
| 1. 现在小孩子，心理强大。🙏不会的占多数。 |
| 1. 上海全民检测估计快了。松江已经开始全民检测了。 |
| 1. 装犊子弄不过上海 |
| 1. 上海这种国际级大城市很难做的，现在这样说实话很不错了，华亭宾馆个例也太特殊了 |
| 1. 精准防控做的再好，奈何架不住多点爆发！疫情防控也许存在疏忽，但是上海已经极力挽回补救，作为承担全国外防输入1/3压力城市，上海已经做的很好了。 |
| 1. 很多人终究活成了自己最最讨厌的样子… |
| 1. 之前是在源头上直接卡死才显得好像很行…其实我早就说过只看表面，我魔都总有出事的一天，之前都用好一点的酒店，所以很多时候根本没有调研，没有数据的操作就是扔硬币 |
| 1. 其他地方有疫情了，只要48小时核酸证明就可以来上海，上海说什么了?苏州有疫情不是照样来上海。不过华亭宾馆确实没做好。 |
| 1. 可怜了小朋友，可怜了医务人员……… |
| 1. 上海一点也不好，别来上海了，谢谢🙏 |
| 1. 疫情面前有多少妖怪真是能看的清清楚楚有些人真的巴不得上海这种量级的城市垮掉，好满足他们的口欲还有指点江山的心情 |
| 1. 沪吹，没必要有些东西吧，说透就没劲了//@博文雄起:那当初是谁把客机推给南京的？ |
| 1. 上海只能上海人自己说！不能巴子来说三道四的 |
| 1. 谁要偶像包袱了，该怎么应对就怎么应对，都是你们乱加的头衔好吧，管理的好不好你生活在这里才知道，至少我觉得政府能力极佳才能让我正常生活 |
| 1. 上海难道是为了自己沦陷的吗？没有功劳也有苦劳吧，为了利索能力的分担点香港入境，把自己搭进去了，然后深圳喷子～还要喷上海打肿脸充胖子，我想说骂得好～上海政府你们长记性了吗 |
| 1. 真相了 |
|  |
| 1. 他们不敢说是他们得错一帮缩头乌龟为了自己的的位置牢固所以一直瞒着 |
| 1. 天哪上海加油我最近幸亏在老家没回上海。就担心春运期间人员流动万一病毒就等三月份回结果。希望疫情早点结束 |
| 1. 成都写的挺好，真的到这种程度了？//@萌宝木易沐:确实上海这次令人失望，不理解为何这么严重了，还不停课，早就应该像两年前一样停课了，这次远比两年前严重的多 |
| 1. 上海还是上海不管怎么黑素质在那里 |
| 1. 大家不要这样冷嘲热讽说上海了，毕竟上海境外输入压力很大，再加上接收香港入境的人，国家防疫是保护人民群众的生命安全，我们能做好自己每个人该做好的事，不要轻易被煽动听信谣言，相信一切都会很快好起来。 |
| 1. 疫情到哪儿都不好过，干嘛还要唧唧歪歪分个上中下，虽然这波上海天天有确认，有点瑟瑟发抖，但是还是要相信政府的，自己也做好防疫措施. |
| 1. 面对问题共同克服，不支持打地域嘴炮，没有意义，大家加油。 |
| 1. 首先，从疫情爆发开始，深圳的核酸就是免费的（2021.10.1去了一趟上海，把这事说给上海亲戚，他们都不相信，因为上海此前一直是收费的）！其次：深圳只要有确诊病历，深圳各个地区基本上隔天就做核酸，持有48小时内的核酸码看到上海那边朋友圈发核酸检测啥的，这些在深圳已经成为了常态 |
| 1. 学渣等着学霸出丑，但学霸永远是学霸！ |
| 1. 一粒🐭💩啊 |
| 1. 已经官宣就是这个宾馆隔离期间外漏 |
| 1. 评论都真的好宽容这要是换其他城市防疫已经三年居然搞出这么大的纰漏导致本市那么多人感染外溢到好几个省市早被喷得体无完肤了上海之前的防疫工作做得是好无可指摘但这事儿也是实打实问题很大两码事儿 |
| 1. 喷子真多. |
| 1. 华亭宾馆事件发现的晚，后续跟进措施又不果断，如果及时封控周边，按区域做封闭管理，也不会弄的遍地开花。 |
| 1. 上海的精准防控现在看起来就是自吹自擂的笑话 |
| 1. 现在的上海每天都有那么多小区封控真的人心惶惶 |
| 1. 哎好烦人哎看评论 |
| 1. 上海人的优越感只有上海共情，其他城市就是在看猴在跳，北京都没上海人优越感那么强，这是上海已经发展成美国了吗，这么拽的 |
| 1. 浙江嘉兴平湖发现一例时：封锁整个城，第二天凌晨就开始全员核酸做一个礼拜。且不是城市大小如何，错过最佳时间都是无法预算和弥补的结果！并上海本地人优越感从骨子里就是这种看不起任何人，封控期送菜，租户是菜品单一，本地人是菜品多的。如今确诊这么多例竟才是第二次全员核酸！无法理解细品 |
| 1. 我也相信上海能战胜本次疫情的上海加油，但是我反映一个信息，上海宝山区通河六村的市民们没有接收到任何发放的蔬菜，希望大家关注哦。 |
| 1. 对的发布会就没提香港入境为啥会有那么多只字不提 |
| 1. 说的太对了，不是上海自己而是外界，是外界把上海抬得太高了，捧杀的太厉害了，这次疫情真的暴露了很多问题，上海不是神话，是时候让大家看看，你们以为的多么高大上的地方，其实也有散沙，也有管理不力的领导。大家都一样。 |
| 1. 上海的日笼包不要到处跑了下乡去给你们上坟么？ |
| 1. 还在吹，服了 |
| 1. 上海封了吧，硬是要闹成全国疫情才罢休？ |
| 1. 香港人是谁要接过去的？深圳都不接了 |
| 1. 上海寸土寸金 |
| 1. 上海那个护士挺可惜的。自己上班的地方不给医治。多么痛苦 |
| 1. 还吹上海不 |
| 1. 普通老百姓生活很不便利，已经封了20天了还在封 |
| 1. 为什么目前酒吧还在开？ |
| 1. 新冠是谁的恩怨？是老美吗 |
| 1. 真无语，突然爆发，很吓人啊 |
| 1. 其实我觉得只有上海才觉得自己有偶像包袱，大部分外地或者受上海疫情影响的地方在经历最初的愤怒之后只会用看傻子一样的眼神看那些所谓精准防控的政策 |
| 1. 现在的难度是无症状，这样传播就很难控制 |
| 1. 虽然网上争议不少，甚至觉得上海模式就是错的，其实不至于，本来就不是神话，一方面靠实力，一方面靠运气，如今也没啥破灭，实力还在。总之，正视现实，理性抗疫，沪吹和沪捶都要不得。病毒在于不在，上海依然是上海，值得在这里的每一个人热爱。 |
| 1. 你写的东西有点作家的才思 |
| 1. 我家马路对面的小区也有个确诊的，小区被封了估计我们小区。。。。。 |
| 1. #上海六院# |
| 1. 深圳才是真的惨，这帮港灿太害人了 |
| 1. 上海城市不错，上海人不行。这是我住在上海的感受 |
| 1. 上海抗疫如果10分满分的话，我最少都可以达到8分， |
| 1. 我身边就没听说过谁自夸上海怎么怎么样，反倒是这次，全在骂市政府怎么怎么不好，比如漕溪北路1200号什么的 |
| 1. 上海人民都觉得工作人民太辛苦了毕竟2000万大部分还是正常生活的 |
| 1. 上海最近防疫出现很大的漏洞？请问即个干部被问责？处理？近来吉林处理很多防疫不得力的干部？上海呢？ |
| 1. 也只敢怪一个宾馆？嗯，没有人负责，没有人为事件买单 |
| 1. 截一图来看看微博上海人如何自夸。//@你永好运:上海从来没自夸过吧？我觉得至少到目前2年多很不错。外防输入压力最大的城市，没有之一。 |
| 1. 上海就是拇指，那些怪三怪四的yp终究还是白眼狼！ |
| 1. 难得看到居然不怪港府无能，被祸害的深沪居然撕起来，呵呵 |
| 1. 很正常啊！有条不紊的 |
| 1. 外溢多市了还全国最好 |
| 1. 除非想躺平，如果想动态清零，全民核酸有必要的，而且要快，之前说上海大部分人没做过一次核酸，我就想早晚出事。 |
| 1. 欠下的终究要还 |
| 1. 我姨去迪士尼乐园玩，只检查中国人戴不戴口罩，两个外国人不戴口罩，都不管 |
| 1. 上海疫情严重了没有啊？这怎么还在扎堆 |
| 1. 被孩子的天真感动了，加油小可爱，照顾好自己 |
| 1. 网红医生你在哪里 |
| 1. 哪个城市把境外输入压力接过去再哔哔呢？ |
| 1. 北京学着点吧 |
| 1. 天龙人的城市，谁敢说 |
| 1. 明天开始，他们的爹妈，会流下断线的珠子 |
| 1. 小朋友们还挺开心的他们以为放假了吧？ |
| 1. 泪水如同断线的珠子。什么意思！你这是幸灾乐祸嘛！ |
| 1. 说不得的 |
|  |
| 1. 我是上海人，我也觉得上海是不是飘了?!搞到全市停课!全员核酸! |
| 1. 港➗能不能死在港啊 |
| 1. 我只想说能不能停了大学生上网课吧刚解封两天又封校了老师密接我真的受不了了求求上网课吧我想活着 |
| 1. 这条微博能开地址吗，好奇 |
| 1. 上海加油，扛过去 |
| 1. 这个是我小时候读书的地方 |
| 1. 敢把自己的问题发出来也是挺震撼的~！ |
| 1. yp们快去吹深圳使劲吹 |
| 1. 上海有偶像包袱？谁把上海当偶像了，就上海人自己捧自己吧 |
| 1. 还有六院的事呢 |
| 1. 上海这次疫情，如网传属实的话，那就是人祸～ |
| 1. 香港人能不能待在自己那边啊 |
| 1. 泪水如同断线的珠子，文案有那味了煽动对立言论发一发多少钱一篇啊 |
| 1. 每次只要上海有点事，下面一堆人高潮，好像上海无可救药。 |
| 1. 管控人口对比全城总人口反映了管控精准度；散发数对比管控区内发病数反映了管控有效性；无症状数对比确诊数反映了防疫速度 |
| 1. 香港那么好干嘛要回来呢 |
| 1. 武汉当年可以封城，为什么香港就要有飞机出来，什么紧急的事要来大陆不可，上海争着抢着出头的一个决定，几乎全城人都要核酸，不需要和老百姓有个说法吗 |
| 1. 作业并不会少，还是堆在那里等她们 |
| 1. 做的不好就是做的不好，要有人担责！ |
| 1. 什么时候偶像包袱了怎么你骨子里的自卑感出来了？ |
| 1. 还查健康码么？ |
| 1. 应该也漏掉华亭宾馆收隔离人员一天700块吧，住在里面被感染的隔离人员倒霉跟谁说 |
|  |
| 1. 奥密克戎专治吹牛逼，科学防疫！ |
| 1. 竹园太惨了，周边的学校都很可怜。 |
| 1. 上海赢麻了，外溢了也是好事 |
| 1. 你在咒谁呢？难怪名字那么刮三！负能量 |
| 1. 开心？你说的是人话吗？ |
| 1. 带节奏的都来了 |
| 1. 别的不说当初深圳沦陷的时候一堆不知道哪来的人哪上海做比较说上海多好多好上海能做好深圳就做不好巴拉巴拉我只能说希望这群人长脑子别毒奶了 |
| 1. 阴阳怪气说话的就不是啥好人 |
| 1. 上海华亭 |
| 1. 服 |
| 1. 最近上海是市区疫情不好，人员密集，9号杨浦区新华医院，鞍山中学，10号徐汇区某小区，菜场买菜买一半被封，出不来了14天 |
| 1. 这是竹园小学们 |
| 1. 可怜了孩子们。上海政府必须给老百姓一个合理的说法 |
| 1. 我早就说过，都在中国，水平都差不多，区别在于有的地方级别高，网络话语权多，买办沪整天秀优越感，这下秀底裤了 |
| 1. 为什么还要让香港人入境？ |
| 1. 打着歇业的旗号，就是为了接香港旅客 |
| 1. 我觉得上海最大的优势是民众的配合。现在多点散发，我出去看路上基本上都带着口罩。地铁里人少多了，大家都配合待在家里。 |
| 1. 他们两天就要想妈妈了！ |
| 1. 你这个评论会不会想管媒还要翻牌子？ |
| 1. 我不理解现在的这个社会都怎么了？饭圈互撕也就算了，这年头，连城市城市居民之间都要开始互撕，那些深圳的，你们自己没家吗？四海为家，好意思吗？要脸吗？别在那一个劲的吹自己，不然会打脸的。评论区的人一个个的都是柠檬精吗？ |
| 1. 上海做的不好就该骂，前段时间做的好的时候都在夸！ |
| 1. 昨晚我们小区沦陷，上海这次有点多🙏 |
| 1. 攀比防疫谁厉害，还不如讨论下清零政策和全民免疫 |
| 1. 成都取星了，也为我们成都骄傲。 |
| 1. 上海这一波确实大意了 |
| 1. 看评论，上海那来那么多优越感。 |
| 1. 我们家旁边一个园区估计有密接，关闭隔离。上千人睡办公室。我在旁边小区，完全没有影响，自由出入。不过我也不出门，屯好东西 |
| 1. 都忽略了一个时间点，两会期间啊！我们住隔离大楼的，周日晚上核酸，周一照常上班。完了我才知道原来华亭被用作隔离酒店了 |
| 1. 华亭宾馆突发意外什么情况？ |
| 1. 国家能清查上海每一个角落就好了，万一有病毒源头，也可迅速拔掉，但愿是我想多了 |
| 1. 评论看了，看来很多人不明白隔离酒店通风和中央空调的关键所在，华亨的问题绝对是决策问题 |
| 1. 大上海是国际化大都市，防疫难度高，首次的全民核酸检测做到了有条不紊上海加油 |
| 1. 阳性感染的密切接触怎么能就48小时管控，起码7天～14天。上海目前密接的风险地区基本48小时封闭，对比很多检测N次才确诊的先例，前车之鉴，这样合理吗？对于上海这样的超大型中心城市，而且是学校是孩子！​​​ |
| 1. 上海核酸检测费40，（21年下半年国家已明确调整收费，远低于上海）2400多万常住人口检测数只有400万（有说几十万），即便苏州疫情非常严重期，22年春节异地人口大规模流动返乡期，入沪仍无需核酸报告。“沪吹”时请以事实论据，人民利益为本，生命至上！ |
|  |
| 1. 上海做事很周全 |
| 1. 上海从来没有偶像包袱，只有在被排挤被嘲讽。被顶在杠头上后的努力坚强不息 |
|  |
| 1. 把这些嘲笑西安的上海人晒一下 |
| 1. 可能被张网红害了 |
| 1. 上海有过偶像包袱？？？？ |
| 1. 哎哟有什么好说的，我作为上海人也没说别的地方做的不好，各个地方按照地方情况来进行判断，同一个题目10个人可能会有10个做题方法。媒体总归会写些让人觉得好的地方，上海人的自我满足也是对这个城市的信赖。喷子永远是喷子，不排除他还喷别的地方。共同合作国家才会好 |
| 1. 正常入境的旅客和宾馆准备装修又被紧急征用有什么好说的？征用隔离宾馆没考虑通风才是问题，精确防控连这个都不在必选项吗？ |
| 1. 张砖家，啪啪打脸 |
|  |
| 1. 在中国自卖自夸最终会有问题 |
| 1. #上海疫情发布会#上海做核算的医院人山人海，人员大量聚集，没病也要得病了，上海政府你们管吗？@上海市政府，@上海疾控中心。@国务院 |
| 1. 没漏吧？上海发布上有说啊 |
| 1. 11号线应该停了吧，怎么自己疫情就不停，苏州疫情第一时间停 |
| 1. 上海所谓的精准防控，其实是一种新模式探索，前期高效，现在遭遇严峻挑战。此时此刻，我们需要冷静、实事求是，为上海加油，期待它探索的新模式能过关，为大家闯出一条更好的路来。 |
| 1. 基本上都是香港入境人员传播的 |
| 1. 为了防疫都不容易，我在上海，小区里发现了一位无症状感染者，然后就把小区30栋楼全封了，两千多户六七千口人呢 |
| 1. 夸也是博主动动键盘，踩也是博主动动键盘，两头话都让你们说了，你行你来！ |
| 1. //@幸运猪MaggieLMillion生活:锦江集团S运作 |
| 1. 上海是我最喜欢的城市，没有之一！上海将改变整个防疫战略 |
| 1. 围观优越感爆棚的上海市民。我就想问问，微信上都传开了的聊天记录是不是真的？如果是真的，这样你们都觉得做得不错，那你们是真的赢了。 |
| 1. 之前所谓上海多精准，都被自媒体带了节奏，其实有脑子的想想都知道哪有那么牛逼 |
|  |
| 1. 竟然不是穿防护服！！ |
| 1. 深圳的香港通关入境人数是上海的多少倍，还有偷渡过来的，风险不比上海高吗？为啥深圳疫情也很严重，但很少有人喷深圳？因为上海那些所谓的精准防控，深圳一项不落的都在做，只不过没有宣传，政府没有，老百姓也没有。出现疫情也没有死撑着精准防控的面子，老实的落实全民核酸。 |
| 1. 大哥，云南瑞丽可是还要接收来自首的上百万的缅北各省诈骗分子的…… |
| 1. 上海真的没有脸说香港入境，深圳还面临香港偷渡的，舔着脸说了吗 |
| 1. 这光景了还有人死要面子活受罪 |
| 1. 可以都别接啊上海人民同意接他们了吗 |
| 1. 我现在突然感觉武汉已经很屌了 |
| 1. 深圳是全国防疫教科书📗 |
| 1. 前一段时间大家都在讨伐西安疫情时上海吹嘘的精准防控，人性化防控在哪里？新冠病毒专治各种不服。尤其是爱吹牛自认为做的很好的。 |
| 1. 深圳面临每天是上海几倍的香港入境（这是正规入境的），还有不知道多少人潜匿城中村的td，还有违规的货柜司机，比上海难太多了 |
| 1. 上海人前几天还在笑深圳呢。深圳还是在香港旁边，上海来几个香港人就不行啦😅😅😅。啊这😅😅😅 |
| 1. 一天天的优越感强的不知道该说什么… |
| 1. 我就说一句，微博是个垃圾桶，正经人上来关心下疫情真实性，至于上海人是否虚荣跟我广州人何干？哪个省的人不吹牛逼？二三十爱装逼的年纪，四五十好为人师的年纪。再者微博上海人跟普通上海人也是有区别的，我相信普通上海人是朴素的。 |
| 1. 偷渡不比光明正大过去的难防？ |
| 1. 好个p我们西安被上海害了两次了我在宿舍之前隔离一个多月回不了家 现在又去不了学校管的好啊 |
| 1. 上海人经常在自夸防疫最好，我校友群里新上海人都在自夸，不然为什么网上反弹这么大。 |
| 1. 拉倒吧，自夸的还少？什么迪士尼的烟花下的核酸。什么只封奶茶店，最小的封控区 |
| 1. 官方数据广东接受全国8成境外入境人员。深圳用全民天天检测替代封城实验效果还是可以，保证城市基本运行同时将感染人数压缩到最少，隔壁香港是每天几万新增！ |
| 1. 外防和广东还是没得比吧 |
| 1. 你想笑死谁上海没自夸过 |
| 1. 上海就是自夸太多才会被嘲的呀 |
| 1. 我觉得微博评论就是个照妖镜，会照出人的自卑，嫉妒，以及内心的阴暗面。上海不是中国吗？他防疫做的好难道不应该赞赏？就算防疫做的不够好，难道不应该多给人家鼓励？难道就讽刺？嘲笑？难道这些喷子都希望上海崩塌？麻痹，都是什么人？ |
| 1. 讲个笑话，上海从没自夸过防控做得好除了迪士尼烟花下的核酸、全国最小封控区、精准防控…… |
| 1. 救命，这楼下面什么玩意儿疫情归疫情天天有人拉踩城市脑回路也是一绝要不然颁个奖吧最佳抗疫城市 |
| 1. 笑死我了，都这样了上海人还在自夸 |
| 1. 官方用媒体炒作，个别市民跟屁吹捧，否则这种舆论反噬为啥深圳北京没有 |
| 1. 还没自夸呢 每次其他城市出事时候你们就跳出来夸自己做得好 现在不自夸了？好话赖话都让你们说了 |
| 1. 2年多一直没清零，就是上海。 |
| 1. 上海就算沦陷了，也是沦陷的上海 |
| 1. 美国就算死那么多人，还是美国呢 |
| 1. 上海人的优越感 也只有上海人才能共情到在大多数人眼里 跟在看猴耍似的 |
| 1. 民国时因为有外租界甩开了中国大部分地区的生活水平，因为这骄傲，和香港人看待大陆的傲慢有什么区部？或者你认为香港人面对上海人也该像你这么骄傲？乌合之众，没迷人的特点，就认定团体的偏见。 |
| 1. 上海就算沦陷了，也是你们买不起房子的上海 |
| 1. 哇，第一次见城里人 |
| 1. 哇偶 上等人是吧 |
| 1. 不是上海人也不是北京人的人，为上海和北京吵起来了 |
| 1. 北京人看不起上海人 上海人看不起所有人 |
| 1. 对哒，哪儿沦陷了都还得叫原来的名字 |
| 1. 大清都亡了 |
| 1. 楼里这么多嫌弃上海的 求你们这辈子别来旅游工作吧 上海不配 |
| 1. 皇城根底下的臭青蛙🐸哈哈哈哈哈 |
| 1. 现在你还是这样认为吗？ |
| 1. 你现在还这么认为吗？ |
| 1. 现在回看，这些天上海发生的怪事，还能称为素质高吗？打脸了，啪啪响啊！ |
| 1. 回复@毛手毛脚就是我:素质没有姿态高 |
| 1. 回复@Nancy郁_爱LH和刘大神:想问问上海6000例无症状感染者有高风险地区吗？ |
| 1. 到处跑那几个素质也很高 |
| 1. 回复@买买买全部都买:我们市最初的三个感染者都是上海带过来的 |
| 1. 回复@Mestallaer:上海也有瞒报的，奉贤平台确诊了怎么查不到 |
| 1. 回复@七二三二七:大家看看，给北方洗地的都出来了 |
| 1. 回复@浦东大西瓜:我在北方怎么不知道一例封城，石家庄这都多少了我每天怎么出去的 |
| 1. 回复@大老虎的微波:并不一样，郑州都很受影响，小城市影响的更不要提了 |
| 1. 你对病毒的理解是否太理想化 |
| 1. 回复@WwAaNnGgAaMmYy:现在上海疫情有什么感受 |
| 1. 没毛病 |
| 1. 你可以爱上海，我们都爱自己的城市，只是别的城市发生疫情的时候，不要跑出来显摆优越感！因为事实证明，谁遇上都一样！ |
| 1. 也可以的，平时是钉耙，偶尔用一下梳子。 |
| 1. 回复@Cherri0:哪有那么多利益关系，每天进来这么多，又承担了深圳一部分香港压力，酒店不够了。早在过年前上海的隔离酒店已经不够了。 |
| 1. 本来歇业的宾馆为什么突然能作为隔离用？其中没有利益关系，我是不信的 |
| 1. 可惜上海不爱你 |
| 1. 对待外地也能这么宽容就好了 |
| 1. 写错了。应该是百密一疏。百漏一疏不是成语，百疏一漏也不是成语。疏和漏是近义词，两者可组成词语疏漏，因此用在百漏一疏和百疏一漏中不正确，互相矛盾，没有意义。因此百漏一疏和百疏一漏也没有本质上的区别，两者都是错误的说法。有一个成语叫百密一疏。 |
| 1. 百漏一疏？百密一疏？百疏一漏？ |
| 1. 你这是黑上海吗 |
| 1. 不见棺材不落泪，处置太不果断了，说到底就是没担当 |
| 1. 因为两会啊 |
| 1. 回复@布拉纳斯:都停了 |
| 1. 泻药 已经停了 可惜上海带学牲不是人 |
| 1. 麻烦多关注一下新闻再出来喷 |
| 1. 已经停课了，好吗？ |
| 1. 停课了孩子你帮忙带？ |
| 1. 不是已经通知中小学停课，上网课了吗 |
| 1. 回复@-LoloEva_:想看你感染一下看看病毒会不会被你酸死 |
| 1. 主要单位不停，所以小孩没人管，毕竟新上海人多，没有老人看的 |
| 1. 回复@ME涨涨涨:听天由命吧 |
| 1. 已经停课了 |
| 1. 3月11日9 |
| 1. 为什么政策转向发生在10号晚上？查查10号发生了什么，什么闭幕了？然后就能想明白了 |
| 1. 吹啥了，之前上海防疫的成效是得到国家层面的认可的，现在上海疫情开始严重点了某些人就开始兴风作浪，兴灾乐祸，搞的好像只有上海会外溢出去，外省市的病例就不会流进来一样 |
| 1. 你看看面对奥秘可容 别的城市怎么应对的 都是直接包不住了然后一刀切封闭。 上海还有余力精准防控 我楼栋被封 但外卖买菜都正常 骑手没有全被封在小区里 生活还很正常 狗还能在楼道门前拉粑粑。比起那种上门打死狗的 买个馒头被打的 就是能吹。 |
| 1. 不仅吹，还驼鸟，上海发布上的留言一堆彩虹屁，所有质疑的声音都屏蔽了 |
| 1. 其实仔细看也不是上海人自己在吹 |
| 1. 无论是3000W还是1500W的房产，只要您坚持楼道里💩，不以为耻反以为荣，您的房产一定还会升值。 |
|  |
| 1. 回复@疯糰子:好吧，我确实误解你的意思了，从第一条回复，我好像确实没有仔细看完 我刚才重新看了一遍，抱歉！ |
| 1. 回复@爱上唐古拉:你非常的好玩，我代表不了那些确实存在的口没遮拦的上海人，废话，我本身就嫌弃他们，我说了半天让你去怼他们，也说了如果你觉得你怼他们孤身一人力量不足可以叫上我，好嘛？我看不惯？还有我一直在遇到不平的时候就立马就事开口，我遇不到的你让我做天眼吗？我突然觉得…你可能从来没看完过我的文字… |
| 1. 回复@疯糰子:虽然你是上海人，但是你代表不了那些确实存在的口无遮拦素质低下自以为是的上海人，我针对那些人说这些话，因为是他们先挑起的事端，我代表看不惯这些偏激狭隘者的西安人说这句话，你就看不惯了，那么这些无知者到处中伤其他城市的时候，你有没有站出来说一句呢？！好了，不跟你掰扯了！ |
| 1. 回复@爱上唐古拉:我作为魔都人…请务必看清楚这个，我不是代表，我只是其中一个，我知道我身边没有嘲讽其他地方疫情的人，也深刻体会微博上无数伪装者，仅此而已，还有并不是我不是偏激者我就可以路过，我不为自己家乡正名我不支持好人谁来？就像你们要反击那些口无遮拦者一样，沉默只会让无数普通人被误解被“代表” |
| 1. 回复@疯糰子:你代表不了任何人，你仅能代表你自己！我不随便认定他们的身份，我也不呼吁什么，我只说我看到的那些自以为了不起的“魔都人”，如果你不是这种偏激人物，你可以路过！ |
| 1. 回复@爱上唐古拉:没事，不管几个，我说了，我作为魔都人，有义务清理门户，那怕人嘴皮再溜我态度在那，另外有一说一，微博这么多年什么环境，挑事的人太多，你如果随便就认为他们的身份是真的，只能说你微博逛的还太少，曾经我也是呼吁理性追求真相的，直到我发现微博上很多人根本不在乎真相，我就开始逛的少了 |
| 1. 回复@数学汤家龙_:真正低调的是广州吧 |
| 1. 跟上海人有什么关系？你确定那些洋洋得意的不是某旦新闻系找来的水军？为了凹造型好让年轻劳动力都来上海贡献GDP |
| 1. 回复@阿荣和他的老公:你没说我，你@我搞毛线啊，你在我这说个毛线啊！真奇怪！ |
| 1. 回复@绿叶飘起来:是你没看到过…我看过年前有人说上海和其他地方是人口素质差距导致的疫情防控差距… |
| 1. 回复@爱上唐古拉:我说你了吗，非要在这对号入座 |
| 1. 回复@阿荣和他的老公:上海人没干过的事，我们闲得无聊非要安在你头上，可以了吧！事实摆在那，网络暴力到处都是，非要在这刚，无聊！ |
| 1. 回复@花残蝶逝:真不信上海有那么多无症状，死鸭子嘴硬 |
| 1. 回复@半亩花田3168895:无症状分很多情况，后面转症状和不转症状，不转症状就代表着疫苗的作用啊，既然疫苗有作用了，为什么还要搞的人心惶惶？ |
| 1. 回复@江南菲尔:上海现在无症状都没人管，被查到阳性也不拉出去隔离，还和其他人呆一个地方，真的无语了。 |
| 1. 无症状很正常啊……无症状转确证也有很多啊，标准和区分就是这样的啊 |
| 1. 回复@VIB_Woo:就是，谢谢！ |
| 1. 回复 @wanglifang:一群拿钱带节奏的 看到举报就行 中国大数据都记录的这些人 |
| 1. 因为是上海，说实话大家也不用辩驳，这些人内心就是恨上海，怎么样都不对，让他们自嗨就行了 |
| 1. 无症状多是因为局地全民核酸检测发现早还没来得及有症状，后期有些出现症状的会变成确诊，有些会直接转阴痊愈，都具有传染性，我们现在连密接和次密接都要隔离呢！ |
| 1. 无症状多是因为华亭隔离酒店内感染，本来没病的一进去隔离就有了，刚刚染上病毒就测出来了，无症状自然就多了 |
| 1. 回复@栾廷石:就是啊！收钱的不懂的。 |
| 1. 回复@江南菲尔:这些是人是鬼不知道，两年多了，这个最基本的国家都有规定的也不懂，机器人吧。哈哈 |
| 1. 看看青岛深圳等，无症状比上海多的多，搞不懂微博骂上海的比骂鬼子霉国寒国台独的都多，上海被开除中国了？那么仇恨上海到底是谁？我不相信中国人会做得出来的。 |
| 1. 无症状就是国家制定的标准，本来上海疫苗接种率就全国最高，加强针也是，所以基本都是轻症和无症状 |
| 1. 无症状不传染吗？不明白为什么天天区分确诊的和无症状的。 |
| 1. 没文化真可怕，无症状是国务院提出的 |
| 1. 。我回过头看评论来了。。。觉得打脸吧 |
| 1. 没必要踩上海捧深圳，但是你同样也没必要踩深圳捧上海。 |
| 1. 北上广深yyds |
| 1. 为你点赞！同是中国人，抗疫在先，不是靠辱骂能战胜疫情的，估计收钱的，拜登发钱了。哈哈 |
| 1. 有一说一，上海城市实力的确强，但就这次防疫，还真比不过深圳。上海那个疫情外溢到十几个城市了，有啥资格吹他们的精准防控的 |
| 1. 所以上海的孩子这么倒霉就要趴着桌子上睡好几个晚上 |
| 1. 说的不错，等上海绷不住了，我再给你点赞 |
| 1. 他们运作本地居民背锅 |
| 1. 没有市ZF，锦江敢S操作吗？ |
| 1. 回复@Cindy628383:也是在上海，今天做第三次，还是咽拭子 |
| 1. 回复@央吉的缤纷四季:上海做鼻拭子真的差点给我天灵盖都捅穿了 |
| 1. 回复@loe_:你是小区 我们那是集中隔离 我进去之前从没做过那么酸爽的核酸 |
| 1. 回复@羊小二在sz:隔离点的鼻拭子可和普通的不一样 从鼻孔一路捅到咽喉 你想象一下就知道有多痛苦了 |
| 1. 回复@羊小二在sz:隔离的肯定是鼻子，准确一点 |
| 1. 回复@loe_:坐标上海我们们小区48小时封闭管理，是鼻拭子和咽拭子一起做，五岁以下的才能只做咽拭子 |
| 1. 回复@loe_:在上海过年之前被隔离每天都捅鼻子 |
| 1. 回复@央吉的缤纷四季:上海是鼻拭子？难道我们小区是例外？我怎么做的咽拭子 |
| 1. 回复@羊小二在sz:见过深圳密接者住的小区的小朋友做鼻拭子，确实一个个哭得稀里哗啦，大人都难受更别说小孩了 |
| 1. 回复@央吉的缤纷四季:隔离点没去过，不知道。深圳社区大规模确实是不需要鼻拭子，好像就密接或有高风险的才会鼻拭子。社区上门做过一次 |
| 1. 回复@羊小二在sz:因为深圳采咽拭子，上海釆用鼻拭子。谁试谁知道 |
| 1. 至于嘛，深圳小区里孩子那么多，从没见过因为核酸嚎叫的 |
| 1. 目前公寓方提出赔偿一个月房租+搬家费，我们还在继续争取公开道歉及其他合理赔偿。 |
| 1. 上海真是一个有人情味的地方 |
| 1. 上海没自夸，微博上精神上海人可会夸了。 |
| 1. 你是说上海这个城市在买热搜？具体是上海的哪位买了热搜？你是把一个城市当成买热搜的明星了吗？ |
| 1. 做到好的就应该夸，做的不好就该批评，但不能因为之前做得好被夸奖过，就是原罪了吧，只要你之前夸过自己，以后就不允许有任何失误了，否则就得被冷嘲热讽，是这个意思吗？ |
| 1. 并没有 |
| 1. 哦……这次疫情是因为骂出来的，还是因为当初吹逼精准防疫，拿吹牛当政绩，导致现在这样被打脸的。就是你们这群人在捧臭脚捧出的疫情 |
| 1. 对，老老少少在受苦受难，喷子拿钱捣乱，总会有报应的！ |
| 1. 疫情下受苦的更多的是基层百姓，居然还有人拿这个来冷嘲热讽幸灾乐祸。行吧，下个不幸的就是你 |
| 1. 回复 @勇者哥哥000:你这么气急败坏现实生活是不是过得很惨啊？ |
| 1. 上海这城市，做媒体的人多，臭不要脸自夸的多，年初的时候封个几平方奶茶店，自吹精准防疫，买了几天头条和热搜，恶心得一匹，现在被打脸，疼了吧 |
| 1. 今天记者会李二哥还是说要更精准防控，央视新闻跟着也说了精准防疫，为什么对4个字那么敏感？除了收钱的会辱骂没有其他人了，中国人会捣乱自己国家吗？ |
| 1. 有毒 |
|  |
| 1. 回复@皮皮鲁523:也不知道雪中送炭还是雪上加霜 |
| 1. 防不胜防 |
| 1. 笑死 |
| 1. 瑞丽真是，牺牲一座城，保全大家 |
| 1. 对啊，只有三分之一的人还在坚守着 |
| 1. 臭不要脸的，接手香港人的大部份都是大湾区城市，偷渡的也一堆，上海才接手几个香港人，而且都是坐飞机去不是偷渡的，也搞得一团糟，真的没能力就不要吹那么大的牛逼，到时候被打脸真的很疼 |
| 1. 可不只是这一次啊！ |
| 1. 是你自己多管闲事，西安我们丝毫不关心 |
| 1. 这个你就不操心了，管好自己就行 |
| 1. 现在香港航班也要过去了，加油 |
| 1. 从发现第一天就开始了防控，目前确诊的也都是集中隔离里发现的，社会面没有，懂么！1号到西安，5号走的时候核酸才发现，这期间接触了多少人，现在都隔离了，发现的也都是隔离中的 |
| 1. 怎么防不住呢，那就对了，大家都一样的。 |
| 1. 在哪能看到 |
| 1. 上海发布辟谣了，一天以后又承认了，真是啪啪打脸 |
| 1. 是的，我也看到了 |
| 1. 你说的就是个屁话！上海不能去？什么高贵的地方去不得？？？？？ |
| 1. 来上海干嘛呢 |
| 1. 那你猜为什么本来计划歇业的宾馆能作为隔离宾馆收钱了？ |
| 1. 我觉得是人性扭曲吧，这种情况就像你去上海工作还不如回老家安稳生活一样的呢，你跟这些亲戚说不清楚，亲戚也巴不得你过得不好。还有一些是真觉得上海做的不好，一向被表扬的尖子生做的还不如别人，不能忍，必须要提点他几句。 |
| 1. 一直是这样的，喜欢就好，酸 |
| 1. 我们在深圳，至今还没开学，疫情严重，还是在家吧，读书来日方长 |
| 1. 上海的学生学业压力很大的 |
| 1. 还80分 直接不合格 |
| 1. 哎 |
| 1. 对，民众素质很高，也很配合 |
|  |
| 1. 你NC吧，跟张文宏有什么关系？市里决定接受高风险地区过来的在华亭隔离！张教授能做的了主？说不？ |
| 1. 微信圈里很多华亭宾馆，浦东教育局不让老师跟学生家长说全民核酸，就说做核酸，不能出现全民核酸的字眼真的是上海人最后的倔强 |
| 1. 啥聊天记录？ |
| 1. 大胆这你也敢说？不怕围攻？ |
| 1. 华亭本来没开空调然后隔离的香港人觉得太冷就去投诉要开空调结果就开了空调香港人也是脑子有问题的 |
| 1. 上海全部停工可不是上海说的算，北京不会同意的，上海要全停工得损失多少经济？ |
| 1. 所以现在所有外省市的阳性病例都确定都是从上海外溢出去的么？有新闻链接么？ |
| 1. 所有用电脑的职位，应该强制远程工作 |
| 1. 5.5的flag都立好了上海封了经济崩溃你负责啊 |
| 1. 你有考虑过上海停工会有多大的经济损失吗？其实很多人并不怕疫情，他们更怕吃了上顿没下顿。疫情以来失业的人够多了，这种经济中心说停就停，那些失业的人谁去资助他们？ |
| 1. 镀金镀过头 |
| 1. 反正我们老板下刀子也得来上班的 |
| 1. 支持 |
| 1. 为什么还不和香港熔断，天天看的气死人 |
| 1. 我们单位一个项目停工一天几十万一个公司二十几个项目全部停工一天我们公司就没了……听不起的 |
| 1. 华亭宾馆就是既然要大修，不然拿出来隔离。不用白不用。绝 |
| 1. 确实啥比 |
| 1. 我们公司不但工资不发，还要员工冒风险去上班，明明是可以居家办公的工作性质，还要通知正常上班，也真的是无语😓 |
| 1. 因为基层努力把事情做的很好，领导就会觉得理所当然，还会继续给你加任务…中国企业都这样 |
| 1. 全停工了就有人骂一刀切，不停工就说没担当zf也挺难办的 |
| 1. 问题是，混检没有报告啊 |
| 1. 建议全国居家隔离，学生不上课，工人不上班，商人不营业，政府每日提供生活必须品，每月每人补助3000元，暂停一切房贷车贷，直到清零。 |
| 1. 上海总是活在被别人的评论中 |
| 1. 国家的钱袋子，怎么可能停工，目前情况下钱比人重要，北京会在乎上海的人？ |
| 1. 可以不停工，但能做点有用功行吗 |
| 1. 天天排队的密密麻麻不是为了确保感染面更大吗？还是核酸检测有谁的利益？全阴没完没了封，菜价上了天不管还要封 |
| 1. ¡评论配图 |
| 1. 就是的，磨刀不误砍柴工啊，这样搞来搞去也相当于停工了，城内的人被折腾，城外被带跑 |
| 1. 不带口罩的人员需要严厉处罚打击，不自觉的大有人在 |
| 1. 疫情防控，市长出来讲过话吗？前几天徐汇做做秀而已。普通人疫情初期，感觉都有封控，现在一天奔1000，无症状感染和确诊病例有区别吗？ |
| 1. 一直上班直到确诊 |
| 1. 大胆！你马上要被删了 |
| 1. 转运老人网上一片骂声！不转运出问题了还是一片骂声！反正上海这次怎么都是被骂！以一个支援过疫区的人来讲，超过70岁的老人都应该被转运至方舱观察！一旦有问题从方舱到医院更快！至少至少方舱有监护设备！ |
| 1. 都养老院了，先想想自己为啥不孝吧 |
| 1. 这个四月之声看到朋友圈有人发十几分钟就封掉了，想看看不到视频 |
| 1. 没躺平都这样了，如果当时真的躺平，上海同胞岂不是要处于更为坎坷的境地。躺平派 |
| 1. 不清零，奶奶起码能马上送医院，家人起码能见最后一面 |
| 1. 新冠只是导火索，上海管理层才是炸弹。换任何一个需要抢救的急诊患者，要等2个小时的救护车！9条命都不够等吧？ |
| 1. 目前看对上海人这个omicron真的真的不是大号流感 |
| 1. 博主节哀这操蛋的防疫政策 |
| 1. 还是认为上海管理层用绑架百姓的方法来软抵抗中央动态清零的政策，表面上是更严格的隔离，而一切支持和支援隔离的方法和举措都人为设置障碍，比如抽空医院，拒收病人，阻碍京东等进城恢复供应，不当使用友省援助...从市政府到基层，干不了的赶紧让位，从中获利的干部会被清算的 |
| 1. 博主节哀奶奶还是要多注意多保重 |
| 1. 我外公昨天晚上也在上海去世了没能等到120没机会看病明明不是大病硬拖成这样我们全家守在养老院楼下不给上去连最后一面都没见到直接拉走火化没有追悼会解封后领骨灰如果防疫真的是为了保护老人那为什么现在为了防疫在草菅人命呢 |
| 1. 问题不是清零是腐败的政府在草菅人命 |
| 1. 看这条因为没能及时就诊老人去世的微博下还有这么多争论躺平还是清零的我就知道这个社会魔怔了楼主节哀这个倒退的时代太不幸了 |
| 1. 帮转，宝贝节哀养老院真的求助无门，离我家不远的一家养老院，浦东新区市南养老院，附近的小区都能听到半夜老人的求救声 |
| 1. 所以那个始作俑者还在论坛高谈阔论 |
| 1. 上海市虹口区虹德养老院。节哀@上海发布@上海虹口 |
| 1. 打过疫苗的话，就是死于基础疾病。没打过疫苗，就是死于新冠。 |
| 1. 抱抱 |
| 1. 120在卖货……O网页链接 |
| 1. 出现大规模医疗挤兑，这都是不可避免的。唯一避免的方法就是初期控制，可惜上海没这么做啊 |
| 1. 然后接下去都没有追悼会直接火化，别问我怎么知道的 |
| 1. //@宝树:不排除一开始放水的可能，后面也不用太阴谋论，就是失控了神仙难救，有些黑心的想趁机发财是另一个问题//@蘑菇目前不改名啦:我真怀疑上海就是故意的，目的就是诱导更多人去攻击现有的防疫政策。毕竟三年了啊，三年了，是头猪都该知道一线城市正常的水平是啥样的啊//@敦煌郡公 |
| 1. 电车难题：学欧美放任，受难五个人与政府无关，主动封控管理，受难的一人这与政府有关。前者可以说无可厚非，后者责任明确，民主议会制国家为了选票肯定选择前者。而主张“王无罪岁”的中国不一样 |
| 1. 必须清零，不清零更多惨剧 |
| 1. 唉奇葩操作看不懂节哀顺变O网页链接 |
| 1. 节哀，照顾好奶奶 |
| 1. 基础病不是推卸责任的理由！慢性病人，慢性病老人为什么就成为这次事件的牺牲品！太难过！难道只有年轻人才配在世间上吗？🙏🙏🙏 |
| 1. 玩精准玩砸了 |
| 1. 节哀 |
|  |
| 1. 节哀，太伤心了。 |
| 1. 博主请节哀 |
| 1. 抱抱博主，节哀 |
| 1. 真的搞笑了，我外公外婆两个人住在长兴岛，从从封城到现在，没人管过他们，没送过物资，做了一次核酸，没有问题后就没管过了，政府没有人了吗？ |
| 1. 博主节哀 |
| 1. 节哀，奶奶保重肿瘤病人也很危险 |
| 1. 疫情是美国的人类清除计划 |
| 1. 随申办-互动-领导信箱-信访投诉养老院12345没用，养老院归民政局管，医院归卫健委管，我就是这么投诉的 |
| 1. 配药我们都说买药 |
| 1. 做试点我信，但是做之前不需要做好准备工作？？？把老人急诊病人都随便扔进垃圾桶就开始试点了？？？//@兴风作浪的大叔777:给全国人民做试点是上海这座城市的历史使命，你们现在可以骂日后心里会明白//@肥yaya的Freya:换任何一个需要抢救的急诊患者，要等2个小时的救护车！9条命都不够等吧？ |
|  |
| 1. 市长的责任最大不仅要撤职还要她赔偿上海的损失 |
| 1. 博主节哀。 |
| 1. 节哀，人在做，天在看。 |
| 1. 这笔血债我看他们怎么还 |
| 1. 要么就老老实实的清零，要么就躺平，上海以为自己很屌，结果全国最辣鸡 |
| 1. 节哀🙏🙏🙏 |
| 1. 真的是这样，根本没人管 |
| 1. 真几把6啊都21世纪了，防控出了这么多乱子，好像还有人饿死，真是魔都 |
| 1. 用软政策兑付zy |
| 1. 这图也夹？？？？？ |
| 1. 按最近的新冠死亡统计来看这个计入新冠死亡因为需要一些死亡病例来引起足够的重视但会强调高龄和基础病 |
| 1. 图怎么又看不见 |
| 1. 总之，都是错！//@狗狗156:转运老人网上一片骂声！不转运出问题了还是一片骂声！反正上海这次怎么都是被骂！以一个支援过疫区的人来讲，超过70岁的老人都应该被转运至方舱观察！一旦有问题从方舱到医院更快！至少至少方舱有监护设备！ |
| 1. 参考香港数据，躺平后养老院就是重灾区 |
| 1. 老龄化严重，干掉领养老金的人 |
|  |
| 1. 博主节哀！另外！博主提出这些质疑是希望医疗救治流程相关机构管理更加合理高效，并没有质疑清零政策。清零政策和合理高效的病人转运救治并不冲突！别一上来就清零不清零的，清零不该以这些无辜的生命为代价，不是任何对现有流程的质疑都是在质疑清零政策本身！可真是刀没砍在你身上你不疼，善良点吧。 |
| 1. 上海市政府有多少官员的子女可都在国外，美国躺平可以有免费特效药，所以上海市委受西方国家的影响特别大，上海人民特别有钱，为辉瑞贡献2300简直就是小case。我们西部地区的穷，买不起辉瑞，所以不敢躺平 |
| 1. 有基础病的，得了新冠的，去世按基础病算，不按新冠算，为了控制新冠死亡数字。 |
| 1. 深圳现在新增不多都要求72小时核酸，没有核酸哪里都去不了 |
| 1. 抱抱你。 |
| 1. 上海的官到底什么背景，这都没给他们换了 |
| 1. 上海的朋友们还是认真的配合政府做好封闭防控吧。 |
| 1. 把老人放养老院是怎么想的，钱多差口饭？确实是轻松不用照顾！ |
| 1. 如果全国到时候每天死几十万人，不知道bbc会如何报到 |
| 1. 很担心养老院的亲人，我们尿片也快要断档了~每天张眼就京东美团~完全不敢想现在什么状况。 |
|  |
| 1. 急诊等不到120，进不了医院。生活在这样的城市，要多绝望啊 |
| 1. 还是那句话，疫情期间，有能力的都接回家养老吧。经历过深有体会。 |
| 1. 评论区没有带脑子上网的吗？ |
| 1. 广州两周清零，上海整个管理层烂透了！ |
| 1. 真的是达尔文主义吗？ |
| 1. 不知道说什么，节哀 |
| 1. 疫情都三年了、怎么还能乱过最初的武汉状况 |
| 1. 节哀🙏 |
| 1. //@鲁镇的柜台:我心目中中国最美的城市，加油//@直播上海: |
| 1. 烂透了，烂透了 |
| 1. 我外婆98也是因为先阳了不给送医院最后在家咽下最后一口气，一个亲人都没见到 |
| 1. 都是因新冠去世的，这些人那些人。 |
| 1. 节哀，不知道情况这么严重 |
| 1. 魔都真成魔鬼之都了 |
| 1. 太难过了，博主你要小心，昨天又有临观的工勤阳了，你知道我说的是哪里的临观。 |
| 1. 没进医院去世的都不算新冠死亡 |
| 1. 大城市对于老年人养老不友好，好的中小医疗条件的城市才适合 |
| 1. 真的难受了 |
| 1. 这是回到文革了吗？ |
| 1. 直接不找中央有毛用哦 |
| 1. 难过也要照顾好奶奶呀 |
| 1. 投诉他们 |
| 1. 我好难受啊 |
| 1. 节哀，不知道应该说什么，只希望一切快点过去 |
| 1. @今天也要喝冰阔落 |
| 1. 心难受节哀愿您奶奶安好 |
| 1. 告死他们 |
| 1. 节哀。 |
| 1. 真的看哭了 |
| 1. 节哀。诶…好心疼 |
| 1. 唉！🙏 |
| 1. 抱抱博主 |
| 1. 还要折磨多久 |
| 1. 🙏🙏 |
| 1. 🙏 |
| 1. 节哀节哀节哪门子哀啊！？该追责的要追责，绝不可轻饶 |
| 1. 无能的天朝 |
| 1. 节哀，希望那些不负责任的人能受到惩罚 |
| 1. 节哀，保重 |
| 1. 节哀希望奶奶身体健康平安 |
| 1. 🙏节哀也保护好自己 |
| 1. 节哀顺变。 |
| 1. 原博主节哀 |
| 1. 抱抱你 |
| 1. 我的天… |
| 1. 博主节哀… |
|  |
|  |
| 1. 这就是上海人选择躺平的后果 |
| 1. 关键是上海这种还不算是新冠死亡，瞒着国家 |
| 1. 都看不到图片的 |
| 1. 节哀🕯️ |
| 1. 这是还没完全躺平医疗资源就挤兑的这么严重，共存了还不知是什么样了。 |
| 1. 怎么好多被封了？ |
| 1. 哎...🙏 |
| 1. 香港向中央请求支援医护的新闻你真的看不见吗？我们大广东派了上千的医护去香港，连张忠德都去了//@白羊座辰辰:回复@Paynard:香港医疗资源挤兑了吗？我看到的数据是他们感染数里8%的没接种人群，其中死亡16%。前段时间算了算他们的死亡率超过2%，不过我一看甲流死亡率超过7%，就不担心了 |
| 1. 缴纳了医保，叫不到救护车他们违约了 |
| 1. 其实很多人是不是把“动态清零”政策和目前上海的“防疫措施”混淆了，作为一个生活在上海的人，支持动态清零，但是目前的这种“防疫措施”尤其是前期的一刀切带来的次生伤害已经超越了病毒本身，很多需要看病求生的人，那可是一条条鲜活的生命就这么因为耽误治疗而逝去了！ |
| 1. @宣克炅@上海电视台案件聚焦@北京青年报@人民日报@新华社@北京晚报@澎湃新闻@上海发布 |
| 1. 上海的医疗挤兑比武汉还厉害，武汉60岁的老人方舱都不收，直接进医院。//@狗狗156:转运老人网上一片骂声！不转运出问题了还是一片骂声！反正上海这次怎么都是被骂！以一个支援过疫区的人来讲，超过70岁的老人都应该被转运至方舱观察！一旦有问题从方舱到医院更快！至少至少方舱有监护设备！ |
|  |
| 1. 血的教训，不能躺平，保佑家人平安🙏 |
| 1. 虹口一塌糊涂，别来虹口 |
| 1. 太多的老人在疫情期间离去，不能办追悼会，甚至有些没穿上寿衣，非常无奈。 |
| 1. 四月之声没有办法被听到的人就此湮没 |
| 1. 不知道怎么安慰你，抱抱 |
| 1. 我们这边即使是很早之前就已转为低风险地区，但是依旧严控市外来员，甚至很多村镇都在劝回家参与丧事的人离开，不让入村。严防死守至此，我不能理解。 |
| 1. 上海再不嘚瑟了，之前还一直嘲笑别的地方的防控措施，现在直接被点名了 |
| 1. 21年，上海承接14137架次国际航班，占入境总量35.6%。今年前两个月1972架次占41.2%。一线、隔离酒店、公卫中心满负荷运转，这样的日子，上海过了三年，上海疏忽了、也累了，但我们坚信他会历劫归来，而所有失去的，都会以另一种方式归来 |
| 1. 奥密克戎真的狡猾，潜伏期又长，为了自己还是老老实实核酸吧。 |
| 1. 上海认为其他城市“过度防疫”“防疫一刀切”不对，作为国际化大都市要做个表率，要“精准防疫”“科学防疫”完全没问题，但不能以把风险转嫁给其他省市作为代价。其他省市老老实实“从严防疫”，多少轮全市核酸，好不容易稳定下来熬过来了。因为“精准防疫”的漏网之鱼，努力付诸东流，谁心态不崩？ |
| 1. 面对疫情防控，多些理解，少些埋怨，疫情防控需要每个人的配合。 |
| 1. 这数据真实吗#上海六院# |
| 1. 没人想吐槽上海疫情，但是上海疫情外溢到外省是不争的事实。而现在上海公布的感染人数，让人觉得不符合常理，实实在在的合理质疑。希望上海和全国各地的疫情得到控制，大家越来越好。不要把自己架在那，搞得自己现在下不来台！ |
| 1. 上海的无症状感染是真多啊，都不是确诊吗？ |
| 1. 上海六院什么情况？没有一家大v出来发声？ |
| 1. 上海的确诊和无症状比例永远与其他省份与众不同 |
| 1. 每天都看到奋战在一线的大白们和120，这个时候就听话，出门戴口罩，少去人多密集的场所。上海，会好起来的！ |
| 1. 精准防控的理念是没问题的，只是管理没有达到，提前嘚瑟打脸了。 |
| 1. 无症状，全国就上海无症状多，怎么上海人民打的疫苗是上海政府研发的 |
| 1. 上海六院到底有没有瞒报，护士到底有没有受委屈。请给人民个交代@上海发布 |
| 1. 江西已经三例全关联上海，可想而知当地有多严重 |
| 1. 说上海境外输入压力的，可以考虑一下深圳的感受，另外，第一入境点可不只是上海一个城市，除非有2021年全年入境排名数据，所以就不要拿这个洗了¡评论配图 |
| 1. 看看六院救救妈妈#上海六院# |
| 1. 听到街上的救护车，真感觉这次感觉疫情真的很近，决不能掉以轻心啊！ |
| 1. 一直这样管控经济毁了，没钱纳税，而且很多人失业，公司关门，生意难做。所以说，慢慢还是要阶段性的疫情管控打开，对国外也打开，一直这样管控也不行。欧美早就全面打开恢复正常了。（不是亲美，只是理性去说，有些人不要听到建议疫情放开就一直喷，除非你家里几个亿） |
| 1. 上海六院呢？ |
| 1. 现在小区封锁程序太乱了，居民都跟惶恐 |
| 1. 上海也从来没说过自己是模范生啊，嘲讽啥呢？HK的同胞们应该给其它地方平摊一点，上海也就没这么大压力了 |
| 1. 这个数据是可以相信的嘛？？？ |
| 1. 无症状转的，也算意料之中 |
| 1. 上海赶紧封城吧，全部人员禁止出门，居家隔离知道全部清零为止 |
| 1. 惊雷哪去了？ |
| 1. 医生打人的事情说一下？ |
| 1. 这个数字包括六院了吗？他们怎么样了？ |
| 1. 上海新闻北京头条报道。。。 |
| 1. 应该这样报道，新增本土阳性病例61例（其中确诊病例6例，无症状感染者55例。） |
| 1. 尼马当大家是傻子吗，海上玩什么文字游戏，有就是有，希望你们全员核酸检测 |
| 1. 上海发布就是个笑话 |
| 1. 上海：我不查就没有，你非要让我查就是无症状 |
| 1. 在看发布会很喜欢吴凡每次都像妈妈一样虽然很能说但是说的有血有肉，苦口婆心，把事情说清楚，能感受到是真的希望大家一起努力早点好起来 |
| 1. 疫情不可怕，瞒报才可怕，某些领导为了自己的官帽真是丧尽天良 |
| 1. 疫苗接种率提上去，轻症无症状没必要占用医疗资源，自行在家隔离就好，恢复阴性就自由活动，最后还是会从动态清零走到共存这一步不信走着瞧，防不住的（奥密克戎传播率高，但毒性小，得新冠就像感冒一样而已了，别说什么后遗症，除非你没打疫苗）杠就是你对 |
| 1. 看到六院的信息，请关注 |
| 1. 关注一下这个事情吧O网页链接 |
| 1. 看来六院的情况不能一直这样遮掩下去，如果是院内感染，将是严重的问题。 |
| 1. 最不要脸的就是上海市，精准它奶奶个腿，不知道外溢多少个省市了，还天天牛逼哄哄的不行，呸！ |
| 1. 总结一下沪城蛆的抗疫表现1；2次拒诊孕妇，攻击孕妇没有去对医院。2；一码通崩溃，狡辩那不是一码通是云健康。3；外溢是好事，打破精准防疫神话，探寻新模式。4；赌运气赌赢了就是优等生，赌输了也是学霸只是这次没考好，外溢啦。5；控评，删评，上海yyds。6；今夜我们都是上海人。 |
| 1. 这波来势汹汹，大家做好防护 |
| 1. 直接说61例不就行了 |
| 1. 我们这昨天就因为一个上海回来的，直接整个市都进入了紧急状态。上海应该自我反省，吉林省长都撸了，上海撸谁？ |
| 1. 这几天最大感受：当初真别骂武汉了，按那个时候的情形搁哪个地方都是烂或者更烂。没有净土。 |
| 1. 要求跟香港全面封关，内地人的命也是命，生而为人凭什么香港人就要比内地人高人一等？香港自己不自救，而且还不接受内地的帮助，还歧视我们的医护和护工，甚至连杀猪匠都被歧视，这是要把整个中国都祸害了吗？你们这样做，经过内地的老百姓同意吗？香港是个什么东西，值得你们这么下跪？ |
| 1. 今天去做了核酸，防控从我做起 |
| 1. 顶上去//@神之近卫军:21年，上海承接14137架次国际航班，占入境总量35.6%。今年前两个月1972架次占41.2%。一线、隔离酒店、公卫中心满负荷运转，这样的日子，上海过了三年，上海疏忽了、也累了，但我们坚信他会历劫归来，而所有失去的，都会以另一种方式归来 |
| 1. 建议上海来次全民核酸 |
| 1. 不要瞒报谢谢！ |
| 1. 但凡在上海的人就不会喷精准防控 |
| 1. 朋友中招了一个学校确诊几粒都没报发烧26小时才有救护车来拉他到医院没有医护人员管他…他隔壁同事确诊了太吓人 |
| 1. 不要再打肿脸充胖子了，捂不住了，赶紧好好做核酸吧 |
| 1. 上海是精准防控还是精修防控？ |
| 1. 数据漂亮！不管你信不信，反正我是不信！ |
| 1. 这么漂亮的数字 |
| 1. 定位徐汇靠近闵行周围真的蛮严重肉眼可见的比疫情第一年严重 |
| 1. 坚持防疫，人人有责 |
| 1. 所以为啥大学做核酸不让拍照，为啥不让社区发全员核酸字眼的通知，解释一下，没人会觉得推翻错的理论丢人，大难临头大家都是中国人 |
| 1. 上海能不能居家办公啊？？起码一些可以居家的职业能不能让居家啊少点人口密度也好啊？！！！！ |
| 1. 上海孕妇流产，六院，上海红楼，上海地铁夹死人，疫情一直外溢，热搜撤的快当别人不知道呢？还给外地买热搜。钱是真多。 |
| 1. 精准防控 |
| 1. 大夫打护士的事不解释解释？？ |
| 1. 其实就是61，没必要分开的报的。上海加油啊，希望我们中国的每个省都能控制住，每个人都能平安！ |
| 1. 华政的到底什么情况，为什么还没通报 |
| 1. 遏制源头，严防死守。早日清“零”，上海加油！！ |
| 1. 上海是不是独立啦？上海不是中国的吗有些人嘴巴干净点吧小心孽力回馈 |
| 1. 心疼医护人员，我相信只要积极配合做核酸，就会好起来的 |
| 1. @北京头条好大胆子，本来人上海捂得严严实实的 |
| 1. 护士有没有被打？？！！？？？ |
| 1. 上海精准防控上海精准舆论防控上海微博会员大户 |
| 1. 配合工作做好核酸，在家不乱跑，一线工作人员太辛苦了 |
| 1. 想知道上海六院的具体情况，这个时候还瞒报要出大事的 |
| 1. 我寻思这难道不是当时被吹精准防控，然后一帮沪吹和沪黑就开始吵，然后把zf驾到那个高度上去了出了问题又下不来。我就没见过我身边的上海人吹防控，我只想骂不给说话捂我的嘴，上海的领导什么时候能把骂的评论放上来，而不是只回复夸奖的话？有点本事行不行？别连累老老实实的上海人给你买单 |
| 1. 我算是看明白了，哪个地方有疫情那个地方被骂🙉 |
| 1. 这里没有控评，看到的都是真实的声音 |
| 1. 为什么咱们总是学着千年不变的传统《装体面》，却总是埋没事实的真相！ |
| 1. 上海“精准防控”（怕不是“政治防控”吧）不要吹，低调一点。自己的城很放松，搞得别人的城负担很大。 |
| 1. 建议以后别分有无症状了，有啥意思，无症状还不是会传染人。 |
| 1. 求求别瞒报了 |
| 1. 笑死每次都搞这种没满24小时偏少的数据上热搜，第二天完整数据是几倍，有意思吗？粉饰太平？ |
| 1. #上海新增本土确诊6例无症状55例#为啥无症状比确诊的多的多呢？ |
| 1. @超级多余余 |
| 1. 还是这里的评论比较正常，其他很多媒体都精准控制舆论了 |
| 1. 都这样了还不通知居家办公！ |
| 1. 希望新冠患者和其他患者早日康复！ |
| 1. 上海对于疫情的态度真的是下头。光说外地这不行那不行。这次西安疫情就是上海来的。还我们平安健康和自由。 |
| 1. 只希望给医护人员的保护做到位，物资各种！ |
| 1. 远不止这点吧 |
| 1. 加油🙏 |
| 1. 作为社会人，自然要聚集，长期的隔离，生病态心理...... |
| 1. 上海防控疫情做的还是好的！ |
| 1. 江苏自媒体联盟超话 |
| 1. 可怕 |
| 1. 应该远远不止 |
| 1. 上海加油鸭，共同抗疫，一起战胜疫情！ |
| 1. 上海加油！ |
| 1. Hk这些不孝子回来干嘛 |
| 1. 俄乌战争之际，中国多地出现疫情？事出反常必有妖，很明显是有人在搞事。一些媒体唯恐天下不乱。......疫情是人为的，存在严重数据造假，无中生有。希望早日将那些搞事的人绳之以法，不能一直让它们兴风作浪！ |
| 1. 疫情是人为的，存在严重数据造假，无中生有！ |
| 1. 笑死，疫情严重开始比谁更烂，内撕了，好幽默的评论区！ |
| 1. 俄乌战争之际，中国多地出现疫情？事出反常必有妖，很明显是有人在搞事。一些媒体唯恐天下不乱。疫情是人为的，存在严重数据造假。希望早日将那些搞事的人绳之以法，不能一直让它们兴风作浪！ |
| 1. 谁都不想看到疫情，两年了，病毒在进化，防不胜防。希望大家都平平安安的。现在的网络上戾气太重了。 |
| 1. 上海精准吹牛逼哪次没给北京送除了会舆论 |
| 1. #上海新增本土确诊6例无症状55例# |
| 1. 严防。 |
| 1. 强烈要求境外航班放到外地，上海真的累了，都是中国城市凭什么要上海承担 |
| 1. 无症状感染多说明症状轻微。 |
| 1. 这数据………真实吗？ |
| 1. 徐汇可以居家办公吗 |
| 1. 脸呢？ |
| 1. 我们要知道真相 |
| 1. 没有惊雷心好慌 |
| 1. 不明白为什么哪个地方一有疫情就要被骂！长春人在上海，有疫情我也没慌过！境外飞机多少落在上海？上海前几次疫情总人数多少？多少天结束的？被隔离的人生活有没有得到保障？！只要有密接，第一时间找到，立刻全楼核酸！我之前广州出差，回上海后广州有疫情了，社区立刻打电话做登记，这效率要被骂？！ |
| 1. 前两年上海疫情控制还算不错，估计产生了麻痹思想，被病毒找到漏洞，这下玩了个大的，居然传到了大学校园，这下是不是该启动问责机制了，吉林广东官员够处理了一大堆，上海呢？ |
| 1. 病毒会变异，人性也会变异。 |
| 1. 我有个想法：何不研发一种能重复使用的快筛试剂，发给每个家庭或者每一个人一只，每人每天自己在家快筛一次，阳性的就自己上报。 |
| 1. 讲真，外地人真的要感谢上海帮你们挡住了疫情，而不是忘恩负义，攻击阿拉上海 |
| 1. 上海春考面试怎么办春考也是高考啊几千学生的命运和时间在上海市教委眼里都不重要是吗 |
| 1. 别的不说但是上海最早最自主人性化让宠物和主人一起隔离开始就比其他地方强，特别是你北京当互联网没记忆么 |
| 1. 这破疫情什么时候是个头啊！赶紧结束吧！！ |
| 1. 一个用嘴防疫的城市，现在全国疫情爆发，必须追根溯源。 |
| 1. 建议上海全民做核酸检测 |
| 1. 上海疫情上了几个热搜，紧张ing，希望赶紧控制住。 |
| 1. 真的有这功夫做好防疫不比啥强，成辈子就爱搞地域黑//@妖女YY:上海再不嘚瑟了，之前还一直嘲笑别的地方的防控措施，现在直接被点名了 |
| 1. 上海，可以啊，管的不错 |
| 1. 这都吵啥吵呀，这种时候要静心，少折腾，交给专业人士，管好自己，在家没事打打八段锦适当锻炼适当吃饭保持自身身心健康 |
| 1. 一直删帖压热度当网友眼瞎是吗 |
| 1. 上海加油！！ |
| 1. 感觉这样也不是办法，还是放开吧 |
| 1. 哎 |
| 1. 北京头条报道你北京吧啊，上海你管不着 |
| 1. 我们没有逞能我们也不想逞能我们只是做了全国想做的事情防疫两年精准防控都是做的很好如果一刀切两年可想而知这次上海的疫情上海发布也承认了境外输入的防控管理疏忽我们勇于承担错误及时止损学校停课公司停业为现在站在防疫一线的医护人员警卫人员说一声你们辛苦了 |
| 1. 经历疫情两年多了，医院起码有必要的储备物资来应对突发情况吧，而不是慌乱抓瞎，医护人员都不容易，疫情当前，首先保护好自己才能帮助更多的人。 |
| 1. 上海的表现毋庸置疑，已经做得很好了，希望疫情早日结束！ |
|  |
| 1. 上海这回真的一团糟了，不知道咋精准的，精准到千家万户？ |
|  |
| 1. 加油 |
| 1. 隔离真难受，快点过去吧 |
| 1. 我们国家突然的疫情爆发到底是咋引起的？ |
| 1. 上海最近辟谣多，无症状感染者多，确诊病例少。 |
| 1. 好 |
| 1. 路过 |
| 1. 啊 |
| 1. 上海精准防控，精准投放吧 |
| 1. 防疫长备不懈！ |
| 1. 我感觉美国又给我们投毒了 |
|  |
| 1. 顶 |
| 1. 自己注意比啥都强 |
| 1. 挺住 |
| 1. 你好 |
| 1. 为了一个逆子害了两个最强的城市群 |
| 1. 南京的就是上海过来的不要杠，已经通报了 |
| 1. 疫情啥时候才是头啊，真是揪心 |
| 1. 评论真是诠释了什么叫孽力回馈 |
|  |
| 1. 你多久 |
| 1. 已经点不了“赞”了，太神奇了 |
| 1. 下面由院领导发表下岗前的最后陈述。 |
| 1. 把上海封了吧真的 |
| 1. 战胜疫情 |
| 1. 全国一盘棋，不要地域黑 |
| 1. #上海六院疫情#，这是徐汇区有些人决策的问题，六院的医生护士已经很辛苦了。是谁决定的人口密度高的居民区附近的老旧宾馆可以作为隔离点的？因为不止华亭。田林宾馆也是老旧宾馆，宾馆后面后门连着田林三四村人口密度极高小区,做这样决策的人到底是谁？这到底是天灾还是人祸呀？不要转移问题的本质，O网页链接 |
| 1. 如果单纯是感染对人体伤害不大的话，非常想知道现在的重症率和死亡率 |
| 1. 加强防控 |
| 1. 真相呢！ |
| 1. 好的 |
| 1. 上海人民，在这高高在上没啥用，拿出真本事呀，做个防疫标兵呀，让全国人民也瞧一瞧么。光在这卖嘴有啥用呢 |
| 1. 疫情防控守土有责人人有责！！ |
| 1. 全国任何地区有疫情都是我们不想看到的，为什么会有这么多人在这阴阳怪气的幸灾乐祸呢？素质可见。 |
| 1. 评论真的是一塌糊涂，一有疫情就开始怪罪从去年西安疫情及冬奥会前后上海很多时候境外输入病例比整个广东都要多，最近一两个月大部分都是香港来的，但其他省市的人可不会这么讲逻辑，他们从不看上家是谁，溯源到底又是哪，一提到上海就喜欢集体高潮属实很无奈 |
| 1. 6例怎么算的 |
| 1. 防疫人员最辛苦 |
| 1. 评论里一帮戆乱趁机地域黑天天盼着上海出事这下高潮了以后别说什么上海是全国人民的上海滚了远点 |
| 1. 真奇葩。无症状不也是确诊感染吗？数据这么拆分有啥意思，自欺欺人？ |
| 1. 真累人啊 |
| 1. 上海医疗资源充足，每天新增比杭州还少，没问题 |
| 1. 每个地方情况不一样，人密集度，管理措施等情况也不同，大家还是冷静 |
| 1. 好烦，这一生都要与病毒作斗争了…… |
| 1. 相信上海防疫！那些口出恶言的人真的是。。。。无语！！！ |
| 1. 挨着海外，依仗强大的流掉团队，以及强大的反应机制，就喜欢玩精准布控，玩大了，补救回来也没事，可怜的就是那些受难者，希望上海政府能脚踏实地点，别太骄傲 |
| 1. 一出事就可以看到很多有心理病的人来指点江山，有时间还是多去关心吉林怡情吧，上海这点数量还不会垮掉 |
| 1. 回复@布布曰:无症状那些应该是6Y的吧 |
| 1. 给全国人民演示一下低成本高效率的精准防控 |
| 1. 这确诊和无症状的比例不得不说还是上海🐮🍺 |
| 1. 建议全国居家隔离，学生不上课，工人不上班，商人不营业，政府每日提供生活必须品，每月每人补助3000元，暂停一切房贷车贷，直到清零。 |
| 1. 众所周知无症状不算确诊…… |
| 1. 今天有东川路800号吗？ |
| 1. 我信了 |
| 1. 为什么别的几十条几百条的能上热搜，上海这么多还一次一次被刷下来 |
| 1. 热搜一上就下去了 |
| 1. 不明白上海现在难道还不算严重吗？为什么还不停止流动活动进行全民核酸筛查，就这样疫情怎么才是个头。天天在上海人心惶惶的 |
| 1. 防疫还是看广州吧，都是埋头苦干，从来不敢说别人，被别人说也不会回应。结果只有自己知道，2例外市来的确诊，全民核酸了三次。感谢政府的默默努力，保障着我们！ |
| 1. 作为一个上海人，说句心里话，这次确实有关部门做得不够好，但作为一个居住在上海的民众，天天出门上班，路上看到的民众基本都是规范防护的，也许会在别人看不到的私密空间喘息一下也是无可厚非，但仍然请不要一棒子打死所有的上海人。上海地方不大，但人口密集，也是一线城市。。。 |
| 1. 疫情不停工，为了gdp，脸都不要了 |
| 1. 魔都这么牛逼，怎么上海人不开口了，当时喷别人的劲去哪了 |
| 1. 上海个别领导的无能不足掩盖上海的整体成绩和能力，几倍香港人口，2者比较下，高下立判，相信疫情会很快控制，做到及时透明，有错必改，全国都会支持上海，保护好医生，这些都是我们的战士 |
| 1. 南京就是上海输入的 |
| 1. 上海碰到非常多骑着电瓶车还往后吐痰的，整个后面的人全都跑不掉！希望这种没素质的人早点回家呆着 |
| 1. 常州今天确诊的从上海回来的。江苏省内其他几个城市确诊的也都上海回去的。不懂上海这么多天了都没全排查完而且每天才这几个， |
| 1. 老沪屁股摸不得 |
| 1. 萧山机场你的福气来了哟，球球了，把境外航班境外人员都接过去呗，右边这位你们浙江的乡亲父老说不怕国外输入呢//@红社会组织:球球了，上海以后别装逼了。现在不怕国外输入，就怕上海不认真输出到省外啊 |
| 1. 为什么压六院热搜 |
| 1. 新上海人觉得上海做的以后很好。 |
| 1. 上海加油，有些人不要地域黑，这些天医务工作者和防疫人员，到学生等老百姓都不容易，谁想这样的！动不动就上升到地域黑，有事就怪这怪那，同理心都没有，疫情当下，应该管好自身！国内已经很好了，不像国外疫情严重，还打仗！ |
| 1. //@舌头小舅妈:每天看微博都能看到一堆人在嘲上海什么精准防控了精准外溢精准投毒了不是优等生吗之类的话疫情那么久以来上海也许不是做的那么完美但是也不至于很差吧？那些一线防疫人员真的是很辛苦疫情当前没必要互相责怪大家都命运共同体我们的敌人是新冠而不是中国某个地区 |
| 1. 删帖快哦 |
| 1. 为什么评论要阴阳怪气的互相攻击？什么智商啊 |
| 1. 一天天的，咱能不吵了吗 |
| 1. 看了通报，感觉上海疫情有个特点，无症状感染者特别多。 |
| 1. //@游天地寻龙鳞:回复@吴小呆www:要是北方随便一些城市。要被南方系媒体黑成翔了。//@像花泽香一样菜:上海六院什么情况？没有一家大v出来发声？ |
| 1. 精准控评 |
| 1. 不真实的数据会让医护人员心寒，也会让百姓恐慌，希望这真的是真实数据！ |
| 1. 文字游戏，这些数字用得着这样？不要自欺欺人 |
| 1. 求求了，封锁上海市吧！我们常州被霍霍了。。 |
| 1. 消灭疫情是大家共同的目标，不关乎哪个城市哪个区域 |
| 1. 指不定自己的城市就爆发疫情了。大家谁也别笑谁。谁也别骂谁。共同加油吧。（这b疫情真的累了） |
| 1. 西安人民谢谢你嫩 |
| 1. 六院的都算进去了吗 |
| 1. 搞不完了还！！！！！ |
| 1. 西安这次疫情就是上海航班过来的唉😮‍💨 |
| 1. 这个数据里面感染者的溯源、轨迹、时间、请真实公布！ |
| 1. 黑社会化的人就要用黑社会的作风去治他们，反正后面如果我摸清真的是她幕后搞鬼的话，那我真的不会对她客气了。你也不需要心软，但是前提是要保证她不用坐牢，不是为了她，是为了你以后能出来。我是巴不得她牢底坐穿的 |
| 1. 张文宏大师您怎么看？哦，现在是确认了不是临时工干的！ |
| 1. 你们这些评论有外国人逛微博的话可能给人家看乐了.. |
| 1. 我寻思不是让全国抄作业？朋友圈各种精准防控大v文章？怎么原来是驴粪蛋表面光啊 |
| 1. 震惊😱 |
| 1. 看了有位护士的老公发文如果是真的那简直太可怕了 |
| 1. 全报出来了吗？（纯疑问） |
| 1. 为什么西安人跟上海人能吵起来啊，这又不是什么可以讽刺的点，之前骂西安的也不止上海的吧，想想去年那会上的热搜没几个好的肯定给人印象就不好0.0真是无语 |
| 1. 还记得2020年造谣被抓的人吗？键盘侠们造谣之前想想清楚～ |
| 1. 有援助嘛？ |
| 1. 奥米克隆传播快，除非你永远封国别和地球上其他人来往，不然这是迟早是的事情，这么简单的事情为什么10亿网民就无法理解呢 |
| 1. 就这次来看这大部分都没啥症状。有症状也大多数轻症。这比流感、感冒要好吧。流感也具备传染性。但是抵抗力好的人，在周围都感冒的情况下，就那一个始终坚挺着也不在少数。所以增强抵抗力。 |
| 1. 任何人任何地方可以接受善意的提醒和建议，但不能忍受无理的谩骂和颐指气使的瞎指挥。都是同胞何必如此。这个时候有能力的可以提供专业知识、做志愿者，普通人就做好防护，不乱跑不添乱。这才是我们中国人在困难面前应该做的 |
| 1. @茶世界-辉:福建疫情虽然减少增加，但昨天来看还是新增100例这样下去咋整 |
| 1. 看来各地都放松疫情警戒了不然也不会一个省多点开花全国也是逐步开花呀 |
| 1. 听说需要隔离风控的小区家里的宠物需要无害化处理是真的吗？？？🙏~ |
| 1. 漂亮数据，打脸充胖子没脑子。日本重要城市海关早就关闭境外输入。上海既然还能允许香港，东南亚，欧美疫情灾区进入。这是疫情泛滥的最大原因。境外输入病毒。 |
| 1. 公子，啊哈，为什么，把我小护士打。医生，啊哈，为什么，穿防护服，不敢接触病人。护士啊哈，为什么，这么可怜，医生随意奴役。病人，啊哈，为什么，为什么？我要医生，偏只有护士。为什么，为什么？因为没有防护服。啊哈，为什么，医生，有防护服不敢来看我，护士，啊哈，可怜，没有防护服。 |
| 1. 无语了😓评论里一些狗借着上海户籍又开始吠了 |
| 1. 球球疫情快过去吧🙏🙏 |
| 1. 明天封闭管理今天中午把文职赶回家办公，让一线员工直接在工厂住不要回去（这么热的天没有洗澡啥也没有）还想尽各种办法和zf去沟通开工要封闭式生产，还去搞个通行证可以往返什么三观简直了。疫情第一年也是，偷偷开工被举报了晚上开夜班！丧心病狂。 |
| 1. 医生打护士这事怎么处理? |
| 1. 转发 |
| 1. 无症状这么多，有什么办法，疫情太狡猾，不要遇到什么事情都落井下石 |
| 1. 事实证明上海的防疫政策还是领先的尽管出现了点小问题相信上海政府的能力加油。 |
| 1. 香港人都是大傻杯 |
| 1. 玖富爆雷一年多来，一直不好好兑付出借人本息，一直暗箱操作，从未按要求披露资金兑付进展，不公布真实逾期率，不公布老赖借款人名单信息，不公布催收进度，不公布每月归集多少钱回款多少钱，不公布具体回款比例、人为制造紧张气氛，欺骗甚至恐吓出借人以极低折扣转让债权。 |
| 1. 大上海也不过如此，贪污，腐败，欺下瞒上 |
| 1. 说实话很难过上海瞒报疫情我们也受了很大罪如果要骂请骂那些瞒报的领导和那些素质极差的人，不是所有上海人都很排外那些自以为是的人多了去了要骂请直接骂人而不要带上上海上海不仅仅只有这些差的东西也有很多好的东西包括这些医生护士他们都在一线努力这关系到他们是哪里人嘛？ |
| 1. 这他妈也能地域黑，我真是笑了，微博还真是厕所 |
| 1. 丶 |
| 1. 这个时候还在吹精准防控的人真的智商捉急，从德尔塔开始就是物传人非常严重了，精准防控只能防人传人，上海这些当官的会不懂吗？继续拿精准防控欺骗无知百姓，为自己的玩忽职守开脱？全员核酸有多难，一千多万人的城市有不少都做了n轮全员核酸。上海就是当官的怕打脸呗！ |
| 1. 没必要吧都是一个国家，都是中国人，不要说一些让同胞伤心的话了，某些人看笑话呢，疫情这种天灾谁也不想的，大家戴好口罩注意防范，不要被某些别有用心的人当枪使就行了，团结一致才是炎黄子孙应该做的事 |
| 1. 这都没上热搜，媒体一点风都没有。资本和权利的乐园。不愧是魔都。真魔幻现实主义 |
| 1. 有些人就巴不得上海疫情防控不好，还是中国人吗？在阴暗的角落里键盘敲比谁都响。 |
| 1. 赶紧好起来呀！！！ |
| 1. 上海人出门有好多囗罩都不戴，到处人群聚集，挖控不给力！ |
| 1. 癌症化疗不让去，数据是好看，小区全封闭。只有新冠是病吗 |
| 1. 建议上海目前入境人员加以限制入境@健康上海12320@央视新闻@北京晚报@新知视频@封面新闻@国务院@国家卫健委@上海卫健委 |
| 1. 评论给我看迷惑了，管好自己得了。 |
| 1. 不止 |
| 1. 我觉得在疫情防控期间，最重要的是全国人民一起互相加油、共克难关，而不是搞地区质疑，或者当网络喷子破坏团结 |
| 1. 一直撤热搜，不就坐实了吗？ |
| 1. 上海六院到底什么情况啊 |
| 1. 回复@凌老佛爷不吃素:酸吧。 |
| 1. 6例？这是除了多少剩下的数字 |
| 1. 哦？我还以为是西安呢（后仰 |
| 1. 蹭一下热度，看看华东政法吧，要穷死了，今天校长打饭，如果吃不完的话，我压力很大（虽然我吃的挺多的，可能还不够） |
| 1. 三年了还在瞒报，政绩仕途就这么重要吗？ |
| 1. 看其他报道好像不止看看#上海六院疫情# |
| 1. 瘟疫最可怕的地方是得了瘟疫的人被病痛折磨死，没得瘟疫的人被恐惧失心疯。明明应该一起携手共渡难关的朋友同胞，却互相撕咬、彼此谩骂、底层互害。 |
| 1. 主要上海的房子贵，怎么做都对 |
| 1. 玩的一手好双标！当事人写的文章看了吗？上海发生这样的事难道不是管理混乱？难道没有瞒报情节？怎么西安当初就被骂混乱，无能，追责，到了东南沿海所谓强省市就是鼓励，加油！舆论都掌握在南方媒体手里，自己出事就各种隐瞒安慰和bbc,cnn一丘之貉 |
| 1. 我就在上海，这个数据我都不信，隔壁小区就有确诊的 |
| 1. 每个入境人员好像都是祖宗对他们太好了吧！全国十几亿人都要为他们买单。但他们是回来放毒的，两年多了病毒都变异了好几代了，隔离措施还是两年前的哪能不出事才怪呢？还在市中心，就像在人群中放了颗定时炸弹随时爆炸。 |
| 1. 群众监督，舆论发酵，哪一个是压热搜能捂得住的？ |
| 1. 有这嘲讽的劲，为啥不去当志愿者，把劲都使出来转化成服务！全国抗疫一盘棋，都好才是好！ |
| 1. 现在全国各个医院领导基本都把抗疫当作捞功绩的好手段对于一线医护人员不管不顾 |
| 1. 我真笑了大众合理质疑不行吗？防疫出纰漏了这不是事实吗？上海确实在发展中承担了很多，同时也不要忘记它本身也赚的盆满钵满，不应该将这一点拉出来作为疫情外溢、疫情爆发的托辞。我们不满的点从不在于人民，而在于政府作为。最后，南方系媒体控制舆论的手段真的很有一套 |
| 1. 热搜没有了，厉害 |
| 1. 说到底窝里斗的还是太闲了，事情都已经出了，还能怎样呢，政府自有安排，过好自己再说吧，要喷干嘛不喷美国 |
| 1. 各位防疫人员很辛苦，我们人民群众很心疼。但是，这一次华亭宾馆管理疏漏，是哪些领导拍板叫香港同胞过来而没做好后续的隔离准备工作和监督工作的，我觉得这点需要给民众一个交代，华亭宾馆，锦江集团需要负责。商铺，小工厂的经济损失，怎么办？有的人举全部家当创业，政府又该怎么解决？ |
| 1. 上海市六院还是临港六院？ |
| 1. 不能因噎废食，也不应以偏概全，现在上海需要每个在沪人士共同努力、共渡难关，也需要不在沪的大家的精神支持。不要实现人生价值时要上海海纳百川，有事就骂上海装逼，上海也会累，也会疼，也会难过。 |
| 1. 六院热搜下的挺快啊 |
| 1. 我爸爸还在六院求求保佑 |
| 1. 这次就好好接受网友的批评吧 |
| 1. 上海六院，能不能别敷衍了 |
| 1. 身为领导能不能发现问题及时想出方案解决问题，瞒报到底是处于一个什么心态，能不能将心比心，每个家庭都有老人跟小孩，第一时间发现的时候，紧急处理比什么都重要。每个地方都不缺缺心眼的领导，也不缺一心为民的领导，疫情真的是能照亮人心的一面镜子🪞 |
| 1. 上海发布你人呢 |
| 1. 热度降的真快 |
| 1. @冲绳最甜凤梨 |
| 1. 住在上海的🐶都有优越感 |
| 1. 这是在撤热搜啊？？？？ |
| 1. 疫情当下，各地方由上到下都应该团结抗疫，而且一方有难，八方支援，咱们泱泱大国，别说八方支援了，十八方都能来支援。为何会敢瞒报，为何要瞒报，先是吉林某高校，现在是上海某医院，纸包不住火这么简单的道理都不懂的吗？团结一致抗疫，疫情终将过去，加油！ |
| 1. 精准泄疫的沪人 |
| 1. 上海加油 |
| 1. 唉 |
| 1. 吉林呢？ |
| 1. 《精准防疫》《抄作业都不会抄吗？》 |
| 1. 那上海最开始的又是哪里跑回来的呢，北上广深是中国的门户，境外输入的压力都顶了几年了，咋滴就活该啊，出了事，都在怪上海，是不对是房价对调一下就没有这些阴阳怪气的声音了啊？ |
|  |
| 1. 这是怎么啦， |
| 1. 噢 |
| 1. 奥密克戎病毒传播力太强了，新冠病毒对所有人类社会都是挑战，这场攻防战要群策群力，打嘴仗就能赢吗？互相借鉴经验，有则改之，无则加勉，有问题就说问题，该落实责任就落实责任，我们后面的路还是摸着石头过河，现在互相攻击真没必要。 |
| 1. 要做内部调查 |
| 1. 疫情也要做好个人防护和配合 |
| 1. 什么国家都这样管控了，疫情还是如此反反复复 |
| 1. 医生隐瞒疫情，逼迫护士无防护工作，上海可真棒👍 |
| 1. 哪里都不保险 |
| 1. 注意防护 |
| 1. 上海太差了，再也不去上海！！！！！ |
|  |
| 1. 也就深圳狠，说封就封，是个狼人 |
| 1. 3年了，疫情怎么就遏制不了 |
| 1. 快用UVC病毒消杀技术 |
| 1. 满了 |
| 1. 咱就是说好好做防控，有些时候很难做到万无一失，咱就是管好自己，祈祷同胞们安稳度过，如何防控也不是我们网上敲键盘能做好的。 |
| 1. 冲 |
| 1. 都怕撤职，都怕担责 |
| 1. 这也破防咯 |
| 1. 愿太平无事 |
| 1. 太难了 |
| 1. 为了自己的仕途，领导不当人啊 |
| 1. 妈的，上海招谁惹谁了？各地疫情严重的时候，上海支援哪一次不是冲在前面？全国各地，哪里没有得到过上海的支持？上海有疫情居然还被骂？也行啊，受过上海好处还骂上海的，祝你倒霉一百年。 |
| 1. 啦啦啦 |
| 1. 六远的医生竟然打护士，我也是醉了，我以为是家属打护士，结果自己人打自己人，我也是醉了 |
| 1. 一定要防控好啊 |
| 1. 疫情赶快过去吧，还我们一个正常的生活环境 |
| 1. 防疫一盘散沙，各区各自为政，上海的没落看来不远了！ |
|  |
| 1. 笑死了评论里竟然还有阴谋论三个字几年过去了还论着那奏嘛呀 |
| 1. 我就很纳闷了，外溢的城市每天确诊那么多，上海每天新增没几个，那么多无症状感染者？ |
| 1. 在中国官方处理问题的特色：隐瞒 |
| 1. 谁再吹公立医院管理好啊？不也就这样？还打架呢？真是丢人！ |
| 1. 大家谁都别嘲讽谁，天道好轮回 |
| 1. 浙江也深受其害，还特么的不承认 |
|  |
| 1. 疫情应该很快会控制。但是六院的男医生竟然会打护士！这就不能忍了，他还是人吗？ |
|  |
| 1. 不夸大不隐瞒，做到一直很难吗？ |
| 1. 可以接香港的人过去 |
| 1. 哎，可恨的疫情 |
|  |
| 1. 整天不给市民做核酸，把疫情放给兄弟省市，不负责，什么精准防控，屁！ |
| 1. 上海为了保证给大家继续做ATM，在这样疫情环境下，宁愿停饭店都不给停工！动不动就停摆，我们也想啊，上面不愿意啊！再说了，你倒是接掉境外的飞机呀，别动不动就飞上海 |
| 1. 让网红出来走几步…… |
|  |
| 1. 我现在都还记得沪吹全网贬低其他城市防疫，吹嘘上海是精准防疫先进典范，并以上海不搞全员核酸检测为荣的模样 |
| 1. 请问动态清零和清零的区别？ |
| 1. 上海疫情北京头条来报 |
| 1. 烦死了 |
| 1. 啥时候居家办公？ |
|  |
| 1. 上海防疫已经信用破产，累计传到七八个省，每天只爆出几例确诊病例，周边城市纷纷对上海来的实施3+11新政，昆山直接封了去上海的路，可见真实情况……。天天吹精准防控，阴阳怪气踩别的城市，这次爆出这么多大漏洞，直到现在才要求出城48小时核酸，可见防疫责任感之差…… |
| 1. 为了祖国那一个逆子，所有兄弟姐们都遭殃 |
| 1. 所谓的一陪一护针对的老百姓，里面的清洁工都是横的 |
| 1. 又有了 |
| 1. 数据好假 |
| 1. @来去之间你可劲儿压热搜吧 |
|  |
| 1. 源头香港为什么还不管控 |
| 1. 都是中国人，疫情难道是他国的吗？为什么会是嘲笑，表示不理解 |
| 1. 呵呵 |
| 1. 加油，上海！ |
| 1. 加油加油！ |
| 1. 持续关注疫情，愿国泰民安 |
| 1. 西安被传染都不敢直说只能说省外还要被骂 |
| 1. 还是北京头条有态度 |
| 1. 某些上海人民真是玻璃心，谁闲的没事追着你们这种无名氏骂？不就是因为六院爆的一些事吗？真就娇生惯养只能夸上海好呗 |
| 1. 愿早日好。 |
| 1. 看看上海六院吧，不要装聋作哑了 |
| 1. 所以六院怎么了 |
|  |
| 1. 啊为什么不一样¡评论配图 |
| 1. 为什么上海无症状这么多，确诊这么少？ |
|  |
| 1. @空纸又严重了什么时候能见面 |
| 1. 没错 |
| 1. 天呐这这这 |
| 1. 说防疫做得好都怪上海外溢的城市？有的城市境外航班都没有凭什么骂上海，北京那么大的机场他们接多少，上海接多少。苏州防疫好，江苏一直封控快，那境外航班直接转弯去苏州吧，都是疫情的祸上海也在尽力 |
| 1. 香港不封内地全封 |
| 1. 不是笑上海，是说上海六院，不严查说不过去 |
| 1. 上海？不是吹精准防控中国第一吗？翻车了这是 |
| 1. 疫情快过去 |
|  |
| 1. 我感觉绝对不止这么少人，这么大一个城市，人流量这么复杂。怎么可能只有这么点呢？我没有诽谤的意思，只是怀疑。 |
| 1. 上海装逼，要不然也不会主动接收香港的同胞，接收倒没问题，酒店消毒不到位 |
| 1. 大家注意，踩和捧有很大一部分是同一批人 |
| 1. 不止吧.. |
| 1. 说一千，道一万上海疫情就是香港宗桑害的！就是香港宗桑把病毒带来上海的！香港宗桑滚回去！ |
| 1. 什么红楼地铁夹人孕妇六院，到底咋回事啊，一觉睡醒就这么多词 |
| 1. 奇了个大怪上海什么时候吹自己精准防疫，踩别的城市了？而且上海这次是因为华亭出了问题，华亭住了什么人不知道吗🤷🏻‍♀️怎么搞的上海恶意传播病毒一样如果没华亭那帮人上海今天也不会出事好吧 |
| 1. 怎么又严重了，疫情快点结束吧 |
| 1. 请查明上海六院疫情真实情况，保护好一线工作的医生护士们！ |
| 1. 加油中国 |
| 1. 这不是传染病啊 |
| 1. 国外输入不断！国内疫情就会断断续续的出现！ |
| 1. 关注了 |
| 1. 中国🇨🇳加油💪 |
| 1. 祈祷上海平安 |
| 1. 🙏🙏🙏🙏🙏🙏上海快快好起来呀 |
| 1. 早说了，抓起把沙子，手指送一送，漏下的沙子总得有人接着 |
| 1. 确定没有人投毒吗？ |
| 1. 66666666666666666666666666666666666666666666 |
| 1. 每个人都要做好防护 |
| 1. 啥时候是个头啊 |
| 1. 精准防疫原来是精准瞒报。 |
| 1. 人家北京广州深圳也没得瑟过。就上海是说不得娘娘 |
| 1. 怕 |
| 1. 疫情早日结束 |
| 1. 上海加油💪 |
| 1. 中国加油 |
|  |
| 1. 真的还有蠢人不知道这次上海是为了救香港才沦陷的吗？ |
| 1. 上海阴间新闻是真的多，上海孕妇，上海地铁，上海监管不力上海政治好差，人民好辛苦 |
| 1. 只求平安 |
| 1. 防控 |
| 1. 上海疫情 |
| 1. 大家不要理那些在评论区的阴阳怪气的人。这些人没安好心，在这里故意引战，挑拨是非，分裂祖国。不要让他们得逞 |
| 1. 不要被黑子带了节奏，所有城市的疫情都希望尽快控制。最近反中势力肯定会浑水摸鱼，希望无论哪个地方的人民都不要攻击自己的同胞 |
| 1. 上海精准防疫就是最大的笑话封了一个奶茶店一片叫好但是背后却是管控不力导致疫情外溢出了问题就是瞒报粉饰太平 |
| 1. 了解 |
| 1. 来自大城市的“危险” |
| 1. 藏着掖着只能消费我党的公信力，要赢得群众的信任才能战胜任任何困难 |
| 1. 压力山大，更须加油。 |
| 1. 从没看见过无症状报道是怎么回事 |
| 1. 就这还瞧不起外地人呢，本地人就先倒下！ |
| 1. 加油↖(^ω^)↗ |
| 1. 🙏加油 |
| 1. 我家小区被三个上海来的祸害了，又突然紧急核酸。 |
|  |
| 1. 现在看来网传都是真的，说不停课，结果两天后就停课了，说不封城，结果现在变相封城，还有哪些网传？ |
| 1. 不能 |
| 1. 精准 |
| 1. 可怕上海真可怕 |
| 1. 加油！加油 |
| 1. 国家抗疫一直不容易，上海这次就是某些人太自信了导致的，那些还在装x的说上海现在还是很松的，这种人怕真的🧠不正常要不是我也在上海，我真的信了 |
| 1. 沪舒宝最让人反感的就是我网暴你是为了你们好，你们网暴我，就是你们的不对！ |
| 1. 真搞笑，素质见高低，有些人真的唯恐天下不乱 |
| 1. HK来沪航班每天几十例确诊但却可以不熔断，谁拍板的？ |
|  |
|  |
| 1. 2022都不容易 |
| 1. 精准防疫真准 |
| 1. 怎么会这样，啥时候是个头 |
| 1. 西安几次疫情都和上海有关，这次也不例外。 |
| 1. 大家别骂了，希望全国都快点好起来。唉，可太烦了。 |
|  |
|  |
| 1. 我妹妹在上海，这下不知道什么时候才能回来，希望这波疫情早点结束吧，没完没了的 |
|  |
| 1. 悲剧 |
| 1. 挺好的，上海本来就超负荷了，入境航班赶紧分散到其他城市吧，比如防空很牛的西安 |
| 1. 你永远可以相信上海 |
| 1. 疫情防控要严防死守！ |
| 1. 早日安好 |
| 1. 加油吧 |
| 1. 坚持党的领导。 |
| 1. 愿世界无灾无难 |
| 1. 真厉害，这么快榜单上就没有了，咱家微博真厉害 |
|  |
| 1. 弄不好了，没完了，开学不开哪哪不能去，两年多了，搞死了 |
|  |
|  |
| 1. 这就是疫情一直拖了三年的原因！一直在遮掩，全国有很多像上海六院这样，只不过这次事态严重实在瞒不住了！ |
|  |
|  |
| 1. 上海还有民乐幼儿园感染了多名儿童呢 |
|  |
| 1. 问一句，无症状感染者和确诊的区别？ |
|  |
| 1. 曱甴真多 |
| 1. 这次香港祸害了不少地方啊！你们就惯着这个反骨仔吧！ |
| 1. 怎么没有人嘲笑上海呢？ |
| 1. 发布会有什么好看的呢，让我们老百姓花时间看他们撒谎。 |
| 1. 上海继续吹继续吹 |
| 1. 我们可以永远相信上海，我在武汉，我是抗疫志愿者，上海有脚踏实地的官，爱心满满的民，扎实的专家（张文宏为代表），为上海加油：以最小的代价，争取抗疫胜利 |
| 1. 昨天登记个上海回来的，我问他疫苗打了几针了，他说没打，我说为什么不打，他说不想打。。感觉上海确实心态好。。。可我心态不好啊 |
| 1. 病毒厉害 |
| 1. 现在为什么这么多的无症状啊 |
| 1. 不管怎么说，加油吧 |
| 1. 你们微博这些人真的好闲，除了冷嘲热讽当键盘侠对国家一点用都没有 |
| 1. 一向以防疫措施和应急处理反应最快为傲的上海，竟然以这种方式上热搜，希望尽快说明情况给大家一个安心 |
| 1. 西安比上海做得好太多了！西安迅速封城，没有造成像上海这么严重的外泄，更没有吹牛皮说自己精准防疫！23-28轮核酸1566万人！上海呢？牛皮都吹爆照样孕妇拒诊，孕妇流产、小红楼、地铁夹死人、宾馆医院大感染然后控评撤热搜！至少西安的信息是公开透明的！西安接受所有人的监督和批评！ |
|  |
| 1. 上海怎么还不要求居家办公？ |
|  |
| 1. 做好自我防护 |
|  |
|  |
| 1. 作为中国人大家这么落井下石真的好吗可以吐槽当地政府防疫不当为什么要吐槽这个城市上海一直作为标杆压力也很大航空熔断也不是上海人说了算的中国作为大国一直在包容国外疫情上海作为境外输入点也在不断接受新的挑战中国几个城市接收境外输入掰指头也数得出这种时候别再阴阳怪气了 |
| 1. 六院六院六院 |
|  |
| 1. 大上海快点清零 |
|  |
| 1. 加油祖国！ |
| 1. 打护士的医生傅一牧，本科毕业进上海三甲医院工作6年不断进央视纪录片澎湃新闻​……啧啧啧，好奇家庭背景 |
| 1. 严格管控 |
| 1. 怎么突然就多了 |
| 1. 无奈 |
| 1. 基本都是无症状了，病毒威力减轻了？ |
| 1. 上海真的是个装逼王。赶紧好好管管这届各路神仙领导吧。自己没做好还找顶锅的。这是高高在上的上海人的做法吗？ |
| 1. 上海人的排外在微博上算是体现了，一句让不得外人说 |
| 1. 可以强制办公嘛，公司让我们带核酸上班，公交地铁停了好了，大家减少流动！ |
| 1. 心累 |
| 1. 上海--干啥啥不行，甩锅黑别人第一名 |
| 1. 首先先学会别瞒报吧！ |
| 1. 网络就是键盘侠战斗的地方 |
| 1. 🙏🙏🙏 |
| 1. 这我是很不能理解上海嘲笑浙江全民检测？还有嘲笑北京？然后现在说话还要带西安这两字，是自己不行吗？人家怎么不说别的地方就说上海呢？也是要自身找找原因。还有说西安自己给自己买热搜停课和飞机我们很闲吗？在我们来说不过是正常的事情而已根本就没有必要还是先把你们防护措施做好再说 |
| 1. 遇到问题撤热搜，遇到困难就瞒报。看来哪里都一样 |
| 1. 为什么这么说上海 |
| 1. 早点结束 |
| 1. 上海真的没必要去喷，要不是上海愿意接国际航班，国外留学和务工人员回国就更加艰难，而且疫情本身就防不胜防，很难做到清零 |
|  |
| 1. 疫情二年了，管理还这么差 |
| 1. 我周围的上海市都在吐槽瞒报确实 |
| 1. 小心，1450及境外组织挑唆制造内陆地区间敌对心态，时局动荡一致对外，搁置争议。少些抱怨，那样没有任何用处，共同努力为国家巨轮增添一份动力能源，再小也是一份力。各自做好自己该做的事，赚该赚的钱。有大家，小家才会更幸福。 |
| 1. 现在已经开始拼“无症状”了吗？不确诊不担责？ |
| 1. 打脸总是太快。之前在网上高呼“抄上海作业，看怎么精准防控”的那一波人去哪里了？ |
| 1. 早日战胜 |
| 1. 在有些人眼里，“上海”两个字就是原罪。不知道是谁嘲笑了外地，确定是上海人吗？如果是，那个上海人可以代表我们所有人吗？这次的疫情不管是天灾还是人祸，总之都是上海的错。 |
| 1. 这次好几省疫情又是上海输入，长点心吧，谎言会戳破的，非得要到为全国人民低头认错的时候，搞实验田利己主义折腾外省 |
| 1. 差 |
| 1. 境外输入不是闭环管理吗？之前还有人科普境外的都是统一隔离核酸检测，是不会传播到社会面的，那这次是哪个环节出了问题啊？ |
| 1. 就觉得如果是真的为什么要隐瞒下来呀，我们又不是“外人”，不怕你生什么病，只怕你有“病”不说，为什么呀，我们不是一家人，不能一起解决吗？ |
| 1. 不是护着上海，咱也不是上海人，防疫这一块做的真没得挑 |
| 1. 不要再给外省输出了，几个省就被你们这种装b的政策霍霍了，还在吹牛逼 |
| 1. 上海加油！中国加油！我们一起扛~ |
| 1. 上海之前因为精准防控麻痹大意了，导致疫情外溢江苏陕西等省份，好好的认错不行吗？非得找回面子？什么境外航班多接的人多隔离任务重。那广州，北京，深圳，四川等等地方也有境外航班啊，广州白云国际机场还是一度成为全球第一多航班的机场呢，深圳每天都要接几百个香港过境的呢，那是不是要吹破天？ |
| 1. #上海六院疫情##上海六院疫情##上海疫情##上海交大疫情#城市一换，评论过千万（西安：又来黑我？） |
| 1. 骗子 |
| 1. 我不理解你批评一个人批判一件事都很合理但有些人否定一个城市是想干嘛？想搞分裂吗？ |
| 1. @央视财经@光明日报@人民日报@人民网@央视新闻@央视网可以调查一下关于#上海市第六人民医院#这个事情，如果是真的，可以管控吗？ |
| 1. 瞒报太多了我们小区也确诊了附近的也封了很多天，到现在都没有黄浦 |
| 1. 上海什么时候嘲笑过别人了，一些骂上海的是受过什么挫折了？这么多疫情城市就怼着上海骂是因为上海是明星城市吗😊😊😊可以理解，明星都是要被人说三道四的 |
| 1. 魔都魔鬼多。 |
| 1. 也就是今天一共新增61 |
| 1. 吵勾8⃣️吵了疫情能好吗？不管南方北方都有自己做的好与不好的地方嘴下积德一起度过难关不好吗？被微博搞地域黑的人带着跑冷静点吗？ |
| 1. 表彰都领导受着，这时候倒没影了，推一帮护士上，给真正的好医生丢脸！这么多护士和病人家属的命不是命吗？！危害这么多生命免职都不足以解决！打人的医生和杀人的未遂的区别在哪里？！ |
| 1. / |
| 1. 我觉得喷这次上海防疫失控也能接受，确实做得不好，但我怎么也想不通做了两天多志愿者，总共没睡几个钟头，临睡了还要被嘲笑，被嘲笑到睡不着。敲键盘地域黑的人哪里都有，但是精准防控的概念本身是没错的吧？对老百姓影响尽量小点不好吗？ |
| 1. 无语 |
| 1. 此病毒不是上海制造，上海承担了大部分的境外帮那些没能力承担的省市包揽了业务好来人无完人一旦出事那些没有能力一直躲在背后外省人心里千百个恨啊你怎么没守住门你怎么还传给我们了你是怎么做的你们上海太没能力了你知道吗就是因为你们的过失让我们不能过像以前躲在你背后的安稳日子了 |
| 1. sh？卧槽 |
| 1. 上海控制的挺好的，希望继续加强严控，加油！ |
| 1. 看不下去了，我就问一件没有这些一线工作人员，谁去检测核酸，防控新冠，医治病人，是你们穿着防护服的领导吗？！ |
| 1. 为了保证精准防疫，脸都不要了 |
| 1. 大上海人的优越感从来没有缺过 |
| 1. 内讧有意思吗？心理太阴暗！ |
| 1. 就说外溢了这么多个地方，而且我相信不止我们这儿在重点排查有上海旅居史的人了。就这样还压。前两天官方通报还说就几个新增，你们这一波节奏着实让人民不相信zf了。🙈 |
| 1. 这次上海的无症状太多了吧…… |
| 1. 总计不就是61例吗，为啥非要把无症状分开说，显得好看嘛。。。。。 |
| 1. 为啥全国只有上海无症状比例这么高呀 |
| 1. 疫情这么严重，各位键盘侠除了会地域黑，又能为疫情做点什么呢 |
| 1. 说的我很赞成，但是话说回来主要还是前面沪吹把上海吹上天了把别的城市骂成狗屎了，所以现在算是反噬，过了就好了 |
| 1. 我一直有问题为什么到现在所有的国际航班还是不给停，现在深圳东莞全部封闭造成的损失比国际航班停运损失大的多得多吧，国际航班关三月，会少什么嘛，因私的国外的别回来，国内的别出去不行吗。。。 |
| 1. 咋说呢，反正各地都有疫情了，摆烂呗，都待家里。 |
| 1. 总感觉这样的政策下去，国内会开始内讧啊这是国外最希望看到的大家要清醒啊唉 |
| 1. 我党好好想想失去群众、让群众寒心的结果是什么就该明白现在到底该怎么做了吧！ |
| 1. 山东这么严重都没上热搜 |
| 1. 这里评论还没被删 |
| 1. 这次公关做得不好，热搜榜都好几个小时了，差评 |
| 1. 都是中国的城市，干嘛互相说不好啊，大家一起相互扶持就可以了呀，现在上海这么严重，应该关心而不是落井下石。 |
| 1. 真的只有55例么？ |
| 1. 上海某些小赤佬：没把控好不准你们说，六院的事不是上海人，医院也不是上海的，用的只是上海的第，就是不准你们喷的意思，金贵的很 |
| 1. 这倒是该说一下，毕竟这个疫情源头是万恶的那啥弄的。咱们这个时候互喷没有意义。还是希望大家能共渡难关。这个时候都不好受。咱们这儿2年多的无新增被上海这波外溢给整破防了。而且，我们也给足了上海面子，报到都是写省外输入，而没有指明是上海。青春那么短，疫情就三年。三年了啥时候是个头啊。 |
| 1. 上海人是真的不行 |
| 1. 捂不住了吧 |
|  |
| 1. 护佑平安 |
| 1. 阳性病例是在临港院区发现的吗？ |
| 1. 法律法规现在明确规定，地域黑网暴都会被被查ip和行政拘留👋，绩点口德吧评论区无脑喷上海的 |
| 1. 行了吧…………都是自己人，都是中国人，本来境外输入就防不胜防的，我们不是更应该团结一条心吗，再说现在变异株体本来就很狡猾，干嘛非要骂来骂去的，有意思吗……… |
| 1. 丧嗨宁说不得 |
| 1. 虚报瞒报 |
| 1. 这两天某书上的上海街拍几乎就没有看到戴口罩的…… |
| 1. 66 |
| 1. 上海人biss |
| 1. 数字对吗？我同事周一碰到确诊的，周五才通知他隔离？4天老早跑好多地方了 |
| 1. 没有指责的意思，但是这一次我们这里两年多的无新增被上海整破防了。不管怎样，都是同胞，都希望全国各地的疫情都快点结束。 |
| 1. 我看大街上没一个戴口罩的 |
|  |
| 1. 上海学学深圳吧 |
| 1. 上海加油！！！ |
| 1. 希望疫情快点好🙏 |
| 1. 阴阳怪气讲话的人到底是在看上海笑话还是在帮助上海人反思，动机是什么？敌我不分，帮西方国家拆自家团结，唉 |
| 1. 三年的疫情了还是这种处理方式只能说一直原地踏步或者说又要五年一波了 |
| 1. 不是太明白确诊和无症状感染者的区别 |
| 1. 疫情防控是大家的事，请不要网络暴力，一味埋怨是没用的。我怀疑那些骂的人都是间谍吧被收买了，来煽风点火来了，想让中国社会动荡。 |
| 1. 怎么上不去呢？ |
| 1. 看完吉林林的再看完上海的我觉得还行啊上海那么大 |
| 1. 听说上海是因为接收乡港人，所以爆发了，是不是真的？ |
| 1. 不可信 |
| 1. 无语子 |
| 1. 下台把 |
|  |
| 1. 上海承担的已经太多太不容易了，此刻只希望赶紧太平 |
| 1. 上海～一个鄙视全员核算，只要不测就没有的城市 |
| 1. q'吉林吉林 |
| 1. 看了上海那个什么电子厂的视频，感觉没有我们广州安逸哦，之前感觉大城市防控有力，希望这次不要让大家失望哦，要是一直爆爆爆群体免疫可能在路上了 |
| 1. 希望疫情赶紧过去!!!上海挺住!!!还有就是别压自己个儿热搜了，也别买别的地方的热搜了!!大家心里都很清楚的啊 |
| 1. 身在上海从没受到疫情影响，为上海的精准防控点赞👍也相信上海很快走出这次疫情。 |
| 1. #上海六院情况说明# |
| 1. 唉热搜一半和疫情有关不是这发现就是那听课 |
| 1. 兄弟，你这数据太假了，都已经都曝出来了。你这样消费政府的公信力。真的没道理的 |
| 1. 目前被隔离在上海松江某小区，第三天了也没有任何物资，饿的不行。关键也不说啥时候解封，居委会也不发公告，就说等通知，向单位请假都不认可，对上海防疫彻底失望了。 |
| 1. 过一会江苏各个市喜提热搜嗷 |
| 1. 认真 |
| 1. 无症状也是确诊病例吧，能不能别说的这么含蓄其他城市很多被隔的人，确诊后也都是无症状者，都是一视同仁啊，为什么上海要这么报？ |
| 1. 六院真丢上海人的脸！！ |
| 1. 人类对抗病毒哪能做到精准，非要搞政绩做宣传，拿市民的健康开玩笑，这场战役就是宁可错杀一百不能放过一个，拿经济换健康，当初二三十平米的地方，好好消毒几个小时都能给病毒冲几个澡了，非要封几天拍点照片做点宣传精准防控，真搞笑 |
| 1. 我就好奇，这个数据通报有第三方监督不 |
| 1. 这精准地区防控远没有网络舆情做的好。 |
| 1. 疫情期间去了几趟上海公共交通畅通无阻地铁火车站没人检查基本的绿码行程码。今天疫情的反复一点不惊讶。 |
| 1. 说实话，现在真的没必要清零了，因为估计这个病毒会伴随人类好几年，应该想想其他方法，现在天天让人排队做核酸有点矫枉过正的感觉。 |
| 1. 哟！ |
| 1. 上海金山检测异常隔离点已经满员了，里面都是检测异常但没有确诊的病人，他们缺少足够的医生，希望这批未确诊的病人能得到国家有关部门的帮助 |
| 1. 看了看言论，喷上海的或说被上海喷的，微博都没发言地域显示的，所以到底是哪里人说的话谁知道呢，干脆强制要求微博显示发言区域就太平了。 |
| 1. 看了评论区，围西救上？ |
| 1. 等什么时候你们的谩骂、诋毁能转变成为对疫情有益的行动，我想中国的思想、素质教育应该不低于其他强国了 |
| 1. 评论里的上海人真是让我开了眼界了 |
| 1. 天津人搬着板凳坐这看。 |
| 1. 上海做的不好还不让人说了 |
| 1. 首先发现有阳性患者应该立马转移到专业医院去，该封院封院，该隔离的隔离，所有省份都是这样的，没有什么好瞒的，若是自己医院有隔离病房那就有专业隔离病房工作的医护人员，让一些没有培训的人直接上（重点还是密接）我不理解这个医院的作为，太不把自己的护士当人了吧，看到这里我真的是很气愤 |
|  |
| 1. 防不胜防 |
| 1. 评论区真是…都2022年了还有人在yygq地酸上海啊格局大点吧 |
| 1. 上海的疫情什么情况我就这么说吧第一次看见家附近的小区封掉了我家在去地铁站一定要做公交车的乡下 |
| 1. 数据是不是要捂不住了 |
| 1. k |
| 1. 上海六院上海六院上海六院上海六院别的不想说，就几分钟全删评了，再过几分钟热搜都降没了。 |
| 1. 致敬医务工作者 |
| 1. 真心搞不懂你们现在这些人，你们能算是人吗，一个省爆发，你们就互相喷互相嘲笑，几万条人命在你们看来很好笑吗，国难当头不仅不互相加油鼓励，一天到晚互喷，真不想骂你们畜牲 |
| 1. 加油啊 |
| 1. 评论里好多键盘侠 |
| 1. 这个上海政府和香港政府有得一拼 |
| 1. 希望六院快点好起来，昨天做着核酸，今天就关门了🙊🙊🙊 |
| 1. 上海一直疫情严重，就是不知咋地那么安静一整年都有疫情 |
|  |
| 1. 上海宣传系统我是服气的 |
| 1. 大家要团结一致去抗疫，上海加油，中国加油 |
| 1. 为什么突然就遍地开花了？ |
| 1. 全国都疫情爆发，希望疫情快点过去 |
| 1. 热搜继续啊，别压 |
| 1. 期待一个真实有效的回应 |
| 1. 上海六院上热搜了！ |
| 1. 防疫魔幻事件：1、所有去过风险地的人都要去社区居委会报备，你来我往欢聚一堂2、黄码红码人群不能去任何公共场所，却可以自由出入社区居委会还是防控办要求的3、所有确诊病例的行迹无一例外都没有提大社区居委会，是他们都没去过报备？还是故意不说？这些到底是精准还是懒政？ |
| 1. 仔细看看?数据和实际真的匹配么? |
| 1. 上海六院领导层能判刑吗 |
| 1. 六院领导咋说不是传染病哦？ |
| 1. 好好查查6院吧，一个医生竟然说新冠不是传染病，自己穿的防护服不上楼，让只带了口罩的护士照顾阳性病人，这就是精准防控的上海 |
| 1. 好好查查6院吧，一个医生竟然说新冠不是传染病，自己穿的防护服不上楼，让只带了口罩的护士照顾阳性病人，这就是精准防控的上海 |
| 1. 西安疫情就是上海传来的。哎！刚消停一个月过了年，又来 |
| 1. 一个区失守，一个医院又失守了，混乱堪比武汉疫情初期。全国防疫，本就没有神话 |
| 1. 开始降热搜了 |
| 1. 上海是全国人民的上海，这次的疫情从全国的传播速度来看都是非常迅速的，大家都有些掉以轻心，上海已经在积极应对，没必要搞地域针对，你在说风凉话的同时，想想如果经济重地的崩溃，对国内其他地区的形势都是不利的，如果想自己城市好，就盼着上海早点控制局势吧 |
| 1. 上海加油啊，控制住，疫情当下人员流动好麻烦 |
| 1. 相信上海，在广州为上海加油 |
| 1. 不是精准防控吗精准到哪里啦 |
| 1. 我就不明白疫情报备为啥一定要去社区居委会不能在网络平台？人人都去社区居委会这不是更大的交叉感染风险吗？最魔幻的是黄码红码人群不能去任何公共场所甚至医院都不能进，去可以自由出入社区居委会还是防控部门要求的，这到底是精准防控还是只是防控懒政？魔幻魔幻 |
| 1. 看评论，真是搞笑死，说什么小浙江，什么别的地方犄角旮旯……要上天么这是，个别上海人真是这嘴吐不出象牙，别给自己抹黑了 |
| 1. 321 |
| 1. 本人判断疫情估计与春节长假有关我所在的公司节后有大量的员工感冒姑且不论是普通流感还是新冠希望科学家能早日开发出抗新冠特效药把新冠当感冒治从而结束疫情 |
| 1. 其实无症状也是阳性，也是具有传染性的啊，这样区别报道，有什么用呢 |
| 1. 其实百姓心里早就有定数了，冬奥开完，疫情猛增，只不过没想道，增的这么严重 |
| 1. 领导：听我说，这不是传染病！而自己全身上下装备齐全！可笑！ |
| 1. 我不装还不行嘛！我撂挑子了，你们这13个城市自己玩去吧¡查看图片//@请结合临床及其他检查:你是不怕境外输入毕竟境外的飞机都往上海落//@红社会组织:球球了，上海以后别装逼了。现在不怕国外输入，就怕上海不认真输出到省外啊 |
| 1. 六院的具体情况什么时候能披露！！！医生打护士！！院领导穿着防护服告诉护士这不是传染病！！这些事到底是真是假，能不能解释清楚！！！ |
| 1. 这个时候还想拿人家西安给自己顶雷，贱不贱啊，真下作真小人！！！！​ |
| 1. 这轮疫情来势汹汹，大家的神经每天都绷得紧紧的。按理说出了问题就该及时上报并找方法解决，可是总有个别人为了保住自己乌纱帽，瞒报甚至强压基层工作人员执行不合理的工作。唉~希望其他地区高层引以为戒，把抗疫放在首位吧！中国加油！ |
| 1. 没事都打了疫苗呵呵 |
| 1. 哦吼，好一个精准防控 |
| 1. 感觉这一次上海有点难，希望疫情赶快结束，这样的日子真的过够了 |
| 1. 我想知道六医院事件什么时候给个答复 |
|  |
| 1. 怎么可能这么少 |
| 1. 热度慢慢在降... |
| 1. 上海第十南京第四 |
| 1. 领导辛苦了¡评论配图 |
| 1. 一觉醒来小区封了，外卖停，快递停，一点准备都没有，三天了，核酸结果到现在都查不到，也不说什么时候解封，好乱，还有一颗白菜… |
| 1. 上海六院不出来解释吗 |
| 1. 中国人还是老样子，窝里横，出国连个屁都不敢放。挺好的，今天骂上海，就像当初骂武汉一样。同胞这个词算了吧。不同阶级的人在一个国家也不是同个世界。 |
| 1. 问责严惩 |
| 1. 每次报道都是上海疫情精准防控，看到网上那么多视频流出，现实估计也挺乱的。 |
| 1. 此处艾特央视@央视新闻 |
| 1. @梅川酷子67你注意啊 |
| 1. 上海疫情，没有瞒报漏报吗？我周围很多朋友的小区都有确诊都封了小区，可从来没在上海发布上出现过名字！请问确诊的人去了哪里？另外，为什么要大家在公司就地隔离？这样不危险吗？上周新闻发布会每天就不停报数字，什么措施都没有，也不停课！那么多孩子感染！！ |
| 1. 傅一牧是怎么回事？ |
| 1. 不管怎么说，打人者该抓抓。院部门领导该撤就撤 |
| 1. 六院领导是干嘛的、自己穿防护服不冲在一线缩在后面，一群护士没有防护服不把他们当人，这种领导立马下台 |
| 1. 《热门》 |
| 1. 几时才结束 |
| 1. 评论真是惊呆了，上海乱套了，别的地方有什么好处吗？国人自己互相地域 |
| 1. 我们小区跟没事一样，没封也没让做核酸，不测体温不看码都随便出入睡觉了明天还要上班呢 |
| 1. 我们小区封了，居委和民警都不让拍照！懂了么？ |
| 1. 还是相信政府，至少我所在的区都有条有序，大家都很辛苦，放弃了休息日坚守在岗位上，谁都有疏忽的时候，尽力弥补吧 |
| 1. 开任务分配（摊派）动员大会，干活高效办事靠谱的即使不吭声，领导的金手指也是会不偏不倚的呢//@CoNnIe-yyyst:我一直不懂那么多人追着骂到底是为了啥？是需要2500w人给你们下跪吗//@红社会组织:球球了，上海以后别装逼了。现在不怕国外输入，就怕上海不认真输出到省外啊 |
| 1. 上海口碑约等于台北的口碑之于台湾吧，天龙人，自视甚高，却极端崇洋媚外，好多人有外国国籍绿卡，看不上外地省市。要不然怎么北京大妈炫耀八旗通天纹，上海大妈炫耀我是美国人。 |
| 1. 现在疫情还有死亡病例吗？ |
| 1. 热搜就这么掉到第十了！ |
| 1. 关注一下绥芬河吧，都是命啊 |
| 1. 感谢北京头条的关键词 |
| 1. 这能压下来，真是对大家的不尊重啊刚刚还第八现在第十 |
| 1. 多关心一下医务人员吧。一年到头一直在抗疫，到了新冠病房做六休一。最基本的生活物资都没有。要自己带，吃的也要自己带，八个小时工作制有时候甚至忙到八个小时不停歇，根本没时间出来换口罩。上面下令医生非必要不进病房，全是护士冲在前线，这份工作真的寒心 |
| 1. 在北京发布下，看上海，西安，浙江等等城市pk |
| 1. 敢情只有新疆在认认真真地防疫 |
| 1. 评论真有意思，黑什么的都有，这时候不是应该越团结吗？我们不是一个整体吗？现在评论撕成这样，都不要造谣就够别人笑话了 |
| 1. 现在喷上海的必有陕西人！ |
| 1. @小桃子警官 |
| 1. 到底担心的是什么，这个所谓的精准防控，是否把百姓放在第一位？明明身边有疫情，隐瞒不报。公司也只是发一纸通告，注释告诫我们上班人注意防护，少乘坐地铁公交。难道我们没车的要走路上班么？有时候也要为我们打工人多考虑呀，不要一味的想着经济发展 |
| 1. 医护人员辛苦了！精准防控不能停留在嘴上 |
| 1. 拿出骂西安，吉林的气势，接着手撕上海！全国骂遍了，你就是人生赢家了 |
| 1. 在大都市时间呆得长了就以为自己水平就是大都市的水平了，见得钱多了以为自己也是有钱人了 |
| 1. 好多人看笑话啊 |
| 1. 上海加油💪//@神之近卫军:21年，上海承接14137架次国际航班，占入境总量35.6%。今年前两个月1972架次占41.2%。一线、隔离酒店、公卫中心满负荷运转，这样的日子，上海过了三年，上海疏忽了、也累了，但我们坚信他会历劫归来，而所有失去的，都会以另一种方式归来 |
| 1. 有些人就恨不得上海出事，一看到上海两个字就开骂开喷！ |
| 1. @留的一点芸香在 |
| 1. 回复@凯溡三:全网最佳！ |
| 1. 想问问具体的阳性人员信息 |
| 1. 听说6院的护士都没防护领导硬让上 |
| 1. 我们小区防疫人员都说是有确诊封小区的但是通报上每天都没有。也不知道是怎么回事 |
| 1. 上海人吃什么？都是无症状 |
| 1. 还记得那个15㎡的奶茶店，上海特色的精准防控 |
| 1. 全国投毒 |
| 1. 在深圳的我们也很难，同样理解上海深圳这样的一线城市防疫难度，面临的挑战太多，太难了 |
| 1. 谁会想要疫情发生的，都是一个国家，大家还是努力共同抗疫吧 |
| 1. 这也能搞地域歧视？有些人的脑子病得好像得过800次新冠一样，脑子萎缩成黄鱼了？//@凯溡三:一个个只会键盘喷，窝里横，就这样子，都不用外媒动手就已经可以内斗了。上海有点情况都跳出来嘲讽，幸灾乐祸，脑子里请有点命运共同体意识，上海如果真崩了，下一个就是你家门口。一帮阿缺西 |
| 1. 我只想活着…… |
| 1. 各位上海的同胞们你们去理睬这些地域喷子干嘛大家做好自己的事情又不是我们的错该配合的配合防疫没事少在外面乱逛增加防疫负担到时候防疫不力的该担责的担责六院管理真的有问题该处理的处理 |
| 1. 上海真的也很不容易，我们大家加油抗过这场疫情 |
| 1. 一场疫情，让我们明白，活在这个世上，除了生死，其他都是小事；除了健康，一切都是浮云。好好的活着，就是幸福 |
| 1. 有钱就是好，买热搜祸害别人 |
| 1. 据说医生和护士都干架了吧 |
| 1. 上海加油🙏🙏 |
| 1. 热搜压下来了厉害啊 |
| 1. 长三角地区所有境外基本都是上海一人扛了下来，不知道有什么资格说上海，哪个有能力的城市别bb你行你上啊。要多感恩，互相扶持。 |
| 1. 就这么点，貌似没多严重啊 |
| 1. 境外势力真可怕，分裂社会的手段真是一套一套的 |
|  |
| 1. 上海真是做色孽，承接各种境外输入还要被左边的江边样子哈讲扒讲//@红社会组织:球球了，上海以后别装逼了。现在不怕国外输入，就怕上海不认真输出到省外啊 |
| 1. 就问能居家办公吗迟迟不通知真的怕了不能我闭麦 |
| 1. 只求居家办公 |
| 1. 呼呼 |
| 1. //@红社会组织:球球了，上海以后别装逼了。现在不怕国外输入，就怕上海不认真输出到省外啊 |
| 1. 看着评论，突然能理解抗日时候伪军那么多是有道理的 |
| 1. 加油💪 |
| 1. 能不能封城，打工人明天还得去公司@上海发布 |
| 1. 上海土著觉得这次真的很严重但喷子哪哪都有冲浪不必较真都是中国人何必在哪说风凉话呢 |
| 1. 楼里别吵了，还不如去顶贴让大家重视起来 |
| 1. 还是深圳的领导雷厉风行，66例马上停运公交地铁，非常有前瞻性 |
| 1. 到底是不是传染病呢？那位领导？ |
| 1. 瘟兵犯汉宫，经冬又历春。精准护毒宝，几度害西京。 |
| 1. 热度不要下去啊 |
| 1. 深圳今晚宣布已经封城，上海也同样被香港所累，为什么香港就不能封关，你们自己爱怎么样确诊是你们的事，为什么来祸害我们，上海和深圳，多么重要的两个城市，就这样栽了 |
| 1. 差点就信了，别骗老百姓了 |
| 1. 我觉得上海防疫整体做的确实好一点，但是大家喷可能还有一部分原因是上海部分人太傲娇了多多少少有点瞧不起别的省，才引来众怒(别喷，喷就是你对) |
| 1. 上海这效率，这态度。青岛，吉林，深圳都控制了，上海都控制不了！ |
| 1. 我只能说上海舆情控制一级棒，从迪士尼开始，外省接受上海输入病例通通不敢吭声只能说是外省不能说是上海。大儿子有爸爸罩着别的孩子都不敢吱声。 |
| 1. 评论删的也不少啊 |
| 1. 真想想了解一下上海到底有多少小区街道单位实行14天封闭管理的….中风险地区只划分那些个没人居住的地方有什么用？既然小区街道单位不是中风险地区那为啥要封闭管理呢？就很不理解 |
| 1. 现在上微博就是有人评论下面就有人骂哎 |
| 1. 🙏🙏🙏天呐 |
| 1. 数据做的是真漂亮啊 |
| 1. 小区居民只要做了核酸就可以自由进出，外卖一概不能进小区，出租车快递车可以进，什么鬼逻辑 |
| 1. 唉，什么时候是个头啊 |
| 1. 为什么有的已经被确诊了这几天网上也不见公布，更不见确诊者行踪 |
| 1. 这是真实数字吗 |
| 1. 祈祷台湾再次大停电，让微博安静一会 |
| 1. @六神驱蚊花露水6688评论里在说还有假数据？ |
| 1. 上海怎么了。求官方说法 |
| 1. 又不是谩骂东北的时候了，深圳上海管控不当咋没人知声呢，真是风水轮流转啊，国人都互相伤害吧，都去看笑话吧，都居家做个键盘侠埋头开骂吧！埋怨完这个吐槽下一个，活该！再出言不逊网络暴力地域黑的人，希望国家出手法律制裁！ |
| 1. 表面工程做那么好，有什么用。压制舆论，瞒报，算什么 |
| 1. 上海啥时候也没嘲笑过其他地方啊真的逗做得好就是好不好就是不好上海每天接收来自全国各地和全世界各地多少游客你们有谱儿吗，这回一个不小心爆发了你们就这么迫不及待显示自己自卑的优越感吗？ |
| 1. 上海这波疫情可给有些人逮住了，真有意思 |
| 1. 疫情稳定的时候，大家都大意了…放任香港等地自以为是… |
| 1. 你去提议让其他地方都封了某城别给进不就好了 |
| 1. 没有全局考量，科学变成了伪科学！ |
| 1. 香港有只鸟进入广州，瞬间广东鸟都成惊弓之鸟！ |
| 1. 上海从疫情解封初期就承担着全国唯一入境筛查压力，就算后来其他城市也逐步开放了，上海还是承受着过半的入境人流。这样不搞精准防控难道还次次都全市封控？那么多无症状感染者哪个城市能提前查出来？ |
| 1. 如果去年听了某医生的建议，在结合香港的现状……预计到现在死亡至少几百万，“放开”没错，但要看时机，但绝不是医生提到的去年的时候…… |
| 1. 香港疫情目前为止数据显示，死亡率和其他国家相比名列前茅…这数据意味着什么？？死亡率为什么比别国高？如果放开，以香港死亡率计算，计算过吗？中西方抗疫不同点在于：中国希望每一人都活着；而西方“与病毒共存”是野蛮的自然淘汰制。 |
| 1. 有些武断了。每个城市地理位置，经济发展，人口数量，医疗资源都不相同。全国各地不因地制宜，采取一刀切是以牺牲经济和生活为代价的。一次两次可以，次数多了谁也受不了。上海本身防疫压力就大，病毒在改变。只要有脑子都不能去照搬别的城市，还得因地制宜防疫才行。 |
| 1. 以体质论实在说，中国人体质比美国要差，据最近数据，中国人75%亚健康（抵抗力差），放开？如果去年放开，那是关乎民族危亡的事情……大都市偷摸的搞，那是建立在国家零感染防控基础之上的…才没出大事，即使这样还是影响了全国……有些人要负责任的… |
| 1. 放开，国家早就想，但要看死亡率……香港的“与病毒同在”，目前750万人口，每天死亡近300…… |
| 1. 我们上海市民自己也很希望跟你们一样管控方式，我们现在不敢上班还是不得不上班，每天都在跑毒！！ |
| 1. 这么久都没事，作为上海人，非常肯定上海的防疫！ |
| 1. 让香港的都飞你们那些地方吧，让上海喘口气 |
| 1. 全国尤其大城市恐怕真的是只有上海没做全员核酸了，周围扩散出去的外省市有一例就都做全员核酸了....上海这所谓的精准真的是蜜汁无语，公司附近基本被阳性圈包围了，街道也没让做，公司也不做...... |
| 1. 上海精准防控那么久都没事。是因为香港三百人来隔离。空调没有装过滤循环。病毒外泄的。 |
| 1. 上海花了多少防疫成本！肩负了多少境外航班，居然如此内涵上海！奥密克隆本来就传染力度大。本身上海这种人口密集城市就不能，也不该担负这么大的境外输入！！！ |
| 1. 经典地域攻击言论不看全局，只挑有矛盾的点说事，这种不客观的言论大家看看就拉倒吧 |
| 1. [福气虎][福气虎][福气虎] |
| 1. 面對疫情，做好防護 |
| 1. 中午快到了，午餐愉快 |
| 1. 晚安好夢💤🌙 |
| 1. 支持博主觀點 |
| 1. 你去点名首都啊，首都也是精准防控，只会说上海 |
| 1. 上海的精准防控比我去的绝大多数都好好吧张口就来 |
| 1. 价值几百万的防控和价值几十万的防控，政策怎么一样呢？你出钱？还是你把全国文化经济一致化呢？ |
| 1. 起码某地人去外省自觉的做核酸传染完了拍拍屁股走人裂了真的 |
| 1. 上海也是一个香人外溢来的，他不是直接飞来上海的，是先进了别的地方，然后在来上海，没有隔离到处走造成的 |
| 1. 坐标上海。我认同上海的防疫政策。你可以建议你所在的城市封闭起来，鸟都不进。 |
| 1. 关键在于那个地方作为全国金融中心不能直接封，代价太大，而且封了也不一定有用，人流量太大等确诊了密接早就全国跑了 |
| 1. 让香港飞机飞你那去，让境外飞机都飞你那去，我们上海还就太平了! |
| 1. 疫情每天全球都有爆发的可能，这个原因不是你一个键盘侠了解的，上海也没有完美，但如果，连上海都新增，那你们那里爆发后将不堪设想。 |
| 1. 精准防控才是上海经济外贸高度发达地区的正确选择，动辄停摆封城对经济和正常生活会带来毁灭性打击 |
| 1. @X车厘子小丸子倩倩子Q |
| 1. 说上海害了这个地方害了那个地方的键盘侠，跪求你们去和上头提，让你们觉得能接手这么多境外输入的人员，那么多隔离人员的城市或者省来接。不否认上海是做的不好，反正啥事儿都骂上海，上海就跟亲妈生后妈带似的。嗷嗷被说这说那，说上海歧视外地人，说上海害了八个城，说上海崇洋媚外，上海欠你们了？ |
| 1. 精准防控没有错，但是适时地调整也是必要的，不能认为精控适用每一种场景，躺在功劳簿上不动了。国际大都市的担当和能力，要拿出来！加油！我们可以的！ |
| 1. 一定要做好防控啊！做好居家隔离 |
| 1. 当时划的有多小，有多精准，现在就有多尴尬 |
| 1. 某些人站着说话不腰疼，天天夹枪带棒带节奏，有意思吗？ |
| 1. 都这么严重了还不封控停课，都外溢多个省份了。 |
| 1. 直接投诉这种博眼球的吧 |
| 1. 你又知道上海不检测了？蠢还是坏啊 |
| 1. 微博上各种V的格局和素质实在太低 |
| 1. 相信上海 |
| 1. 上海的精准防控屡赌屡输？现实更加证明了，其他的二三线城市的无差别防控，除了给老百姓添麻烦之外，对疫情也没有起到什么作用。优等生一次考差了，一堆差生过来落井下石，可笑。//@大风吹奏:所谓的精准防控近乎于赌一把，还屡赌屡输。 |
| 1. 你是官媒吗？这样说话的是想咋样🙏 |
| 1. 啊对对对，正面消息酸上海，负面消息黑上海，反驳了就说我们上海高贵说不得，太典了，差不多得了 |
| 1. 我记得上次我说上海一直没有全民检测很危险，下面还一群妖魔鬼怪说上海多么牛逼我们多么不懂呢！ |
| 1. 学好自己的专业、做好自己的本职工作多么重要。“以高调开始，常以低调收场”，现在怎么不高谈阔论了？ |
| 1. 不宜这么说，在总体规范的前提下的探索，在一定情况下也取得一些成效。当然还需要更努力，更加实事求是，科学态度最重要，当然对于上海昨天指出的谣言惑众，信口雌黄，瞎忽悠，胡咧咧的不学无术的不规范，如同阿飞一样就会胡扯的要批评。//@大风吹奏:所谓的精准防控近乎于赌一把，还屡赌屡输。 |
| 1. 回复@一片海的愿望:医院和各个机构都开着，可以自行去检测啊 |
| 1. 这也太可爱了吧 |
| 1. 希望疫情赶紧过去吧 |
| 1. 不要祸害四方 |
| 1. 快扎紧你的篱笆，把门锁死，别出来，也别让其他的生物进去，那一定安安稳稳的。 |
| 1. 上海人表示对上海领导班子很失望！追责吧！ |
| 1. 上海实际上很多地方都已经在全员核酸了，只是没有集中性报道而已。对于上海五条独立传播链的疫情，遍地开花式的传播，就算有再强的防控管理能力也会散拨在一定范围内。至少目前社会面确诊极少，基本都在传播链上管控了，对于一个城市管理来说，即做到范围的精控，还要尽可能减少对市民的影响确实不容易 |
| 1. 群体免疫现在看起来还是有点道理的 |
| 1. 沪卫兵都赶到了，小心点 |
| 1. 图片评论¡评论配图 |
| 1. 各地发展情况和执政能力不一样，一样的防控政策和力度付出的代价也不一样，各城市定位也不一样，在当今的国内外环境中需要多种平衡，不是能够简单一概而论的，所有统一的，如果那样子，那么就需要放弃很多东西。 |
| 1. 试点放松清零政策不是好事么 |
| 1. 上海这一波还没有因奥密克戎死的，但我相信封闭小区搞一刀切，肯定有人会因急病不能就诊死的。 |
| 1. 全员核酸不可能的，2020年新冠最疯狂的时候都没实行全员核酸 |
| 1. 现在恐慌才是最可怕的 |
| 1. 同意政策防控需要一致，但也需要考虑民情经济和目前的疫苗普及状况，三年了病毒都进化几轮了还按照以前的一样的方式走，真的还可以吗 |
| 1. 我觉得上海的精准防控很好，只封该封的，不封不该封的 |
| 1. 我再次建议你去了解上海精准防疫的政策和举措，在保证城市高效运转的背后，是无数防疫工作人员在精准防控，岁月静好是有人在替你负重前行，很多城市的防疫是平时疏忽，出事封这个封那个，相比于西方确实是尽职尽责，但是在花费大量精力在平时的上海，这就是一种懒政。 |
| 1. 上海的防疫工作你可以去了解一下，在没有疫情的时候有多少工作人员把防疫工作做在平时，才做到不仅达到精准防控，而且保证学生可以正常上学，成年人可以正常工作，这次的疫情，防疫部门已经公布是因为一个管理疏忽导致的。上海在很长一段时间的精准防疫的成效是显而易见的。 |
| 1. 不现实，不说上海没这么多人手实现全城防控，每天的花销也得以亿做单位 |
| 1. 上海人非常肯定上海的防疫 |
| 1. 这次上海是管理疏漏引发的，跟精准防控有啥关系？ |
| 1. 兄弟，你不是上海的吧 |
| 1. 为了一个逆子把自己搭进去 |
| 1. 全国各地防控政策一致，那要不要境外航班也都往全国各地飞不要只飞上海广州等城市，这些城市承受着多大的压力，你真的是站着说话不腰疼得了便宜还卖乖 |
| 1. 为什么不是全国向这两个城市看齐？ |
| 1. 香港什么时候与病毒共存了？乱说 |
| 1. 短视者，你就不想想都一刀切封经济民生能承受？你去养活那些低收入者？ |
| 1. 精准防控做得挺好的啊，上海这么大的城市不可能动不动就全员核酸吧 |
| 1. 没办法一致的，还有国外呢，国外没法和我们统一，以后总要打开国门， |
| 1. 精准防控到底怎么样你感受过吗真的张口就来上海接收境没人夸一出事就来内涵还某地方…..马后炮这种话少说 |
| 1. 我认识一个香港来的孩子，我说你来上海，别乱跑。他说巴不得能得新冠。还说新冠也阻止不了他出去玩。没法沟通。 |
| 1. 瞎说什么东西不检测就知道阳了吗用眼睛看的吗 |
| 1. 上海别的不知道怎么样，甩锅水平全国第一名。六院出事，外地患者住院阳性。感染多人，大头都是无症状，确诊是个零头。难道恰巧外溢其他地区的都正好是确诊？ |
| 1. 哪有空着的离岛，把上海和香港一起打包送去相亲相爱完事，两地挺互有好感。只要不闹独立，随他们精准防控装逼去 |
| 1. 我记得不举是轻后遗症来着 |
| 1. 丫天天装逼精准防控，结果外溢把好多城市都害了 |
| 1. 不会有事的上海和美国一样发达 |
| 1. 难怪娃读书内卷那么厉害，防疫也卷 |
| 1. 让境外输入飞你们那边呗，上海的防疫政策和力度科学精准，国内没有哪个城市更好了吧 |
| 1. 某市的不检测精准防疫政策，为国内多地爆发贡献了病原，这属于疫情防控不力吗？ |
| 1. 不懂不要瞎说 |
| 1. 上海从头到现在承担了多少境外你们看不到上海遭难了你们如此得意咋滴了中国人欺负中国人啊有意思吗我们不慌魔都加油 |
| 1. 上海防疫水平没毛病，发现问题及时有效能控制 |
| 1. 为什么现在还有人嘲笑精准防控了。为什么连上海都有黑子在网络批皮上海人吹上海。上次和这次性质不一样。上海这次都全员核酸了！医护这么累，志愿者那么辛苦，蟑螂躲在网络背后嘲笑有意义么？ |
| 1. 看到有人喷你，我就放心了[冰墩墩] |
| 1. 在上海的人都庆幸自己在上海，没有精神压力的管控，其他地方都给老百姓巨大精神压力制造恐惧感，愚蠢 |
| 1. 你又知道不检测了，基本所有企业每周一次，有的两次，不知道不要大放厥词 |
| 1. 死要面子的沪城河 |
| 1. 你除了当喷子还能做什么，妖言惑众！奥密克戎这个病根本没法，高福也说了当时的预判失误，以为打了疫苗可以全民免疫屏障！现在我们要意识，她的共存，改变防疫策略 |
| 1. 很多国家都开始和病毒共存，我们如果不死防，就实行共存政策，但是，不实行也不严防的话，浪费的就是大白们的牺牲。 |
| 1. 这次疫情过后我们上海再也不接收输入性新冠源体，外国回来的各回各家各找各妈，谢谢你们 |
| 1. 你的名字就取对了，来上海的出上海的都自费隔离21+21天，大家就都太平了。 |
| 1. 一国两制……政策制度问题，做不到的，大陆也没法完全管理香港 |
| 1. 这波疫情本质是香港政府不作为，防疫政策与大陆政策背道而驰导致的，凭什么上海人要背锅，别的省市疫情爆发的时候上海人说什么了吗 |
| 1. 要不把国际航班都赚到北京去？看看你还骂不骂？ |
| 1. 放开估计是最后的选择 |
| 1. 小心你号没了有的人为了洗白，连航班号和籍贯都改了 |
| 1. 注意安全，注意防护 |
| 1. 全国各地防控政策必须一致啊。 |
| 1. 唉，希望疫情早点儿结束吧！ |
| 1. 真心的希望所有人都能够远离疫情 |
| 1. 好可怕不会传播吧 |
| 1. 疫情赶紧过去吧 |
| 1. 太难了吧希望疫情可以早点结束 |
| 1. 香港这是管不了吗？以后湾湾更难。。 |
| 1. 希望你们不要来上海 |
| 1. 香港🇭🇰一定有内鬼不作为想搞乱香港拖中国大腿！务必谨防！！！ |
| 1. 把香港看成和其他共存的外国一样就可以了，香港是传染源，但不是唯一的传染源。 |
| 1. 那些想与病毒共存的都是已经感染的吧？与病毒共存的后果就是人口减少，经济下滑 |
| 1. 墙倒众人推被你诠释的淋漓尽致 |
| 1. 全民同心共通防疫不好吗？非要站在上帝视角阴阳怪气的去职责，是不是心理有问题？与其蹲在电脑前无脑喷，还不如为自己城市疫情防疫做出点绵薄之力！如果实在控制不住自己的言行，建议可以看看心理医生！ |
| 1. 为攻破中国防疫，东原乐多少水军，看下面评论就知道了 |
| 1. 张网红医生内心向往美国新加坡抗疫模式，必然对中国模式有抵触，抗疫工作懈怠 |
| 1. 说实话，上海承担了很多。如果可以的话，那种本地有三甲医院和隔离医院的地区帮上海分流一点就好了。像成都那些的话，其实压力也挺大的…… |
| 1. 建议你的当地政府启用熔断机制OK，全部自给自足，相信就没有疫情影响了 |
| 1. 你以为为政者不比你聪明？ |
| 1. 一看中医粉，就懂了。 |
| 1. 远在上千公里外的我因为受到上来疫情精准防控的快准狠的防疫政策的影响，此刻正在居家隔离呢14天，工作都不能做就整个一离谱！ |
| 1. 上海全员检测一次比较好 |
| 1. 支持老铁 |
| 1. 在苏州站进出站都要查验48小时核酸的时候，多次出入上海站，却从未查验任何内容，单从上海市市民个人体验来说，防疫确实相较其它很多城市没有那么严格。原以为是精准防控下的自信，但前几日太仓通勤的病例确实证明上海疫情外溢，希望上海能够提升大城市担当。实事求是。 |
| 1. 无脑跟风 |
| 1. 我最讨厌这种人，有本事你去和国家申请把浦东机场承接对外输入人员管控的任务拿到你们城市去，看你能做到什么程度？ |
| 1. 关键是香港继续这么随意永远都弄不好啊然后这边一直要收 |
| 1. 傻子 |
| 1. 中国2022年这波疫情就是香港人传开来的。害深圳年前核酸不停，封禁不断，深圳就是不肯把深港口岸给停了，禁止香港人入境！ |
| 1. 太可笑了，其他地方全员核酸封城，就是为了保卫上海北京这样的经济政治中心尽量过正常生活。你本末倒置，不理解国家政策和全局。 |
| 1. 上海两年多的精准防控，取得的成绩有目共睹，风平浪静时漠视上海，上海防疫工作者一直在默默付出，从未松懈。目前的确出了问题，大家也没有想着帮助上海，而是用文字抨击上海，文字可以抗疫？不如去当地做做志愿者大白，为疫情真正的做出自己的贡献，我们相信上海防疫部门~会还我们安全的生活环境 |
| 1. 港府豁免机组人员免隔离的时候市民专家都是反对的，有用吗，弄出个大窟窿出来港府现在就装死，破罐子破摔了，如果这群尸位素餐的官员中央不问责就搞笑了。 |
| 1. 现在非得把确诊和无症状分开算，无症状一样是被确诊阳性有传播能力，然后统计里基本都只写确诊总人数，不写总无症状数，都jb搞不清到底有多少人真得新冠了 |
| 1. 建议核平香港 |
| 1. 你为啥觉得自己那么懂，去应聘一下疾控主任吧 |
| 1. 阴阳怪气你是什么防疫专家吗 |
| 1. 不检测？？你臆想的 |
| 1. 太不负责任了🙏 |
| 1. 这次决定接收大批香港人的决定的人应该出来负责人 |
| 1. 可是马上要中考高考了，要真上网课，我们这届就完了 |
| 1. 今天公布的五例和21例无症状还没计入 |
| 1. 2020年的表吗？ |
| 1. 为什么自告奋勇要分流香港居民？出事了吧？ |
| 1. 适当做一做压力测试也行不能永远死守 |
| 1. 奇怪，这回突然上海严重了 |
| 1. 周六还要聚众教资😅 |
| 1. 继续上班上到确诊 |
| 1. 疫情到现在已经不能像以前一样死守了，无症状越来越多就是征兆，是时候尝试放开了，而且中央已经在提倡精准防控，这就是给放开的信号了。 |
| 1. 哈哈作为过去三年生活在广州的表示真的没啥去年12月底刚来上海，小区就封封楼两次，有一次还是我住的楼，只能说之前上海防的太好了一些人接受不了这种落差吧，该干嘛干嘛呀，服从管理 |
| 1. 周六的教资....又是一拨.... |
| 1. 市教委还在硬撑什么啊我不理解 |
| 1. 楼主看发布会了吗这些病例都是在集中管控时期发现的不能只看数字不看实际情况啊… |
| 1. 我不是杠哦，因为各种观点，甚至截然相反，停课歇业又有另一批人骂防疫过度 |
| 1. 与病毒共存，群体免疫是最终的选择，清零政策在没有“疫苗”出来之前都是苦最下面的人… |
| 1. 闵行已经停课两天了。谁说没停课啊 |
| 1. 一哥你好red今天 |
| 1. 青浦一例都没报就离谱根本不知道哪里有 |
| 1. 实话，如果现在再全部居家，那可能，国内经济问题更大了 |
| 1. 2020年是封城了，你要封城吗？ |
| 1. 为了所谓的GDP，为了政绩！ |
| 1. 因为国外都开放了这是才测试国内疫情抗压力，以后肯定与疫情同存 |
| 1. 不仅不停，现在直接连周末都没有了 |
| 1. 有没有人管管松江大学城朵拉大学城 |
| 1. 呵呵，上海的思维可不会那么落后，学会逐渐共存，上海一定时走在其他城市的前列，现在还在坚持清零的，早晚会被时代所淘汰 |
| 1. 就不懂为啥。我们已经被有确诊感染的封闭小区包围了，孩子们还正常上学的 |
| 1. 上海根本就没有打算严防死守哦，想太多 |
| 1. 崇明是净土 |
| 1. 漏了，青浦多的是 |
| 1. 命到底重要吗？是不是要整个上海所有人都确诊了才肯停课居家办公？打了疫苗有什么用？该中的还是中，不该中的也中了，这是在拿老百姓的命在开玩笑。这样下去上海早晚沦陷 |
| 1. 我们公司已经要求明天开始在家办公，暂时先到3月底 |
| 1. 想问考研复试还能正常进行吗？ |
| 1. 虹口这个，压根没有报道 |
| 1. 居家办公？公司让我们准备牙膏牙刷毛巾去公司办公，我真是操了他的🐎了 |
| 1. 我也不知道为撒学校都不停课却要求线下培训全不停课？可真牛逼 |
| 1. 最近在忙着为两hui删微博，没空办正事儿 |
| 1. 闵行区好多难怪周边医院学校都封了。 |
| 1. 为了能继续吹，上海速度，精准防控 |
| 1. 真的无语，附近小区就有几个都封了，隔壁小区全员核酸，就是不停课停工，疫情刚开始的时候都没这样过 |
| 1. 感觉是个放开的试点，不然干嘛不停工停学 |
| 1. 继续上班刚做完核酸周六聚众考教资 |
| 1. 学校门口就有个确诊的小区。。明天还要继续上学。。出了门都怕回不去。。😭 |
| 1. 真的不知道在想什么普通百姓的命就不是命了就离谱 |
| 1. 应该到别的城市就被查出来的的也算上 |
| 1. 不知道哪些煞比瞎传造谣的。一边不相信政府，一边又相信公布的数字？？ |
| 1. 从公司有确诊爆出到现在30➕h过去了从昨晚就在说的第一轮核酸安排到现在还没给安排上大几百号人被封在楼里、基本都是密接、次密接想问这样真的安全吗？还有、这就是所谓的上海速度、精准防疫么？30+h过去了啊！！！O网页链接 |
| 1. 整得到处是密接，次密接，就上下班的功夫，就接了 |
| 1. 精准防控么。上海不一直是好学生交作业大家跟着抄 |
| 1. 呵呵，上海真是个好地方，不敢说，说了就有护城宝出来喷 |
| 1. 浦东惠南的教育机构出现确诊！！教育机构不是明令禁止了吗？还出现这样的失误？彻查！！ |
| 1. 我们学校停课了 |
| 1. 还不停课。上到确诊也不停课真是大城市啊 |
| 1. 我在摆烂上海也在摆烂 |
| 1. 没事的，热搜能压就压，压不住等过了这个风头，领导买两条宣传热搜就好了，依旧还是国际大都市 |
| 1. 最可笑的是很多公司非但不让居家办公，还要求三天做一次核酸，凭证上班，这是怕员工去不了人群密度大的地方交叉感染么？…… |
| 1. 我是真怕，家里有小孩，哪里也不敢去 |
| 1. 6月要中考家被封了学校上课一分钟都不能少啊现在不让我上课了能不能都别上真的对我公平吗我要上高中的啊我要听课的啊别人可以上课我就只能在家坐牢，再不停课等封更多感染更多吗 |
| 1. 我们明天幼儿园师生一起核酸检测了 |
| 1. 学校被包围了 |
| 1. 绝了，天天在家都还是觉得不安全，门都不敢出 |
| 1. 闵行我真的会谢 |
| 1. 是不是在试点 |
| 1. 快点让本地大学生回家网课吧我真的求求了学校明明没有任何人确诊但就是吊着不上网课 |
| 1. 上海都是些来镀金的领导、弄的上来越来越不像样、就知道数据好看 |
| 1. 我不敢发截图，教育局发布的通知：关注学生的出勤率，出勤率低的个别沟通 |
| 1. 崇明还没有是吗 |
| 1. 我们公司倒是周日就通知居家办公了。这波接收香港人算是坑苦了上海 |
| 1. 明天会更新不？加上3.10的估计要超400了 |
| 1. 去年静安寺奶茶铺爆出疫情的时候就和同事说了，上海真叫没搞全员核酸检测，要搞的话感觉会有很多，现在不幸，被我言中 |
| 1. #上海疫情发布会#闵行居然名列前茅 |
| 1. 今天早上4+76，晚上又5+21… |
| 1. 为什么我开始怀疑这个数字…（现在很严重，那时候大爆发为什么只有349……单纯疑惑……别骂我） |
| 1. 真的烦！香港的滚哇 |
| 1. 321例是现在的？ |
| 1. 这个病毒这辈子肯定结束不了了，还是学会和它共存吧 |
| 1. 真无语了还不停课有病吧 |
| 1. 全军覆没. |
| 1. 不能松懈，带好口罩！ |
| 1. @月光灼桐屿_天呢上海在想啥 |
| 1. 听专家的做好自己 |
| 1. 周六周日还要继续上课，连上十一天的课 |
| 1. 我们刚才还在讨论这个问题，第一次的时候感觉真的没几个，这次这么严重居然还不居家 |
| 1. 停了，只是部分，我就是停的一个 |
| 1. 嘉定人多的我都害怕 |
| 1. 救救 |
| 1. 上海这几年挡了这么多境外，看来这回是真的累了～。大白们辛苦了！ |
| 1. 你搞笑吧！只是你那地方没停而已，这就要检讨了，领导你来当，我现在住老师家蹭吃蹭喝就等着回去呢 |
| 1. 因为产能刚提上来吧，不舍得停摆，停摆太影响经济了 |
| 1. 回复@人生是一个茶几:安徽浙江江苏都有回沪的感染病例了上海还是一点点的挤也是神了外地人跑上海一趟回来就有了所以上海到底是个什么情况了？ |
| 1. 兢兢业业守护了上海两年兢兢业业，出一次事情就被骂，唉，上海政府真难 |
| 1. 因为是奥密克戎了，要开放的前奏了。 |
| 1. 能不能居家办公现在完全看老板了 |
| 1. 我拒绝。 |
| 1. 说的很对 |
| 1. 到现在还不居家办公就很离谱了。。 |
| 1. 去崇明岛吧 |
| 1. 瞎搞精准 |
| 1. 是的呀烦死了明天还要上班都不敢坐公共交通 |
| 1. 为什么上海没有人被免职？ |
| 1. 停课了啊！ |
| 1. 很多单位已经在家办公了 |
| 1. 上海确实不严，正常下了高铁就该核酸检测。然后疫苗也是，我加强针都打了大半年了，上海的朋友还不知道去打。个人感觉各方面都很宽容 |
| 1. 放心上海发布，已经通知网课了 |
| 1. 上层建筑你不懂… |
| 1. 回复@SHERRY_yunyun的表态:看上海教育发布新通知 |
| 1. 毕竟还要接受香港过来的呢香港才是国家儿子 |
| 1. 一个政府如果不是为自己人民而只是想做出政绩来，那么早晚会失去市民的心！ |
| 1. 就是就是 |
| 1. @希望永远睡懒觉😅😅😅😅😅 |
| 1. 你这条居然没被压下去 |
| 1. 虽然感恩感谢医护人员们🙏但是也请真实面对这次上海突发的疫情，请求上面不要再瞒报了！拜托🙏现在哪哪都是人心惶惶的 |
| 1. 呦呦！怎么觉得你有点幸灾乐祸呢？ |
| 1. 脑子里都是💩 |
| 1. 上海不愿意承认精准防疫失败。 |
| 1. 疫情最厉害的时候都没这样，坐标闵行，昨晚突然小区就被封了，说是普通筛查，却要14天，也没爆出有病例 |
| 1. 现在官方发布都是留言精选，一片和谐，两年前可都是接受群众的批评的 |
| 1. 格局打开看问题的角度自然不同 |
| 1. 还有就是既然有的地方做全员检测，也不好好配套做，乱糟糟的 |
| 1. 事实就是以后这种情况就是常态，不可能让城市停摆的，国外做群体免疫情决定也不是全无道理，再基于过去两年的抗疫经验，上面肯定觉得目前还是在可控之中的，咱们配合就是了 |
| 1. 你什么都懂，去抗疫啊 |
| 1. 是为了钱吗 |
| 1. 上海这次一塌糊涂，管控地方，120车也没，进了医院，出不了医院，没有120接送，不可以自己走，一天了大家都回不去，上海到底有多少人在叫120，简直是想骂人了 |
| 1. 因为不敢，一旦停工停学等于打自己脸。而且现在是什么时候，在干什么 |
| 1. 生病事小，饿死事大 |
| 1. 同感 |
| 1. Gdp5.5的目标 |
| 1. 是需要好好检讨 |
| 1. 看看这次撤职哪位 |
| 1. 坐标上海闵行一小区，昨天开始要封14天，他封小区我要疯了 |
| 1. 说句你们都不信的话，我们厂到昨天为止进门都不需要测体温的 |
| 1. 到处在封，既防不了扩散，又折损了经济，邻居上班也是跑客户，防不胜防。呼吁封城。 |
| 1. 这次明显厉害多了 |
| 1. 上海真的被坑惨了，境外输入隔离酒店根本不够住，人家不管接着硬压指标，很多酒店打不到隔离要求，很多人员还不具备足够的防疫素质 |
| 1. 小区里隔壁楼都被封了还得正常去上班，只能说心是真的大 |
| 1. 身边很多朋友的学校都有同学家长确诊 |
| 1. 这波疫情真的影响挺大的关键学校居然不像以前一样停课搞的那么多学校需要检测还是带着家人一起的 |
| 1. 我靠我靠我靠我又不敢睡了 |
| 1. 我们公司都发内裤了，意思是工作到确诊，然后老外本周五还要去开40个人的派对。牛批，顶风作案！服了！ |
| 1. 海纳百垢去他的吧 |
| 1. 其实上海的防疫工作全靠上海老百姓怕死的心态，防护自觉的不得了 |
| 1. 不明白为什么香港那么多严重还要开放航班出来？ |
| 1. 我不信西安来两个上海的都30个了上海怎么可能这么少 |
| 1. 跟香港情况还是不一样吧，这里没有出现大量重症挤兑资源啊，不筛查都不知道附近小区有隐形感染者。 |
| 1. 确实很精准，遍地开花罢了 |
| 1. 教资都头铁还要考 |
| 1. 深圳高校一个都没有还都封校了，教职工全部居家办公 |
| 1. 害人 |
| 1. 公司通知明天居家办公，但是办公室正常开放，我们领导特意强调：要居家办公的跟他打招呼，这意思就是不让居家办公了吧，我没理解错的话 |
| 1. 20年上网课，中考高考结束上海多少学生跳楼？！希望领导们珍惜孩子们的命！ |
| 1. 这就是防控精准到人，不影响别人的生活啊，有问题吗？只要没被通知，该干嘛干嘛 |
| 1. 之前全名打疫苗搞了鸡飞狗跳！现在学校又要强制孩子做核酸了！tm卫健委几人开发布会没有一个带口罩的。要求人家不要聚众。出门带口罩。自己都没有以身作则。 |
| 1. 过几天学校要核酸检测还不停课不过中考快来了不好好学不行呀 |
| 1. 几十万餐饮、娱乐、旅游服务行业就业人员怎么办？都奄奄一息了，还能封城不成，涉及百万家庭 |
| 1. 上海会精准定位防护，可以控制管控区在几十平米內，大家不要慌 |
| 1. 真的太可怕了我们学校都是被叫回家的同学还不听课而且在学校也不戴口罩甚至就在前一天早操时候校领导和大家说下楼做操时不用戴口罩太闷了 |
| 1. 无语了还你妈不停课 |
| 1. 如果重复感染的几率很低的话，还是有序开放共存吧。给感染过新冠的通行证，可以出入自由，随意跨省出国。 |
| 1. 上海真的头铁 |
| 1. 抗疫都抗魔怔了，动不动就封城，动不动就管制，寸步难移，这样的日子没完没了了吗？逐步放开不行?非得天天关禁闭? |
| 1. 这样的格式很一目了然呀，以后发布能不能用表格的形式 |
| 1. 精准防控不行了？ |
| 1. 不停课反而通知我们上班时随身带被褥折叠床洗漱用品去他妈的宁可抱着集中隔离的决心也不停课头铁的哟吐了 |
| 1. 公司都倒闭了上哪打工 |
| 1. 正常上班，不过大家都把口罩戴起来呀，在公司也应该带。外面来访公司谈话也都没带口罩 |
| 1. 求求让小学生在家上网课好吗 |
| 1. 我上海客户说了居家隔离成本太高了 |
| 1. 看到的还是被压过的 |
| 1. 对于学校通知带好洗漱用品，保暖用品和睡袋这个骚操作我不理解。把老师就在学校镇压病毒吗？熊孩子怕老师病毒又不怕 |
| 1. 已经完全不是一种病毒了。 |
| 1. 有没有可能病毒可控 |
| 1. 别站着说话不腰疼了，作为家长、不想要停课，想停课的可以直接请假在家空中课堂 |
| 1. 把官方平台骂到N次方的，肯定忘不了//@水笔小欣:要政绩拍马屁啊，2020年疫情刚开始骂扑天雕骂到封词条都忘记了？//@人生是一个茶几:这次决定接收大批香港人的决定的人应该出来负责人 |
| 1. 好可怕 |
| 1. 这么多了救命🆘 |
| 1. 怎么说呢，停工停产，社会咋办， |
| 1. 已经有不少小学停课了 |
| 1. #上海疫情#请问小区隔离了，没有任何方案可以让人出去打狂犬疫苗吗？狂犬疫苗的致死率可是100%，上报就告诉你会继续向上报备，要报到什么时候？已经超过打针时间了 |
| 1. 就这松江欢乐谷，上海迪士尼还开着呢 |
| 1. 管的最严的时候只有20年头两个月而已 |
| 1. 上海被网上自己发点新闻夸懵了吧 |
| 1. 到底在坚持什么啊我不想戴口罩线下上课在路边吃食堂打的饭因为食堂不让堂食一进学校就担心下一秒被封所有活动都停了还坚持线下上课图啥啊我 |
| 1. 很简单这样是对正常的生活和工作影响最小的哪里有问题封哪里也可能是未来的趋势。 |
| 1. 现在是实验放开的前奏吧 |
| 1. 键盘侠抗疫第一 |
| 1. 真要没了 |
| 1. 这是高传播性的奥密克戎，不是2020年的老株 |
| 1. 哎～怎么肥事儿呀～ |
| 1. 全国疫情看上海感觉我们这次风向标是要和病毒共存的样子毕竟奥密克戎干预速度比不上传播速度清0政策不适合这代病毒了主要是清零后一旦恢复运作疫情又来了上海感觉在走一个新方向但是中央没开口地方也不能自己做主。看吧！ |
| 1. 上海稳住啊 |
| 1. 停课可以居家办公是打工人的狂想曲只有老板面临压力居家了可能就裁员了还办啥工 |
| 1. 我们早就居家办公了 |
| 1. 搞得到处都是了，我们这也有了 |
| 1. 精准防控厉害了 |
| 1. 建议上海市长下台 |
| 1. 我觉得很好啊，没有影响生活不是蛮好。 |
| 1. 群体免疫吧累了生死由命吧 |
| 1. 那些当初吐槽深圳表扬上海的人，知道深圳人有多难了吧 |
| 1. 青岛也很严重 |
| 1. 在家办公我们一家子就得饿死，唉，这疫情真的快点过去吧 |
| 1. 继续上学上到确诊三公里内八九个小区有病例周围所有小学初中都停课就我们高中天天跑毒决赛圈 |
| 1. 可能在下盘大的吧 |
| 1. 相信政府 |
| 1. 上海其实也是压着没有爆出来实际上也是有很多例 |
| 1. 上班上到确诊封城前的西安如出一辙 |
| 1. 也是没办法在规则下为了活着 |
| 1. 实际数量谁知道 |
| 1. 不过呢，2020年初当时都没打过疫苗，是应该停摆一切的！现在3针打完了，再这样封城的话，就要质疑……到底有效吗？纯属个人看法 |
| 1. 娱乐场所今天停了，电影院从明天起也停了。 |
| 1. 居家办公就无法压榨打工人了，就不存在内卷行为 |
|  |
| 1. 我在嘉定继续搬砖恐慌中搬砖 |
| 1. 我是大为震撼啊 |
| 1. 两全马上要结束了…… |
| 1. 应该是奥密克戎传播性很高的一方面因素，然后用经验来看新问题没重视吧？ |
| 1. 检讨什么？检讨你为什么没病么？ |
|  |
| 1. 请不要给国外反对势力递刀子，相信政府。谢谢 |
| 1. 我买的夏目友人帐展是不是只能退了到四月十号 |
| 1. 没打疫苗的孕妇真的害怕 |
| 1. 为什么要停课？ |
| 1. 青岛一千多了，说不定还更多 |
| 1. 民不聊生 |
|  |
| 1. 明天早上发布还会翻倍，崩溃！！！！！ |
| 1. 一直信任上海 |
| 1. 毒圈跑酷哈哈哈哈，我爱上班 |
| 1. 上海市民应该委托人大代表向市政府问责… |
| 1. 精准防控，当然停课！ |
| 1. 号对我来说不重要，有停Ke的但不让发，你猜他是要里子还是要表子…上海加油 |
|  |
| 1. 我今天才入职啊 |
| 1. 精准防控执行得很不错，说到做到，为啥要检讨。病毒感染危害流感化和国际社会逐步开放的大背景下，防疫只是生活的一部分而非全部，有疫就防无疫该干嘛干嘛才是正常的生活节奏。 |
| 1. 是的，因为上海来了个人到南京，南京多了3例确诊病例 |
| 1. 看了吉林农科，上海学校还不采取点措施！！ |
| 1. 感觉逐渐要跟病毒共存了，不如以前管得严了 |
| 1. 然后在上网课的我，下周要去外地上学了 |
| 1. 20年5km内有病例都全体紧张，目前大家就只希望不要被隔离在公司 |
| 1. 这表不对吧，宝山3.8号没有这么多呀 |
| 1. 那要快看看吉林，一天一百五，把你半年指标达到了， |
| 1. 我知道很多学校因为有密接，次密接都停了，学生和同住人都要做很多次核酸，我觉得上海防疫做的很到位了，能留在外面的会有多少是真的危险的？？？ |
| 1. 累死一线医护，一线居委，一线保安，公安人员，封这封那，就是不停学。不停工可以，为啥不停学，真是看不懂了。今天这个学校闭环，明天那个学校闭环，折腾小学生，折腾家长，折腾老师。哪个白痴领导出的政策，真是火大… |
| 1. 我们苏州算是挺过来了，但是都是正常上班的 |
| 1. 无语了 |
| 1. 上海大学 |
| 1. 按照这个趋势，明天无症状感染者破百了 |
| 1. 学校不停课，那就只能等着孩子直接被封在学校里了 |
| 1. 虽然你说的有道理，但是有一说一无症状应该是不算在内的……这种连中风险和带星都不会。 |
| 1. 要命了……… |
| 1. 附近5km就有疫情，还有人不戴口罩，就是因为现在大家都松懈了，才会爆发这么快。 |
| 1. 真的无语了昨天今天还在全校核酸已经有高中的老师确诊了就不停课。真·坚持上学上到确诊 |
| 1. 有道理～每天送娃，然后上班，提心吊胆 |
| 1. 可能因为这次致死率低了，不按照20年那样对待？ |
| 1. 8号越洋广场临时封闭跑出去很多人第二天又要召回但并不是所有人回去写字楼接受隔离不服从疫情管控不接受核酸检测的大有人在也并没有看到防疫相关部门有什么管控措施防疫形势大于实际意义 |
| 1. 上海这次很严重么？上个月因为工作跟同事一起去了趟，最近才发现带星了居然。。。 |
| 1. 这就是精准防控 |
| 1. 每天数据麻烦更新一下 |
| 1. 不知道在想啥 |
|  |
| 1. 难道是探索新模式？ |
| 1. 设立风险区上海说第一没有省份敢说话积极的很其他防控政策就没得堵死 |
| 1. @培根芝士双层牛肉堡 |
| 1. 可以停课了 |
| 1. 还没停课🙏 |
| 1. 上热搜让上海政府看看呀 |
| 1. 终于有人说实话了 |
| 1. 🙏🏻🙏🏻🙏🏻 |
| 1. 你这表做的比卫健委疾控中心发布会清晰明了多了 |
| 1. 能不能彻底封了香港人入境，嚯嚯广东还不够，又去嚯嚯上海，也是服了 |
| 1. 真的搞不懂，这么严重为什么不停工停学，害的别人现在喷上海精准防控，上海人民也瑟瑟发抖好吧，上面不把我们当人，下面老百姓还排挤我们，我们的苦谁懂，继续上班上学吧，上到无症状上到确诊 |
| 1. 传染力不是一个级别的 |
| 1. 话说松江大学城这边都开始封校了 |
| 1. @C阿君呐我累了 |
| 1. 今天已经发布全面网课了 |
| 1. 今天发布的要听课了 |
| 1. 同事小孩，包括上大学的都回家上网课了…… |
| 1. 检讨啥？再来摘乌纱帽？一刀切？疫情这种情况是谁愿意的么？大家都在努力 |
| 1. 我们学生都被拉去当志愿者了真的好痛苦 |
| 1. 这个才字，过分了 |
| 1. 可能在搞压力测试，总有一天要放开 |
| 1. 唉，为了居家办公这事，看透了领导的虚伪 |
| 1. 慢慢放开吧，别折腾了 |
| 1. 想开点，我感觉这是在慢慢适应开放，听说这次这个病毒只是和流感差不多 |
| 1. 这次是真的厉害了，前年疫情爆发的时候我们这里郊区都没有的，现在叫我们这里那么偏僻的地方都有病例了 |
| 1. 你不去吆喝禁止外出，就盯着学生不放，你们大人都消停点，学生也不会被连累 |
| 1. 其实是以前运气好，就蜜汁自信的当自己是优等生，然而现在奥密克戎太凶悍，以前那套玩法不灵了 |
| 1. 病毒传染性不一样，有可比性么 |
| 1. 因为都已经2022年了呀，出现病例就停课停学，那老师不疯，家长都快疯了。生活应该慢慢走向正常化，而不是倒退 |
| 1. 真他妈。。。能不能不要影响上海人民生活啊。。。工厂又得关。。。真就乌鱼子。。。 |
| 1. 上海人表示被香港人坑死 |
|  |
| 1. 公卫护士表示真的很忙很累很心酸，有苦说不出 |
| 1. 因为开放的态度 |
| 1. 别说了，居家了！ |
| 1. 和20年的病毒株不一样，传染性高。目前没有危重和死亡，轻症痊愈就好了。还有人因为过度防疫投诉卫健委呢。 |
| 1. 强烈建议中小学停课 |
| 1. 对啊，我们学校离竹园小学只有1km多一点，20年的时候都没感觉疫情有这么近 |
| 1. 上海这是被吹出来的‘精准防控’自乱阵脚了还是有啥别的原因？ |
| 1. 这次就属实不太能理解了 |
| 1. 别说听课了，我们公司隔壁一个阳性，我们依旧上班，物业政府没任何通知 |
| 1. 还让香港人进上海就离谱！ |
| 1. 就是啊，我明天还要去上班，浦东到宝山。。 |
| 1. 呼吁停课停工吧，天天看新增的人数，瑟瑟发抖，周围也有封掉的，要是被传染了，谁还干活，暂时不干活，为了以后天天干活，留得青山在不怕没柴烧 |
| 1. 现在的政策是动态清零，不是绝对清零，如果一有病例就停课停工还动态清零干嘛，直接绝对清零啊 |
| 1. 两会期间他们敢报吗 |
| 1. 我只知道外溢到其他省很多 |
| 1. 上海人身体好！都是无症状 |
| 1. 一把支持 |
| 1. 都是被网友给吹出来的胆子，说啥精准抗疫 |
| 1. 淡定点，迟早要放开的，无症状这么多根本防不住，除非中国从地球村移走，都说了政策根据情况调整，反正我听政府的，该干啥干啥 |
|  |
| 1. 2020那是过年回家，不一样，这回是人都来了上海 |
| 1. 精准防疫当政绩搞得高调过头了，现在多点爆发不减少流动那就是四处传播 |
| 1. 因为要改变应对方式了，动态清零比较困难 |
| 1. 关键很多防控在捣糨糊！尤其核酸检测，松江某小区第二轮核酸检测随便瞎叫叫，白天来核酸，到点下班，有很多人都没回来？请问这个核酸有什么意义？排队扫码登记信息是一个队伍，到了核酸的时候分三个队伍，请问信息跟人员怎么对的上？这些机构就是赚国家的钱！浪费国家资源！让老百姓受罪！ |
| 1. 希望上海和吉林市人民平安，要健康！ |
| 1. 对对对我们不停课还要集体核酸 |
| 1. 爱上网课的就自己申请上网课呗！不要把别人拖下水行吗？ |
| 1. 就这周末还不延考？ |
| 1. 早上6点核酸现在没出结果，密接但是接的大巴等了半天没来，疫情到底怎么样了 |
| 1. 只有崇明没有了 |
| 1. 这么夸张？ |
| 1. 深圳夸张多了，不还是一样 |
| 1. 说实话我不理解上海这次怎么会这么严重，辐射那么多省份 |
| 1. 稀里糊涂各种松懈，进楼查码假模假式，隔离酒店各种感染，境外来的隔离不到位。 |
| 1. 可以停课了，市教委还要害死多少孩子 |
| 1. 都是轻症无症状的，毒性还真以为和两年前一样啊。说停就停说封就封完全不考虑老百姓的心理 |
| 1. 居家办公说的容易，很多工作根本居家不了啊，该上班的都在上 |
| 1. 外溢很多城市了已经 |
| 1. 这还不算境外输入我附近有一个小区今天确诊无症状，然后这附近的小学还在上课¡评论配图 |
|  |
| 1. 这个表在哪里看呀 |
| 1. 我们吉林已经600多例了，人口还不到你们的十分之一啊。。。 |
| 1. 快居家吧 |
|  |
| 1. 我们办公室10个人已经2个人去隔离了，还不居家办公 |
| 1. @Fan-Zong我觉得很有道理还不停课 |
| 1. 看市委有多大的胆量吧！ |
| 1. 当枪使呗失职也只是被免职 |
| 1. 崇明挺住 |
| 1. 我在上海目前都不知道多少例了 |
| 1. 上海有些方舱可能在你认知范围之外 |
| 1. 不是骂转不转，而是执行的过于粗暴 |
| 1. 武汉的是方舱医院，上海的是方舱 |
| 1. 主要是具体操作的时候很操蛋，在方舱也没人管医疗条件也不行，还折腾老人(各种接驳效率极低)。送不进医院不知道是不是因为已经爆了，院感也挺严重的。世博方舱都改建重症病房了，目测情况还是比报道的严重很多…… |
| 1. 有没有一个可能，你支援的地方不是上海 |
| 1. 你知道现在的方舱是什么样的吗？屋顶漏水，马桶不通，根本没有医疗服务，你忍心让你的爷爷奶奶外公外婆半夜2:00被拉过去吗？ |
| 1. 上海的方舱很多都是临时征用，不具备条件的，非常混乱 |
| 1. 上海方舱啥也没有。 |
| 1. 但是他还是死在了方舱 别人已经很难过了 你嘴下留情吧 |
| 1. 上海的方舱没有监护设备。连花清瘟都没多少，出仓时候有人有多余的，给到护士台，护士都不停道谢 |
| 1. 不是太同意……我觉得超过70岁需要直接转到设备全的医院了。好些都有基础病的。方舱连基本设备和药都无，也无人看护，所以老人才宁愿在家啊。久病的一般家里都有些基础药和仪器了 |
| 1. 你知道上海的方舱长什么样嘛 |
| 1. 一千人一个马桶还不通水，屎尿横流的“方舱”吗 |
| 1. 方舱算很好的，很多就是临时隔离点，往里面一丢，死活不管 |
| 1. 这是一家带护理的，如果你知道医院病房有多难收，就不会说为啥送到养老院了 |
| 1. 那个“全篇负能量”的视频做了什么呢，它收集了封城以来很多上海市民们有记录的音频，配上了情况简述……要猜猜“负能量”的究竟是什么吗 |
| 1. 并不是医疗资源不够用，是医疗资源跑去搞核酸了 |
| 1. 别的城市都恢复正常生活了，就它们要躺平要自生自灭，多可笑 |
| 1. 清零要果断快速，而不是拖拖拉拉。错的不是清零，而是拖延。 |
| 1. 不清零，医生感染一轮又一轮，死的最多的就是老人，然后是医护 |
| 1. 医院瘫痪，救护车转运不过来，上海政府这波绝逼有问题， |
| 1. 量变会引起质变，2500万人的城市正常运作其复杂程度不是你能想象，上海在努力延缓疫情扩散，但终究是挡不住的，给全国人民做试点是上海这座城市的历史使命，你们现在可以骂日后心里会明白 |
| 1. 对，算小号流感，我们一家都转阴了，包括老人，但是如果生病就看不了病，你觉得呢 |
| 1. 其实国外早就研究证实了，对于奥密克戎来说，严重程度排序，不打疫苗＞流感＞打了疫苗。所以对打了疫苗的人来说，就是小号流感，只不过国内没有报道，国内还在一如既往地妖魔化。 |
| 1. 新冠毒力一年比一年弱，封控力度却一年比一年高压，真的痛苦!我经历了今年泉州30多天高压封控才能和上海人感同身受。一群累的要死的医生照顾着一群精神抖擞的病人，不知道意义何在! |
| 1. 这脑袋没救了，竟然怪上防疫政策 |
| 1. 我今天看到个思政题目，关于上海的，而且政府职能转型的，而恰恰上海浦东就是政府职能转型的试验田，就是小政府大社会，依靠社会力量来管理城市，结果上海这次最乱的一次尤其发挥了社会团长的力量 |
| 1. 然后他们也成功了，很多百姓都觉得中央动态清零政策很有问题，别人说觉得还是要按政策来，他们回复说，恭喜您，被圈养的猪也是这么想的 |
| 1. 明明几年了，上海阳还是越来越多，医疗条件尚好的上海都这样，不敢想放开会怎样。老家村里全是老人小孩。 |
| 1. 早期防疫不到位，任由后期疫情泛滥控制不住，是上海政府防疫政策不当而不是清零防疫错误 |
| 1. 这不是防疫造成的，是当地防疫不力造成的，真正的动态清零，现在就早回复交通了，哎，当地政府简直太拉垮了。去年西安的封城也造成几个人的死亡，初期送医院也是不及时，都是政府前期不作为造成不得不大面积封城 |
| 1. 没本事你清什么零？自生自灭也不会这样吧 |
| 1. 因为人们看到了根子所在，这就是早期放任后的恶果 |
| 1. 所以120几个小时不到，救命药买不到是那个人造成？脑子真是混乱 |
| 1. 回复@猪小蒸:救护车不够用是因为都拿来转运🐑了 |
| 1. 这是哪里？确定是上海吗？ |
| 1. 放任受难五人与欧美政府无关，于我们政府不可能无关 |
| 1. 发我？ |
| 1. 真的有用吗，有没有人处理呢 |
| 1. 回复@半盏清茶吖:有的啊 我是长宁卫健委受理 在等答复。相比之下12345是摆设 |
| 1. 理想是美好的，现实是残酷的。 |
| 1. 上海人民已封闭3个星期了，极端的都有40多天了，还要怎么配合？站着说话不腰疼。 |
| 1. 你在搞笑吗 老人90几岁 父母70 怎么照顾老人 我们要上班要加班 你觉得是差口饭的问题吗大聪明 老人是需要24小时照看的大聪明 |
| 1. 很难理解吗，有个40万人阳性但0高风险的地区，不劝返让人怎么办 |
| 1. 有什么难理解的 有外省回去感染扩散的话 村镇的能力根本撑不住 |
| 1. 上海什么时候嘚瑟过，什么时候又嘲笑过别人，姐姐可别张口就来，嘴会烂掉的 |
| 1. 之前嘲笑浙江全民核酸的上海人吗 楼上的选择性记忆是吧 |
| 1. 至今还记得朋友圈有个上海的学姐，当初西安疫情的时候骂西安那叫一个恶毒加泼妇！还说再也不来西安了，现在呢，考研没考上，现在又来西安了呢！现在又开始在朋友圈里洗白了它的家乡了！真的贱呢！ ​​​ |
| 1. 刚还给西安买了个热搜呢，评论几百的“西安飞机”直接热搜第一 |
| 1. 当时嘲笑西安这不行，那不行。说西安废都 |
| 1. 上海有没有嘲笑别人我没看见，我第一个就看见你在这落井下石了 |
| 1. 我以为只嘲笑了西安，原来还嘲笑了浙江 |
| 1. 上海笑其他地方？？？？上海是个城市啊，上海又不是个人，怎么会笑？？？？你怕是有点懵噢 |
| 1. 还真嘲笑了，之前深圳年前一波疫情的时候全民核酸我记得有不少人说浪费纳税人的钱，费钱费力，还拿上海只封了一家奶茶店做对比 |
| 1. 西安和吉林被骂惨了 |
| 1. 笑死了，一说嘲笑就说是西安人，敢问全国哪个省市没被你们嘲笑过 |
| 1. 那个十六夜爱黑兔是黑西安的老人了，我见过她很多次，但是她不是上海的，只是善于利用矛盾挑拨离间的而已。 |
| 1. 其实吧，上海的优越并不在于防控措施有多先进，也不在于防控有多精准，而在于抗疫过程中，行政部门试图释放的最大善意，尽力尊重和少折腾市民，以及努力实践把人民当主人，自己当公仆的姿态。 |
| 1. 你的意思是经济不需要考量 生活中除了这个病毒以外的其他事情不用考量对不？ |
| 1. 归根结底还是国外民众所谓的自由，结果成了病毒培养皿，变种快的连亲妈都不认识 |
| 1. 管香港什么事……真的是奇怪深圳现在都好了，上海还能怪到香港去。 |
| 1. 上海这边嘛是等检测到再封无用功很可能人早就全国跑了代价太大没啥用，香港那边你可以去知乎看看，那边的人普遍反对封城 |
| 1. 疫情已经两年多了，也就中国受得了这种防控。其实我挺理解香港不封城，因为迟早得与病毒共存，一个国际城市封封停停解决不了问题，奥密克戎也算是抗体疫苗了。 |
| 1. 偷偷说一句，香港大概率是试点，不过对深圳冲击太大，不敢试了！上海可和这个关系并不大！就目前深圳受冲击以后的到现在稳定下来后！ |
| 1. 香港孩子大了，叛逆期不听父母的啊 |
| 1. @桃花味的果果 说别人的时候也不觉得心虚，呵呵 ¡评论配图 |
| 1. 还有深圳五区昨天发布了“非基本运转党政机关人员一律就地转为志愿者”，看看这个力度。 |
| 1. 别用“大家都”，广东这么大的压力，一样做得好好的，我一个北方人在这边工作，两边的防疫紧张程度感受很深，即使没有疫情，也是飞机落地就做核酸。 |
| 1. 人没钱更可怕 |
| 1. 骂林郑吧， 都是她搞出來 |
| 1. 如果你觉得你们地方管控非常合理的请申请你所在城市接待国际航班和香港分流人群 |
| 1. 你去看下之前每日输入病例再再来阴阳怪气 |
| 1. 大意了！民意一方面，历史已经证明关键时刻靠英雄！ |
| 1. 那你还是视野不够，这是全球性疫情，全国各地没用。而是要与世界同轨 |
| 1. 深圳2022年一点没放松的封控前提下，确诊人数破四位数了，你觉得这只是惊弓之鸟？ |
| 1. 深圳人已经连续第十八天核酸了，明天后天大后天……还要，对面的人到处跑，我们喉咙捅破了也没用，放着我们哪个城市这么严重的疫情，人还能全国跑，隔离又怎么样？深圳上个月确诊十几个隔离酒店的员工…… |
| 1. 错在，大都市明里暗里一直在搞“与病毒共存”，自以为是…别忘了大都市不是孤岛…是在大陆，大都市造成的无症状者引起了全国多点地的爆发……即使这样，国家也没说什么…咋了，你们现在自己出问题了，还要碰瓷？ |
| 1. 再多的理由，也无法摆脱现在上海现在近于失控的责任…… |
| 1. 那个时候有没有奥米克隆？说话不要就只揪着一点。现在上海躺平了，我看看昆明卖花群里，都是滞销的花。我们上海人不买了，都去买青菜了！ |
| 1. 某医生是谁啊 我记得张文宏说过 他不同意躺平 我看到视频的啊 |
| 1. 要不要打个赌？看4月份防疫政策取消不取消？ |
| 1. 大都市有个医生，比你说得还好…但是做的一般化… |
| 1. 回复@扎紧你的篱笆:防疫政策老百姓说了不算，一个医生说了也不算。专家组，政府那么多人，一个政策制定下来要层层批示。出了问题先解决问题，去怪一个说话的人，那以后谁敢说话。 |
| 1. 同在上海 非常赞同上海的精准防疫政策 很安心 |
| 1. 我们现在也希望政府能够停工停学，但是我们希望没有用啊，我们只是老百姓 |
| 1. 这次感觉好多人都在内涵上海 搞不懂上海这几年承受了多少境外输入没有点赞的 这个时候了 都来落井下石 我很是心痛这些人都是按的什么心思 |
| 1. 我就问一句现在这样情况了要不要全员核酸？ |
| 1. 表示很委屈 |
| 1. 停下经济全员核酸不适合上海的，上海的经济也不是只贡献上海的。总得有个办法长期共存的。精准倒溯有效治疗是目前比较好的一个手段。 |
| 1. 看你的前半段以为你在夸上海 全员核酸有什么好的？单纯好奇 杠就是你赢 |
| 1. 说少了，一班飞机三百人，一天N个香港航班 |
| 1. 还有一条线，就是香港那个偷渡来的 |
| 1. 起码某地人没有明知道有疫情，还特地往外逃，当年类似的事不是没发生过。我是某地临省的，客观如是说 |
| 1. 你确定他们那边有机场？ |
| 1. 朋友 别担心 上海和美国一样发达 不会有事的 |
| 1. 孙悟空画圈了 |
| 1. 昨天一粒确诊在我小区隔离，怕啥 |
| 1. 劝你善良 |
| 1. 嗯，就怕过了这阵，又跑来建设上海！ |
| 1. 你们没外溢过 |
| 1. 呵呵 你觉得国外群体免疫牛逼的话 买张飞机票送你去实地体验 |
| 1. 我们人口基数大 一大意医疗系统就会爆 到时候会有多惨烈 只怕你想象不到！ |
| 1. 你刷身份证就能看到你核酸没 是不是绿码 |
| 1. 根本不考虑上海老百姓的感受。最倒霉的就是医护人员。真的气这次 |
| 1. 上海领导要搞政绩呀。 |
| 1. 真的是不要脸，搞得大批香港人入境，不把上海老白姓的命当回事，等着现世报 |
| 1. 外地领导为了自己，把上海人卖了 |
| 1. 谣言来的，上海政府并没有说要自告奋勇接受香港人，是港人深圳关那边实在进不去了，所以很多就开始动上海脑筋。因为北京需要核酸检测，香港飞上海不需要核酸 |
| 1. 要政绩拍马屁啊，2020年疫情刚开始骂扑天雕骂到封词条都忘记了？ |
| 1. 能把香港航班熔断吗？ |
| 1. 香港人又不把大陆人当人的 一贯在上海拿着比同级高的待遇一边鄙视 |
| 1. 青浦也是香港人去专柜修手表 据说是这样 |
| 1. 深圳都不敢趟这个雷，魔都够魔性，大家跟着倒霉 |
| 1. 没错，对得起上海百姓吗？为了自己升官发财，不考虑一下上海入境人数的饱和度吗？恶心死了 |
| 1. 香港确诊都有211302了还不封城我是真的会谢害得我也要居家隔离了 |
| 1. 看看哪个才是天龙人了，现在看来，香港更胜一筹 |
| 1. 香港和武汉一样也是我们郭嘉的一份子，上海作为一线城市帮助分担一下抗疫压力难道不是好事吗？这也是作为一个大城市的担当。只是中间发生了一点意外而已，谁都不愿意这样。 |
| 1. 回复@桃映qwq:亲爱的，生命诚可贵。才能考虑其他的 |
| 1. 为啥？其实网课质量也不差的 我网课上来数学突飞猛进 如果都初三高三了 自己还不能自控 说明根本就没想好好学 这种时候就是看自控 |
| 1. 明天早上肯定更多 |
| 1. 之前天天笑外国防疫差，现在被打脸打到手软 |
| 1. 这是最近几天的确诊和无症状。 |
| 1. 我看着确诊的人数咋没有填上？我们这里有学校停课了 |
| 1. 没有自告奋勇啊，这谣言来的，香港人深圳进不去，第一想到就是从上海进但有点很扯香港飞上海不需要核酸检测 |
| 1. 香港已经测给你看了，快3000死亡，就内地这个医疗资源，多少人死哟 |
| 1. 香港现在死这么多人“测试”还不够?敢情死的死的不是你的亲朋好友就能无视? |
| 1. 香港输入472名病人 |
| 1. 我觉得是换领导的问题 |
| 1. 我直接不去了，主要是没看书 |
| 1. 还有市考面试，绝不延期哈哈 |
| 1. 是的，松江整个教育系统通知明起3天内，所有教工和学生做核酸，不知道为啥还不停课 |
| 1. 哈哈哈哈哈哈 |
| 1. 香港输入太多确诊病例，472人。 |
| 1. 香港害人 |
| 1. 怕感染你可以不去啊…… |
| 1. 麻了，怕感染的可以不考ok？没人逼你去 |
| 1. 这波疫情中的病例，也有自行去就诊发现的。 |
| 1. 回复@不渡_Sigma:本地人支持，排查一下更安心吧 |
| 1. 大夫，社区的人真的累吐血了，他们太不容易了 |
| 1. 不是所有学校都停哈 |
| 1. 我弟学校还没停 |
| 1. 青浦不是早就报了么 |
| 1. 是的，其实是经济扛不住了，减税降费，防疫支出，根本吃不消 |
| 1. 如果为了疫情防控有必要，我全力支持封城！ |
| 1. 疫情可怕，经济不好也可怕。如果国内几个特大城市也一关了事，会发生什么事？很多省市都指望北京、上海、深圳、广州的税收转移。 |
| 1. 准备核酸了 |
| 1. 回复@一条看不到未来的咸鱼轩:天…真的怕了 |
| 1. 说的轻飘飘哦，与疫情共存是要拿人命来垫的。 当感染到你家人时 ，你也能这么理性和“先进”吗？ |
| 1. 担惊受怕，只能嘱咐小孩子尽量保护自己 |
| 1. 我特别想回去有吃有喝不愁 |
| 1. 放心，明天无症状破400，本土确诊依旧个位数 |
| 1. 本来就是线上复试吧，应该没什么影响 |
| 1. 回复@frankcq19:我们线下考手绘，线上就说不准啦 |
| 1. 那请问你怎么知道他经过上海之前没确诊？ |
| 1. 哪天就有人说实际确诊人数是公布的十倍。 |
| 1. 医疗资源过度消耗，不够用了 |
| 1. 还要自备干点 笑死我了 |
| 1. 退了吧 |
| 1. 无症状真的 救命 |
| 1. 哎周六我们学校全员核酸，真的为什么还不停课，还有封校只封学生，不封教职工，有什么意义，他们就不会遇到密接，他们全是免疫体吗 |
| 1. 可以倡导，尽量的话，总归少一些危险 2. 共同加油！ 3. 上海会好起来的 4. 切块封控，时间差就是里面最大的漏洞，同事A街道封控了，回去隔离，但他是夸了两个区来的，还和办公室的其他人发生交叉。 5. 这个人口罩能带规范点吗？国际化大都市上海发言人带口罩露出鼻子？这还有防范意识吗？ 6. #上海目前没封城也不必封城#上海市召开疫情防控新闻发布会，在回答上海是否会强化管控、是否会封城时，上海市政府副秘书长、市疫情防控领导小组办公室主任顾洪辉表示，目前上海没有封城，上海也不必封城。上海将根据区域风险程度，划定若干个风险区域，实行网格化核酸筛查，进一步强化社会面防控 7. 上海现在就是摆出一副你不喜欢又干不掉的样子 8. 这样的管理的确远超国内其他城市水平 9. 上海市委市政府就这种组织动员能力 10. 小区挨个封了而已，我和我同事轮流被封 11. 地方政府很有智慧和魄力 12. 上海为了人性化防疫殚精竭虑跟某地一刀切只顾新冠数不顾百姓日常可是完全相反的现某地对落井下石异常热衷喜欢把自家政策带来的巨大弊端拿来同比当初那几条某地关乎人命的新闻全国可都是惊掉下巴的如此漠视生命…当初有多关切某地民生骂得就有多凶如今有多理解自己的政府心疼就有多甚 13. 整个中国负重前行至此都不容易，那些恶言恶语阴阳怪气并不会阻挡我等前进的脚步也不会扰乱我等防疫的决心和信心。防疫是一盘大棋，不存在哪个地区拖累哪个地区（除HK）大家都付出很多。当务之急度过此次难关尽快恢复生产，前面还有更大的困难需要攻克。 14. 这么自信，那为什么这节骨眼上还有的人需要自费核酸，免费核酸只对小区居民，其它外来务工者缺需要自费去医院才能核酸？ 15. 上海接近半封城 16. 放下偶像包袱，认清现实 17. 坐标宝山，路上人流量和往常一样，老百姓该干嘛干嘛，完全不受此次疫情影响！ 18. 嘉定这都封三天了 19. 还不如封了，现在做物流疫情影响太大了。客户各种逼各种催 20. 不恐慌、不添乱，做好个人防护！ 21. 华东剿总的老巢不是被端了吗？ 22. 可以通过网格化的筛查达到相同的效果，减少对经济的影响 23. 下文件居家办公吧赶紧每天坐地铁还是人挤人 24. 工业区隔两条马路出现阳性，公司还让上班，园区大几千人呢，政府明确出台规定行不行 25. 停工停运什么事也没有了现在病例比武汉那个时候多那么多传播速度提高了更应该在家里不聚集一个一个封人是流动的确诊的人流动了你再来封一个区块有什么用该停就要停啊为了经济方法错误最后人还是越来越多业绩少了也就少了病例感染人多了乌纱帽保得住吗？ 26. 多多买菜和美团优选都停了几天了，马上没纸用了 27. 多多买菜和美团优选都停了几天了，马上没纸用了 28. 可以不封城啊，也可以精准防控，但别外溢啊，外溢十几个省几十个市，真牛逼啊，要是不外溢，精准防控确实是典范，但是你外溢了还吹啥？ 29. 上海疫情花桥被封了 30. 新冠专治各种不服 31. 我去 32. 一个月前说没必要封？？现在看看真是有点好笑 33. 那就别外溢了 34. 愤怒的有理有据 35. 就自己不停摆……到处再往扬，扬得别人停摆封城呗城市都会精致的利己主义了 36. 希望可以在严格管理一下，妹妹在上海上学说真的每天看见新增真的是太担心了 37. 太小看奥米克绒，BA2专治不服。 38. 你不封城结果把病例传的全国都是，精准外溢能不能别吹了啊， 39. 周边城市自己封高速入口，所以强调不封有什么意义 40. 不封城可以别在外溢了呀，外溢了那么多 41. 没封也相当于封了。目前没有过封控管理的还有哪？从一开始就没必要死鸭子嘴硬，导致现在严重的后果 42. 没封也相当于封了。目前没有过封控管理的还有哪？从一开始就没必要死鸭子嘴硬，导致现在严重的后果 43. 你哭啥呢？ 44. yaoming生物为了让员工不会因为隔离不能去上班，让小区被封掉的或者可能会被封掉的人不要回家，去住公司旁的酒店，还要自行先垫付 45. 还不如封城，至少我可以居家办公，不给社会添乱。 46. 不如先落实下居家办公政策，政府不明确居家，领导就是不放，哪怕因疫情没事都要去。 47. 小区都封了跟封城有什么区别自欺欺人 48. 别再自以为是了生病的不是你后遗症也不是你承受能不能负点责任啊人还在增加 49. 不发文公司怎么会让职工居家办公呢，说的都是没用的话，根本没有考虑上海本土市民的安危，大白们多辛苦啊，早些停工停学也不至于这样 50. 现在和封城有啥区别呢，班不让上，人不让出，菜也不让买，家里都没粮食了。 51. 不封城？那到处封的小区是啥？感觉一个接一个的封，但就是不说，给大家造成了极大的不便，不是说不配合疫情，你这样只封小区，但是单位还是要开门，上班的人要疯 52. 还是面子比较重要，苦了基层，苦了百姓。 53. 别再倔强了承认错误那么难？ 54. 精准化现在根本不适用了，也不看看情况 55. 一会两天，一会七天，一会按单位组织核酸，一会按学校核酸，一会按小区核酸，一会按区域核酸，一开始就没有接受事态的严重性，弱化各种事件，防控工作重复化，无效化，浪费各种人力物力 56. 阿弥陀佛！愿大家都平安健康！愿疫情早日结束！ 57. 可是封小区了菜都没得吃了 58. 要想完全控制住疫情，不封城是不合理的；上海目前今天关这里，明天关那里，就像打地鼠一样，这头按下去那头又起来，还是流动太强的关系；期待政府勇敢封城 59. 隔离第三天，希望疫情早点过去 60. 宣传工作做的太好，反噬了吧 61. 还不如封城，大家都关在家里安全 62. 上班上到感染然后全部分得病 63. 这口罩戴的 64. 就这还没必要？每天新增一百多。 65. 只进不出好点，潜伏期去的人携带了也不知道 66. 唉，希望快点儿好起来吧 67. 非不要不离沪! 68. 封了吧，现在情况已经转好了，就怕某些老乡再来霍霍我们 69. 你也进不来别的地方？ 70. 看结果吧，成王败寇。 71. 股市也关了吧。 72. … 73. 对呵呵 74. @朱_就这样吧一个月前 75. 这些人不用处理吗？ 76. 我只相信白猫黑猫，有一点：防疫时间机会是有限的，感染数量的理论可承受数也是有限的。 77. 谁给上海的勇气不封城??！！要点脸行不！！ 78. 已经封了 79. 我到宁可封城 80. 说的话轻飘飘的建议企业错峰上下班，建议不要乘坐公共交通。比如公交地铁，拜托有些人的通勤时间单程就要一个半小时，难道天天打车吗，关键打车也不安全 81. 可怜的打工人，每天上班都在跑毒，不知道出了门能不能回来，回来了能不能楚门，啥也不说我就呵呵，讲真还不如全部一起封闭管理， 82. 现在已经封了，雅称封闭所有小区 83. 咋地，封城碍着你挣钱了 84. 不容易啊 85. 不封不全员核酸，能干过奥密克戎变异株？静看实践摸索效果 86. 嗯...上海旁边的平湖市封了...新仓镇封了... 87. 上海没钱了，所以查查邓伦收缴点钱填补亏空 88. 上海确实是一座让人无语的城市 89. 我只希望那些骂上海的人有骨气点千万不要来上海！感谢！ 90. 不要拿江浙沪和东北比 91. 为什么不居家办公 92. 国际化大都市 93. 他这个口罩……没戴好吧？ 94. 商长和银行等停止营业，这跟封城有啥区别？ 95. 实在不行封城吧，把境外航班分分流到别的城市去不要一个人硬扛了 96. 能别天天说上海传播给其他地区疫情嘛，这不正说明这波奥密克戎BA.2传播速度快传染性强吗，而且隐匿性也强吗。别的城市是怎么防不住上海来的，上海就是怎么防不住别的城市来的，一样的道理阿，上海人跑到别的地方传了，说明别的地方防疫也没做好阿，上海是制造病毒了吗？ 97. 停课了，汽运停了，上百小区封了！还没疯？！怎么想的？ 98. 是啊不要乱跑啊你们是阳光明媚、不必封城，全国28个省替你们负重前行。 99. 外地人别再来上海了啊外地更香啊特别是安徽人！ 100. 理解不封城，就是能不能管控严格一点 101. 我们就1例封城了 102. 上海现在的政策是绝不会像深圳一样出文要求居家办公，上海的政策是不封城，不停摆，坚持上班，上到确诊。 103. 上海现在的政策是绝不会像深圳一样出文要求居家办公，上海的政策是不封城，不停摆，坚持上班，上到确诊。 104. 天龙人，我发现了不光是市民高贵，政府更高贵，建议自立为上海合众国 105. 不聚集 106. 社会面清零 107. 转发微博 108. 嗯嗯 109. 后悔吗 110. 打脸 111. 如果上海这批没有处分我真的会不理解小城市疫情处分一大片这玩意不用管？ 112. 现在回头再看这条视频 113. 上海还区别对待外地人，给本地人送菜，不给外地人送菜，垃圾，有什么好牛 114. 你知道近期山东新增病例全是从上海来的吗 115. 上海了不起啊！这下好了，疫情控制不住了 116. 资产阶级竭尽全力的向民众灌输一个观点。资本家的财富就是整个民族的财富。即使是伦敦东区最贫穷的爱国者，一想到英国的财富和工业。便会不由自主地挺起胸膛。--恩格斯《论英国工人阶级现状》 117. 这人吧有时候就不能把自己太当回事，城也一样。 118. 去年买了块表 119. 今年疫情不封城，封城就说“暂停键”“慢生活”“静下来”。 120. 最近我们这好几个确诊的都是因为上海回来的人导致的，有什么自信这样说 121. 请问现在核酸多少时间出来开什么玩笑5天都没出来 122. 铜陵1+3源头上海来的 123. 精准防控，把病毒全防出去了是吧 124. 然后全国各地区都开始封闭了，也挺好的，毕竟我们都是乡下人嘛 125. 精准防控，真牛啊，精准到全国都是了 126. 能力不够别吸血当什么超大型城市 127. 自由 128. 无锡被上海人害死了 129. 还是上海牛逼，有自信，还能干实事赞👍 130. 先把居家办公落实起来吧不出通知企业都抱侥幸心理 131. 动态清零万岁 132. 我们上海能做到精准外溢，凭什么要我们封城 133. 因为直接封小区 134. 可是上海跑回十堰的，确诊了啊！还经过了汉口 135. 上海精准防疫就是最大的笑话封了一个奶茶店一片叫好但是背后却是管控不力导致疫情外溢出了问题就是瞒报粉饰太平 136. 这次封小区确实多，但是并没有都封啊，坐标杨浦，很少很少被封，上班也正常。zf不封城是想尽量不影响群众生活。动不动就封城才是懒政做法。 137. 一帮收入不高的小老百姓在网络上大放厥词，什么上海人不乱跑咯，什么精准防控放嘴边咯，我想说的是:中央的决策领导上海，到底到哪一步上面最清楚。你们该支援二三四线城市就去支援二三四线城市。别的不要多管。你听过上海需要支援的嘛？最好的条件和医疗设施都在上海。过来也是此地无银三百两。 138. 早封现在估计都好了 139. 看了下评论，满满的对奥米克隆的恐惧感，哎！ 140. 能不能把口罩带带规范在讲话？ 141. 出去照样被隔离，有啥用呀 142. 没封城？上两天不是发非必要不离沪，客运站也停了。这么多小区都封了，承认一下会死啊 143. 可以问什么时候恢复家装吗？ 144. 这叫精准防疫，都学着点 145. 不封快递别停啊…… 146. 求你封吧 147. 发言人口罩戴的个寂寞 148. 领导，请睁开你的双眼。每个区核酸检测点就那么几个，天天排2个小时长队检测，拿着检测报告才能进大楼上班，第二天下班又要去排队做核酸。那么多人挤在一起不危险吗 149. 隔离酒店都不够用了你们知不知道啊到底 150. 不封城可以理解，但是得保障每天正常上下班的人的健康安全问题啊，建议早日出台居家办公政策。 151. 不是新冠才是病，还有很多病人要去上海看病 152. 真踏马扯淡，疫情正是要紧的时候，自己竟然松劲儿了，14天的隔离10天就全放出来了了，每天一百多无症状，早晚高峰毫无措施 153. 但是学生都封了 154. 上海封不封自己还真的做不了主，这个得听国家的安排吧 155. 对地域黑来说上海这两个字是原罪疫情当前，大家为什么不能团结一点不要吵来吵去了 156. 每天至少跨三个区上班的人就看看 157. 自己不封靠周边地区封，上海一些低风险地区都不能正常去外地了，去了就被14＋ 158. 上海加油，我现在就因为小区有确诊在隔离期，希望春天马上到来，期待解封的那一天 159. 不能说上海。。只能骂北方（东北，西安等） 160. 我真的好烦，啥玩意刚进小区十分钟封了，都不能说一声我就不进了 161. 先把居家办公落实起来吧不出通知企业都抱侥幸心理 162. 居家办公吧！！！ 163. 真牛逼啊 164. 这个口罩带了个寂寞。。 165. 五雷轰丁了。 166. 华新镇怎么封了？ 167. 反正咱公司是没有条件 168. 上海就是废 169. 身边的小区都封了希望上海的疫情能遏制住 170. 反正家在上海，不会跑的 171. 不封城我们也不会逃去外地OK？全球都没有比上海更安全的地方了！不要自作多情以为我们会跑去你们老家！ 172. 封城吧 173. 其实各省可以对上海来的全部隔离 174. 抵抗疫情，备不时之需，需要鸡蛋，鸭蛋，鸡鸭等生活食用品，上海各个小区都可以专车送达，需要的可以联系 175. 不封城封小区啦，一回事 176. 上海疫情到底严不严重，怎么能带回到我们这边啊 177. 公司今天货都发不出去了、还不是封城是什么？ 178. 笑死了 179. 核酸四十八小时没出结果了，还有用？ 180. 明明安亭就是封城 181. 不封城没关系、你倒是把城里控制好阿 182. 搜索“上海疫情”给我推荐“西安疫情”¡评论配图 183. 不是在闭环了，就是在闭环的路上。 184. 叛徒太多！！等疫情防控好了，再抓也不迟！ 185. 现在是按街道轮流封。不是一次性封，大规模排摸呢 186. 小区被管控48小时的出来集合下 187. 我的店从4号开始就不准开了，和封城有什么区别？什么时候能开也不给明确消息，到月底的话是不是这个月房租就白交？全家吃什么？ 188. 我同事封了一半小区，你敢信？ 189. 没封城。。但是周围小区都要封 190. 都被封在家里了，也不说多久，做了几轮了，就一直封在家里，服了 191. 真有意思，小区封了。我已经在体育馆睡俩礼拜了 192. 死鸭子嘴硬 193. 核酸报告能不能快点啊，说好的封闭小区两天，到现在因为核酸报告出不来不能解封，真的烦死了 194. 没封城快递进不来 195. 虽然没封城，但是已经有很多国内航班停飞或者取消了～ 196. 这就是上海，上海yyds～ 197. 花桥封镇了呢 198. 那我们学校怎么不放我们走 199. 还不如封了呢 200. 2+12的前两天已经听话的在家配合核酸度过了，第三天一整天也没等到解封的通知，不知该怎么向公司老板交代，要打工人怎么申请居家？ 201. 没封城可是也买不到吃的啊，小区出不去，周边商场商店都关了，网上买菜也买不到，也没提前通知，一觉醒来就出不去了，已经吃了三天泡面了，坐标嘉定 202. 闵行徐汇区大多全员至少一次筛检，但还是好多区域连一次都没有…一样地铁上下班，这是什么逻辑… 203. 你先学会怎么带口罩吧 204. 不见棺材不落泪 205. 坐等打脸 206. 死鸭子嘴硬 207. 坐等打脸 208. 呵呵 209. 分批封就不算封城 210. 精准溢出 211. 双职工家庭苦不堪言啊，孩子在家网课，家长不休息，只能请假，老板还不乐意让你请假 212. 嘉定这边外卖都吃不到了 213. 不封城也别让乱跑啊，这无症状感染者核酸都检测不出来，控制太难了 214. 封呀，你们以为我们不想封？入境航班你们哪个城市出来全接走，体现一下你们城市管理水平。千万别还回来！ 215. 别吹了吧 216. 看看这里都是什么地方的人在酸呢？你们那里没本事，不科学，就不要出来啊 217. 原定今天的入职也推迟了，彻底无业人员了。加油吧，早日清零 218. 希望不会有反转 219. 呵呵 220. 动态清零和精准防疫都是为了最大限度照顾到群众的生活，所以现在上海现在需要封的地方封，不需要的地方最低限度生产活动。大家都在坚持，一线防疫人员和流调人员非常辛苦，他们只是希望可以尽早恢复日常的生活活动。 221. 不封城，不停工，外面人员任意跨区流动，坚持上班，上到确诊。 222. 还想重演20年历史 223. 回复@shiziwuji2021因为没文化没知识所以骂来骂去只会这几句，感叹社会不公出生不公无力反抗，网络真好是个人都能畅所欲言，两句不合唯有先骂为敬 224. 这里却是一刀切…全部静止 225. 老年舞蹈队又可以跳起来啦 226. 说外溢嚯嚯其他地方的也别站着说话不腰疼，哪个上海人不希望上海别接境外别接香港，一天都是境外输入多大的风险。真要封掉其他地方的人一个别进来老子开心的做梦都要笑醒，会有那么多危险?不如以后境外的直接送你家门口隔离吧出事了你也别嚯嚯别人 227. 上海的底气！我们会胜利✌ 228. 都封在家里了，封城也没有意义了 229. 羡慕 230. 居家办公吧，我上班都胆战心惊 231. 疫情不严重的地方好多高速都封了 232. 可能就我们企业还在上班 233. 其实自觉的都很自觉除了上班买菜基本不怎么出门但你非要说那些没事出门溜达的人我也没办法 234. 没封城？你从曹安公路安亭检查站还有外青松公路白鹤检查站走走试试看啊？你看看你出的来上海不 235. 。。。 236. 坐等打脸 237. 上海不会忘了之前就是个小渔村吧 238. 呵呵呵呵 239. 特殊时期，多一些理解吧，防疫人人有责 240. 好的，这就出差去 241. 环上海隔离带 242. 辣鸡 243. 一说挨饿就要删我评论，好吧，党国万岁 244. 我们就在上海，党和政府怎么说都行，但是不让出门，快递全停，靠着发的一点点物资根本就不够吃，一直在挨饿，党和政府到底想怎样？ 245. 不要外溢了！ 246. 你牛，你清高，连累老子周六日都得上班 247. 谢你八辈祖宗 248. 不封上海封别处什么意思 249. 现在全国投送小阳人，来考古这个憨批 250. 不封城，祸害其他城市 251. 精准投毒谢谢您 252. 确诊患者和无症状感染者的迷之数据对比 253. 口腔科什么时候停诊啊！！！ 254. 封城是个蠢主意，是没招了的办法。 255. 上海早该全员核酸了，显摆什么精准防控！ 256. 不封城，周围省市都把你封了，＝封城，骗谁呢？！自欺欺人好玩吗？上海的领导 257. 希望彻查，不姑息，真的苦了老百姓 258. 应该对徐汇区都调查一下 259. 徐汇区区政府＋徐汇街道＋卫健委干部都要换几个了吧 260. 还是调查下香港吧！ 261. 连累了国家，连累了老百姓！祸害啊 262. 香港防控的失职又该有哪个香港的人来负责？哎。。。 263. 教训深刻。全国每个地方都一样，真的有吸取教训了吗？本土好不容易控制了。境外输入的把关，何时才能更紧？ 264. 【目前我市已成立调查组，正对华亭宾馆集中隔离点开展专项调查。我们会适时公布调查进展情况。（上海发布）】没下文...... 265. 为什么要追责，你们不吃下香港这波疫情会有上海什么事？也不知道是谁无能 266. 严查真的 267. 还真是华亭宾馆造成的啊，这严重的必须严肃处理 268. 还真是华亭宾馆造成的啊，这严重的必须严肃处理 269. 最好也要检查一下其他隔离酒店的管理质量 270. 怎么举一反三啊 271. 不应该啊！ 272. 比感冒症状还轻，不要恐慌 273. 最新的新闻说已经把上海的入境航班分流了 274. 最新的新闻说已经把上海的入境航班分流了 275. 最新的新闻说已经把上海的入境航班分流了 276. 华亭宾馆本来因为内部设备老化要停业装修了，后来不知怎么的就变成隔离宾馆了 277. 空调系统的问题。。 278. 上海这次的防疫工作让我大跌眼镜！今天大晚上的居委通知我们做核酸，然后先让隔壁小区的人跑到我们小区来集合，让我们去其他小区做检测，大晚上的窜来窜去，说是分流。没有登记哪家做了哪家没做，居委自己承认他们也不知道谁没做，所以这就是所谓的全员检测？ 279. 想不通，这些境外人员为啥在市区隔离，放到滴水湖不好吗 280. 请严肃处理接受香港航班并安排去华亭宾馆的决策者。海纳百川也需要量力而行，隔离酒店都塞不下了还举双手欢迎是啥心态？不要老是想着业绩，上海虽然是给领导班子漂履历的地方，但也请多考虑考虑我们群众。医疗条件再好，也经不起疫情的霍霍，关了那么多家排行有名的三甲医院，耽误了多少国内患者？ 281. 就不该用普通酒店做隔离点 282. 官话少些 283. 上海老是垫背，上面的没事想不到上海好，有事上海先开刀 284. 隔离酒店赚了钱，隔离人员不愁吃住。我们不能出门，没收入，付房租，还买不到菜。人生无常，大肠包小肠，赶紧是个头吧。 285. 香港停掉啊本地疫情上海人都抢不到菜 286. 现在有多少是来镀金的？没能力…… 287. 还没处理呢？ 288. 直接问责民航局吧！否则就是多做多错！ 289. 曹立强出来走几步！ 290. 举一反三 291. 厅级干部应该会免职 292. 受疫情影响，大量鸡蛋鸭蛋，大米滞留，需要的朋友可联系，专车送到小区门口 293. 抵抗疫情，备不时之需，需要鸡蛋，鸭蛋，鸡鸭等生活食用品，上海各个小区都可以专车送达，需要的可以联系 294. 至少有个态度了 295. 老百姓现在都人心慌慌的，华庭宾馆到底在干嘛？你收治病人就好好治，不要出差错好吗？非要让整个上海市都不安稳，希望相关部门尽快整治清楚。 296. 谁拍板的 297. 华亭宾馆没那么大胆子吧，罪魁祸首在哪里？ 298. 政府问题 299. 彻查，害人不浅 300. 请暂停香港 301. 为什么境外飞机大部分都往上海开？几年了能让上海歇啊 302. 彻查 303. 反正就是用我们的血肉铸起新的长城呗 304. 不一定是这个宾馆有可能某国是故意投毒 305. @在嘉定的鸡蛋哥：受疫情影响，大量鸡蛋鸭蛋，大米滞留，需要的朋友可联系，专车送到小区门口 306. 好好叫俗俗伊拉锦江集团卫健委徐汇区，才来了搞点撒？个泡烂乌到底撒宁测额 307. ？撒晨光发觉额？发觉为撒伐立即采取措施？满满抗抗要到几时？精准防控额牌子才 308. 那做摊忒了，好来现在把宁尬外地宁乱粗欧，糟斯摊色了 309. 疫情早日结束🙏🙏🙏 310. 全国抗疫是一家，上海加油！ 311. 上海加油 312. 上海，早点好起来 313. 上海加油！ 314. 上海加油！ 315. 加油上海 316. 上海快点好起来，加油 317. 上海加油我的阳春面 318. 疫情快点结束吧 319. 加油！！ 320. 上海人民有信心 321. 加油加油，事情只会向好。 322. 上海加油💪 323. 上海加油啊！ 324. 上海加油！ 325. 疫情早日过去，上海加油！ 326. 上海加油呀人人做好自我保护防范 327. 疫情来势汹汹，但我相信上海，加油 328. 上海需要鼓励！ 329. 共同抗疫一起加油 330. 上海加油 331. 加油 332. 上海加油！！！ 333. 上海加油！ 334. 上海加油鸭 335. 上海加油早日清零 336. 早日结束！！ 337. 上海加油 338. 评论内容 339. 以为放开后待在家里就没事的显然是太天真了。我在新西兰，自从政府躺平以后各行各业都出现了劳动力短缺的情况，结果就是超市商品缺货，点快餐没人送，网购快一个月才收到（快递病倒了一堆），厨师要隔离餐厅只能关门……同事一个个病倒所以我要做两个人的活 340. 共存了也回不到原来的样子，三天两头会有同事感染，三天两头的居家隔离，单位也不会满员，还要提心吊胆怕被传染 341. 这个问题本来就是看个人的。有些人生活很困难了他就觉得宁可病死也不饿死，有些人还没有到饿死的地步当然不希望全面放开。就像我发小一直在国外，之前一直是全面放开派，没事儿就嘲笑我在国内被封，今年他外婆新冠去世了，现在变成坚定的管制论者，网上也开始和放开论者高强度对线。 342. 关键是现在没有余粮的老百姓怎么生活？ 343. 说了很多遍，但我还是要说，所有吹全面放开的都可以去香港。有钱的去做生意，没钱的那边也招保安 344. 国家太难了大部分自私自利的人都要求国家保证他不死又得让他正常工作赚钱！两者能兼顾，美国还用死1百万人？？？ 345. 说完全放开的普通人大概家里没有老人小孩。 346. 问题是放开了可能更难吃饭，甚至没命吃饭，就算感染后活下来，一堆后遗症，能不能很好的吃饭都难说，如果是精神类后遗症，可能连滴滴等工作都做不了 347. 不用问，小区里出了一个阳性你看看小区其他邻居什么反应。网上的不是水军就是自己住在城市、父母住在老家那种，目之所及只有自己，脑子就全是自己轻症就没事我寻思也不用丢了工作饿死，就外卖一停看看他们饿不饿死 348. 总有人觉得共存后就皆大欢喜，属实是一厢情愿了，但病毒是唯物的，不是唯心的。 349. 国家只要说一声，新冠自费检测自费治疗，医疗床位价高者得，除了50w立马就没人说共存了 350. 最终肯定是要跟新冠病毒共存。这并不是我们的意愿来决定。而是新冠病毒已经不可能被从人类社会消除了。关键是，病毒的危害性要低到什么程度来选择共存。人类已有的好几个经典冠状病毒，危害性很低。就是带来感冒而已。虽然仍会有死于它们，但危害性很弱。 351. 但是我的朋友、亲戚在国外生活的，很多都已经感染完了，也没见他们有啥后遗症，也没见有啥不好的变化了，这里面甚至有70来岁的老人，现在也都还精神矍铄的天天出去运动 352. 感染后屌会变得很小，真的有人愿意吗？ 353. 深圳和上海疫情发生的时间点都是差不多二月底三月初，现在深圳基本恢复正常生活，上海才开始“划江而治”，如果上海是实验对比组，那么结论就很明显了 354. 放开的话就是生产消费双向奔赴了，一起跑向完蛋 355. 现在中国的动态清零模式很适合国情，只要控制的严格，一般半个月就基本恢复正常，慢的话20天一个月，到时候该吃饭吃饭，该逛街逛街，该旅游旅游（至少城市周边没问题），不比什么都没准备就放开了，大家更不敢出门强？到时候一部分人在居家观察，一部分人在医院治疗，我真的想象不出来经济能有多好？ 356. 谁主张放开，就先让他去海外疫区待几天 357. 来自香港.在有条件下，还是合理封闭管理好.病毒不认贫富、国家地区.生命没有就没了，而且这个病对老幼有影响力，即使打了疫苗复元后，也是有后遗症，是长远医疗负担.加上现在变种大约一年更新一至两次，还没稳定变完.总之有能力下，千万不要躺平.只能难听说，病和死不到自己身边的人，还是不知痛 358. 喊开放的，很多都是二鬼子。 359. “感染了，还是吃不上饭” 360. 支持放开的话人要有这个准备，就是病了看病花钱或者对身体造成的损害要能坦然接受，不能再啥都怪国家了。人家外国人得病了，也没有赖着国家 361. 别拿国外比较，人口基数都不一样，单拿美国来说，中国死亡人数按他们死亡人基数乘五，都能接受再说全面放开，另外还有一个感染人数上亿，免费治疗医保局底裤赔光都填不了这个坑 362. 我完全支持清零。可我家现在真的太难了，小生意赔的一塌糊涂，被银行催债，上有老下有小，孩子还有自闭症需要康复，爱人有肝病要养，我也想不到赚钱门路，有时候真觉得死了算了。偶尔也想放开，可一想到放开那我家老弱病残穷首当其冲，会活的更惨。所以我只恨，恨那些躺平的地方，一直拖累我们。 363. 不用其他国家，就香港作范例，棺材都不够用了 364. 宁可感染，也要吃饭？真放开共存了就是：既感染了，也没饭吃 365. 就那我生活的城市来说，只要一有疫情，立马口罩戴得严严实实的，外出吃饭的基本没有 366. 放开，大规模感染，不能上班，劳动力短缺，医院人满为患，医保枯竭，社会收入减少，社会支出剧增，社会发展停滞，百姓生活倒退。资本家有钱治病，普通人因病致贫。放开就是自杀。 367. 先让香港放开，我们观察观察 368. 现在谈放开的都是间谍！商业间谍也是间谍！ 369. 图片评论¡评论配图 370. 共存经济也不一定好，共存动乱的可能性很大 371. 放开有一个前提，那就是治疗费用全部自费。否则的话，面对暴涨的感染者大军，脆弱的医保非得垮了不行。 372. 病毒一直在变异，全面放开不是找死吗？ 373. 感染后家中抵抗力差的老人面临着死亡，因为到处感染，人心惶惶，人有的因为后遗症可能丧失劳动能力。这是疫情战争，想着能全身而退不受影响就是幼稚幻想。选择共克时艰，而不是放弃本可以保护的人的生命才是最应该的。 374. 感染了，就是另一种腔调了，哭天喊地满地打滚 375. 躺平国家的经济一定起飞了吧 376. 反正我的观点是无论你是什么措施都必须是有计划的，就算开放也得是按照步调落实好疫苗接种，隔离点到位，每个人做到每天自测，然后有序开放，而不是碰到一次严重的病毒就不管了被动开放 377. 他们是觉得自己放开了病毒就会跟他过家家吗？该发烧该住院不还得废在病床上？家里多厚的底子让他脑子有这种想法？ 378. 其实清零政策，才是对于经济影响最小的，可以对比一下中国GDP和外国的，还有中国各省的……（我记得国内控制最好的西藏，他们2020还是22年GDP增长是10%）。 379. 如果客观条件不变，开放共存，那我肯定不敢出门，非要上班，必须全套防护，绝对不在外面吃饭，下班就回家。想想去年我们西安封城前，人人恐慌，真开放，我的恐惧肯定比那时候更严重，毕竟那时候我知道大部分地方还是安全的。感觉开放了，那才真是真的要完。 380. 轻症和吃饭哪个重要？ 381. 哪里高了？？我并不想共存，求你别代表我，我就觉得国家这样清零真好 382. 转发微博 383. 我不赞同放开躺平，不过现在经济真的岌岌可危了。。。看看法拍房增加的数量，真的很可怕 384. 人就是记性差，忘了武汉那段时间怎么过的；如果真的放开了，等到时候新闻里每天都是几百上千万感染，医院里尸体多得处理不掉只能放在过道里或者病房里，同一波人又要我们反思为什么要放开了 385. 很多人幻想中开放的样子是回到从前，实际开放后的景象可能还不如现在…… 386. 迟早要放开。封了再封肯定受不了，小个体户真受不了。两害相权取其轻，疫苗，特效，卫生，刚开始慌乱，时间一长都会找到适应适当的办法。 387. 我也不明白为什么还会有人在散播一些新冠就是大号流感的言论，明明是需要严肃对待的事情到他们嘴里就轻飘飘的一句感冒一样 388. 喊开放的是不是以为他是天选之子，不会被感染？ 389. 不敢想象放开，放开之后的人生感觉的就是一轮接一轮的新冠反复感染，要么呆在家里不出门，被新冠一点点蚕食生命，活着可能真的就是等死 390. 你是指放开跟病毒共存的那个上海吗？不用你说我也不会去//@吐司边杀手:回复@李实Sebastian:希望你这辈子别来上海//@总是忘记用户名的小宅:说了很多遍，但我还是要说，所有吹全面放开的都可以去香港。有钱的去做生意，没钱的那边也招保安 391. 可是全面放开了，抵抗力弱的老人和小孩首当其冲会被感染。如果家里老的小的都病了，谁还有心思去上班赚钱呢 392. 放开是迟早的事了，这病毒消灭不了了。但放开必然是社会能承受病毒带来伤害的时候（每年疫苗的普及，特效药的完备等）不然谁都可能被时代的一粒沙给压垮。 393. 想共存的可以去外国啊 394. 他们总拿防控的成果去论证开放之后的经济，把所有的经济不景气全部归结于防控政策，从来不想想开放之后疫情怎么可能是现在这种状态 395. 我不会说其他……反正开放后、老子立刻转行殡葬业 396. 前两年的正确政策让我们处于非常有利的位置：护住国门的同时普遍接种疫苗，静候疫情结束或者病毒毒力足够减弱，而不用像国外那样用人命去填。但现在有一个紧迫的问题就是老年人疫苗接种不到位，如果不能及时解决，只怕这两年的努力要白费。 397. 天天叫着开放的人都不知道国外开放是什么样的，得了新冠在家躺着就能好吗？不能的，要打针，打抗生素的。去医院，人满为患，不是重症不能住院，医生给拿了药，回家自己打去，打好了就运气好，打不好就点背，转重症上呼吸机就能住院了。这就是国外开放的情况 398. 刚刚前几天还有朋友和我说，老米那边不隔离照样没事。反正我是不相信的。即使不隔离，自由了，谁敢轻易出门？那种zi由，是没有she会道德感的人的自you，是那些即使感染了到处乱窜的人的自由。而具有道德感的人，只会被殃及。 399. 是的，不是放松隔离政策经济就能全面恢复的 400. 真的放开，我也不敢到处跑 401. 肯定不行的，家里有老人，谁能保证没个闪失 402. 放开后自费治病，又会哭天喊地 403. 在没有明确病毒毒性不致死，没有后遗症的情况下，感觉全面放开只会让经济更差，本来目前防控政策下能去敢去的地方全都不敢去了，各种小微企业服务业人流量更少。 404. 反正坚持动态清零，这波疫情非常特殊，是内外配合的投毒攻击，非常严重，所以封的比较狠，舆论场上有人带节奏非常正常 405. 首先，你调查对象有问题，但凡能待家里的，起码是躺平了啥也不愁的，那些体力劳动者，房贷车贷还要还得人，谁愿意被封 406. 妹妹在乌克兰上学，刚回国，一宿舍所有人全都得新冠了，医院给拿了抗生素回来让他们自己打针，几个人互相打，都不敢下手的。如果国内开放也出现这种医疗资源跟不上的情况，那最好你家里有个人是做护士的。 407. 就我身边来看，大家对说这种话的人的态度就是，"你家身体力行先死三个给我们看看" 408. 说到影响吃饭的，我就问一句，像上海这样，现在要出差的谁敢接？国内大城市交流流动受影响不影响经济？ 409. 新冠和流感不具有可比性，单看致死率是耍流氓。第一季节性不可比。第二目前已知的脑损伤，生殖系统损伤不可比。第三传染率不可比，包括病毒生存力，传染百分比等等。所以说，单看致死率其他不考察，有意义么！炒作这话题的人不是蠢，是恶！ 410. 今天我校友群因为这个问题吵了几百来条，就差线下约架了。每个人的家庭状况确实不一样，但是吵着放开对我们一个人口庞大、医疗还在上升期的发展中国家来说绝对不会更好，只会更坏。看看隔壁的香港，再看看上海，躺平有用吗？哪一个不是付出了庞大的代价在疫情上。 411. 多少人都没饭吃了，谁还在乎自己的有没有得病，被饿死还是被病死，哪个几率大一点？ 412. 中国有句古话叫“不见棺材不落泪”。想不开可以，别带上我和我身边的人就行。别人要上天入地自己有本事闹去。 413. 香港放开了，结果都看到了 414. 非常支持国家防疫政策，但最“怀念”的是疫情20年的时候，大家真的团结抗疫，社区管理的不错，国家压着物价没怎么涨，店铺房租也减免了，线上办公也基本让按时下班收入没减，清零了就解封不搞一刀切大家都很开心。但现在有些事情真的越做越差了，不如20年 415. 我们放过病毒,病毒会放过我们吗 416. 香港已经宣布躺平了，看看那边的情况做个参考。 417. 就像博主调查的，真以为满是疫情，就可以出去做工，就可以饭店开业挣钱吗，我相信满是疫情的世界，大多数人还是不愿意去感染的。就算新冠不要你的命，你反复发烧也够人受的。 418. 如果放开了，我家人肯定不会外出了，尽量就宅家了 419. 这病毒如此反常反复变异，我心里就一直认定是美国生物研究所研发出来的产物，搞得全世界都不得好过，他们只研究病毒却没有研制出解药 420. 放开了也是卷王的天下。现在还没粮的躺平你就是太平间的一份子。根本没你什么事 421. 共产主义下，封闭国门都没问题，关键市场经济下封闭意味着没有了收入来源，西方的躺平完全可以理解。 422. 问题不是封不封的问题。很多人不希望如此保守的政策执行下去。不要说老百姓家里没有余粮。地主家也没有余粮了。昨天还嘲笑某站给你搞毕业的。现在是大厂都在裁员。小厂直接关门。按月给你发半薪都算是有良心的老板了。大家饿死索性躺平。蚂蚁也知道爬出洞就有被人踩死的危险。要死死一个不能死一窝。 423. 可是我好怕死，我是真的怕死，我觉得如果条件允许我选择多活几年 424. 那以后口罩会成为一种生活必需品，不带口罩生活的日子再也回不去了 425. 看看海外华人都在过什么日子吧！开放就能经济正常化？做梦吧！ 426. 其他不说，放开肯定新冠看病肯定没有国家托底，这些低收入的万一得个中症或重症，不要说赚钱了，看病的钱都没 427. 然而大众是听不进去这种声音的，因为媒体已经把新冠渲染成染上就会有很多后遗症很难治愈的疾病 428. 放开了不是共存，是共亡。不光是身体抵抗力低下的老年人和儿童会亡，青壮年也会亡。即使幸存下来的也会带着后遗症，甚至丧失劳动力，那就是灾难了 429. 我受不了天天戴口罩的日子了但是我怕死，我怕我在乎的人被疫情迫害，所以呼吁共存亡的S13自己先狗带好了，就不做慈善，不给任何人当替罪羊 430. 这几天感觉大家都越来越不耐烦了，对新冠也有点麻木的感觉了，去年的时候出了两三例路上都没人了，这几天每天都增长着都感觉不到大家紧张的感觉了 431. 苟利国家生死以，岂因祸福避趋之 432. 当年抗战内战前辈在那么艰苦的环境都熬下来了，现在我们作为后辈难道要比前人逊色？ 433. 别人我管不着，反正放开了的话我肯定能不出门就不出门了 434. 上海大多数人不想躺平，香港也不是完全躺平。建议去缅甸吧，那边已经躺平了。 435. 求他们看看有关后遗症研究的报道吧失去味觉，脑子萎缩变笨，我真的不想经历 436. 拉倒吧，他们是觉得病倒的肯定是别人不会是自己 437. 首先要知道世界回不到原来的样子了，开不开放人类都要重新开始新生活模式 438. 完全躺平对生产力影响是非常大的，可以看看躺平国家的GDP。 439. 全面开放后很快就会暴露很多香港现存的问题，员工确诊请病假同事要加班补缺口，人手短缺物流公司派送不及时，超市哄抢货品蔬菜水果价格成倍增长，普通医疗资源紧张私家医院坐地起价，香港这些问题没有严重到让城市瘫痪只能说因为我们背靠祖国这座大山，没有深圳伸来的援手，香港人连猪都宰不了。 440. 现在真的很疲惫 441. 疫情会像非典一样消失吗？好难挨。 442. 有个尼泊尔博主在尼泊尔感染三次新冠 443. 人类无法消灭病毒，只能与病毒共存！再说一遍：尊重科学尊重医学！ 444. 应该让每个人向政府报备一下，如果全面放开，自己是否愿意成为那1%。 445. 两年前欧美疫情第一波大爆发的时候某些人洗地就是这个调调，两年过去了v，隔壁南棒开放结果日日增30万，他们还是这个调调，呵呵 446. 放开之后，经济也不见得会好，甚至更糟 447. 我身体天生的免疫力差，我很害怕。我家里有九十多岁的奶奶，我也害怕。 448. 关键是躺平了，病毒肆意变异，万一搞个超级毒株出来了，年轻人可能还好，老人和孩子怎么办？呼吁放开的人有想过变异的病毒夺走家人生命吗？ 449. 先问要放开的人：你家准备死几个？ 450. 严格封城没错，但是某些地方随意撕开口子造成传播这损失太大 451. 私心是不想放开的，但这轮不到我说什么。但是就一个想法，如果分开以后治疗费用国家全包，那我同意放开。毕竟生死有命，但是因为生病治疗的无妄之灾而花多余的钱，我是问我不愿意的，宁愿多隔离几天我就是这么小市民 452. 犹太特效药加入医保上海沦陷外溢可不不共存谁买药。犹太医药这么特效怎么不救救美国人？ 453. 哈哈哈哈哈哈哈哈🐸和不明生物，笑死我了怎么这么可爱哈哈哈哈哈哈 454. 这个隔离的日子我要生活不下去了，得病了可以治好，房东催房租的时候我是真的拿不出来钱啊 455. 看到名字就知道是三体迷！我看了4遍了。对章北海印象太深了！ 456. 以中国的人口基数和医疗能力，尤其是二三线以下城市的医疗水平来讲，一旦放开共存，一是会造成大量死亡病例，哪怕致死率只有0.5%；第二以奥密克戎的传染力，海量病患涌入医院，会迅速冲垮医疗体系，以及医疗资源挤兑造成其他病患的伤亡，也是一个大的问题。所以我个人感觉真的很难，国情毕竟复杂。 457. 主要是病毒肯定会进一步变异的，它的发展趋势怎么样也不确定，放开风险有点大。 458. 居然还有想放开得病的。而且是不知道有没有后遗症，或者之后什么时候突然显现后遗症的。就是无法想象还有主动想被感染的。我年轻，我也不想冒这个险，谁知道会不会得了之后体力就跟不上了，记忆力减退，或者其他隐性的慢性的。 459. 主要还是节奏问题，不过！！！我提个意见，国家对于因为疫情，导致生活比较困难的家庭，个人。能否提供低息或者免息的“灾害帮助金”。因为确实有一个不小的群体，在疫情到来之前，可能就过着月光，甚至超前消费的生活。一旦停止正常的工作，这部份人是最难的。这部分人群的问题不应该被忽视。 460. 确实只要问放开派一个问题就可以了，放开后，死到他家头上，他愿不愿意 461. 因为它们探索出来的，经济也没变好 462. 我真的想夸夸北京的防疫，进京需要核酸，学生每周核酸，重点企业和服务行业定期核酸，小区隔一段时间来一次免费自愿核酸，这样即使有疫情也基本在两三天内就发现，然后小范围封控隔离，其他地方完全不受影响，我觉得这才是精准防控该有的样子 463. 我们有十四亿人，真的放开不要看看人口小的国家。看看隔壁印度就知道后果了。 464. 现在有统一的预防和诊断方案，没有统一的治疗方案。与病毒共存只会造成更多新问题。 465. 你说的很有道理，转疫情求生蔬菜包，仅此一份，看我微博。（我好像营销号哈哈）真的转 466. 完全赞同，中国至少大半还是很在意自家老人小孩的，只要有隐患的可能性，谁愿意去共存冒险呢。所以如果共存，社会活动照样不会活跃，照样不可能正常发展经济。 467. 一旦放开就没有回头路，所以一定要做好万全的准备，现在准备还没有做好。用来专门医治病人的方舱不够多，用来治疗得特效药不够成熟，用于针对奥密克戎的疫苗还没出来，新冠的死亡率也没有降到足够低，我的建议是再抗一年，同时把这些准备做好，做准备的同时也能创造一些就业岗位。 468. 三个月重复一次淘汰体弱多病的健康人能坚持几次 469. 真是有点太天真了。。。 470. 怎么样都回不到从前了吗？这该死的病毒 471. 你完全再说屁话 472. 有问题应该改善，但不是躺平！ 473. 赞同博主的想法没想到2022年了河里还有石头 474. 前几天确实高，现在少了一点 475. 哎，再防十年吧，病毒一时半会能减弱？ 476. 发出这些噪音的也可能有本地产的未知物种，是越来越多这种生物体在洗了 477. 上海本地人解决生活问题不大，外地打工的早就回家了吧？我猜主要是外地在上海买房的。 478. 同意 479. 再等等嘛，等四价疫苗上市。 480. 上海实验区 481. 我马拉西亚的同事也是感染了平时挺健壮的有很多女同事感染过都痊愈了但是他一下就没消息了都好担心🙏。只能大家把工作分了一下先。香港也居家办公了，实际只有大陆办公室运作。 482. 朋友圈已经有人开始造谣了 483. 如果是硅谷的人才，可以在家上班，影响不大，如果不是， 484. 其实就是积蓄的钱花光了，我的工作允许我居家办公，我和我父母也有房子可以住。家里别墅还有院子能种菜，别人我真管不了，物竞天择，疫情下失业的人我救不了也不想救，反正封禁对我有利，我就支持，我暂时也不会变成手停口停的人 485. 因为病毒没有突然消失，回不到从前的。这些声音大的其实也是想回到没有新冠的时间。。直接放开是恢复不了经济的，只会在恐惧中 486. 很多事是难两全其美的 487. 还全面开放？代价是什么？普通人就是代价，如今这个状态日子可能会难过点，收入会少点。全面开放丢的可是人命，之前的防疫措施长假还能去旅旅游，不是最近某地区搞得全国扩散，可能清明还能促进下消费 488. 把疫苗打打好，自我防护做做好，我还没结婚没孩子，不想绝后。 489. 谁支持全面放开4000+嗷🤗🤗 490. 又不是没有试点看看香港放开的后果再说 491. 如果全面放开，我能做的就是减少一切非必要消费，能躲就躲，绝不掺和，等差不多了再继续 492. 蛙岛医生已经预测蛙岛要走向韩国一样了 493. 基德有个视频就是讲这个的，开放是要考虑的，但肯定不是现在 494. 我觉得完全不用担心，其实国家完全可以成全他们，呼吁开放那些人，去登记，以后感染了自费。被他感染的人的治疗费用都由他出就好。 495. 影响经济的不是封锁，是疫情，只要已经在哪怕躺平，大部分人还是不敢出来经济好不了，至于生死看淡，死了就算的，呵呵 496. 有个问题就是想放开的你需要考虑好放开之后的后果而不能只看相对的人身自由。 497. 千万不能躺平 498. 说的非常好，国外躺平了，但是公司三天两头有人感染，没有人全勤到岗上班，大家都很恐慌，三天两头测核酸 499. 明明好好防疫就能解决的事。偏偏国外把摆烂偷换成共存就没人谴责了。甚至还有舔狗。摆脱让你们共存就是放弃你们了明白吗？积极防疫才是为了孩子为了老人为了所有人的生命着想 500. 这新冠，不是让人病死，就是饿死啊 501. 一打开微博，全是上海的求助信息。吓了我一跳！结果一打开热搜，哎呀！这是上海防疫的成绩 502. #疫情中的长春#看看吉林吧 503. 疫苗没覆盖95以上想都不要想。老年人接种率不够死亡率根本压不住，何况人均医疗资源如此堪忧，发生挤兑之后。。 504. 中国人均医疗资源太少肯定不能全面放开啊。 505. 你头脑太简单了 506. 说放开的有很多是香港的，看IP就知道 507. 坚决不放开，说放开的我觉得你别有用心，说不定是间谍 508. 怎么做都是错上海这一把还没完全放开呢一片混乱还一片骂声 509. 你到底从哪里感受到了第一句话我就想问 510. 疫情是认知问题了，咱们对新冠的恐惧来自于媒体的宣传。如果想改变，先从对病毒的认知开始。否则我们自己就愿意待在家里。 511. 🐸岛和境外不明生物， 512. 这种帖子，是了解过多少国家啊，其他国家马上连口罩都不用戴，国际旅行都不需要强制疫苗了。我们只希望谁阳谁隔离，不要封其他人，不要封小区，封城要求很过分吗 513. 谁放开谁就是蠢坏。哪个单位敢用一个阳性员工？医疗挤兑，老人孩子怎么办？这帮人缺大德！ 514. 其他国家…不是已经开放了吗 515. 说共存的，我就想说QTM，谁这么说，先无防护扔新冠病房，我朋友在香港被感染，这都一个半月了，还胸口疼 516. 比起疫情，我更怕防疫 517. 开放是迟早的只是结合国内国情需要缓慢进行而已 518. 现在无症状感染很多，应该是疫苗起作用了，如果说大家都积极打完三针疫苗，感染也只是无症状或轻症状感染的话，那新冠是不是和普通感冒一样了？共存也不是不可以了 519. 喊放开的也就在网上喊喊罢了，无能狂怒，你看现实中愿意放开的有多少 520. 长春现在的确就是吃饭要紧，很多人真的要饿死了，没阳要先饿死了，不是比喻，是真的快饿死了，老人跑出去哭着求超市，有人一顿泡面都舍不得吃，还有四处求助的，没人管的，还有建方舱不给钱，爆阳跑的哪儿都是的。 521. 我只希望，不要出现有急救的病人辗转医院最后因得不到及时救治而去世。也不要出现防疫人员随意进入人家乱棍打死宠物。疫情下的这些事件比疫情更加让人害怕 522. 想象全面放开躺平，医院人满为患，老人孩子紧接着中年人年轻人感染...一天几十万甚至百万的增长，死亡率上升，犯罪率上升，那是怎样的人间炼狱...真的不敢想 523. 共存这概念本来就是伪的，这是传染病是需要治疗的疾病，即使流感也要重视的，但那些人却以为躺平共存就以为经济可以复苏，啥事都可以为所欲为，而政府管控是限制他们生存，逻辑上就是错误的却在上海得到认同，可怜。 524. 反对全面放开我家有老人是免息系统疾病无法打疫苗（是疫苗禁忌症患者）如果躺平基本上生存困难 525. 是是全世界都是错的，你是对的 526. 并没有要全面放开呀，国家现在也正在探索逐步放开，所以也只能在上海这种各方面都完善的城市。这种事情不会搞一刀切的。 527. 那些叫开放的想什么呢，是身边没有老人和孩子对吧，如果开放了医疗资源肯定崩溃，治安暴乱，我就不信经济还能稳定，人口基数不一样到时候控制不了了穷人受苦可别又怪国家了 528. 其他国家的人不都基本探完路了吗 529. 要饿死的时候还呆在家减少外出？？？？ 530. 不是隔离的反对面就是完全放开，中国肯定得有自己的一套，我们的国情也决定了不可能完全放开，现在是需要探索一条路如何跟病毒共存。 531. 中国出资200亿美元请世界先进企业为中国研发一个最强疫苗，很多国家躺平了但是我们不服。 532. 把老人孩子隔离起来保护好，年轻人与病毒共存出来工作 533. 嗨呀，国家直接说，感染的按照医疗系统的承载能力抽签50万一个人自费治疗，你问问谁喊放开？ 534. 終有一天要放开，这病毒消灭不了 535. 反正我没觉得要共存，必须是它死，死不了也要等到有特效药再说，这万一是传染病，死不了也挺麻烦的，如果宣布共存，就会有很多不自觉的人得了也要出来上班上学，反正就是有些不清白的会害人，大概就是这些喊着要共存的人！随便这些人去哪，别回来就行 536. @Leo-爱雪 537. 辉瑞已纳入医保，有些不明生物已经急不可待... 538. 热度给我上去！！让每个来看这个热搜的人都先看到博主的这段话！！ 539. 应该首先了解一下这个病现在到底致死率是怎样？感冒也传染也能死人……可是没见过国家为感冒兴师动众啊！人类应该接受大自然的优胜劣汰，一味的过度的躲开绕道，还不如勇敢的冲上去，我同意开放 540. 一切的一切还是因为美国病毒 541. 我觉得根据病毒的属性慢慢的调整政策当然执行人一定要懂这个的不要外行领导内行的闹剧 542. 别人不是已经探过路了么 543. 放开了，之后呢？新冠得后的并发症，对身体机能的各种影响能忽视掉吗？现在在好好防护的前提下，该做核酸做核酸，该防护防护，总有一天会有对策的，至少能把新冠并发症的影响降到最低再说吧！ 544. 中国人口基数多大？能跟欧美比吗？如果全面放开，家里有小孩的真的希望孩子生活在随时可能被感染的环境中？家里有老人的，真的不担心会有并发症而撑不过去吗！？美国放开了，代价是什么？死亡奔100万！中国人口是美国几倍？全面放开要死多少人？也许你会说，你看看这次奥密克戎，美国又死亡了多少？ 545. 还有些虽然发的是汉子，但后面不一定是我们中国人。 546. 可千万别放开，我姐在加拿大，她们全家四口除了我爸妈都被感染了 547. 既然投票大多数人回答是:待在家里，减少外出。那么多人愿意待家里就呆家好了，减少了不必要的流动。而那些急切需要外出的也不会因为管控焦头烂额，现在因为疫情，有一点点风声，三四线城市一下的都封个死死的，哪都不能去，整个的有期徒刑。 548. 放开是必然的。就如当年的改革开放。 549. 就说一点，放开之后，假如你阳性了，你猜你公司还要你吗，哪个单位招你 550. 我不想全面放开，因为我有可爱的孩子，我想要他健康快乐的长大，而不是时不时看着同学老师亲戚朋友有人因为疫情而短暂的离开！ 551. 那么你第一句话的根据在哪里不是文章越长越有说服力 552. 数据说话吧不清零我们的人口基数按照目前最低的死亡率也得死40万按香港的死亡率也得死80万你觉得呢 553. 有一些人以为放开后，TA会是个站在抵抗力顶端的幸运者，事实上，大部分人到时就是在网上发布求助信息“阳性了但没医院接收没床位蹭不上医辽的”弱者。此外，事实胜于雄辩，HK和上海已经从实践上证明全放开和半放开都不行，最后还大面积连累其他地方，还需要吆喝着要支援。 554. 抱歉我没有听到这样的呼声 555. 关键是一旦放开，感染之后国家不会出钱给你治了，没钱就一起等死吧 556. 不能开放 557. 说得真好 558. 虽然，但是，可是，护士医生好累哇 559. 那些宁可感染也要吃饭的人，拜托去吃牢饭吧！ 560. 多的我就不说了，我儿子十三个月又不能打疫苗，感染了怎么搞？之前有个三个月宝宝感染了，重症，我因为哺乳也没有打疫苗，生完孩子身体素质大不如前，我是怕感染怕开放的。 561. 放开就是美国的样子，几百万的死亡， 562. 共存就以为好了吗？三天两头感染生病去医院耽误工作，家人被传染去医院，治疗还得花钱，家里老人随时可能顶不住，估计到时候更加觉得疲于奔命 563. 看到上海突然不敢了上海的医疗资源在全国来说肯定是第一梯队的他们的医疗系统都顶不住更何况我们在疫苗接种达标特效药什么的出来之前我们还是老老实实防控吧有病不能医发生在现在我简直觉得太可怕了 564. 又害怕又期待终究是回不到过去了 565. 奥密克戎传染力这么厉害，这次全封检测完，人口流动能停止吗？能禁止港澳台和海外人士入境吗？总不可能出现一次全封一次，总还是要想最终解决办法的，比如奥密克戎的后遗症是什么？有没有特效药可以医治？不能一说共存就是敌对人士，而是应该理性探讨可能性，不是说共存就是立刻放开，而是找到解决办法 566. 说的有道理 567. 火化场到时候都不够用吧，印度的例子摆在那里 568. 如果放开了打死我都不生孩子 569. 那些躺平的国家借助疫情“去老龄化”在它们眼里，不能给社会创造价值的人不如狗，而中国传统文化比较重要的核心之一就是尊老爱幼，所以它们眼馋却没办法只能疯狂抹黑 570. 在疫苗接种率没有达到一定强度之前放开就是找死。 571. 我想问一下有哪个躺平国家经济比中国恢复的好的？ 572. 不现实的，医疗系统承载不住的 573. 所以在经济和安全里面尝试找平衡啊，谁家里没个老人小孩，至少我不想冒这个风险，就目前这个传染性和症状而言开放还是太危险了，医疗系统崩溃怎么办 574. 有钱的当然要开放了，他们又不愁没钱没病房治，要是中国待不下去最多拍拍屁股走人嘛，那些没钱的只能留在国内的还在叫我就想不通了 575. 可以参考印度，因为人数和居住空间只有中国和印度的比较接近，其他的国家无法参考，毕竟别的国家人口数字和居住空间都很大，可以做到独自隔离，中国没法跟人家比。 576. 新冠对家里一些有心脑血管疾病的老人伤害太大，年轻人免疫力好是能扛过去，谁都不希望家里的老人出问题。 577. 因为现在疫苗国家免费给打得了免费给治房贷可是自己还的开放就自费治疗呗看还有多少支持开放的 578. 我国人口基数大，嘴上喊共存，死到谁家谁得骂政府不作为 579. 奥密克戎不一定是新冠的终极模式有些地区德尔塔+奥密克戎结合的毒株，加上美国死100万人，其实不需要在中国土地上实验了，近距离差不多的毒株，香港就是最好的例子。而且上海影响的仅仅是自己吗？其他城市的防疫和医疗也要被挤兑，去支援上海。希望以后还是劲量实事求是做防疫。 580. 说共存的，是要牺牲很大一批人的，目前我们只有尽力做好防控等特效药。这个牺牲要有多大要用数据说话，具体可以看下b站上的【基德】—用数据说话分析的很到位 581. 国情真的不同，咱们14亿人口啊，真要放开了，大面积中招，医院都塞满了，而国外很多人说就跟感冒一样，能一样吗？你根本没办法保证自己是轻型还是重症的那个，我是不敢冒这个险。 582. 主要是我们国家人口多！就算打了90%的人！剩下的也是1.4亿！这1.4亿基本就是3岁以下的儿童和有基础疾病的老年人！想想他们要是感染了就算没有肺部感染就发烧都够国家医疗资源挤兑！ 583. 回复@风湿科聂医生:？您先给大家做个案例分析吧，就需要您这样的临床个案！ 584. 没错，不要放开是对的！ 585. 上海太自私了吧，政府就这么作为？ 586. 国情不一样的，就我们国家这个人口密度，全国有多少人是那种郊外独栋别墅的？城市全是垒的几十个几百个楼挤在一起，一个感染不控制很快全国到处都是，到时候就等死吧。 587. 我不赞同放开，人是自私的，我承认。我有外公外婆，还有孩子，如放开，我这十八线县城会被波及。现在疫情之下，至少我这不受影响。当然，我也理解放开的人，国家或许可以考虑出台签署自愿放弃新冠治疗回家自愈承诺书，避免挤兑医疗资源，自费医治新冠。同时由传染者给予我们这种不幸被传染的人一定补偿 588. 现在最主要的老人和小孩其他都好说 589. 不放开，你过吧！你看看你的一生有几个3年 590. 没有呼吁全面开放！那些都是水军！！ 591. 放开之后，自费医疗。 592. 所言极是，煽动开放的肯定都是那些五十万，扰乱人们情绪，制造矛盾！ 593. 开放了，危险最大的还是老年人和有基础病的人，我不想赚太多钱，我就想自己和家人平安，至少现在不用提心吊胆 594. 早日研究出特效药吧 595. 其实我们的现有政策就是在抢时间让防疫手段更好一些让病毒毒性更轻一些 596. 中国一旦选择躺平共存，国外舆论立刻态度三百六十度转变，忘了武汉封城的时候国外媒体是怎么报道的了嘛。外国巴不得现在立刻来个超级加倍版的变异毒株击穿中国的防疫体系，至于他们，你觉得西方和漂亮国在意它们的底层百姓吗，精英阶级巴不得清洗底层人口，因为有钱总是能治得好的 597. 拉倒吧，因为蔓延不控制，波及我们都没法正常，打工人难死了 598. 如果是章北海做选择的话会怎么样？ 599. 选择躺平的那些人就很自私，没有想过人家免疫力差的人吗？别以为自己免疫力强到时候自己被感染了，又要怪国家！ 600. 去年GDP不是增长8.1%吗？为什么要回到疫情前？ 601. 。 602. 开放躺平狗，他家老人孩子先进焚化炉。要是骂我那就一户口本进焚化炉。 603. 我单位附近有家烧烤店有过新冠，后来好了，出来重新开张，结果到现在都没什么人去，改了招牌也是一样没有任何起色！说共存的，想过没有，哪哪都是阳性病例了，谁敢去逛街消费，去看病？！ 604. 说新冠共存的人是想害死谁呢，国家几年防疫工作成笑话吗？ 605. 坚持动态清零，让别人先探探路。。。毕竟探路是以人命为代价的，有人帮忙探路，为什么不再等等呢？历史经验告诉我们，摸着别人的石头过河，是条不错的经验。。。 606. 如果大家能接受医疗击穿后自费医疗，另外不要无事呻吟开放应该没问题。 607. 动态的清零的本质就是牺牲少数利益局部利益以换取整体利益的最化。这个整体利益是全国是中华民族的整体，不是封了某个地方地球停止转动的某个地方。 608. 这个问题，就是抗美援朝的问题，打还是不打？！早打还是晚打？！施大仁政还是小仁政？！ 609. 想早点死的可以选择自己跳楼！不要等着被感染上新冠了连累周边一大堆！ 610. 现在每天还会出去吃饭和旅游，真要放开与病毒共存了，除了上班我肯定不会去任何地方，要为家人和身边的人负责，也要为自己的健康和工作负责 611. 就是，急什么，全民疫苗了吗 612. 黎明前的黑暗 613. 说是这样说，真全面开放，不用多久。大家都出来了 614. 疫情严重的地方隔离，没有疫情的地方全民做好防疫措施，这种一刀切肯定不合理，毕竟人都要生活的，无法工作还怎么生活， 615. 个人看法。这个玩意儿对于有基础病的老年人太不友好了。虽然现在的症状都比较轻，但是中国的老年人数量庞大，如果全面放开躺平，那么对于老年人是有很大的风险的，从而会不会到时候大范围的老年人感染的话，医疗挤兑出现的了，我们国家的医疗体系能够充分应对这种情况吗？ 616. 放开与不放开都很难啊，放开大家都会提心吊胆，一旦感染就会有人说闲话，不放开就没法生活了，就像现在潍坊这边一封控，老百姓菜都卖不出去，物流园里的的菜拉不出来，最终经济损失还是老百姓自己承担 617. 放开可以等国产特效药上市后慢慢放开。现在肯定还不是时候。而且我们不能依赖辉瑞芯片的教训历历在目不能重蹈覆辙 618. 笑死，开放了就更加困难了，你每天疑神疑鬼的，我们成年人不怕，那些新生儿呢！那些孩子呢！撑得住没完没了的发烧吗？要死了 619. 瞎几把扯微博上有人bb两句就成呼声很高了？？？ 620. 所以那些喊共存的傻缺根本就没深入考虑，张嘴就来。 621. 提出和新冠共存都是自私自利的人，因为厌烦了没完没了的新冠检测，就希望和病毒共存，就算得病了，也要医疗团队最好的治疗他们 622. 开现在不能开，但是保障做好了啊 623. 那是还没有全面放开，一声令下全面放开早跑起来了。 624. 共存最大的问题是致死率，香港目前死亡率0.5%，假设死亡率我们控制在千分之一，也就是说14亿人口要死亡140万人口，而且我们这几年老龄化多，医疗条件平均水平比国外差，请问这几百万人命谁来负责？ 625. 🙏🙏该喷还是喷的，就是不要让🐸乱搞 626. 那些开放疫情还选择在家里的只能说不够穷 627. 唉，我们公司已经3个月没有发工资了，源头是zf没钱付我们工程款，也没钱搞基建，大家大眼瞪小眼，看看谁先熬不下去。还有今年大多互联网公司裁员的信息漫天飞舞，人心惶惶，唉。。。难啊！难！ 628. 🐸岛是啥意思，一直没明白 629. 啊？我还是比较怕死的我不想与他共存 630. 反对共存最后还是要共存，这个病毒根本消灭不掉，而且现在上海吉林的情况已经很难做到清零了，理想很丰满，现实很骨感，寻求更科学损害更小的共存策略才是目前急需解决的 631. 支持动态清零，坚决不共存。谁家没有老人孩子，共存了，他们最危险，最容易重症，甚至失去生命。我宁可现在这样难受，也绝不想失去任何一个亲人。 632. 有能力的话就绝对不选共存，咱们是地球上唯一还能对抗这个病毒的希望了，都沦陷了就是黑暗时代真正降临的时刻了。 633. 不可能共存的，绝不能共存 634. 喜欢严控抗疫的可以去看看长春的词条，也可以过去骂他们 635. 五千年的王朝更迭，我党怎么可能放开！ 636. 说放开的人都无脑 637. 我就想知道，放开后万一感染了？还会给大家免费治疗吗？要是不免费，到时又会有一大帮人出来说国家不好，治这病还要钱。要是免费，凭啥？不是你们要求躺平开放的吗？到时医院让用神药，确定大部分人用得起吗？到时只会更乱！相信网红之流的资本家，就等死吧！ 638. 有一个不知道夸不夸张的想法先勿喷，如西方躺平，得个轮回，全是弱智脑痿缩，咱们复兴之路会不会更顺畅呢 639. 日本奥密克戎的死亡率是千分之一点三，如果放开，按中国的人口基数和密度，死亡人数很快就会变成每天一架737坠毁。如果放开是zf的无奈之举，那我照样接受，但是像你们这些公知跳出来说这种话，我只能理解为没人性。你真的没有资格和立场来说这种话。 640. 如果新冠治疗费用需要个人承担，还有这么多认同是大号流感，应该躺平共存吗？ 641. 全面放开那之前国家做的努力医务人员的付出就白费了，国外的路适合咱们吗？任由疫情扩散，自然免疫?国家的的经济能恢复吗？现在新冠治疗费用包括新冠疫苗全部国家承担，一个新冠患者起码也得几万吧，重型患者也得十几万，国外是怎么做的呢，有钱就治没钱就等死……这就是所谓的自然免疫 642. 探路🉐時間需要多久？我們還能等到嗎？ 643. 我可不想共存🙏求祖国别放弃 644. 支持放开 645. 放开之后的待在家里，和现在被迫待在家里是两种不同的性质，现是剥夺了想选择的权力 646. 不想放开虽然不会死但会有后遗症 647. 全面放开？14亿人，还有很多医疗条件很差的农村，怎么放开？永远都回不去了。。。 648. 宁可感染也要吃饭。这逻辑不对吧，重灾区，感染几率90％，你感染了更没饭吃啊。 649. 不是涨到三万了吗//@你比从前快乐117:对，香港现在招护工，去养老院照顾濒死的老人，每个月两万多，不怕感染的，觉得自己可以扛过去的，赶紧过去挣钱//@总是忘记用户名的小宅:说了很多遍，但我还是要说，所有吹全面放开的都可以去香港。有钱的去做生意，没钱的那边也招保安 650. ……其实疫情封城期间暂停各种贷款费用缴纳，基本生活物资例如馒头跟水可以分配到手，免疫情封城房租水电气，那百分之90的人都不会再提开放配合封城会更积极……就这么着吧现在……无论什么政策我个人都服从支持拥护 651. 超级赞同而且国情不一样咱们多少人什么医疗设施欧洲多少人什么医疗设施 652. //@苍蝇的左大腿:感染后屌会变得很小，真的有人愿意吗？ 653. 完全放开在拆那确实不合适，但某些城市过度防疫，某些城市过度自由，只能说拆那太大了 654. 不能放开，我家里有老人我害怕 655. enmm假如...即使和新冠共存也需要时间融合吧，自然选择、优胜劣汰？ 656. 共存真的很可怕….如果共存那在打工人天天要睡公司睡工厂太可怕了…. 657. 先不说共存与否这件事，先聊上海这次疫情防控的反应，行政系统看起来从上到下忙成一锅粥，然鹅忙了个寂寞。老百姓看病得不到救助，封控居民吃饭得不到保障，应急预案约等于0，大方向既错误又混乱，那些甩手的基层组织，职业素养不知道在哪？这就是你上海的行政体系该拿的出手的答卷？不丢人？ 658. 不要讨论躺平！美国替我们试验过了：躺平死亡率很高！美国死了一百万了！还不算后遗症！！上海不是不封控吗？每天新增两三千例！！现在上海封控了吧？！所以我们国家“人民生命至上动态清零”政策十分正确，不用讨论！！ 659. 我真的，真心实意想说一句，rnm新冠！ 660. 无解 661. 借个热搜哈麻烦进入#疫情中的长春#看看2022这迷幻城市 662. 生活不是二极管，至少在那些低风险区，学校就别封了吧！！清明节都要关在学校吗？ 663. 恢不恢复以前的经济跟咱们打工人没关系，只希望别动不动就封，上不了班就赚不了钱。 664. 防疫成本已经远远超出疫情本身带来的损失。 665. 不可能，很多普通城市承受不了 666. 我宁可饿死顿顿泡面也不感染 667. //@ALIENrei:以为放开后待在家里就没事的显然是太天真了。我在新西兰，自从政府躺平以后各行各业都出现了劳动力短缺的情况，结果就是超市商品缺货，点快餐没人送，网购快一个月才收到（快递病倒了一堆），厨师要隔离餐厅只能关门……同事一个个病倒所以我要做两个人的活 668. 之前看到以为医生分析，说现在不能开放因为我国人口密度实在太大，如果放肆感染会一时间医疗机构人满为患全部瘫痪，谁都不敢保证会面临什么。感觉政策还是要因国家而异，这个时候还是要相信国家支持国家 669. 嗯 670. 不可能放开，看看香港的死亡比例，一旦放开，医疗资源根本跟不上，人人自危，认清现实，对待疫情只有精准快，除非有一劳永逸的方法 671. 太天真了，放开只会情况更差，医疗资源绝对超级紧张 672. 还有香港的 673. 感染后，变笨，失去嗅觉，失去味觉，性无能，吃的不香，睡的不香，还老干蠢事，总给自己和别人添麻烦，这就是共存后的后遗症，谁要？反正我不想！ 674. 求求了，祈祷千万不要开放🙏🙏🙏 675. 99.9%的中国人都不希望放开，只有有心人 676. 我觉得现在的情况就是不说话，等待，配合。虽然很烦。但是还是要坚持下去 677. 现在已经是最好的了，收入是下降而不是为0。🙉一旦放开，我自己就不会出门消费了，上班下班，也不会欢迎别人来我家了，代价承受不起。 678. 不放开，只会饿几天。放开了，可能就真要饿死了。 679. 只有中国才是真正能做到人民的生命高于一切 680. 现在回家的政策还是动态清零支持 681. 放开吗？可以啊，放开了你感染后单位让不让你上班那是你的问题，治疗费用自费 682. 就算放开，也是没钱赚 683. 我非常赞同，今天已经看到一位上海妈妈两个月大娃娃确诊后发烧不知道送到哪里，以上海的资源尚且如此，何况其他地方？放开后，在没有强制要求统一管理的情况下，就等于马上全民感染。医疗资源会不会更紧张？社会环境会不会更焦虑，有那么多国家正在经历，我们可以选择慢一点，这事有什么着急的呢。 684. 西方高福利，不上班拿的钱也能过，出来上班容易被感染，干脆待着了，导致劳动力减少，通货膨胀，加上俄乌战争，天然气，石油，粮食价格爆涨，看他们还能撑多久 685. 哪就非常高啊？宁可感染也要吃饭你真让他感染你看看他敢不敢 686. 首要的是消除国民的恐惧症！ 687. 那些可以加强防疫的东西，吸入疫苗或口服药好像一直说说而已，这些普及的话保障就更好吧？ 688. 没饭吃的嚷嚷的响，是因为躺在ICU的没法玩手机。到时候得病住不上院哭天喊地怨zf不作为的，还是这些人。 689. 泰国儿童医院都没有床位好多小孩感染新冠我老师的小孩跑了三家医院最后找了最贵的有个床位三天花了折合17500人民币 690. 躺平放开的国家，都是没有办法防控又不想花钱去防控的国家。 691. 我要还房贷啊，真的 692. 真要放开，自费治疗，看谁还支持 693. 关键现在某些地区领导拿着鸡毛当令箭，态度相当恶劣，无风险地区封路不让过，回家绕5公里。这个事儿也不能做过了吧，经济什么的我一个小人物理解不了，但我不工作马上面临的就是没钱还房贷，信用卡，吃饭都成问题!!! 694. 城市套路深，我要回农村 695. 这个问题是个宣传口径的问题和信息不对称的问题，普通老百姓看不到真相，国家认真决定就好了 696. 没办法，感染了你不一定死，没饭吃却一定会死 697. 我觉得你说的太好了 698. 回复@苍蝇的左大腿:短3后也有15，也还可以接受 699. 上海在趟雷不是么…… 700. 开放？我不想三天两头生病 701. 很多人以为完全放开就是大家一起掩耳盗铃，当没事人一样工作生活，以为不用戴口罩，不用核酸不用隔离，生活一切回到从前，只能说太年轻了。你不会以为美国零元购假装医护人员喊你做核酸，然后入室抢劫是闹着玩的吧。你不会以为你的邻居同事小商贩对待阳性的你和阴性时一样吧。 702. 别开，不开已经这么难了，开了以我国人口基数岂不是要养蛊 703. 欧美都是傻子，我们最聪明 704. 等别国共存成功再说，家里有老有小的赌不起 705. 没放开上海都这么了，放开了还得了？ 706. 唉，两难。那么我猜想会不会这样，在某一天，新冠病毒变种了N次之后，国外与之共存，然后把它变得对人体无害了，而我们由于坚持清零，到那时候国外再也传不进来有害新冠病毒，然后世界就变好了，口罩可以脱掉了 707. 国外群众是不是对病死1个人毫无关心，但可以肯定国内病死一个人，全国一定会群情激愤的 708. 赞同你 709. 我觉得是好了伤疤忘了疼，现在的放开不过是仗着国家兜底，国外是放开了，可国外核酸检测和治病可都是要自己花钱的，14亿人每人感染个两三次真当国家不会坐吃山空呢，真兜不住了，让自己花钱治病的时候，可以看看结局如何 710. 共存了经济也不一定能好，起码现在我还敢出去吃饭，能流动的大部分人是安全的，要放开了我是不太敢出去吃了，谁都有可能是小阳人啊 711. 回复@prickly栗子c:[雪容融]就会被人骂你不要吃饭的哇你不要工作上学考试的哇 712. 我们人口基数大，要是放开，医疗压力不敢想象 713. 搞笑了谁想共存最好让他感染然后半年后看看肺部CT看看自己的小弟弟有没有变小 714. 只能说这个想法在国内来说太理想化了。看看这次上海外溢那些城市的反应就知道根本行不通的。 715. 思路清晰，有些人被外部势力洗脑，总觉得放开了就自由了，其实这些人心里面压根不觉得自己会得新冠，但是你怎么知道你就不会是那个1%死亡率里的分子呢？ 716. 想要放开，先看hk。内地大部分地区的医疗条件还达不到hk的水平吧……大城市人口密集，传染率更高，然后流动到二三线城市，那里医疗跟不上，放开之后死亡率就会更高 717. 要求开放的不是蠢就是坏 718. 我不要感染，但真的要吃饭！ 719. 这显然不能代表所有人，我生活的地方不仅和病毒共存，大家也都正常上学上班出门购物逛街，马路上多得是不戴口罩的，包括老年人也这样，根本没什么可怕的 720. 放开的话医疗系统肯定最先崩溃，我们平民更加看不起病了 721. 全面放开不就是等于跟病毒友好相处吗，早晚会感染，这不就是自欺欺人吗。想开放的去韩国美国吧 722. 外国很多国家已经放开了，撤销了很多防疫政策，也没啥事啊…… 723. 英国法国德国韩国人口基本都五六千万左右，每天死亡200左右，中国人口14亿，放开以后大概每天死亡4000人，如果你可以接受每天新冠死亡4000人，可以接受自己感染以后不一定能够及时就医。 724. 如果全面放开，新冠的治疗必然会是自费的，国家不可能再承担这个巨大的成本。 725. 这个说的很客观了。其实就算共存，一下放开也是不可行的，我们的基础（比如疫苗普及率等）根本还不够。现在让人不舒服的其实是（划重点）“某些”“极少数人”的过度防疫，以及真的有些人在面临着生存和生计问题，所以会逐渐出现支持共存的声音。有不同的声音，这个世界才是鲜活有生机的。 726. 国外到是放开了，经济涨了吗 727. 我们现在比共存强太多了 728. 少来了，人家@小儿外科裴医生早就做了这个统计了窃取人家的革命果实在这博流量 729. 主要还是对病毒的宣传问题。最近比较多的宣传是毒性弱而传染性强，这么一对比下来清零政策明显是成本高而收效低。再加上身边的同学出了国也并没有怎么样，其他国家全民免疫确实付出了不少代价，但目前来看结果是好的 730. 国外早就开路了 731. 去你的猴子🐵 732. 讲个笑话，信不信只要宣布共存后一切费用患者自觉掏腰包，这些共存派的声音马上就消失了 733. 既然鼓吹共存论的不少人是以经济和生活为理由，那么这就是一笔经济账，那就按科学的成本预测来说话，全面放开与病毒共存，会有多少人尤其是免疫力弱的老年人和儿童会因此殒命，住院人数急剧升高后准备如何应对医疗资源挤兑（别说美国，美国是以医疗收费来淘汰底层，没钱的底层得了新冠就是等死） 734. 迟早放开，但不是现在，起码也要全面接种第三针疫苗，或者第四针第五针之后，让病毒的伤害性减到最小，不放开就等于永远清零，清零就等于消灭新冠，还是有点难度的。 735. 首先医疗系统先饱和运转春秋季的东北医院没多少叫嚣共存的人去过吧？都是你爸你妈住院你不陪着的？？其次医疗财政系统再崩现在得病的报销都是要账期周转多几个1%都是系统性风险最后国家还管着就听话点吧真共存了多数受苦的就是无能为力的老百姓 736. 防控的影响有多大不能和疫情前比，要和不防控都是病例比。 737. 我个人看法：三针疫苗普及率80%以上，而且老年人普及率90%以上才可以讨论放开；而且放开前三个月有必要把疫苗变成无严重禁忌症（高血压啥的不算）一律强制接种。 738. 共存的后面就是国家放弃现在新冠免费检测免费治疗的政策，然后多少中重症治疗完家里也差不多见底了 739. 按美国的死亡人数约100万，认可基数约30000万，比列是0.333%，乘以中国人口140000万，要去世466万人，还不算后遗症。谁也不能保证自己不是其中之一。 740. 共存要看看我们每个家庭能不能承担后果，很多人认为得了就是一个简单的感冒症状，这个传说的症状是不是所有人都这样，还有很多老人孩子还没有注射加强针她们该怎么办，大家还是冷静想清楚吧，别人家干什么我们就干什么！用脑子好好想想再嚷嚷！ 741. 共存不了，共亡就有。谁也保证不了病毒不再发生变异，有可能下一次变异势头更强劲，那又是新一波疫情。所有国外鼓吹共存的是因为他们防疫做的差才会无奈出此下策。 742. 主要政府考虑的是老年人很多人没有打疫苗，打了的也不见得扛得住，政府真的很为人民着想了，我们这边鼓励老人打疫苗都到倒贴钱的程度了，依然很多人不打。 743. 天天喊着说哎呀数据表明重症和致死性嚯，比感冒还低哦。马勒戈壁，哪个感冒的传染率会有这么高？一屋子一办公室一楼栋的传染的？关键是还不断的在变种。今天是奥密克戎，明天呢？ 744. 短时间的有饭吃，结果是最终大家都被饿死。 745. 只有全民努力，一到二周清零。清零后大家才敢出门工作和消费。 746. 办法很简单啊，严控可以啊，中国人很听话的，发钱就行 747. 看新加坡 748. 恕我直言，那些疫情就吃饭困难的，群体免疫了也不一定会好，我们这有封控期间卖菜挣了几十万的 749. 放开就等于有人会来你店里消费？放开就等于不管制？放开去医院有床位吗？如果都不是，那一定就是没死到自家人闲得慌的 750. 人都是趋利避害的，就算彻底放开，没几个人会放弃防疫的，减少外出，减少不必要的交流，这些本身就是对经济最大的抑制，还不如现在这样，能够最大范围的复工复产 751. 現階段還是支持動態清零 752. 病毒会变异，谁会愿意共存，我觉得还是安全健康第一吧，仅是个人观点 753. 疫情不过是经济下行的借口罢了，其实没疫情中小企业也不好过，国家财税政策一直偏紧，房地产熄火，国际环境不好，科技发展进去瓶颈期，这些才是主因。疫情结束会好只不过是个念想。给人个希望，其实没有疫情人们会过更难，因为没有希望了。 754. 现在到没到放开的时候？医疗能不能保证？国家还免费治疗不？如果不免费，我能不能挣到药费？ 755. 如果全面开放能拉动经济，国外也不会那么多国家经济倒退了…… 756. 现在严重的也就是吉林上海，别的新增基本就是个位数，吉林上海控制好了，那些就不怕啦。 757. 我的观点是：如果不能彻底消灭新冠病毒，共存是早晚的，只不过应该有前提，比如：病情不重、预后好、后遗症清楚、有特效药、特效疫苗等等 758. 这玩意关键会反复感染的，看看欧洲，一波未平一波又起。 759. 辉瑞要高价卖药给我们，共存使更多人感染，让我们不得不买，通过药物，来绑架中国，提出更多过分的贸易协定 760. 先看看新加坡的效果吧 761. 英国等国家全面开放是因为他们加强针已经60%覆盖了，而且是付出1/4的人感染的代价才开放的，中国可不行啊 762. 想不通，共存了，你觉得会比现在清零好？随便吃个饭，不小心就感染了 763. 看看吧！头脑清醒～～ 764. 我就是选择那个待在家里的人因为已经带了两个多月了，除了下楼买点吃的，基本没去哪什么意时候是个头啊！两年了 765. 这里是中国！！！ 766. 别的行业呼吁共存我理解，我特别不理解的是餐饮行业。尼玛这病毒会破坏人的嗅觉和味觉，让人丧失食欲。如果全民都感染这个病毒，餐饮行业这个行业都可以变成历史了。。反正吃啥都跟吃屎一样了。 767. 很多人，哪怕不是为了生计，都不愿意封闭，我邻居在隔离期间想方设法溜出去，就是想遛弯，因为是地头蛇，社区也不想法办，最后妥协就让他在小区里转悠 768. 我在西安经历了两次，可以封控，可以封城。但是要考虑到人民生活生存吧？尤其开小店养家糊口的，封了没有收入，店租照交不误，还有房租房贷一样要还，国家能出政策给暂停吗？能照顾吗？反正西安这两次是一点都没有，全部自己承担死活；封一到行政命令的事，封了之后的政策呢？有没有？ 769. 新冠专治头铁和不服，上海这一波还打不醒吗？犹犹豫豫一个月，霹雳手段才是菩萨心肠永不过时 770. 新冠不是感染一次就不会再感染，我还年轻可能可以扛得住一次，两次或者三次，但是我的爸妈爷爷奶奶已经上年纪了，一次可以，但是好几次都能抗住么，赌不起 771. 外面开放的经济真恢复的有哪个，越南工厂不还是崩了 772. 真，想死别拉我我绝对是我班上口罩戴的最严实的，每天戴只要出寝室去干啥都戴@小鹿努力暴富 773. 线下我没有听到身边任何一个人说这样的话 774. 放开了现在微博上哭着说上不了班看不了病没人救治的就是常态了，按我国的巨婴数量，基层光投诉电话都得打爆了 775. 我身边没发现有支持全面放开的人，不知道那些支持的是什么生物？ 776. 可以把要求放开的集体集中起来来进行躺平实验。地点选择新疆没人烟的地方就行了。 777. 3岁以下没注射疫苗的婴幼儿怎么办？ 778. 再精选的文字也挡不住冰冷的数字，请再务实一点🙏别再折腾老百姓。 779. 浦东限时3小时买菜挤爆各大菜市场，不就是大型感染现场吗，政府不知道怎么想的 780. 就这个吴凡前两天还在大谈上海封城对国际国内的影响呢，今天又谈封城的必要性，其实前两天上海的疫情数据已经高位运行了，吴凡们的预测研判能力也太差劲了吧，上海真正的防疫专家上哪去了？怎么不说话呀？ 781. 不是这两个词之外再弄点啥新词比较合适？局部和全面还有啥？ 782. 那么，这种状况的第一责任人是谁呢？谁主责？谁负连带责任？ 783. 我都不想喊上海加油了，希望上海好自为之吧，自己作的，前车之鉴有作业给你抄，你不抄，还嘴硬说自己是比任何人都好，不需要。那我还能说什么呢？ 784. 这哪里是全市散发？这已经散发到全国了好吗？ 785. 上海从一开始就错了！！！！！ 786. 简单粗暴的说，就是兜不住的意思吗？ 787. 上海这是在搞笑吗？，我们二线城市这边管制这么严，防疫这么规范，最后就被你们最发达城市管控不力搞成这样，一大堆病人入境我省给我们造成史无前例的灾难，你上海必须要付出责任 788. 感觉上海在很努力的减少对人们对生活影响，探索一刀切之外的方法，他失败了说明全国其他城市也没有能力做到，那以后只要有病例，直接封好了，我想这也不是全国人民希望看到的 789. 不就是全面爆发么，浦东昨晚临时开放小区，一窝蜂地往超市菜场跑，这种人员聚集想过后果么，各地来驰援也不见发个通稿，本上海人真的脸都丢光了 790. 早干嘛去了，一个月了，这么严重了，才开始管理 791. 躺平吧，毕竟你们崇拜的西方早就躺平了。 792. 看到上海心中还是蛮伤感和愤怒的。世上还是苦人多，人民要生活已成为最卑微的祈求。不知道领导的良心都到哪去了？可以感同身受的理解百姓，但是理解不了一个政府不为人民考虑，一切建立在人民群众生命之上的防控，这波操作上海政府太让人失望，上海加油！ 793. 还是上军令状把，搞不好领导下课吧。。 794. 上海的病例数字说明，之前精准防疫时攒下了大量的病例，病毒已广为传播，只是上海自己不知道而已。 795. 坐标上海，对上海这次防疫真是无语死了，不管你是个什么城市，疫情管控不住就是失败，快一个月了，一块一块的封的小区也不少，老百姓医护人员都没少遭罪，搞成这个样子都不觉得脸红💢 796. 一天3000多了还不算爆发呢？ 797. 还有个特点是外溢 798. 哦我都被封22天了你才发现这次疫情爆发了遮不住了啊 799. 全覆盖区域性打击，地毯式轰炸，差不多这意思 800. 搜下上海疾控中心和辉瑞，就知道上海的精准防控是什么原因了。2300一盒的辉瑞进医保啦， 801. 你封城，然后让各地派人来支援，不比全国散发，自顾不暇好嘛。非要一条道走到黑 802. 从个人开始，配合好这次筛查，尽快阻断社会传播链 803. 吴凡快闭嘴吧，天天不知说什么 804. 不应该是全国散发吗 805. 几千万人的城市，真正遇上事了，才能看得出治理能力，稀巴烂 806. 这个吴凡标准的精致利己主义者，遣词造句十分狡猾。和张文宏的故作理性客观的心态特别像。它们说话不让你拿住把柄，但是带风向的立场十分具有煽动性。//@真系变成老坑公:再精选的文字也挡不住冰冷的数字，请再务实一点🙏别再折腾老百姓。 807. 加油，上海。 808. 这一波迷之操作是为了带货2300那个药么 809. 昨晚pyq的上海朋友都在抢菜，约等于聚集然后分散？ 810. 君住黄埔西，我住黄埔东，共濯一江水，不得复私通。鸳鸯本缠绵，分离两处愁，区区五百米，天地不相同。鸳鸯锅封 811. 上海加油！ 812. 啥叫“面上暴发”，直接说失控不就好了 813. 全部封起来两周不就得了吗？拖拖拉拉的这持久战还得打到什么时候？ 814. 就不能诚实一点？防控政策错误，导致全面爆发，为了弥补错误，现在封城！？一定死要面子？不承认自己错误？党性呢？ 815. 我感觉自己好文盲。都是中国字，但就这个标题我都理解不了 816. 上海政府在这波疫情中表现优异与时俱进，让上海人民与病毒🦠共舞 817. 上海搞成这样，是该问责了。 818. 上海小老百姓们什么都做不了只能听那些砖家瞎比比，作孽还是自己作孽老人买不到菜病人配不了药 819. 嗯，知道了 820. 我可以反馈吗？湖北疫情的时候真的是无不出户，不出户，下楼就走人哄，不让户与户接触，除非拿菜，我朋友小区一直封确诊一直多🙈我还不理解，我们小区封了，我才明白，大爷大妈不戴口罩满小区溜达，聚集聊天，小区一瞬间变得格外热闹，这就很危险呀 821. 昨晚放大家出去抢菜不是在聚集？ 822. 到目前为止都没看见上海市政府的领导出席发布会的 823. 疫情爆发的初期大家都喊着武汉加油，北京加油，为什么最近一年只要哪个城市爆发疫情大家都开始地域攻击？谁想要被感染？谁会去带着病毒恶意伤害别人？现在无论是疫情中心普通的我们还是第一线的医生护士社区工作者在为了抗击病毒努力，已经很累了，不要再互相谩骂和埋怨了。 824. 每一个人都做好配合，战疫肯定能胜利 825. 上海这次的防疫有没有参考意义，我认为有！最少让其他省份知道，滚动网格式防疫，在现今当下病毒的传播型态中，不可行。 826. 为什么要责怪上海呢？上海没有阻止流动人口进上海工作，也没有阻止流动人口返乡。他们回家乡不可以吗？怎么不责怪他们？ 827. 上海加油🙏 828. 上海这次管理混乱，主要责任人为什么不撤职不追究！ 829. 作为上海人，真的蛮无语的。一开始把控不严，现在想控控不住了。城市人口整体素质高不代表所有人都能自觉，也不代表市民在这种情况下不会恐慌。聚集测核酸、聚集抢菜，都是高风险行为。就想问问政府和专家，想出来的政策脱离理想主义照进现实，自己不觉得离谱吗？ 830. 吴凡，呵呵 831. 抢菜时聚集，做核酸时又是聚集，希望能科学施策。 832. 这俩词不能结合着说吗？ 833. 少了个全国外溢 834. 而且是严重外溢附近省份！ 835. 不知道是谁出的馊主意在上海搞群体免疫实验现在这局面。一开始零星遏制住该多好啊。 836. 吴凡还没被撤换啊那上海疫情结束还不知道啥时候 837. 上海的领导呢作业抄一抄出入都不管制的吗 838. 我们公司还在上班 839. 真的是大言不惭的说上海对全球举足轻重，不能按下暂停键，广州，深圳，香港这些地方真的是比你上海小得多了，人家没你大，人家在国际地位不重要？上海人人均博士学位而人均写论文就能拯救世界，但凡按下暂停键十天的功夫全员核酸排查都不可能像现在这样，只有每天早上八点前，上海新增确诊病例为零的情 840. 吴凡献策其心可诛 841. 不影响上海经济发展了？吴主任讲话真厚脸皮啊 842. 同心抗疫！上海加油！ 843. 要追责吗？上海这波操作很迷幻，不让聚集不给物资封几天聚集买菜抢物资。 844. 我们怎么还当做绝症一样 845. 这吴凡还有脸出来呢？话说上海防疫被复旦系包圆了？ 846. 个人建议以后开什么疫情防控新闻发布会的时候能不能请几个人民代表回答记者的问题，比如请几个区或者街道派出的老百姓去参加疫情防控新闻发布会。 847. 赶紧下台吧 848. 不是不封吗 849. 封城吧 850. 这发现还需要研判？？？我们十几天前就看出来了 851. 那为什么还要集中核酸？！为什么？！不是说足不出户吗？那集中检测核酸是什么鬼？！要把上海跟上海人民折腾到什么时候 852. 友善评论么？那我还是别说话了 853. 所以wu凡，东海邮轮现在哪里 854. 再加上外溢 855. 可以不是全面爆发是面上 856. 封城半个月，效果肯定明显 857. 上海主政者通通滚蛋 858. 咱就问你张文宏处理不最起码纪委要查吧？ 859. 说的挺含蓄 860. 上海这么严重的疫情，还不限制离市吗？确诊病例全国各地蔓延，全都是上海输出的！ 861. 就不能说人话吗？ 862. 上海还没有高风险地区呢，为什么啊？ 863. 小赤佬 864. 还以为上海zf是不在乎呢 865. 说得坦诚一点吧，文字游戏和话术没意思 866. 咋就这么滑稽呢？？？啊？？？ 867. 不是早就应该封城了吗不理解 868. 别的不说了，我爱深圳 869. 吴凡好自为之 870. 精准防控，一起守“沪”！相信上海 871. 外溢了吗 872. 爆都爆了潜什么潜 873. 到底敌人是谁 874. 真是早干嘛去了，这是传染病已经两年了，有现成的经验不用非搞别的，现在好了管不住了，而负出的更多代价更大。 875. 反正都是你说的，老百姓跟着折腾就对路了 876. 上次说不能封城的也是她吧 877. 那不就是全面爆发嘛我总感觉离五位数不远了 878. 看不懂这新词的意思 879. 上海太难了 880. 唉~~~~ 881. 世界上海 882. 中华文化博大精深 883. 其实早风控会快点截住病毒发展，这点广州做的很好 884. 中文越来越差了已经看不懂这文字到底表述啥意思 885. 做好个人防护、勤洗手，做好个人防护、勤洗手，做好个人防护、勤洗手——重要的话说三遍！💪#上海疫情存在面上暴发潜在风险# 886. 新词get√无语 887. 啥叫面上？那里上咋样啊？想上班 888. 上海市疫情呈现区域聚集和全市散发并存两个特点，存在面上暴发的潜在风险。 889. 我真的好害怕小区封闭了之后家里的库存越来越少，不是很敢去人特别多的菜场抢菜，但是现在平台买菜真的很难抢而且特别贵。。。我甚至看到朋友圈的一些黄牛开始改卖水果蔬菜，简直是发国难财啊 890. 哦 891. 还上班呢？让浦东的员工连夜赶到浦西住下就为了上班有意思吗 892. 吴凡，牛逼 893. 她今天才发现？前几天说东海漂游轮的也是她吧 894. 赶紧封城不行吗？为了你们的面子，不顾老百姓死活！快一个月了，到现在只看到每天几千几千的上涨？转折点在哪里，啥时候才能恢复正常生活？？？？ 895. 上海最大的错误就是没把无症状感染者当成确诊病例，无症状感染传播力度必然大于有症状的确诊病例。 896. 完了，地球快转不动了。 897. 面上爆发米上都有了！ 898. 我只想说三个字，不要脸 899. 要么躺平要么封，没有两全其美的办法 900. 都算全爆发了还面上面上，整天搞些小九九文过饰非还不如踏踏实实走群众路线做好防疫//@NextSat6am:那不就是全面爆发的意思吗 901. 啥叫面上暴发？能说点能听懂的不？ 902. 就问你新冠致死率多少？重症率多少？何至于为了表面数字逼死其他病患者！ 903. 这个人……前天说上海不会封城的不也是她吗 904. 看了昨晚的操作，只觉得这情况实属是乱套了，疫情防控能搞这么乱 905. 三千多病例还不一定是真实的估计有好多隐瞒的 906. 我现在能理解欧美当初为何抄不了中国作业了，一旦遍地开花那是真难控制，想想就头大，遍地都是咋弄，一天几万几十万的欧美咋弄？只能头铁硬抗躺平了之 907. 不是全面爆发了么？一个月了，之前在干嘛 908. 没有人担责吗搞了一个月搞成这个鬼样子领导站着说话不腰疼不愁吃喝不愁失业华东医院随便住 909. 都这时候了，还玩语言呢啊？！ 910. 老百姓不能上班，没有收入，到底要不要人活………………………… 911. 这次大上海的表现真不如我们小乡镇啦 912. 上海作为抗疫模范生，也就那样吧 913. 别说潜在和面上了，已经全面爆发了好吧！ 914. 网格不灵了现在 915. 被西方专家潜伏了？ 916. 到现在公司都不下通知居家办公！ 917. 昨晚超市都抢疯了 918. 刚发现吗？之前那么松的管控措施不爆发才怪！ 919. 为什么这么多人骂上海，搞不懂。 920. 已经外溢到我们徐州了 921. 说了等于没说 922. 为什么不全面消杀呢 923. 早点封就没这么多了 924. 面上是什么意思 925. 上海外溢这么严重为什么不封城 926. 明天浦东解封，好怕怕 927. 谁不学，非要学香港！ 928. 你们上海都摸了3个星期了，还没摸清吗？害的我们苏州至今还是寒假模式 929. 早干嘛了 930. 都外溢到莆田了，上海还不封城，大无语！！ 931. 这个无妨是唱戏得吗？ 932. 吴凡官气 933. 这个吴凡能不能不要出来说话 934. 说得道理全对，做得呵呵 935. 再也不吐槽别的地方疫情管理拉垮了，我们已经快被上海这拉垮的政策折腾死了。 936. 上海这次很拉跨，被拉走的患者六个人一个房间，到现在没有晚饭，只有中午一点给了面包鸡蛋 937. 网红不是说拐点了么？ 938. 上海的鸟人们，求求你们别乱跑了可以吗？今天这边因为个上海来的阳性。又全部被封了、艹 939. 早干嘛去了快一个月了日增4000 940. 生完孩子坐月子坐完月子在做一个月子我这三个月一直在做月子 941. 这届市政府管理能力实在太差了，没有这能力。放到应勇和杨雄根本不会有这种事情发生。 942. 集体做核酸和买菜的时候扩散的吗 943. 不就是爆发的意思吗？zf能不能好好说话，欺负我们读书少吗？ 944. 连续三天早上五点半起来抢菜，根本抢不到。老人在家独居，突发癫痫，打120等了五个小时才到，在医院打完点滴，老年人一夜没合眼想回家，没有车能回去，120说前面排队50位，求助民警，居委社区，打了所有能打的求助热线，给到的回复均说没有办法，只能等120的车，怎么也不敢相信这是上海 945. 我们小区还在搞精准封控，出了阳性的楼封锁，其他楼解封正常活动，出疫情的楼还按楼层划分，有阳性的楼层7+7，暂无阳性的楼层2+12，这操作够骚的。另外一幢楼从3月27日出阳性封锁后没测过一次核酸，就干封着，其他楼从3月22日以来就没测过核酸，牛，不阳才怪 946. 简单说就是一团糟呗 947. 人间四月天，我们在隔离。不能随便骂，就等小孩做错题骂骂TA消消气 948. zf能管管菜价吗 949. 不要再折腾全国人民了，，外溢疫情让多少人跟着付出了惨痛的代价。。 950. 只要不妨碍俄罗斯打乌克兰北约美国怎么封都行 951. 区域聚集和全市发散，这个不是一对反义词吗？这是一句废话文学吧 952. 已经爆发 953. 阿拉的那几位，现在学着某位也想当网红…… 954. 突然这么厉害了，感觉是某些国家的投毒 955. 🙏加油shanghai 956. 境外输入有闭环转运，上海承压，百密一疏，应包容的，属于受害者；但在全国疫情99%一刀切的防控机制下，上海疫情扩散后，即不控制外溢也不控制内流，是不负责的。最残暴的帝都，地级市有1例就不许进京！可上海外溢了半个中国，在浙江找不到一个城市没有1例！哪天大家标准一致了，再命运共同体，可好？ 957. 说来说去不就是封城的必要性吗，早行动啊，非要弄的很严重了再封城 958. 上海老百姓真的苦不堪言有苦难言aaaaaaaaaa希望半封能彻底解决问题 959. 人家国外两周在家隔离就自愈了我们在国内还搞什么全民清零那些不会用手机的老人们估计是先饿死在家中了 960. 能不要让这个家伙说话了吗？前天鬼吹上海是世界的上海，不能封城，昨天被打脸，今天不脸红嘛？早干嘛去了，现在才发现问题 961. 2020疫情开始以后，去过广州，西安等地。哪怕低风险的时候，不论公共交通，公共场所筛查口罩，健康码等，都要比上海严格的太多，昨晚发布封到4.1时候，老人还一个人住，连忙跑去超市购买，简直了场面，有插队的，抢菜的，想想后怕，多少人啊交叉感染。发文说外卖正常，确实有，只不过预约全满，失望！ 962. 上海不要再追求所谓的颗粒度了 963. 估计不光是上海吧 964. 翻译一下，只有封城能解决问题了 965. 嘴硬的后果 966. 那前个月干什么来着!没你们块状切会造成这样的局面吗 967. 我在想，以后人人都得的话，我该怎么办呢？我会不会年轻轻的就死去？还是病歪歪的活一辈子生不如死？感觉好恐怖！人类会因此而灭绝吗？ 968. 今天才知道啊 969. 有笔记也不会抄hai 970. 早点封，能有现在这些危险？ 971. 还是错判了病毒传播太快的形式，现在核酸检测不知道还能不能搞的定，失控的最后结果估计和HK一个路子去了 972. 有什么用啊，朝令夕改，早一点干嘛，老百姓是试验品吗 973. 可以不要让她出来讲话伐看到她我气急攻心！ 974. 牛逼！希望你们不要外溢 975. 封了个寂寞，核酸检测覆盖面不全，封了五天做了4次核酸蹦出来个阳性不知道哪里来的。封了很多天的小区放出去买菜就无语了。 976. 这届政府，语文水平都不错～用词非常精准个恰当～果然只适合做领导 977. 现行的做法，没完没了，人都快疯了 978. 汉语言文学博大精深 979. 真是个笑话， 980. 建议上海试点躺平 981. 浦东听说要封控，全部跑出来抢菜，这风险也太大了，说不能聚集，那做核酸的都排了几公里 982. 说点通熟易懂的很难么啥叫面上？求科普 983. 一个医生谈点防疫专业的事就可以，何必掺乎自己专业外的事！不是蠢就是坏！ 984. 中文博大精深有学到 985. 你那是全市发散，你那是全国发散 986. 热度不及一个黑匣子高 987. 你才知道啊，你前段时间吹了不起尝试时我就知道会爆发，我不如坐你位置 988. 做好防护 989. 文字游戏 990. 老是聚集测核酸，能阻断吗？ 991. 这数字还不算爆发吗还有再爆发的潜在风险？ 992. 狗屁专家，只会纸上谈兵，异想天开！把老百姓当小白鼠呢 993. 笑死人了，不封害人，周边都跟着受害 994. 生死时刻了还文邹邹干嘛[苦澀][苦澀][苦澀]//@NextSat6am:那不就是全面爆发的意思吗 995. 好烦她 996. 全面爆发改成面上爆发，有意思吗 997. 前两天还说截断爆发可能性? 998. 嘴硬成这样？？ 999. 哎…… 1000. 全面=面上？莫非使用了降重软件进行了近义词替换 1001. 🙏🙏 1002. 我都看出来会爆发，你们不知道？早点封至于这样吗？ 1003. 网格化失败了，开始格式化了 1004. 摆脱这个吴烦不要再指挥了行嘛全上海都乱套了爆发您没有责任吗 1005. 丢掉所谓的优越感，好好防控吧！ 1006. 做核酸各种扎堆敷衍，吓死人 1007. 一堆黑话，麻烦干实事 1008. 新华社都发声了：防疫不能虚！ 1009. 狗头军师咋不发表网红意见了 1010. 啥也不说了，上海加油。上下一心，早日战胜疫情，恢复正常生活秩序。 1011. 上海领导撤职吧，别的地方没管理好都撤职了多少，上海也不能少啊要一视同仁 1012. 以前觉得在上海很安全防控医疗都很靠谱现在 1013. 这次疫情，认识了好多新名词，感觉到中文的博大精深 1014. 新冠又不死人，因为防控倒是真的死人了 1015. 吗的核酸10来次如果有一例是不是群聚的几百人全完蛋还在用这种傻乎乎的采样方式 1016. 面？何面？脸面？！ 1017. 🙏🙏🙏。。。希望上海能度过难过！。大家都做好防护吧。再有条件的情况下多囤点食物。平安！ 1018. 昨晚抢菜考虑后果吗？ 1019. 上海请重视医院收治病人！！！我刚刚刷到一个两个月宝宝感染没地去，120打3个小时都没人理的，太可怜了，这么小的宝宝高热不退真的会烧坏的 1020. 不管因为什么目的把疫情搞成这样，受苦的还是老百姓 1021. 我不知道有什么好埋怨的，疫情这种东西谁能料想到，你们这批在网上埋怨国家埋怨上海的难道和在网上祈祷东航平安的是一批人吗，既然已经发生，那就相信国家相信政府，一起携手抗疫，众志成城，你们在网上说这些难听的话，只会让奋战一线的工作人员寒心，做个人吧 1022. 上海，耗子尾汁 1023. 面上爆发又是啥？天天造新词，能不能说句人话别搁这造词 1024. 不要成为下一个香港！还是祝福上海加油！ 1025. 早干嘛去了？喝咖啡红酒去了？ 1026. 之前辟谣的都是真的了！ 1027. 这么多专家还不下课？ 1028. 上海不怕打脸，打脸就尽快遏制住疫情， 1029. 领导们在走盲棋，猜一步走一步，把之前的努力都消耗殆尽 1030. 能不能说人话？ 1031. 呵呵，伊拉老娘鞋底洗准备好了，估计今朝葛顿打逃唔忒了。 1032. 精准爆发 1033. 评论区都是专家，我就看专家组远程会议。 1034. 前天说上海不能停的也是她今天浦东闭环第一天对面马路上还有推着婴儿车溜娃的 1035. 群众的眼光是雪亮的 1036. 早在干嘛？所学的专业知识不足以支撑你们的预判了吗？从3月初拖到现在，一定要拖到爆发才能分析研判出来结果吗？前期没有做模拟吗？ 1037. 论语文，还是他们专业 1038. 呵呵 1039. 中华文化博大精深，面上面下是啥意思 1040. 已经陪跑了15天，老百姓苦死了！ 1041. 这波疫情，别的没学会，新词学会了好多 1042. 嗯，你开心就好 1043. 为在上海的人员感到心疼。除上海杠精。 1044. //@梭哈986:美国的数字好看，都放弃治疗了//@真系变成老坑公:再精选的文字也挡不住冰冷的数字，请再务实一点🙏别再折腾老百姓。 1045. 又创造新词了？做事能力没有，忽悠人一套一套的 1046. 你们说的都对，上海失守了，你们乐去吧。 1047. 折腾了一个月了 1048. 说实话，同为一线城市，广州深圳都控制得蛮好的，至少没有上千吧，北京也是一出疫情就抓得严（当然首都肯定是要严的）；同为直辖市，天津重庆也没那么夸张啊 1049. 上海的同志们，吸取吉林人民的教训，多屯粮，没毛病封了，卫生纸都可能买不到 1050. 有人拉裤兜了，被人闻着臭了，知道找厕所了 1051. 别再搞这些专家网红了，毛用没有，乱引导，踏踏实实解决问题！ 1052. 悔就毁在这个吴凡女士。太骄傲 1053. 这不是废话呢 1054. 自己把自己脸打肿了 1055. 坐标长宁区，小区昨晚六点突然被封都没说的第三次了，通知也没贴都不知道，菜也没给时间去买，排到半夜12点下楼做核算鼻咽拭子，今天继续做，网上早晨六点就开始等着根本抢不到菜，愁人。 1056. 不是说不封城吗 1057. 现在看还在咬文嚼字就有种说不出的感觉 1058. @X车厘子小丸子倩倩子Q 1059. 人口聚集核酸有几个落实保持距离的，超市聚众抢菜会造成密集感染 1060. “面上暴发”，又造新词了，啥意思，解释一下 1061. 面上？ 1062. 呵呵呵，尊贵的外省 1063. 为什么不用中药控制，武汉就是中药控制住病毒的 1064. 现在是不知道源头究竟有几个，在哪里。 1065. 文字上玩花样，不就是要封了吗，说得那么清新脱俗。 1066. 上海别加油了，毁灭吧 1067. 买不到菜怎么办，一颗白菜70多没人管了吗，在软件上天天抢不到，年轻人还好，让独居老人怎么办突然通知两个小时时间可以买菜，都涌出去了，发生感染了怎么办，能不能管一管上海的送我上去 1068. 呵，果真是魔都。没事乡下人，有事一家人。还往浙江转送病人，自己处理不好让别人擦屁股。 1069. 感谢吴亦凡的妈妈吴丹送来的小阳人 1070. 还有别的特点吗。服了 1071. 吴凡你踏马打脸不？ 1072. 祸害全国 1073. 原来国自科面上项目就这个意思 1074. 上海zf女内瓦萨 1075. 张邬吴这三人误导政府防的防疫判断决策，给上海及国家造成的损失和伤害太大了，三粒耗子屎祸害了一座城，应该严办 1076. 当断不断必受其乱 1077. 上海这次疫情这么严重，政府还玩文字游戏，多少个谣言变成真的了，等着秋后算账吧，一群才白吃猪 1078. 上海探索失败 1079. “实事求是”4个字还记得怎么写的吗？这群官僚 1080. 把我们这害惨了，吴凡还不撤职？？？人员安全第一位被谈到脑后了？ 1081. 玛德 1082. 疫情大家都是受害者，现在这个疫情不都是那些所谓的西方“绅士”导致的！ 1083. 就觉得ummm挺会造词的，所以什么叫面上？ 1084. 大家都明白的道理，到了国际大都市的吴专家这里，花了一个月才搞明白表示不理解啊可能人家是专家，需要科学严谨吧。可就是苦了我们这群打工的 1085. 我是真的无语了将近一个月这样真说不过去吧 1086. 要不吴凡去见吴亦凡吧 1087. 有史以来对上海第一次如此失望，我们在这座城市工作、纳税，我们为这座城市更美更好付出了这么多年的心血，最后……一次抗疫就暴露了如此多的谎言和骗局，真的不希望在电视上看的古代那些草菅人命的故事在现实中上演。。。希望上海尽快好起来，希望有担当的人尽快带领上海走出这次阴霾。。。 1088. 我们公司让我们去外面自己做核算，准备入园区工作，不是聚集感染吗 1089. 浦西还在努力奔波上班…… 1090. 领导，话都让你们说了 1091. 真的是这一个疫情看清了每个省的实质，这样下去什么时候才能到头啊，整天发一些不清不楚的文字，真的让在上海的人寒心和气愤 1092. 可能来个专家管管上海，我们安徽陪跑的一个个在家都不敢跑，回个老家还得经历三次核酸，出门都不敢去商场，日子过的是真憋屈，确诊病例都是国际大都市上海来的，凭啥呀 1093. 我今天是被隔离的第二十二天，有没有比我长的，中间从来没有解封开过门的哦，从三八妇女节到清明节，希望清明节看到希望 1094. 希望上海下个决心完整的封闭，而不要搞这种区域/分批，现在人还是在流动，有流动就有感染，这样永远遏制不了啊！而且那么多人都不戴口罩。真的不能再继续这样下去了！浪费国家资源财产人力物力都吃不消啊！拖延不如短痛啊！ 1095. 这个话题，就是有可能全面爆发力 1096. 就害怕上升趋势控制不住，周边省份的普通老百姓真看下不下去了，每次都是刚清零又来一个。 1097. 面上是啥意思？？ 1098. 抢菜的时候感染一波，集中核酸再感染一波，感染完放出去抢菜再去感染一波 1099. 到现在为止封控区内还在遛狗溜孩子呢。不爆发才奇怪了。上级要求足不出户，实际执行足不出小区，但小区里随便溜，聚集都没人管。 1100. 大家在抱怨上海的时候，就是完全接受了过去三年麻木的防疫生活。三年抗疫，病毒也演化了无数次，上海作为中国医疗经济都与国际接轨的城市，承担了巨大的压力。也在积极探索着新的更好的出路。可是人们在探索的过程中就开始抱怨责骂。最终上海失败了又能怎么样？继续面对一次又一次的封城核酸戴口罩？ 1101. 昨日上海新增无症状4381 1102. 放大家出去抢菜，能不爆发吗 1103. 都半个月了还没一点减少的趋势反而越来越多，上海了不起。 1104. 精准防控防了个寂寞 1105. 我好奇的是，不是已经爆发了吗？ 1106. 啥叫面上爆发 1107. 追责 1108. 还不如吴亦凡 1109. 感觉上海可以封城了。昨天确诊已经96了，今天估计得上百，无症状4300多例，一直在往上加。 1110. 风险确实存在不少，《封》只是对部分觉悟高的人，虽然小区封了，但很多人还在小区活动平凡，而且不戴口罩，聚集一起，封两天，放两天，聚集核酸，聚集买菜，可不照样传染。 1111. 就光想着造词了是吧？玩了老百姓一个月防疫防了个啥？？？ 1112. 这句话就是说，我告诉你们有可能爆发，希望你们心里有数 1113. 能不能直接骂领导？西方先进的抗疫失败的经验，非要搬到上海，亲自复制一遍，不撞南墙不回头。口口声声为经济，但经济显而易见受损失。口口声声为市民，但人民群众饿肚子。领导们没水平不作为，疫情暴发到现在，都在忙什么？ 1114. 不想骂上海人，上海人已经够辛苦了！但是，能不能直接骂领导们？ 1115. 早干什么了？大家上班那么久 1116. 一个月了，别再分析了，该干嘛干嘛吧 1117. 上海真的最差疫情防控，早封城不就好了，“精准防控”让疫情得不到遏制，shi拉身上晓得脱裤子了是伐？不能上班怕聚集感染，超市挤满人抢货就不会聚集性感染是伐？菜价暴涨连大超市都在发国难财，不管？ 1118. 现在，弥补过去的错误，并坚持着拆未来的台 1119. 亡羊补牢 1120. 能不能放10万个骑手小哥哥出来呀我们小区3.28之前刚刚关了4天，又直接被关，而且还没有明确究竟4.1还是4.5出来，关之前的买菜抢菜时间为0。网上根本抢不到，不是菜没货就是骑手小哥哥没货 1121. 真的是垃圾闵行我们已经封闭半个月了做了那么多次核酸了，为什么现在还有这么多才被发现呢！到底是怎么管控的 1122. 抠字眼，面上爆发，所以请问我白欠的半个月班，领导们还吗 1123. 文字游戏666 1124. 前天晚上，满朋友圈都是菜场里人挤人抢菜的视频，突然公布政策的领导没有想过这种局面吗我理解不了，难道不担心密接？有这个结果一点也不奇怪 1125. 坐标普陀区，自从上周开始封闭小区，小区里的大爷大妈只要不下雨就在楼下单元门口聚集聊天，口罩也不戴。出太阳小区花园里全是人，好多人也不戴口罩。自从上周五小区解封，现在每天都满是出门抢菜的人，现在的小区比以前热闹多了，全是感染的风险。发的抗原试剂盒给每户，全靠自觉。漏洞百出的防疫策略 1126. 昨晚上海封城，我以为今天的太阳升不起来了。3月28号:地球停转日 1127. 浦东封了后在本小区内传播，浦西不封全浦西传播，等到封浦西时候已经晚了，4天完全不够，核酸检测都不够。 1128. 真的摆烂了，哪怕现在我们小区都是自觉楼下核酸，一个小区被确诊了两栋楼了。真的每天心惊胆战的 1129. 面上？又是个新词儿 1130. 吴凡下来，张文宏上 1131. 就是全面爆发的意思吧今日新增4千多例了，不把每个人都阳了誓不罢休 1132. 吴凡你可闭嘴吧，封城几天难道地球就不自转了？天天MD的打脸，疼不疼！ 1133. 不是我吹牛，对比天津！天津这赢麻了！ 1134. 不然呢做个核酸排五个小时队几千人聚集在一起绕来绕去s型排队做核酸真的是充分利用每一寸土地这样聚集意义何在 1135. 早就该封城，天天想着造词 1136. 现在不是疫情，主要的是人民群众生活保证！！！强烈要求快递正常运转！！！！！ 1137. 先考虑考虑活人吃啥吧 1138. 能决定上海抗疫政策的人既不懂上海也不爱上海，还欠缺对自己zz跳板最起码的尊重和责任感。 1139. 防控新模式。加油 1140. 喷也没必要喷了，就只想上海抓紧处理赶紧清零，别在抓着自己的脸面不放了好嘛，都什么时候了，我们只想赶紧上班，要不然买菜钱都没了！ 1141. 全面爆发，当初怎样报道欧美，现在轮到自己 1142. 简单点说就是有像香港一样的潜在风险？ 1143. 小区排查不一一核对万一有不下楼核酸的呢那小区里永远有隐患啊 1144. 论一个好居委的重要性。具体内容请大家补充。 1145. 一个多月了，明明可以在前期控制住的，发展成这样谁的责任？有人担责吗就问 1146. 一直很配合但是耐心要被磨没了 1147. 上海这样子扩散到那么多个外省上海的领导怎么还没被问责罢免我记得以前那个省管控不当都被罢免了啊 1148. 正在危急时刻了！ 1149. 什么叫面上爆发风险？上海在造词方面好像比较拿手 1150. 上海这么严重还不封城。 1151. 物种同病毒是协同进化的。病毒，注定要把其基因片断植入人类的DNA，成为对我们人类子孙后代有益的内源性逆转录病毒基因。从合胞素病毒基因就可以明白，没有病毒，就没有人类。人定胜天的思想要不得！上帝是严历的，也是仁慈的，不付岀代价？！想得美！ 1152. 承认吧，就是你们的防疫措施错误、失败，事后看看要不要追究责任 1153. 睡醒了？ 1154. 我们小区有个人板条做出来阳性，还不主动上报，等居委会的人来追问，才吞吞吐吐给出来。这个时候这个人都跑到医院拿核酸结果了 1155. 这是在打预防针了，大家对之后的数字有心理准备了 1156. 天天测核酸聚集越测越多。小区天天2➕12重新计时有意思么？没完没了了还 1157. 这个吴凡可以滚了吗 1158. 每天玩文字游戏有意思吗。。中午去超市就连个菜叶子都没了 1159. 打倒沙皇细菌战！ 1160. 上海还不果断采取更好的防控措施吗？ 1161. 我们的损失上海承担得起吗？你们不是不需要封城吗？你们不是不需要管控吗？居然能谈到国际损失我也是笑了骂归骂，还是希望上海加油！专家学者们别再卖弄了，实际点吧 1162. 请直接点，完全失控。脸打得啪啪响，还死要面子。被外溢的城市倒了大霉了。已经好多天不能出门了。 1163. 人祸 1164. 这是在讲些啥？ 1165. 区域聚集和全市散发是啥意思？强行挽尊呢 1166. 龙吴路已经是重灾区了，也不消毒，也不天天做核酸，没事的人都被感染了，核酸异常就打电话说一下，也没有后续了。这就是上海？？？ 1167. 是爆发，不是暴发，新闻媒体都错别字了 1168. 都暴发了，还需要你们分析吗？ 1169. 赶紧封吧，到底哪天是个头啊 1170. 我们黄浦区楼下确诊两天两夜阳性患者疾控都不来转运隔离，上海是瘫痪了吗？ 1171. 洋泾街道崮山小区四街坊（羽山路1445弄）。在3/26抗原自测后确诊13例阳性患者，涉及9个楼栋。涉阳楼栋没有专人监管，楼下只有细绳一根予以“封锁”。楼里依然有人员出门下楼扔垃圾等。据悉，截止到现在3/28晚上只有2名阳性病例被带走隔离。整个小区也没有做过消杀处理。 1172. 4月2会不会变成1万6人左右… 1173. 无意冒犯，但，请专业、务实的人来做吧[小红花] 1174. 吴凡讲的真好鼓掌👏 1175. 不就是为了卖2300一盒的所谓进口特效药么 1176. 我们附近小区，有很多感染的都没有被拉走，小区业主着急得不行 1177. 我觉得大家都别喷了，深圳怎么做得好上海怎么做得不好，都是政府的决策问题，身为平民老百姓的我们只能配合政府决策。现在就是希望上海能赶紧找到适合自己的抗疫路子，大家能赶紧复工复产复学！ 1178. 还有个人问题：啥叫面上爆发，米上不爆发吗读不懂 1179. 这种人还能出来发言，没有人管吗 1180. 说这话的意思就是，要全面爆发了，做好心理准备吧 1181. 再上网课就把祖国的花朵全耽误了、孰重孰轻啊病毒不可怕、管理制度最可怕 1182. 一言难尽，想骂人 1183. 听君一席话，犹如一席话 1184. 我现在的小区属于闵行东兰路欧风花都小区没封，但是刚刚扔垃圾就看见封，有人阳性了，但是我们小区已经解封了三天了 1185. 我能骂人吗？哦，不能啊，那无话可说了 1186. 张一鸣都没你们会说黑话 1187. 今天说的和昨天说的，打自己脸都不知道 1188. 早封城隔离14天就可以了，现在可好，隔离14天了，还要继续隔离14天 1189. 区域性聚集和全市散发并存不就是防疫崩了的意思吗 1190. 这是优化吗？这是亡羊补牢 1191. 求大家帮帮我，我家中有一个1岁半病重孩子需要我回家照顾，但是我23天前被确诊后，一直没有给我治疗，一直把我关着，而且不断的像羊一样被转运。我已经好了半个多月了，昨天把我换到一个新医院后告诉我还需要再关8天才给做核酸。算了一下，等我出去就已经2个月了，我想回家照顾小孩，求大家顶一下。 1192. 玩弄文字也要不回脸 1193. 疫情结束立马离开上海这个鬼地方 1194. 全市散发确定不是全国散发 1195. 然后还是没有高风险区，然后还是不封城，给全国各地输送传染源 1196. 我看同城信息，很多已经通知核酸异常的，竟然都没有人管，求求上海做点人事吧 1197. 多数地区核酸检测都挨着排长队，一个张完嘴下一个上去直接张嘴，买菜也是一窝蜂，哪有什么安全可言 1198. lj 1199. 上海人民扫墓的权利也被剥夺了，什么奇妙的防疫政策导致的 1200. 面应该等于社会传播层面吧😂 1201. 感觉深圳办事还是很高效的 1202. 上海快点好起来吧🙏 1203. 本来刚开始我也很相信上海的觉着没事的，渐渐地我开始无语开始骂上海了 1204. 赶紧滚下台了，祸国殃民。 1205. 上海滩 1206. 在精准防疫下没有几何增长稳定了世界经济 1207. 早干嘛去了呢？不说也是聚集和分散两种传播方式呀，还砖家、院长？？ 1208. canuspeakchinese？Icannotunderstandyourmeaning. 1209. 上海人别到处跑了，安心在家居家办公，能影响多少，别人都行到你们就不行了，真可笑哦 1210. 在上海，我党是地下党？ 1211. 好怕明天一觉醒来，新增四千余人 1212. 领导舍不得经济又舍不得乌纱帽，两难哎 1213. 疫情初期不管控还说封城影响世界经济，现在大规模爆发控制不住害得我们也没法生活 1214. 上海市委不作为，疫情初期不管控还美其名曰为了世界经济，现在疫情大范围爆发管不住了害人 1215. 又不封城，隔这说个锤子 1216. 新鲜又精辟的词语将层出不穷 1217. 才发现吗？ 1218. 真是恬不知耻从初三个感柒者弄一个月现三千多个感染者封城了，竟然说取得阶段成果不要脸到了极点。 1219. 上海人也都无语了 1220. 暴发？已经到了用这个级别的词的时候了吗？ 1221. 上海怎么这么久了感觉更严重了呢？ 1222. 人手一盒特笑药 1223. 面上爆发 1224. 前一天说不可能不封城，大家已经不囤菜了，第二天晚上直接给浦东人民四个小时抢购，发布会的话还能信吗？浦东人民太苦了 1225. 聚集加散发那不就是全面爆发嘛！ 1226. 面上爆发！几个意思？ 1227. 养小孩的（或者你曾经是个小孩也行）都知道，护，是护不了一辈子的，迟早要出来混。看大家能封个两代人吗？崩溃的出生率可玩不起。 1228. 青椒看见面上表示很淦 1229. @幸福平安1C:为什么不用中药控制，武汉就是中药控制住病毒的 1230. @Anneyuki:人口聚集核酸有几个落实保持距离的，超市聚众抢菜会造成密集感染 1231. @天天快乐必须的:老是聚集测核酸，能阻断吗？ 1232. @空调有话说:浦东听说要封控，全部跑出来抢菜，这风险也太大了，说不能聚集，那做核酸的都排了几公里 1233. @我就是那腕儿:那为什么还要集中核酸？！为什么？！不是说足不出户吗？那集中检测核酸是什么鬼？！要把上海跟上海人民折腾到什么时候 1234. 我邻居阳性不带走的，他妈的，还能出门 1235. 离家300米就有疫情，但每天还要去上班。隔离的几天没有去公司，到了正常休息日还要义务补回来，这能控制得好吗？ 1236. @壹拾贰灬:浦东限时3小时买菜挤爆各大菜市场，不就是大型感染现场吗，政府不知道怎么想的 1237. 住在上海，从头到尾的管控方式要把我们气死的节奏 1238. 愿上海dl，莫要祸害中国 1239. 真的对政府失望这波操作我无语 1240. 昨晚的超市抢菜这一操作就很迷，咱就不说别的，诺大的上海，连个民生都不能保障，这种时候扎堆聚众，核酸检测不能跟上，老百姓叫苦不迭！面子有啥用，脸不就是用来打的么！都说知错能改，善莫大焉，百姓兴才国家兴啊！ 1241. 给我们闵行封了半个月说要搞精准防控，结果新增越来越多，垃圾 1242. 没有一次核酸距离保持一米的！！！形式主义！ 1243. 吴凡这女的，总是… 1244. 毁灭不是梦 1245. 上海真的有在认真防御吗？？？？为什么有人阳性在家没人管还带着一个孩子至今孩子都还没有做核酸.打了很多电话都没人管这个事情有意思吗？？？ 1246. 这跟互联网黑话有什么区别？？ 1247. 别创新词了，弄的那么矫情！这次不处理几个（尤其张，邬，吴）下次还会来个更大的。。每次都是老百姓最倒霉！！ 1248. 防的好了叫精准防控，没防住叫为全国探索新方法，咋说咋有理 1249. 上海能装逼 1250. 能不能正经儿的封控十天半个月的如果一开始就封控，疫情可能早就控制住了 1251. 然而，我们还没被封，进出自如，坐标浦东川沙 1252. 浦东封控之前大批市民扎堆出去买菜难道没有风险？ 1253. 能不能保证老百姓正常生活的前提下再说这些每天定闹钟抢菜还抢不到好家伙封城前还放人去抢菜聚集都被你聚集了接触也让你接触完了还封啥呢？真是大笑话了 1254. 我看成吴亦凡 1255. 123456789 1256. 文字游戏玩多了 1257. 就是控制不住了呗，装逼装大了，气球吹爆了 1258. 都这时候了还在玩这种文字游戏，是个人都看得出来，不完全封闭，根本无法做到阻隔疫情传播 1259. 魔都的领导们能不能扎扎实实把疫情防控方案落实到基层？封控前几个小时，特地全面限时解封扎堆抢菜打架，那么高的感染风险没人管。文件上讲保证孕产妇危重急症就医，结果一封控，人也找不到，不管是孕妇/癌症化疗/血透有些小区就是咬死了不放，居委电话没人接，120打半天，定点医院不明确是否收治 1260. 张网红不出来说几句吗 1261. 昨天临时放出去了多少人统计过吗？经济全靠上海市民在贡献！人命是一点不看重啊 1262. 关键是光封不管有什么用，发现阳性病例都不能及时收治，什么密接次密接更是无人管，这次疫情充分暴露了上海管理水平的欠缺，上海整体素质高大家遵守规则的意识强，规则有大家都自觉。但真遇到事了就是考验管理水平的时候了，上海的管理就出状况了，处置疫情准备不足执行不力，基层管理跟不上。 1263. 应该追一下领导的责任 1264. 这么说吧，上海干脆首先放开，危重症救治，轻症集中隔离，无症状居家隔离，就当做实验吧。 1265. 这个人今天说的跟昨天的不一样 1266. 绕啊绕啊绕 1267. 又学到了新词汇“面上爆发”！谁说全面爆发谁就是造谣！等下官方又要出来辟谣！官方整天出来辟谣也挺辛苦的！大家就别麻烦官方了！ 1268. 能不能不要创造新词 1269. 还不封城呢？全国开花儿 1270. 你们到底能不能关注一下吉林的疫情！！那帮领导摆摆样子你们就当没事了？各家各户停水停电，没有吃的，根本就买不到！你们发的资源发到哪里去了？你们解决不了事情就别压长春疫情的热搜 1271. 咱这边就是说能不能不要造词，什么是“面上”？脸上？表面上？全面？都啥时候了还不敢实话实说 1272. 能不能换领导班子管一下上海大无语一直疫情外溢别的省份就该受着吗 1273. 这次上海政府真的拉垮，民生都没办法保证。 1274. 听君一席话身高一席话 1275. 吴凡直流的对爆发没有兴趣，只对搞新名词糊弄百姓有兴趣，百姓苦啊！ 1276. 不行！上海这么一封，东海飘荡的货轮怎么办？全球经济你就撒手不管了？还有世界和平、地球自转，整个太阳系都得乱套！ 1277. 面上暴发是什么意思？？ 1278. 上海是国际最重要的大都市。怎么可以封？封了会影响宇宙的正常运转。我日增几千又如何？我骄傲了吗？你们外地人懂什么？再敢乱评论乱喷的，一律删除，精选评论。不要不识抬举。 1279. 赶紧控制住上海吧！烦死了！ 1280. 最好的时间窗口期已过。上海复星一年前订购的1亿针剂复必泰一直过不了国药监注册许可，刚进口区区2万盒的辉瑞口服新冠药一盒国内定价是2300，普及不了。不管应不应变，3个月的黑暗时刻是要经历的，可惜了，钱如黄河之水滚滚流入核酸检测上#上海疫情##吉林疫情##上海将以黄浦江为界分区实施封控筛查# 1281. 新词不能断 1282. 先管好抢菜的和核酸检测聚集的乱象吧 1283. 只是睡一觉变化就这么大吗 1284. 这么说话绝对让标题党无法拿捏，我承认我点开看了两遍才明白撒意思【#上海疫情存在面上暴发潜在风险#】#上海核酸筛查区域施行交通管制# 1285. 就别玩文字游戏造新词了，赶紧筛吧 1286. 唉，不管怎么变着法换说法都不如每天这个数字直接，连钟老都未敢对上海抗疫有半点置喙，就网红每天在发表高见 1287. 一点看不懂大批量人去买菜，为什么不是配送到家门口放着看看买个菜还能打架，真心寒 1288. 面上爆发里子不爆发？ 1289. 我已经无话可说了，和男朋友上海苏州之隔，已经快一个月没见面了，这个情况看样子起码还要一个多月，大写的服气🙏🙏，一开始隔离谁不愿意配合呢？一个月过去了数量反而才开始剧增，谁能不气呢？我真的很无语 1290. 将帅无能，累死三军！中国的老百姓真是苦，逆来顺受得… 1291. “精准防疫”四个字现在看来多么的讽刺，在生命面前请务实一点吧！不要拿人民的生命安全开玩笑！网格化、切块式网格化，下一次又是什么新名词？！希望这次所谓的“压茬推进”能做到切实到位！ 1292. 面子都不要了很快就里子了 1293. #上海疫情存在面上暴发潜在风险#合着一个月来都是地下默默爆发吗？ 1294. 那么请问面上爆发的原因是什么？是因为聚集检测吗？ 1295. 倒霉的还是普通人，有上海领导因这事免职吗？ 1296. 上海以后作为防疫的反面教材来总结经验和吸取教训。 1297. 你们市里爱怎么样我们根本不关心，别把你们上海的阳性送到别的地方了行吗？？ 1298. 早点封了这个月都能结束的 1299. 上海大法好：因城施策，精准防控，静态清零 1300. 警察威胁转阳的妈妈离开自己已经感染的孩子，核酸检测报告隐瞒不报，就是因为你们精准防控害我不小心被感染阳性，非要哄着骗着我离开两个小宝宝去隔离点，说是什么有医生救治，一天了，除了喝口谁吃了点饭还有啥救治？ 1301. 把人民当傻子忽悠 1302. 什么狗屁网格化管理，精准防控都是扯淡 1303. 赶紧封城吧，各地疫情都上海跑出来的带去的上海防疫就这水平？？屁 1304. 面子上… 1305. 先管管一些独居比较弱势的老人吧 1306. 上海zf疫情可控范围时候，还标杆自己精准防疫，真正疫情爆发了，打脸结棍了，完全无头苍蝇，现在一地烂摊子，阿拉老百姓苦死，这些官员只适合平日喝茶看报朝南坐 1307. 老百姓让你们折腾的不行了，早点封城的话，现在是不是早就好了，现在呢，封了开，开了封，没完没了 1308. 就不怕国际货轮在东海上漂？就不怕影响中国经济和世界经济？ 1309. 普通上海市民很难，周边城市很慌……城市管理和防控务实一点可好！南京今天又一个沪返宁确诊… 1310. 所以上海为什么不封城啊！你们城市有疫情为啥还要来祸祸别的城市（西安），你们经济高就可以为所欲为？你们嘴上说控制疫情，反而没控制还越来越严重，你们城市的人不惜命我们还惜命呢！能不能为别人考虑一下，有点素质好吗 1311. 什么叫“面上”，我真的不知道这个词什么意思 1312. 不管是全封还是区域封，你可以试错，但至少还是清零政策，但是某些专家开始提议开放群体免疫，这能忍？ 1313. 集体核酸，集体抢菜！能好才怪呢 1314. 日增3500了，就这还不叫爆发吗？ 1315. 让已经居家隔离14天的我情何以堪啊不还得继续隔离8天吗 1316. 上海防疫搞的真崩溃，一塌糊涂。网格化核酸肯定容易交叉感染的，同一个位置空气摘掉口罩检测~ 1317. 每家每户送点菜免费大家挤在一起 1318. 可以一波抢菜 1319. 是全市爆发还是全国发散啊！求求了，你们有你们的政策，但别了，因为上海，我和男朋友多久没见了，今天又外溢一个。 1320. 哈哈哈哈！！！上海真的会每天给你惊喜，每天都有打脸的事发生！！不是说指数级增长已经消除了么，又来个面上爆发？？上海真的是造词大王 1321. 我只想知道这次的政府部门领导的名字都有谁？TMD 1322. 区域聚集，全市散发！这两点用得着总结吗 1323. 无症状感染的这么多，是不是都有抗体了 1324. 我们读书少，求翻译，什么叫TM的面上爆发，面上爆发该准备多少粮食，全面爆发又该准备多少粮食//@想你了不小心又弄了一手:造词小天才//@大周实话部:你造谣，我们这叫面上，你说全面那就是造谣//@紫罗兰Oran:面上爆发？这个用词绝了//@NextSat6am:那不就是全面爆发的意思吗 1325. 4天能保证清零的效果吗？ 1326. 早干什么去了？ 1327. 除了整新名词，魔都到底哪里做好了？防控和民生，哪样做好了？ 1328. 这还不是爆发吗？ 1329. 上海老百姓真是苦 1330. 这也叫封城嘛不是照样可以进出上海吗我们还是没有封城喔外地狗子们 1331. @陈老师岐黄课堂 1332. ZF死了 1333. 还不如直接封城，等到全面爆发来不及了 1334. 钟老才能带好队 1335. 过好当下的日子你有脑有腿就去没有疫情的地方呆着少埋冤你的埋冤一文不值没人会理会没人会按照你说的做一针不打坚决不做集中检测是对自己最大的负责 1336. 《聚集》 1337. 希望早日好起来富强民主文明和谐自由平等公正法治爱国敬业诚信友善 1338. 翻译翻译，什么叫面上爆发 1339. 普通老百姓早就看出来危机重重了，你们这些专家咋才发现 1340. 吴疫凡 1341. 大家聚众做核酸，能不互相感染吗？谁可以做到1米距离排队？上面的人都不懂就瞎指挥！上海卫健委和疾控中心到底在干嘛 1342. 上海老百姓苦啊 1343. 么得菜吃，死贵死贵的 1344. 我都阳了三四天了还在家，也没人来带我走 1345. 苦的是老百姓 1346. 搞学术的好好搞学术，搞行政的好好搞行政…各行各业技术管理不分家，专业做做就转管理，这种模式不太好。各有所专各有所长才。记得前两年至少还能看到福奇这种在舆论媒体上和特朗普相互挤兑硬刚的新闻，可咱们的专家就有点儿没劲，一个医护专家堂而皇之的拿经济影响说教指导防疫政策的选择就不地道。 1347. 天天聚集核酸就是违法，明明可以唾液采样非要鼻子嗓子眼 1348. 早就应该果断坚决了隔离第15天 1349. 呵到现在还在玩问自己游戏早就全面爆发了好吗每天只知道辟谣都一个月了都干了什么上海老百姓苦透苦透 1350. 已经这么多了，感觉只有控制人流才是最有效的办法，一定要借鉴别省成功的防疫经验呀 1351. 翻译一下就是：接下去我要报出来的数字你们做好心理准备 1352. 精准 1353. 现在才说是不是晚了点？东明路街道为什么京东都不发货了，是想饿死我们吗？偌大的上海肉菜蛋奶一点不配，现在已经忙着写歌颂稿了，要不要脸！！ 1354. 为啥聚集呢？一核酸一大片，能不爆发吗 1355. 区域聚集+全市散发。。副院长真能整新词啊，这不就是到处都有了么 1356. 省流：要封 1357. 该作为的不作为，失望。但是为了其他城市安全，还是不要放上海这边的人员进出吧！虽然我在上海，虽然我们被封了半个多月，虽然一斤西红柿18块，买菜全靠5点59分抢… 1358. 别开发布会了，请他们下基层干实事去行吗 1359. 存在面上暴发的潜在风险=存在全面暴发的潜在风险？上海官员精通语言的艺术！ 1360. 上海官方发言越来越互联网黑话了 1361. 就是脸面上要爆发，挂不住脸的意思，上海其实还是很安全的，全上海没有一处高风险 1362. 社区里还在流动的羊，是当务之急！ 1363. 一个星期前就应该全封了 1364. 老吴专家早点为何不说呢？ 1365. 你tmd什么东西指点江山 1366. 知道要爆发昨天还放人聚集买菜？？这什么脑回路 1367. 上海是世界的上海，🌍要停转了，加油加油 1368. 面上爆发，就是脸上爆发的意思吧 1369. 学到很多新词，以后写报告能用 1370. 建议上海市这次浦东和浦西的核酸筛查一定要本人带身份证原件才能做核酸。因为深圳前期核酸筛查的时候，有人怕聚集做核酸的时候感染新冠，所以把自己的健康云登记截图发给别人，让别人冒名顶替做核酸，让自己的健康云有核酸阴性记录。上海现在做核酸只用手机二维码就可以做，容易被人钻空子。 1371. 狗公司还不让居家办公 1372. 服了！别整新名词了行不行！那不就是全面爆发吗？！ 1373. 已经买不到食物，不给配送，配送费500起步救救我吧 1374. 要求严惩上海抗疫不利的领导，必须追责 1375. 《面上爆发》你们除了会发明新词汇你们还会干啥 1376. 存在爆发风险？现在日增的3500例还不算咯？莫非会爆发到日增5000到10000例？ 1377. 简直无耻至极！！ 1378. 为什么不能学香港？ 1379. 上海这波啪啪打脸 1380. 所以就让老百姓用血汗钱去买昂贵的菜来促进经济发展吗 1381. ”存在全面爆发的潜在风险“？这不已经全面爆发了吗？还他么”潜在”？非要日均万人才是爆发？ 1382. 大姐，老百姓封了快20天，商铺也封了20天了，想起还有全面了 1383. 谁跟我解释解释,什么叫“存在面上”爆发的可能性？ 1384. 就这还清零 1385. 说的真的蛮好听的 1386. 不是有人传8000吗虽然辟谣了但是是真是假谁知道呢三千多上了热搜那8000也不远了 1387. 昨天网红医生还说已经掐断拐点 1388. 小区没封，本不想出门，但不出去买菜，线上根本买不到蔬菜（或者囊中羞涩买不起），马上要封浦西，今天只好斗胆出门，菜场早封了，街上小门面买了些能储存的菜，赶紧回家酒精消毒，洗澡，换洗衣服。如果通知统一配送的话（自费），谁要出去？！还是自己家里最安全！🙏🙏🙏核酸检测认真通过 1389. 拐点，往上拐 1390. 当然是这样的现在外面还有不戴口罩聚集的，乱窜的，为什么还不出台相应政策法律法规，我昨天还看到阿姨妈妈去森林公园玩 1391. 很高兴上海被推上特搜，希望上海以后不要什么事都硬扛了，自己过自己的小日子吧，别一会帮助这个一会支援那个，你也就是个普通城市而已。大家都去深圳吧，深圳真的挺好的。 1392. 就是在放屁，都现在了还在逃避责任呢 1393. 上海领导放下面子吧，面子值几个钱，百姓的安危才是根本。 1394. 给上海点赞 1395. 作为上海人，真的蛮无语的区域聚集全市发散这些老百姓早就知道了，你们这些专家才刚研究出来吗？一开始把控不严，现在想控控不住了，聚集测核酸、聚集抢菜，就想问问政府和专家，想出来的政策脱离理想主义照进现实，自己不觉得离谱吗？反正这种事苦的是老百姓，累的是基层 1396. 不懂就问，啥是面上？咋那么搞笑呢？文字游戏玩的真溜！ 1397. 领导换了吧，这一波不太行，能把疫情搞成这样，也是头一个了 1398. 早就该全封城了有些素质差的没封小区的全城到处晃还说路况好城市空呢我们封闭的小区前面这么久难道是在给他们享受空城让路？ 1399. 早点儿封怎会成了这样？如果封了，一个月也该解决了，现在倒好，一发不可收拾，才想起封了做核酸真。。。优。。秀。。。。。。 1400. 说的都是什么啊，每次都要一个字一个字的琢磨 1401. 这段话是给中央的人看的 1402. 还能送外卖收外卖就极不科学，啥叫无接触，到小区门口拿外卖这难道不算接触吗 1403. 稀巴烂 1404. 那昨天浦东有那么多已经在封闭的小区忽然放开几小时让居民出去抢菜是什么鬼操作？！ 1405. 聚集抢菜、聚集核酸我谢谢了！ 1406. 先封浦东再封浦西就是不封上海昨晚还解封两小时让广大群众去超市抢菜这操作 1407. 你不清零就不解封吗、大家失业饿死事小是吗 1408. 早干嘛去了 1409. 上海人民那么配合难以遏制蔓延的话，香港感染更多又不配合又不封禁就不可能控制疫情 1410. @璃染绿@我喜欢你们叫我晨儿俺们不是全面爆发，俺们是面上暴发，知网查重一遍过 1411. 为什么还要玩文字游戏？承认一句管控失败没做好很难吗？街道小区管理一团乱！今天3.28我们小区还有黄码女子硬闯小区最后报警警察说他们只管出不管进最后还是让她黄码进来了！就这样的管理上海何时能清零？！ 1412. 什么面上，有病啊 1413. @_Sssshadow洋盘面上爆发咋不在咖啡上爆发 1414. 你就是个辣鸡，一天一个说法，完全没有考虑实际情况，什么上海不能封，表面的平静怎么可能一直掩盖 1415. 那是因为一会这个政策一会那个，大家都去抢菜了，饿死比❤️冠可怕吧 1416. 不断的提炼推出新的文字。 1417. 遣词造句的能力！国家需要这样的好领导！//@赫魯暁夫:回复@人美声甜空酱:你全脸长痘了，你脸上长痘了，是不是听起来脸上没那么严重一点//@NextSat6am:那不就是全面爆发的意思吗 1418. 被封的要造反，不封又外溢被全国骂，反反复复不伦不类，咱就是说疫情三年了，少折腾点老百姓不好吗？到底要采取怎样的措施坚固民生经济，国家是否还是需要转变策略，不要来一下就封城非要坚持清零，说到底都这个阶段，抗疫不是医疗体系的问题而是行政体系的问题了 1419. @猛男小公举_ 1420. 没见过打疫苗大奖赛，奇葩这里有，就知道会有问题 1421. 这话说的有水平，啥意思，是中午不能吃面了吗？ 1422. 这还用分析吗。。。。。网友们早都看出来了啊。。。。 1423. 一定不能封城，专家说会影响全球经济 1424. 虽然没在封控区，但是上次封闭小区两天全员核酸的时候就发现线上买菜抢不到，所以现在每次都会囤很多，两个冰箱满满的，生怕哪天一睁开眼，小区封了 1425. 就是全面爆发不可zudang阻挡，不以zf为 1426. 大家很配合隔离的，就是隔离的太久了 1427. 新闻发布会不应该说的通俗易懂吗？你面对的是老百姓不是专家！整天听你崩出新词！ 1428. 先把用永康路那些街头咖啡馆管管好吧？那么多人大周末不戴口罩，聚集在那里喝咖啡，疫情期间，喝咖啡不能回家喝吗？ 1429. 别折腾老百姓有问题那个姓吴的负责人么？！！ 1430. 昨晚的浦东就是大量的人流聚集现场 1431. 理科生把文科生的精髓发挥得淋漓尽致。 1432. 说是封闭做核酸，又不告诉我们具体时间，去了核酸医护没到，去晚了医护下班，不知道上海是怎么规定的…… 1433. 那就是之前判断失误？ 1434. 上海防疫政策真的没有问题吗，经济真的比老板姓的安全重要吗？顾不上老板姓，还谈全国经济，真的符合当前上海的局面吗？ 1435. 不玩文字游戏行不行 1436. 上海的大方向没错，不能走老路，动不动就全封，上海现在压力很大，其他省市应该借鉴，该建方仓的建方仓，该整改团队的整改，该普及抗原检测试剂盒的普及，该区分无症有症的区分，难道大家想动不动就被封吗？封起来不是王道#上海疫情##上海加油##支持上海# 1437. 控制了这么久咋还要全面爆发呢为啥会爆发呢？哪里出了问题不说一下么 1438. 上海处理这次疫情强弩之末 1439. 聚集和散发两个特点，说明白点就是哪里都很多就完了 1440. 那些个网红专家对说的就是张和吴，下台！滚出上海！ 1441. 日了狗@但斌 1442. 这个吴凡怎么还出来说话？ 1443. 然而我们还是在正常上班，感觉贼危险啊 1444. 全都让你说了呗 1445. 面上爆发是啥意思 1446. 就是说可以举报那些躲在家里一次都不核酸的人吗 1447. 上海有很多區，也有控制得很好的區，比如評論區。​上海zf可以的。 1448. 一点不耽误公司要求上班，让员工直接不准回家，吃住在公司隔离 1449. 无语了，早干嘛去了 1450. 所以瑞幸咖啡总部公司还让上班 1451. #上海疫情#已经是瞎搞八搞试验田了，数据通报有延迟，特殊时期可以理解，但阳性不封楼、报告阴性告知阳性等，也有发生，这样人心惶惶，公信力要st了 1452. 上海的探索失败了，只能这么说，我不喷谁，都不容易 1453. 上海咋搞成这样，疫情什么时候过去，没人管吗？一个多月了 1454. 做核酸，那么多人，队伍中有一个阳性的，得传染一片，封个几天，出去抢菜，一窝蜂的，再传染几个，几轮下来，可不是就这样。今天浦东封了，昨天他们去抢菜，马上浦西也封了，再来一波抢食 1455. 这个东西我们都知道，专家才研判出来，是不是晚了 1456. 求问：面上暴发怎么理解啊？ 1457. 这个吴凡可以闭嘴了吗？自己说话颠三倒四，惹得上海老百姓被骂！一将无能累死三军！ 1458. 就是感觉之前都白封了，真是累啊。 1459. 浦东就是上海，上海就是浦东。 1460. 做的不错，只有两点不行：1.这也不行2.那也不行 1461. 面上暴发是什么意思 1462. 为什么不能想深圳一样封？ 1463. 还有人在为zf嘴硬上海是在探索。一个上海让全国买单一个人让全城买单。国外探索的还不够吗？错了就是错了就算是在探索也是错了，总不能一句探索就把锅甩干净了吧，总不能让上海群众、上海医生、全国群众、全国医生都白白付出这么大的代价，你上海傲慢的一句探索就完了吧。承认错误就好，都是一家人 1464. 什么叫面上？ 1465. 一天天的尽整新名词，少点虚的尽早封闭早就控制住了 1466. 这吴凡哪儿蹦哒出来的？会不会说人话？？ 1467. 还在玩文字游戏，真他粮的服气。 1468. 面上暴发什么意思？ 1469. @绵绵冰豆糕芜湖 1470. 话都被你们专家说了去了 1471. 美好的一天从核酸医护人员碰到我的嘴唇开始崩溃不敢想万一 1472. 感觉这文字就是为前期力度不大的委婉措辞。。。这次的疫情管控实在是。。。 1473. 将帅无能累死三军 1474. 还“面上”，一个劲的捅新词 1475. 开方舱啊，开方舱啊，别往普通医院里塞，@上海发布别死要面子活受罪了。 1476. 问几个问题吧1.为什么22号搞个48小时隔离，但是不做核酸，外卖快递随意出入，2.对于核酸检测点没片区规划。3.在24又突然解封一天，那么为何通知25又要封闭但时间未知4.到了26下午又突然晚上通知封14天。5.核酸报告完全跟不上你们，你跟来了大姨妈一样。探索精准防控？请先把基础打牢固再探索，再创新！ 1477. 前期通知集中做核酸会不会导致密接感染？四面八方的人大家全挤在医院做核酸会不会导致确诊增加？为啥一开始不一刀切 1478. #上海疫情#就别再玩文字游戏了，承认失策，抓紧解决问题才是关键。封了就封的彻底一点，别半封不封的折腾基层，折腾老百姓了！ 1479. 别说的那么委婉、你可以说肯定 1480. 早就知道了！ 1481. 不会说人话了吗面上是什么意思 1482. 上海政府这波操作真的够大跌眼镜，菜价飙高，基本民生都保障不了，就想着保面子，苦的还不都是老百姓 1483. 现在的还不叫爆发吗 1484. 网红上海网红害人 1485. 吴凡说了不能封，她哪里来的自信说结果在变好？ 1486. 面上工程 1487. 别老捅新词了 1488. 上海为什么不能封城？为什么不行，因为上海存承载了不仅仅是上海人民自己的上海，我们这个城市在全国经济社会的发展当中，承载发挥了重要的功能，会影响我们整个国家的经济和全球的经济 1489. 请吴凡闭嘴 1490. 希望上海疫情结束这帮领导班子有人管管，导致现在这么严重影响，无人问责吗？还能稳居高位 1491. 我们小区被封了26天一直静默管理状态。不让出去买菜买不到忽然昨天出现一例阳例这是怎么出来的（居委会天天放人进进出出）就很good不把我们命当命不知道怎么管控的 1492. ？？ 1493. 市政：你造谣，我辟谣；我知错，我不认。 1494. 哈哈哈，跟上海学新词 1495. 让真正为人民生命着想，懂防控的专家上，让这位越俎代庖抢经济学家说话的吴副院长，张网红之类的赶紧下课。 1496. 这批换了吧，害普通人民不聊生，陪玩 1497. 政府睡觉还没醒吗 1498. 整这些花里胡哨的词有啥用？ 1499. 都这样了我居家，我室友还每天出去上班，我真是醉了，有何意义 1500. 总结一下上海疫情可能全面爆发我语文不好 1501. 承认之前的方法不行有那么难吗？ 1502. 面？哪里有面，我抢不到！！！ 1503. 早干嘛去了！！！一个月了！早当机立断估计现在都清零了！前几天新闻还在自欺欺人的说风险排查率在下降 1504. 世界的上海怎么能封闭呢？ 1505. 降低什么流动性？不是说今天开始封了嘛？航班铁路都没封，浦东街上还是人来人往，商店还开着，感情这波疫情是因为浦东浦西互通造成的？上海人民真的是彻底心寒了… 1506. 我百度了一下面上的意思1坟土之上2情面面子3方面 1507. 面上暴发还只是风险？咱就是说，现在是在脚上暴发的吗…… 1508. 不是说拐点要来了吗…… 1509. 能不能说点咱老百姓能听懂的话 1510. 没点文化连话题的几个字也看不懂了 1511. @氯丙嗪mh来听说话的艺术 1512. 脸真疼啊 1513. 没球用，去浦东看看。今天照样有人能出去逛街。买东西。封了个寂寞 1514. 吴凡?上海不能封……浦东，浦西那啥。 1515. 面上爆发应该就是已经筛查出的近万个无症状感染者存在转确诊的风险。 1516. 整这些词冠冕堂皇的干嘛呢？昏官当道，世风日下，这些人毁了多少人的生活，为什么国家不让他们下台，为什么不参与制止？从开始的不管控，任其发展，到后来的2+12，让感染源均匀散播，到最后瞒报感染数字，八点播报封禁，大批百姓涌入街道采购，引起大规模传染可能性，你们在这里说着有的没的，就是摆烂 1517. 你们看看上海现在的超市！哪家不是排队排到爆炸！大家去看我发的视频！上海发布把评论关了，精准呀！ 1518. 上海政府真是垃圾中的战斗机 1519. 目前的奥密克戎毒性已经没有那么强，危重症很少，全国也打了那么多疫苗，是否可以考虑放开？有人说放开会挤兑，请问如果本轮疫情没几个重症的，怎么挤兑？你现在动辄封控，难道对别人不是挤兑？西安孕妇、吉林小女孩、上海护士的事件，是确实因为有危重症导致医疗挤兑还是人为的挤兑？ 1520. 可是人上海不想更果断啊~只想留着底线.不能封..封了就全宇宙停了呢~ 1521. 面上是啥 1522. 上海的领带知不知道打仗怎么打啊？一鼓作气再而衰三而竭，这不应该是人人都知道的兵法吗？这场阻击战一开始战略就有问题，锦江集团、徐汇区卫健委、疫情防控领导小组不需要负点责任吗？？？ 1523. 源头是不是境外的航班，只能在上海进 1524. 我们小区在确诊的情况下还聚集核酸，并且住户是不知道有确诊的！物业都不告知！直到今早通报上看到了门牌号！真新街道，完全没有考虑到群众的安全！只为了上头派任务完成核酸指标！ 1525. 不要脸。 1526. 现在既已采取措施，就放下心来配合就好了 1527. 复旦的不规范“博士”领衔上海防疫的报应，这个也是复旦的。 1528. @GINACc：我们小区在确诊的情况下还聚集核酸，并且住户是不知道有确诊的！物业都不告知！直到今早通报上看到了门牌号！真新街道 1529. 要么严防要么放开，病毒面前没有中间道路。 1530. “面上”爆发？我TM真无语了 1531. 为什么没有领导出来担责呢？为什么市里一二把手都不见了？为什么要我们老百姓半夜抢菜，小孩子只能待在家里个个都变成四眼？ 1532. 面上爆发，解释下，反过来就是里子不爆发，就是好像要爆发的样子 1533. 上海人已经不知道怎么说了，我已经被关的麻木了现在只想早点结束疫情，这一次疫情确实没有搞好，从一开始就拖着实在不行了才关起来，不管怎样加油吧上海 1534. 居委组织的一塌糊涂、前几天还好好的一栋楼一栋楼、今天又开始乱来、服了 1535. 我们小区在确诊的情况下还聚集核酸，并且住户是不知道有确诊的！物业都不告知！直到今早通报上看到了门牌号！ 1536. 还是不够果断！死要面子活受罪 1537. 兄弟们，无症状说明人已经适应病毒了，不用治疗 1538. 扎堆做核酸，抢菜，难道是老百姓的错？ 1539. 我在浦东居家隔离，水，泡面都买不到，让我们等死啊 1540. 小词整得一套一套的 1541. 不要个碧莲 1542. 关于上海疫情的新闻表述我经常看不懂…… 1543. 封不严有啥用啦！闹着玩嘛 1544. 超市聚集懂？ 1545. 外溢那么多地方， 1546. 上海真是造词带师，啥面上爆发。表面爆发实际没爆发是吧？搁这薛定谔的爆发呢。还有啥云南蔬菜驰援上海。我就纳闷了，周边城市吸血不够，开始搁这西菜东送了是吧。 1547. @霉子酱哟 1548. 什么面？方便面还是小面？ 1549. 真tm服了，别再说空话了 1550. 别的不说，上海在语文这方面没得说，绝对头一等 1551. 上海加油，上海挺住 1552. 老百姓都是想好好生活的，肯定会服从安排，只是指挥层真的太不给力了 1553. 差不多的了，还在玩文字游戏，刻在骨子里的傲慢。 1554. 抄作业都不会？狗屁领导组 1555. 我真的不知道上海是怎么想的，这么严重的疫情还一个劲儿的提经济问题？疫情严重起来后那经济能发展的起来吗？你就数数现在上海到底有多少外溢的疫情，别的城市不需要经济吗？不需要发展吗？上海那边人挤人的去买菜都不管理，你不能因为叫上海就真去“伤害”吧？丧害人！ 1556. 全面爆发天 1557. @星星茶刹车：能不能别咬文嚼字了防疫这几年第一次看见这个词说到底还是上海卫健委有文化啊 1558. 赶紧封城吧，一年西安的疫情都是当地阴性过来就阳性了，一波又一波上一波刚完，昨天又一个来从魔都来西安的阳性了，不管全民免疫还是精准防疫都是上海自己的事，别外流就行 1559. 挣钱不要命，别扯全球经济，全宇宙经济崩了，买东西不花钱了也得先好好的活着 1560. “下决心封上一周不行吗？不行🙅” 1561. 配个送菜的图干嘛 1562. 这次词条咋读的那么拗口 1563. 我们公司的园区被封了然后老板要求我们去他家里办公怎么办 1564. 有这时间搞文字游戏不如多做点实事 1565. 自己打自己的脸 1566. 受不了了有什么可骂的啊🇨🇳人永远学不会团结还天天吹吹吹中国凝聚力我真是一点没看见一个两个都是什么垃圾格局微博真的是乌烟瘴气 1567. 少说没用的，多干点实事 1568. 为何上海没人问责 1569. 有人要把上海变成xg这下满意了？ 1570. 一天一个说法，说的一套一套的，呵呵 1571. 加油 1572. 把宣传作秀的精力用在防控上吧，别整天宣传什么防疫网红，什么优雅防疫，什么最小封控了，做点实事可以吗？ 1573. 吴凡不简单 1574. 造新词很厉害，百般掩饰什么呢？ 1575. 倒春寒有点大了 1576. 别搞文字游戏了好吧 1577. 没捷径，就是减少流动，筛查再筛查，凭核酸报告流动，找出社会面感染例，剔除隔离，核酸结果一定要快 1578. @上海发布 1579. “呈现区域聚集和全市散发并存两个特点，存在面上暴发的潜在风险”🐮啊🐮啊，遍地都是的新说法，不愧是国际性大都市呢，玩起文字游戏那真是一套一套的 1580. 早封控现在估计都已经恢复正常生活了，从去年开始，一直拖到现在还在持续，周边的杭州宁波苏州跟着倒霉，老百姓也是跟着遭殃 1581. 现在想快能快起来吗？医疗资源还够吗？您这折腾了两周多，一块一块的封。 1582. 希望3/28～4/5鸳鸯锅之后局面可以得到稳定 1583. 为什么总是医务工作者发言说这些？！ 1584. 买不到菜我只想活着 1585. 面上爆发什么意思，听不懂 1586. 有没有一种可能，上海和长春根本就是放开了？？为全国放开先做个试验？？？ 1587. 早就爆发了，只不过离更大的爆发又近了一步 1588. 这样抢菜，核酸不是增加了交叉感染的风险 1589. 上海是国际化的，要和国外一样，全民免疫，根本不听话 1590. 网红张呢一顿带节奏飞起现在出来走两步？ 1591. 下级县市一刀切的政策都是为了一线城市的经济一线城市自己作死要搞精准防控，失败了，倒霉的还是下级县市我们一般都是一边骂一刀切一边下楼做核酸 1592. 南京走在上海前 1593. 政府无能啊 1594. 呵说那么多结果都是废话要你出方案不是叫你开虚会！一户户发罐子唾液测试统一收集后检测再我看来就是目前最好的解决方案！聚集做核酸这踏马要交叉感染到设么时候！ 1595. 猫眼看海上酒囊饭袋出骚招一纸封文万事消黎民百姓多无奈哮喘心梗人命凋百万人民齐买菜聚集一团情况糟期盼明朝開天眼一帆風順上海滩 1596. 清零本来就不科学，控制致死率加强疫苗才是第一位 1597. 早些这样管控就不会出现这样的局面了，有人应该为此负责！ 1598. 没责怪上海的意思，只说疫情，不只是全市散开而是全国散开了。这几天封起来辛苦了，加油，希望这次能彻底控制住 1599. 昨天外溢湖北一例，汉口站下车做的士到鄂州，今天武汉又开始鸡飞狗跳的流调该说什么好？为什么外省到现在还不是高危？ 1600. 面下，面中，面左，面右还没事了，面上爆发用的很严谨 1601. 不要再玩文字游戏了 1602. 乌莎保得住不？ 1603. 说话的方式能不能简单点这几天看到好多新说法 1604. 前几天不就这个吴凡说上海不止是上海人的上海，影响世界经济不能封？现在又变脸了？反正你家里人不会得呗 1605. 还在玩文字游戏，上海这帮老爷是要把全国人民都拖下水吗？怎么还没有人被处理，问责制度呢？#上海疫情##上海新增50例确诊3450例无症状# 1606. 还有刚才的，切块 1607. 上海zf这管理早干嘛去了？一开始就马上封会这样吗？一群饭桶 1608. 实在是人员不够，医院也纳不下源源不断的患者了，有的患者确诊了在小区隔离着还能出来丢垃圾，能不传染吗！ 1609. 这不就是说芭比Q了吗 1610. 在重点区域。现在每次测核酸都是按单元喊出来排队，每次排队1小时以上，很担心，这不也是人群聚集吗？ 1611. 谁来解释下面上啥意思？语文不好 1612. 别tm天天整点新词搞文字游戏了，现在上班提心吊胆怕被传染，吃饭担惊受怕怕吃了这顿没下顿，还好意思自称大都市宇宙中心，不嫌丢人？危机处理能力也太差了！！！ 1613. 前两天你可不是这么说的 1614. 疫情三年，处理思路肯定要变的，是日子不过了就管住疫情，还是寻求对居民生活工作都影响更小一些的方式，没人做过的答卷考不了满分，希望试错后给更多城市积累经验，加油啊，上海！ 1615. 放过其他地方的人吧，刚解封，真的会谢 1616. 狗官文字游戏玩够了没？ 1617. 意会这玩意儿应该纳入学校教育里，一天天的越来越看不懂了 1618. 我就想问防疫做成这样，哪些领导要追责？ 1619. 说的废话 1620. 可能，嗯，SH有一些骄傲在身上吧 1621. 继续折腾老百姓！继续宣传自测盒！老百姓有饿死的权利！中小型企业有倒闭的权利！ 1622. 能不玩文字游戏吗？？？ 1623. 大上海吃螃蟹扎着嘴了 1624. 上海疫情日渐严重，这是所有人都不想看到的。我们希望所有地方的疫情都可防可控，但是这确实不是以人的意志为转移的。国家一直在说要坚守动态清零和精准防控，固然有它的道理。但是我觉得应该有自己的思维和判断，审时度势才行，目前来看动态清零的策略似乎在一点点的碎裂。 1625. 早干嘛了？？？ 1626. 取乃买地。我终于知道了，不是谎报，是不作为根本不知道当时疫情已经泛滥 1627. 又是这个吴凡，前两天说不能封城，影响世界经济之类的。现在说要降低流动性，要封城。我他妈笑了，这不是耍无赖嘛，还得是你啊！ 1628. 上海市民一周前就帮你们判断好了，你们专业的到现在才清醒 1629. 上海这一次的操作从头到尾笑死了 1630. 还不管管啊？不管管你别让上海的出来祸害别的城市啊 1631. 面上是什么意思？还有里子？ 1632. 疫情有完没完有完没完有完没完有完没完 1633. 不精准防疫了 1634. 那天看报道，医学院教授发言内容是上海经济发展，一时语塞了，在医言医，在经言经好吗，这种时候就不需要跨专业人才指点江山了吧//@兔斯基-tsj:就这个吴凡前两天还在大谈上海封城对国际国内的影响呢，今天又谈封城的必要性，其实前两天上海的疫情数据已经高位运行了，吴凡们的预测研判能力也太差劲了吧 1635. 全面反过来面全 1636. 封城也要保障生活物资供应啊！买不到菜啊！ 1637. 还面上？玩文字游戏不就是全面爆发吗？ 1638. 什么？脸上爆发？ 1639. 所以上海能别出来吗，我们一个三四线小城市昨天上海来一个，核酸直接阳性，害我们一个市小区都封门外卖都不可以了，全员核算，真是无语 1640. 怕你还看东海上的国际货轮 1641. 求你了，别爆发了，快点一刀切，周边城市遭殃 1642. 别的地方说加油，沪国好自为之吧 1643. 核酸检测人挨着人排长长长队算不算聚集呢 1644. 我男朋友在浦东，小区被封了10天，居委会没有消息说是有密接还是其他情况，然后昨天晚上他的隔壁小区有确诊的都解封放出去买菜，他们小区什么情况没有，继续被封…… 1645. 喝咖啡吧，多优雅，喝红酒吧，多浪漫。 1646. 转发微博 1647. 继续折腾老百姓！继续宣传自测盒！ 1648. 整天套话，官话。说保障供应，完全没有保障，管理混乱，筛查混乱，应该要问责。 1649. 我觉得上海这波挺糟糕的 1650. 有本事就别采取最笨的措施：封城 1651. 全市爆发？你确定吗？现在全国不都是了 1652. 管控不严流动性太大国民不积极配合隐瞒行程这是基本原因 1653. 啥叫面上爆发？ 1654. 国际大都市不是不封嘛？ 1655. 这会儿知道封了 1656. 有人知道常州的事吗 1657. 局部爆发 1658. 这次抗疫策略完全看不懂，3小时买菜简直就是大型聚集现场。看来不是以前防疫有多优秀，只是运气好而已。 1659. 现在普通老百姓和抗疫基层被折腾的多惨！吴凡的发言能不能务实一点！前两天绝不封城的也是你，现在有必要果断分块的也是你！政府性发言这么随便的吗？ 1660. 上海政府做的没错，上海要是一开始就搞封控，中国经济就垮了，全中国人民都会引发恐慌，就不只是我们上海市民要抢菜了。上海是要慢慢的创造新的防疫模式，与国际接轨，慢慢开放，不可能永远不开放吧？每次都做全员核酸自由都没有了！ 1661. 上海脸都不要了，平时死命黑外地人。出了事不封城把阳性病人往苏锡常送，邬惊雷还有脸说上海自己解决？周边城市跟着你上海倒霉？ 1662. 到现在才发现？？？这也真是后知后觉了，从隐瞒那天开始就注定了 1663. 连隔离点选址这种最基本的都要搞出问题。所谓专业性怎么体现？ 1664. 正常的城市运作和民生保障体系预案做得拉胯了。这么大一个超级城市，面对传染力如此快的奥密克戎，为什么不是社区统一采购后，按社区购买呢？刚开始可以说措手不及，但是经过这个把月了，完全不应该出现这种局面。这抢菜抢得如此人潮涌动，密集感染风险实在太大！苦的是上海老百姓和基层啊！ 1665. 我们这里上海回来一个人，阳性，封了一个县城。 1666. @伦萨东京柑橘老实在家呆的 1667. 今天小区核酸尽然是两个人并排挨一起同时核酸。 1668. 又发明新词了？ 1669. @detectiveMARVIN 1670. 全面放开试点城市(个人感觉。狗头护体) 1671. 面上 1672. 好失望真的感觉过去的一段时间在被当猴耍 1673. 又是你吴凡。东海货轮不管了吗？ 1674. 好想知道上海不能封城的话谁为疫情外泄买单？ 1675. 长春控制的很好，有一些区控制的特别好，尤其是评论区 1676. 小区开车出入随意，非小区人员直接开门进入，爬墙出去溜达晚上回来的，核酸聚集性没有制度的，这些种种情况去哪里全面清零？ 1677. 这个标题让我怀疑自己语文水平 1678. 其他地方超过50都赶紧封城上海还是上海挺了这么久大城市就是不一样 1679. 张医生现在不怎么敢讲话了 1680. 离离原上谱 1681. 现在要把我们闵行封一个月了！老老实实封它全城一两个星期快速搞定不行吗？！死要面子、百姓活受罪！ 1682. 张医生昨天不是说拐点快到了吗 1683. 我着实不明白开通三小时限时抢菜是什么决策，这不就是大型聚集现场吗？之前的隔离一波抢菜全部作废，看吧，后面阳性会越来越多 1684. 天天造词是吧，都有点看不懂了 1685. 家人们，咋们坐等是哪一位大官先写辞职信 1686. @夏天不用穿秋裤 1687. 你这样临时封，让大家出来抢，不就是放任交叉感染吗 1688. 当断不断，反受其乱 1689. 各种文字游戏 1690. 啥叫面上暴发？ 1691. 早全面爆发了，附近几个省都是上海回来的，你装不知道哪？没人说你不代表你没问题，早控制能变这样吗 1692. 为何不追责呢？还有从上海来的人到处跑，管控措施在哪里？ 1693. 精准文字游戏 1694. 给我一种其他国家防疫政策的感觉 1695. 就是发面的意思！酵母发面你们乡里怎么能不知道呢！ 1696. 一会封，一会又放出来，恐慌性的买菜聚集，没问题的都要有问题了，基层居委大多数不作为，强制性差，靠自觉，真的摆烂 1697. 上海不能封，千万不能有序流动，动态清零就好 1698. 重头再来，玩呢 1699. 这个吴凡不是前两天还说上海不能封城的吗一天天竟搞新词汇啥话都让他说了 1700. 吴侬软语？ 1701. 话能好好说么 1702. 承认吧，何必如此咬文嚼字，就是局部失控了 1703. 面上爆发…面下没事…？ 1704. 精准控评 1705. 各平台骑手成为沪上顶级男团，最难约的男人。 1706. 瞎搞。 1707. 就是不想全部封禁，非要这边封完那边放，互相流窜感染，也不知道咋想的，GDP比命重要？还是说实在是能力不行，无法做到全封禁的人员物资周转，安排不上？ 1708. 大无语、真是害苦了老百姓、、、 1709. 把苏州给害了我朋友在那边停工都停了两次了才上班几天 1710. @zhizhi不倦。 1711. 砖家呢？出来继续叫唤啊 1712. 升级防疫策略，周边县市大力支援，有什么需要赶紧说，别藏着掖着了。一天天的整什么新词，直接说担心会大规模爆发不就完事了嘛，给老百姓一个有心理准备的机会，不至于摇摆不定。 1713. 什么叫面上也不解释一下 1714. 深圳封城了，紧接着传言上海封城，督导组来了仍旧不让封，这的确是让上海寻求一个解决办法。可惜失败了，但不代表封城是最好的。就像数学枚举法也有效，但终究还是要找到最快的。总说自以为是，但空海港各大办事处总部聚集，怎么能轻易停摆 1715. 头条新闻可以啊，不控评 1716. 这么多了还不算爆发？？？还存在，摸着良心 1717. 赶紧的吧！！！！别祸害别的省市🙃🙃🙃 1718. 潜在风险算什么，哪有东海上漂着的货轮重要，潜在风险与2490万上海市民的安全和全球经济相比，屁都不是！！！吴凡，我理解的没错吧 1719. 上微博学语文 1720. 很想说一句上海加油，但zf骚操作掩人耳目。“面上”又是什么新词汇？表面上暴发吗？两周前及时补上封控漏洞也不至于祸害那么多省市。 1721. 吴凡打脸能不能道歉 1722. 叫兽 1723. 前半个月在搞什么玩意儿你现在给我说这种风凉话啊脑缠啊 1724. 本来隔离不焦虑，上网看网友骂上海，看的好焦虑 1725. 啊，为啥封闭的小区社区不送菜？我们这里封闭的都是每日每户配送蔬菜水果肉蛋禽啊 1726. 前天还是你俩（吴和张）可说不能封，还打断了指数增长，今天又是封又是全面爆发的 1727. 什么是面上爆发？ 1728. 所谓超一线大城市管理就是这个水平了 1729. 沙比 1730. 存在谁造成的呀，贻误战机 1731. 这位吴凡副院长昨天刚说什么来着？不能封城 1732. 咋了？上海不是精准防控的吗？不应该啊？是不是搞错了？ 1733. 不精选评论了？ 1734. 香港和上海两扇资本进入天朝的大门被堵死，内忧外患，为极端事件的爆发积蓄洪荒之力。极端气候，地质，人文，此时的联动效果，立竿则见影。 1735. 是在给特效药铺垫吗？辉瑞自己国家死亡率那么高都不用，要套我们医保肥油吗？ 1736. 需要来一个中文翻译 1737. 汉语表达意义的博大精深什么“面上”？爆发就爆发好好面对别在玩游戏玩文字游戏 1738. 是不是这个吴凡说的不能封？封了全球经济受影响，他到底是研究啥的 1739. 上海不能封啊，世界中心 1740. 死鸭子啥硬 1741. 现在真觉得还不如躺平，起码没病的人还能活 1742. 话都被你们说完了 1743. 继续放开抢菜啊 1744. 全市散发？ 1745. 和中国修路一个道理这边修好那边坏那边修好这边又坏搞不好的 1746. 知道吗因为上海的疫情本来苏州已经可以开学的也开不了而且外溢了多少城市不清楚吗 1747. 真果断 1748. 砖家 1749. 倒春寒够冷！ 1750. 隔三差五的造出新名词，文化功底真好 1751. 。。。。。 1752. 吴凡说话不要信 1753. @欧阳十七姨：@欧阳十七姨：全宇宙的中心，要坚持住不然全国都要。。 1754. 看成吴一凡了 1755. 难道一定要出现三维空间爆发才不会唓唧吧啖？ 1756. 面子上下不去 1757. 面上爆发，讲人话，全面爆发 1758. 别造词了，有这功夫还不如把心思放在防疫上 1759. 我读了半天，都怀疑我以前的语文白学了，什么叫面上爆发可真行啊。 1760. 那么多专家防疫团队，香港那么小的地方，之前天天被骂 1761. 相信张网红，没问题的 1762. 才发现？ 1763. 就像欧美对俄乌局势的胡说八道一样，新闻会说谎，但战线不会……摸摸良心，你们心里装的是上海的老百姓么？你们心里的经济账是老百姓的经济账么？ 1764. 上海有点东西，语言大师！ 1765. 我要喝咖啡 1766. 早点面对现实多好，吴凡前几天淡定说不能封城，今天就说要爆发 1767. 晕，你突然封浦东让大家抢菜就是神助攻啊 1768. 面上暴发？又是啥新名词？打脸被打肿的意思？ 1769. 这啥新词汇 1770. 吴凡 1771. 真会造词 1772. 我们小区现在确诊的被封在地下车库，因为没有人采取措施带走病例隔离！ 1773. 哪个地方也逃不过，继续吧 1774. 赶紧封城行不行 1775. 这位吴凡真的看不懂在讲什么？怎么复杂怎么讲，故弄玄虚，现在就是爆发了呀！没有全员核酸的情况下3000多无症状！你那么自信不封城的，现在呢？专家组出来发言到底是口嗨还是研判后的结果？坍台 1776. 那里儿啥样呢？ 1777. 能不能，全部封，西安受不住了好几次了 1778. 我还有一个月多月就要生物地理高考了疫情求你放过我 1779. 下一个热搜：上海人民有多喜欢喝咖啡 1780. 不是说上升指数掐断了么？ 1781. 撤职查办 1782. 直接摆烂 1783. 终于承认了 1784. 有没有官方解释一下什么叫面上 1785. 目前脱下口罩随地吐痰如何防控？如何严惩？ 1786. 赶紧封城 1787. 用得着放开去买菜吗，上海人口也不少，是都不愿意去志愿者送个菜，政府么也给志愿者发点辛苦费，大上海又不缺这几个铜蹀 1788. 不是不是，网红不是才说拐点来啦吗，这咋又爆发了啊，到底听谁的 1789. 哈哈 1790. 每天买什么拐点热搜有什么意思，加上管控力度才是最重要的，疫情打的不是舆论战 1791. 小区有确诊都不封小区只封楼 1792. 可别在给上海遮羞了 1793. 可以下台一大批领导了 1794. 步子太大扯着蛋了 1795. 筛查发现已阳人员，及时隔离，恢复正常生活，给上下一个交代。然后不要折腾了，也别扯什么潜伏期了。这么一轮大规模的样本应该也是够够的了，真的求求全国都不要折腾了。 1796. 什么话都给你说了 1797. 所在楼被封了，居委会通知是密接，上海发布那边是确诊，然后让每户做核酸，集中下楼做，这样不是再次集中密接了嘛？说是一户一户做，我在群里问叫一户下去一户吧，还有人说，肯定的，集中不都成密接了嘛，然后等通知做了，事实就是集中在楼下排队做核酸。 1798. 每天都搞些新词 1799. 你封浦东，人家去浦西，你封浦西人家去浦东 1800. 死鸭子嘴硬，干嘛不学学我们大深圳在那死撑 1801. @詹姆斯得分超科比开心伐？ 1802. 大城市就是大城市，用词都是我们这些小地方用不来的 1803. 然而浦西今天还在坐地铁上班呢 1804. 随便爆发，反正没事 1805. 这还只是潜在风险？不都已经摔脸上了吗？不行了不行了！赶紧喝杯咖啡压压惊 1806. 之前的精准防控，就是为了尽量不出现现在的局面，尽量不影响百姓的正常生活，感谢尽力保护百姓的领导，虽然没有成功但你们的努力还是有很多人看到了 1807. 什么叫在原有政策上进行优化别优化了这叫整改 1808. 草菅人命 1809. 这时候了为什么要集中抢菜，为什么不能按社区专门的服务人员送货上门深圳有个现成的模板抄都不会吗 1810. 默默 1811. 太让人失望 1812. @我愉快_ 1813. 别霍霍到山东 1814. 这人前后短短几天两种说辞，不知道的人还以为她有精分。 1815. 现在才发现吗？你们追着病毒跑的时候就该预料到了！ 1816. 那人家总要买菜吧真不知道上海政府怎么想的 1817. 上海领导层有没有换了？？ 1818. 网红建议上海，应针对「疫情规模超出现在5倍或者10倍」做好预案，包括：实战演练、加强救治体系建设、加快抗疫科技平台建设等，同时应做好对更基层医疗机构的培训和分级诊疗的培训及物资准备 1819. 所以，上海赶紧管管个别公司，不要再让我们冒险去上班了好吗 1820. 官腔话语创新能力令人惊叹 1821. 砖家🐮🍺 1822. 防疫搞成这样？不追责吗 1823. 封8天，数字超过10万，怎么办？正确的方法是无症状不报，只报确诊病例，但是无症状必须居家隔离，自行监测治疗，把所有无症状全部居家隔离。化整为0，个个击破，不要把大量人力物力资源都浪费在无症状上，切断无症状感染的路径。 1824. 不是说不封城么打脸了吧 1825. 某网红张院士不是说上海拐点了嘛？ 1826. 疫情防控最好的评论区今天也躺平了？ 1827. 早干嘛去了犹犹豫豫搂不住了才敢封城完蛋玩意 1828. 请加大防控管控力度 1829. 上周说的已经达到解封标准呢？？ 1830. 还是吴凡？？？前两天说的国际大都市，今天自己打脸 1831. 是面上，还是面子上？ 1832. 日尼玛别玩文字游戏了晦气玩意儿 1833. 家里有宠物的，保护好自己宠物 1834. 香港已有先例，为什么没有疫情大规模爆发的紧急预案？乱糟糟苦了老百姓！ 1835. 风控上海只需进不许出，在上海搞个全民免疫，试点！就像当初在深圳搞特区那样的！不然这次通过风控搞好了，三月后又来了，还照样风控吗？如此这般，病毒搞死了，病毒的载体人也不要活了 1836. 这都啥词啊，听不懂了 1837. 这特么谁都明白，那前几天嘴硬什么呢 1838. 还吹呢江桥市场为什么检测到风险不落实宿舍里的人反而把他们全部赶走？里面的人全部露宿街头是你想看到的还是周边小区开始遍地开花是你想看到的？ 1839. 又玩文字游戏 1840. 中国语言博大精深，这回玩明白了 1841. 还以为只有我们吉林防疫差劲呢，原来一线城市也是一样 1842. 不是嘴硬吗？ 1843. 论不要脸还是这个数第一 1844. 无语 1845. 面上，都学会了吧 1846. 疫区，天价菜，喊加油，吃不上饭，喊加油，看不上病，喊加油，不能工作还不上房租房贷车贷，喊加油，都快死了，还喊加油，我不愿做站着说话不腰疼的人 1847. 求求你们，别再口嗨了。 1848. 把“潜在”两个字去掉。。 1849. 上海创造了不少新词汇 1850. 真无语… 1851. 当一个社会什么都不做，只做一件事的时候，呵呵 1852. 什么叫潜在？那现在算什么？ 1853. 所以为什么不封城 1854. 严防严控，别外溢了 1855. 面上是啥意思啊 1856. 语言博大精深啊//@赫魯暁夫:全面不叫全面，叫面上//@NextSat6am:那不就是全面爆发的意思吗 1857. 面下的不能说//@赫魯暁夫:全面不叫全面，叫面上//@NextSat6am:那不就是全面爆发的意思吗 1858. 全国各地都比sh知道sh疫情的严重性 1859. 吴凡:上海不能封城！！！… 1860. 肯定存在这个风险啊解封的聚集买菜不知道谁想出来的 1861. 还是等下一个变种更轻的时候再放开吧 1862. 每一个字我都认识，但是就是看不懂 1863. 封就完事了 1864. @王老师家在外地 1865. 呵呵领导什么时候下课？别再玩文字游戏了……闵行被关了多少天了，啥都没发过，全靠自己想办法，不然真得饿死。 1866. 大家都配合你鸳鸯封了麻烦你这次能不能弄弄干净啊谁天天吃饱了陪你们一会48小时一会无限➕2又一会鸳鸯封的真当老百姓各个都闲的没事吗 1867. 为啥不封城呢 1868. 浦东发布的保障菜价和我们小区居委组织的蔬菜盲盒比贵太多了。只是不知道居委还能坚持多久。 1869. 我觉得下次还要再打脸今天说的 1870. 直接承认精准防控失败，不丢人，真的！！！为啥就是不敢承认呢？？早点封城，也不会有这么多呀 1871. 有人乌纱帽不保了 1872. 封城吧，真的。不要得不偿失。要看长远的利益。上海加油 1873. 张文宏前天不是说已经切断了指数上升链了吗？不是说拐点条件已经形成了吗？搁这儿自己骗自己呢？？？？？ 1874. @天很蓝tianhenlan 1875. 这个吴凡你别说话了 1876. 谁还看不出来了？ 1877. 呵，无语 1878. 不就是你们号得，还咬文嚼字，害死我们上海人啊！ 1879. 面上，还米上呢 1880. 还要你们说，还是你们现在才发现？不是一直在爆发？ 1881. 集体免疫刻不容缓 1882. 专家发布情况很清晰，一线工作者万分辛苦，但是篱笆扎紧的环节很多，疫情当下，一视同仁地管控，聚集性的管控要立即管起来，于公于私祈盼上海尽快好起来 1883. 早点封城是不是不会那么糟糕😰 1884. 吴大主任不担心地球停转了吗 1885. 给大家讲个笑话上海精准防疫 1886. 妈的天天说废话 1887. 天天搞这些文字游戏有意思吗 1888. 假新闻，一定是 1889. 说“上海不能封，世界少不了上海”的也是她吧 1890. 我还疑惑🤔了一下，什么叫面上？ 1891. 的确关在家里也没事做核酸的时候一堆人感觉被感染的多 1892. 论中文的博大精深 1893. 这个吴凡是那个只顾经济的吴凡吗 1894. 已经爆发了吧？还风险潜在？ 1895. 咱就是说真的这个时候应该考虑到群众而不是经济问题了，南京好不容易好起来又来一例上海的阳性 1896. 国际化大都市哟不得了哟 1897. 新词汇？ 1898. 多干实事，少整新词 1899. 48小时不够吧至少也得两次核酸间隔72小时吧，我们这里居然间隔没有12小时的，前天昨天今天都在做核酸普通人真搞不懂精英是咋想的哎想活着都那么难啊 1900. 防疫策略我相信官方，这个新闻对上海的防疫策略的选择作了科学的解释，网友别胡乱喷了，没有谁能做出完美的选择，相信中国政府会做出相对最可靠的安排，这是历史证明了的，现在也是这样 1901. 在上海的人能不能自觉点，不要往别的省市串了，谢谢您嘞 1902. 上海不能封，毕竟支撑着各种经济，上海封了影响太大，地球都不能转了 1903. 那可封不得 1904. 整个乱套了 1905. 名片城市原形毕露 1906. 华山北院阳转阴出院的一车人，被拉去唐镇维也纳隔离7天，298一晚，100一天的饭钱。打疾控中心电话说去了解一下，之后无回复。打100电话，说他们不管。走又不能走，不办入住不给上厕所。为什么同样别人隔离安排住别的酒店不收费！！！ 1907. 这么久就分析出个这？ 1908. 阻断传染源，从我做起。 1909. 上海领导该换人了 1910. 非必要不来沪，非必要不出门不离沪。防护三件套五还要都做好 1911. 怎么会爆发呢不允许啊 1912. 好家伙，前几天刚说上海可是堂堂的中国经济中心，不能封要动态清零!可现在呢?笑死，为了GDP拿百姓的生命开玩笑! 1913. 哪个地方的疫情暴发不是这两大特点？说跟没说一样。要深究一下，为什么同样是输入性疫情，北京、深圳能控制得很好，而上海反而规模性反弹暴发？ 1914. 跟着上海学新词儿 1915. 现在都这么严重了，需要被问责。 1916. 我觉得悬，身在吉林，奥密克戎传播力真的强。天佑魔都。 1917. 早趁1000例以下时候，封城10天，测5轮核酸，就可以下来，偏偏要所谓精准防控，最后看起来天天3000例，，估计能持续到夏天，持续三个月，折腾啊！非要标新立异！真是不会算账啊！ 1918. 別玩文字游戏了 1919. 上海凉凉了这个确诊人数 1920. 害，别整那些没用的新名词了，赶紧从高高在上的自我陶醉落到地面来做点实际的工作吧 1921. 我们这边的水果蔬菜市场都封了，家里没囤货，怎么办 1922. 还有这个！！望博主加上🙏🙏¡评论配图 1923. 我想起我爸就是新冠最猛的那时便血赶上湖北封城，没法去医院检查，等到解封去医院一查，结肠癌晚期。不到一年人就没了…… 1924. 变了味道的防疫措施，一而再，再而三的突破我们的底线，你可以得其他所有的病死，哪怕不是病死，隔离跳楼，被辞退跳楼而死都可以，但唯独你不可以得新冠，哪怕它是轻症不至死，也不可以 1925. 上海真的牛皮…已经全线崩溃了。成都上次疫情在控制下，我们专门有医疗救助组还能处理联系医院救护车专门接透析，孕检，各类发烧，甚至通乳都给处理了。上海应该是太多太多已经崩溃了整个系统。 1926. 希望能帮到大家我的经验我妈妈乳腺癌患者需要放疗现在已经成功到达医院了方法先是直接找居委会让居委会上报到120一定要说的严重一点并且要态度坚决不要唯唯诺诺我妈搞了一个星期才搞定说身体很不舒服然后提前联系好医院打电话问是否接诊现在一般都是接的 1927. 疫情就是一面照妖镜，平时说的再好，等事情来就能看出来这个城市的应急能力！ 1928. upup 1929. 我来告诉政府怎么做，社区人员分工，一个人管理5～10栋楼。每一栋楼拉一个群，前两天一直发信息艾特居民把需求写清单发过来（日常用品必须提前考虑三天）统计好发给采购，采购买对接发货和物流。老人不会用手机转门电话对接记录。分管的人还要统计基础病的人员及必须治疗的时间提前安排。 1930. 我看基本都是定期血透，在官方还没救援的时候，有没有专业医生科普一下这几种病得应急处理方式，什么姿势，什么外物，什么手势揉捏，能够延缓病患痛苦，或者暂缓病情（我不懂医，希望能有懂的科普一下） 1931. 昨晚看了一晚上，热搜掉到28了已经…… 1932. 要是不及时做血透…… 1933. 一刀切不应该切掉病人的生命线，到这个地步真的很悲哀 1934. 老百姓的人权何在， 1935. 🙏 1936. 🙏upupup 1937. #上海疫情#救助#浦东疫情# 1938. 哎民生艰难啊 1939. 尿毒症靠透析维持生命！每个星期不及时透析，哪怕拖1~2天都有生命危险！呼吸困难！人巨难受！！！别喝水，如果自己能吐就吐掉一点，人会舒服一点……虽然很残忍但真的人会舒服一点的 1940. 主页已经都转发了，可是热度还是不够好怕帮不上他们该怎么办 1941. 人命关天忘转发上海加油💪 1942. 浦东潍坊新村街道竹园新村：1小区很多阳性病人没有收治，至今仍在楼内；2阳性封楼的环境至今没有人消杀，这点是最离谱的！小区持续还有新增阳性！3阴性的人也要经过没有消杀的楼道下楼去做核酸，而且是扎堆。这样的做核酸真的是为了防疫吗？让阴性配合防疫的人民群众也感染吗？ 1943. 扩 1944. #浦东疫情#@上海发布#上海疫情# 1945. 还有个视网膜脱落的，救救他们吧 1946. #浦东疫情# 1947. 帮扩，希望能有真实落地可执行的动作#浦东疫情# 1948. 有的已经解决了，可以删了 1949. 帮扩 1950. 这没用啊病毒传播开来这些人到医院基本就都感染就……，为啥老外死的人都计算在新冠就是这种去医院看病就感染了抵抗力弱的基本没救术后的生孩子的都有在医院感染去世的 1951. @氛围帅哥大赏 1952. 🆙 1953. #浦东疫情#若有余力，请多关注身边的老人，孩子，和这些急需帮助的弱势群体。🙏 1954. 帮扩#浦东疫情# 1955. #浦东疫情#🙏🙏🙏🙏 1956. 真的离谱啊 1957. #浦东疫情#转！ 1958. @人民日报@上海发布@澎湃新闻怎么的，现在除了新冠确诊的人命值钱，别的病人人命不值钱哦？？？ 1959. 不知道这些因为新冠防控而死亡的人有多少？估计永远都是一个迷 1960. 帮扩，太能共情了朋友的父亲去年年初病重，因为疫情防控措施，辗转几个医院都被拒收、被踢皮球，最后因为没有得到及时医治而去世，他甚至没能去见父亲最后一面。这样的事情发生在身边真的很让人寒心，“他本可以活着”是终生无法弥补的遗憾。 1961. #浦东疫情#政府有空评论精选不如睁开眼 1962. 不因新冠死，却因新冠防疫而死。 1963. 浦东人口本来就很密集，受浦东新区卫健委管辖的只有7家医院。浦东新区范围的仁济，六院都不受浦东新区调配。在本来血透病人各家都是饱和的状态，平时没有疫情的情况要做到晚上十点。现在浦东浦西管控，七家医院已经超负荷运转，为什么还不让市属医院一起协调。 1964. 不理解上海都乱成这样怎么上海政府还是不作为不解决问题，是政府机构没连网吗，还是听不到人民群众的呼喊，要是不行就下掉，喊能者上 1965. 真不敢相信这是经历了武汉疫情两年后的上海 1966. @共青团中央@紫光阁@侠客岛@中国长安网除了蓝v广场控评，这些老百姓家属求救还有人管啊？头上有青天否？ 1967. 疫情出现前不就应该准备好预案吗，比如可以准备专门的大巴车将需要特殊医疗的人群固定时间拉到固定的专门医院医疗，闭环管理相信老百姓会理解的，但是现在，只看到一片混乱，明明跟全国没差别，却非要搞特殊，这下完了，全乱了，希望每个普通人都挺过这一波吧 1968. 救救，别看不见我们@央视新闻 1969. 我真无语，为了防疫就不顾人命，上海那些管理人员懒政 1970. 应该先解决官员不行就换人什么玩意 1971. 这个望博主加上¡评论配图 1972. @烟眷眷i服了服了 1973. 救命🆘 1974. #上海求助#逆境退散 1975. 疫情赶快走🙏🏻看到图中的老人跟我外公外婆一个小区的就很心疼，希望早点得到治疗 1976. 希望看到 1977. 早日收治怎么办啊 1978. 看到这些太难过了，遇到问题没人解决，一刀切的政策 1979. 魔都就不能放下那所谓的面子求助一下内省嘛，这样下去苦的是魔都人民 1980. 能做到的只有帮忙转发无力 1981. @月白僵尸 1982. 上海已经崩溃了吧 1983. 浦西是不是上海的富人区啊？ 1984. 疫情不可怕，可怕的就是这样一刀切，切出来的人道灾难和人心惶惶。就继续作吧。 1985. 希望上海领导看到吧，太可怜了 1986. 扩扩扩，有疫情的时候，有基础病的人真的太苦了 1987. 第一个解决了 1988. 上去 1989. @想去慈宁宫烤面包唉 1990. 过度挤兑资源了 1991. 转转 1992. 刚爬楼梯做完核酸（电梯是不敢坐了人满为患）美好的春日仿佛身处无间地狱人世间就想好好活着怎么就这么难 1993. 有关部门看到会不会给你删号 1994. //@十月十七柒柒柒:#浦东疫情# 1995. @人民日报 1996. up 1997. 苦难之下，一览无遗的无能！疫情快些过去，祖国繁荣昌盛！ 1998. 转了转了 1999. 上去上去 2000. 疫情如此严重的情况下，有限的医疗资源和接连不断急剧增长的患者必然会产生巨大矛盾，那些本该得到救助但却陨落的生命都是时代的眼泪。作为大环境下的小小个体，能尽一份力是一份力 2001. 廖晨雨是男的这位u博主也有人对接了，麻烦up编辑一下吧，留更多的信息空间给接下来的求助者，人太多了 2002. 上海抗疫求助超话或许还可以加上这个超话 2003. 比烟火永恒的博主已经有人对接，up编辑一下吧 2004. 转发，人在北蔡，完全能理解 2005. 愿平安🙏 2006. 微薄之力转发扩散希望政府早日拯救人民心痛！祈祷上海早日清零 2007. 不敢相信这是上海，突然感觉之前西安被骂的好冤 2008. @省月 2009. 要我说这一点上海真的比不上南京了 2010. 提前买个大喇叭，不行就录音挂窗户放 2011. //@狐狸三千是只猫_:希望能帮到大家我的经验我妈妈乳腺癌患者需要放疗现在已经成功到达医院了方法先是直接找居委会让居委会上报到120一定要说的严重一点并且要态度坚决不要唯唯诺诺我妈搞了一个星期才搞定说身体很不舒服然后提前联系好医院打电话问是否接诊现在一般都是接的 2012. 恶意求助 2013. @澎湃新闻@上观新闻 2014. #上海疫情#。#浦东疫情# 2015. 张文宏呢？ 2016. 心痛💔 2017. 真的政府看看吧，不止有疫情这一种病 2018. 多转转看看能不能上去 2019. 其实上海很多小区封很久了结果两周多过去温饱和医疗没有解决疫情数字也越来越多一事无成 2020. 抗疫三年后的上海？ 2021. @上海发布⚠️这是在杀人 2022. 求扩散 2023. 转发 2024. 看着揪心啊连上海都这样 2025. 扩一下#浦东疫情# 2026. #浦东疫情#真的让人心酸 2027. @上海发布@人民日报看看人民吧！ 2028. 帮转🙏 2029. 110看看 2030. 真的有毒，疫情这样，上海反应慢，遭罪的都是这些老人！ 2031. @上海发布@浦东发布#浦东疫情#不知能否帮忙推热搜？也请上海政府用点心好么 2032. 帮转！！疫情最不愿看到的事情了 2033. 放心，我去帮你们联系疾控中心 2034. 没应急预案患者不能去医院让社区医护上门服务嘛新冠可备中药重症咋办办要命呢 2035. 赶快安排一下。妈喔好惨 2036. 帮转 2037. 真的难受 2038. 上海没多少确诊的？都是无症状的，怎么就医疗挤兑了？上海在说谎？还是管理不当？ 2039. 新冠没有si多少人，因为gz防控又si了多少人呢？ 2040. 顶顶 2041. 是谣言不？ 2042. 🙏🙏🙏🙏🙏 2043. #浦东疫情#这都是些什么事啊。。转扩#上海疫情# 2044. 限流了居然 2045. @提提是Titi@马思唯喂喂看着都感觉透不过气了 2046. @kikiqo 2047. @向毛爷爷致敬先 2048. @SIIMbA7@needcaaat 2049. //@天津祖安彭于晏:#上海一无症状感染者自述隔离点生活##上海新增50例确诊3450例无症状# 2050. 救命！！！帮扩！！！ 2051. 帮扩！！！ 2052. @瞌睡小羊消失版 2053. #浦东疫情##上海抗议求助##上海新增确诊326例无症状5656例# 2054. @Meiko太惨了 2055. 疫情期间可以保障病人的治疗嘛 2056. //@大家每天都要快乐呀:#浦东疫情##上海疫情#话题为什么不是热一啊 2057. #浦东疫情#@上海发布@浦东发布你不发声我不发声，轮到自己来没人发声 2058. 刚刷到一个长宁区武警总队医院可以做透析，不知真假，需要的可以打电话咨询一下¡评论配图 2059. 顶顶顶求助@上海发布@新华社@人民日报@宣克炅@看看新闻KNEWS@STV新闻坊@警民直通车-上海@澎湃新闻@上观新闻 2060. 上海抗疫求助超话#浦东疫情##上海抗疫求助# 2061. #上海疫情##浦东疫情#@人民日报@上海发布@央视新闻@澎湃新闻救他们！ 2062. 对上海我也是醉了自己管控不合理还以为自己很牛逼说什么自己不能封，别人对它好给它送物资一堆自称本地人的还歧视别人送的东西不好，隔壁安徽的疫情有多少是从上海回来的心里没点数吗，你不封城就管好人别乱跑祸害一堆城市行吗夸自己牛逼的时候一点也不吝啬现在怎么不好好保 2063. 以前是真喜欢上海小时候就喜欢现在真觉得恶心怎么样死都不能新冠死除了新冠是病其他都不是病对吧 2064. 🙏🙏🙏 2065. #上海疫情##浦东疫情#顶顶 2066. @别动-我在发电 2067. #浦东疫情#大家看看 2068. @-胖头鱼胖胖oO那个最后一张武汉的看得我破防了 2069. 作为一个在上海的外地人也不希望看到上海变成这个样子，虽人微言轻也要尽自己的一份力，希望看到人们都给予转发，在微博接力一条生命通道…..🙏🙏#微博客服中心##微博客服#@稀土部队@敬一丹@韩红 2070. #上海疫情##浦东疫情#🙏🙏🙏🙏🙏 2071. #央视网##人民日报##国家卫健委##上海疫情# 2072. 帮忙扩散，希望可以帮助到有需要的人！看来踢皮球的，推卸责任的，不作为的，乱作为的基层机构真的很多啊。可是为什么他们的作风都如此一致，嘴脸一模一样，可以邀功的时候，他们无处不在，背地里，却又冷血自私，毫无操守。@上海发布@九里亭发布没错，九里亭街道，你也是这个样 2073. @人民日报@澎湃新闻#浦东疫情##上海疫情# 2074. 转发求助，救救这些老百姓吧#上海疫情##浦东疫情#@人民网@观察者网@上海发布@环球时报@央视新闻@央视网@南方周末@上海交通广播 2075. //@莲黑屠宰场:#浦东疫情##上海疫情#求助@上海发布@上海卫生健康监督@健康上海12320上面领导只是一句话，下面基层根本不知道如何执行！市政府医院基层这中间多条程序链不明确任何一个小关口不明确如何执行都会让普通百姓卡壳！ 2076. #浦东疫情##上海求助#帮扩📢 2077. 好像当初武汉的情况 2078. 这是上海吗 2079. #浦东疫情##上海疫情#@人民日报 2080. @简直好 2081. #照护3岁以下婴幼儿这样减个税##新冠治疗口服药到浙江了##上海抗疫求助[超话]##上海疫情#@上海发布 2082. 坐标上海……我们这里血透病人，癌症患者，孕产妇都是可以出去的，居委会开证明……慢性病人的药每天也有志愿者去代配的，但这次防疫真让人很无语一丝一丝切的烦透了 2083. 能做的只有这么多了，希望每个人都能健健康康的挺过这一关。帮帮他们！#上海疫情##浦东疫情# 2084. 救救这些人，救救他们，天啊 2085. 我爸现在是肝癌晚期深圳疫情防控我爸现在非常危险还不让超过一人陪护我妈也快累倒了 2086. 帮扩，希望都能得到妥善解决#浦东疫情# 2087. #浦东封控# 2088. #浦东疫情##上海疫情#这还是上海吗？？？这就是所谓的国际大都市？？？我yue了💩 2089. 这时候医生怎么不是医者仁心了？救人不是第一位的吗？怎么还见死不救？ 2090. 刽子手出来谢罪 2091. 亲历上海疫情：重新认识“白菜价”O网页链接 2092. 就这个价晚了还抢不到，你说可笑不可笑，可悲不可悲 2093. 当初马云说房子会白菜价我以为房子会降价闹半天原来是白菜涨价 2094. 高岛屋百货发国难财呢¡评论配图 2095. 现在上海随便一个超市，菜市场价格翻几倍。为什么没有人管，巡查？网络上报道的菜价涨疯了看不见吗？还是选择性看不见。三根莴笋59元。平时5块一斤翻了3倍。¡评论配图 2096. 这些91，那颗白菜31¡评论配图 2097. “烽火连三月，家蔬抵万金”果然段子来源于生活 2098. 丝瓜28一斤，四根丝瓜+一颗娃娃菜，75块 2099. 发国难财的生意也做不久 2100. 80一颗白菜，已经被罚50w 2101. 谁能想到十几年前QQ空间的闹钟偷菜会变成现实 2102. 宝山区，两根莴苣，两棵杭白菜，5斤米，一颗娃娃菜，两个土豆，两个洋葱，三个青椒，107块¡评论配图 2103. 大家仔细看价格¡评论配图 2104. 买了一点小葱和土豆，平常应该是10元的东西今天花了48。古人说得对，无奸不商。 2105. 不光是上海，别的疫情严重的城市也查查吧，发国难财 2106. 反而白云到虹桥的机票价格直逼真白菜价 2107. 来了来了¡评论配图 2108. 关键是这个价格我们也买不到额 2109. 吃雪菜，吃罐头，吃鱼干，吃牛肉干，吃腊肉……这么多选择，高价的蔬菜放着让它烂了罢。特殊情况下可以不吃这些蔬菜，20天之后就好了…… 2110. 现在发国难财的，将来会有报应的 2111. ¡评论配图 2112. 很多都是高价进来的吧（除了那些特别离谱的） 2113. 昨天晚上路过马路边菜摊，老奶奶称了一颗白菜（黄芽菜）店主说46元，老奶奶吓得后退两步连忙说不要了。坐标上海杨浦控江鞍山区域 2114. 4.98元一斤，这个是正常价格吧，我在别的城市，没有封城，豆角14元一斤 2115. 普陀，今晚，一个小菜场，红皮鸡蛋，平常5-7元钱一斤，今天卖17.5元一斤，简直疯了，我想举报他！！！ 2116. 现在抢菜就是飞机票价格了两天花了快两千了 2117. 我都不吃菜¡评论配图 2118. 今天居委提供的保障套餐，50元，这个价格我个人可以接受。坐标上海。¡评论配图 2119. 疫情期间发的菜，还给了一箱奶。西安在疫情封城期间也没有缺过菜吃，当然我说的只是我小区跟周边小区，其他地方没有经历也不能评判，不论是本地人还是租客没有听说缺过菜，当然菜价肯定是要比平时贵些。¡评论配图 2120. 深圳海吉星十万分家庭蔬菜包今天就来了，不要慌¡评论配图 2121. 西蓝花现在到处都快看不到简直是奢侈品，冰箱里放了半颗，舍不得吃¡评论配图 2122. 昨天看了一下小米椒39.9一斤 2123. 放心贵州人民今天已经送过去，二十吨白菜，二十吨包菜，还有葱 2124. 想当年广州封城，35块1斤菜心。。但是肉是便宜的，可能这就是不能冷冻的烦恼了吧 2125. 几个土豆20，一百多的蔬菜包盲盒¡评论配图 2126. 我四十块钱买了三斤土豆第二天找菜贩子昂她一脸你爱买不买的样子坐标杨浦 2127. 32块的不算贵吧，不能只看价格不看重量。78的就贵了很多。我去年有次买白菜6.8一斤，因为一直下雨，平时买两三块，还有一块多的。 2128. 愿在上海的亲人朋友一切安好！ 2129. 发国难财的，请一律重重的罚 2130. 好想给你们寄点菜这边生菜今天2.6一斤 2131. 听说现在飞上海的机票白菜价我不信，过来一看白菜价更高 2132. ，终于罚款了但是50万太少了建议一百万 2133. 二道贩子赚疯了。他们恨不得把蔬菜卖出黄金价格 2134. 这就是发国难财吗 2135. 我们这儿开始65一颗白菜，三线城市，但有关部门发现很快就不许涨价了，白菜不许超过3元一斤。我们这儿应对疫情非常积极，有序 2136. 我今天买了一颗西红柿6块钱 2137. 相比之下广深虽然不是世界经济中心，但抗战疫情是井井有条，胸有成竹，而且没有上海一样的白菜价，大上海加油吧。 2138. 就应该严惩哄抬价格的商家 2139. 一定要好好治理 2140. 物价控制下吧 2141. 发国难财迟早有报应 2142. 这样对比下，油价就非常人性化了 2143. 3根黄瓜36¡评论配图 2144. 这颗娃娃菜花了我13¡评论配图 2145. 莴笋一根50¡评论配图 2146. 关键这个价格都未必能让你抢到 2147. 平时在家里屯点粮食的重要性现在就体现出来了 2148. 昨天花200块钱买的菜都没塞满冰箱，我还觉得4.98的生菜，5.98的番茄🍅贵。是我觉悟不够。 2149. 挖槽我提前一天参加了¡评论配图 2150. 坐标上海，昨晚十二点左右，小区挨家挨户发的一箱物资，终于等到，。¡评论配图 2151. 我这还便宜点，不过也是前两天的菜价¡评论配图 2152. 哪里能买再贵都行 2153. 真就这样，三根黄瓜27，两个小西兰花40，吃不起啊 2154. 一把蒜黄一把蒜苔两颗小白菜101块 2155. 宝鸡市前两天疫情，一把韭菜，一把菠菜，一个包菜，一把蒜薹，两个西红柿，两根胡萝卜，两根黄瓜，两个白萝卜，一把芹菜，外加一个生姜和大蒜，一共30块。 2156. 徐汇，80¡评论配图 2157. 圖片評論¡评论配图 2158. 白菜，天价//@悬壶问茶:上海白菜价！//@松树林姐:上海疫情下一下重新认识的白菜价 2159. 在家弄点豆芽吧这个还能吃 2160. 感觉这个已经够贵的了，一比，不算啥¡评论配图 2161. 图里白菜已经很便宜了，实际上现在是翻倍卖的，倒是把所有地方都罚一下 2162. 买了一把韭黄48，啥条件啊 2163. 疫情严控期间，商家的成本也在增加，肯定会比平时贵，但这贵得有点离谱。 2164. “……家蔬抵万金”段子果然来源于生活 2165. 今天我们这边菜市场也开始抢菜了，价格也涨了很多，说是菜都支援上海了。坐标杭州 2166. 水果的价格才高呢 2167. 女儿说根本抢不到蔬菜 2168. 我们小区的菜店，一根大葱10块，昨天还在和老公笑那条为了追20块钱3根大葱追了两公里的新闻🙃，谁知今天我们小区更离谱。但是目前大润发菜价还行 2169. 今天买的12一斤呵呵哒 2170. 以后的上海应该会热衷于阳台种菜 2171. 小城市的菜O网页链接 2172. 这些菜47O网页链接 2173. 发国难财的人比比皆是。 2174. 花菜58，举报无果O网页链接 2175. “烽火连三月家蔬抵万金”这句也太有才了吧 2176. 不敢相信 2177. 这些菜230你不买后面大把人等着买，去晚了有钱买不到O网页链接 2178. 我是长春的，疫情来了感觉哪都一样，跟城市发达不发达没啥关系，最开始长春就是这样的，开始你嫌菜贵，后期都买不到菜，只能买高价菜 2179. 离谱一个包菜一把豆角46 2180. 普通青椒17一斤 2181. 昨天青菜15一斤，今天葱38一斤 2182. 今天四个土豆一捆青菜30元，两斤肉🥩58元。这是明抢 2183. 以后不能说飞机票白菜价了！#上海有超市白菜10元一斤# 2184. 重新定义《白菜价》 2185. 今年的豆角是真的贵，西安14块8一斤…一根豆角5毛钱… 2186. 220的水果，这么点苹果108，当车厘子吃了¡评论配图 2187. 买了一个土豆10块钱 2188. 一颗大头菜都买不起了 2189. 早点重视疫情，早点控制住，不应该是这种结果 2190. 今天抢到了叮咚，何其的幸运 2191. xuhu徐汇区，4根大葱30块 2192. 白菜像奢侈品一样啊¡评论配图 2193. 进货价？都是菜贩子赚去了，这么高价格农民也收不到 2194. 我买的白菜9元一斤，加两根超大胡萝卜60元 2195. 现在发现叮咚才是平价菜 2196. 别说疫情，就是平时下雨或者天冷，我们这边菜价也会涨啊，广东哦，我去年买过一次菠菜，一斤16，今年春节，一斤春菜10元，所以现在上海菜价贵不是发国难财吧，也是特殊时期，供不应求导致的短暂物价上涨，我觉得很正常 2197. 青菜贵别吃了，吃猪肉，不行在吃点维生素补补 2198. 不止上海吧，其它地方蔬菜也在涨价只是没这么夸张 2199. 蔬菜大省的山东白菜都八块一斤。。。 2200. 趁火打劫吗？ 2201. 我妈今天菜场买的菜椒，20块一斤 2202. 大城市确实很难，人那么多，特别是基本生活物资供应不容易。 2203. 超市价格还可以，菜场价格涨疯了 2204. 就罚这点？就这惩罚谁在乎？无期一个全长记性了。 2205. 这些发国难财的人真的要重罚 2206. 一颗白菜70块，被罚了50w，好划算哦 2207. 今天无锡白菜也是10元一斤 2208. 疫情前7毛一斤白萝卜，早上我妈买的5块5一斤 2209. 四个西红柿两个土豆六个橘子74 2210. 蔬菜价格这样上涨，老百姓确实吃不消！ 2211. 买了六个苹果，六个梨，六个耙耙柑，160没了就跟夸张 2212. 我昨天买的一颗白菜也是30元 2213. 白菜鸡毛菜只要是菜都要十块以上一斤了能买到蔬菜谁还看价格呢（麻木了.. 2214. 蔬菜水果做梦都没想到：自己也有属于自己的高光时刻 2215. 有的地方蔬菜滞销，有的地方卖高价菜 2216. 应该好好管管了，这是什么风气 2217. 蔬菜批发商今天我们这白菜批发价1.5/斤，莲花白1.2/斤。一个星期前更便宜 2218. 很有可能疫情现在遍地开花！外地蔬菜进不来，只能自给自足，量供应不上就得涨价，现在哪哪的菜都涨价 2219. 昨天买了个39.6的大白菜，看到现在77.9我释怀了 2220. 这些商场和超市还能被监管小商贩卖的不比这个便宜甚至贵很多但是完全没有人管🤷🏻‍♀️ 2221. 两颗火龙果65.6 2222. 我在上海，网上不可能买到菜的，蔬菜没有低于二十一斤的，咱不吃蔬菜，吃不起，老干妈就白米饭凑和一个月吧， 2223. 图片评论¡评论配图 2224. “白菜价”这个促销口号，怕是以后没有商家再会用了。 2225. 买了个最小的西瓜快80块了 2226. 一般至少10块一斤 2227. 三颗生菜21，爱买不买，能不能罚一下这些乱涨价的 2228. 永辉杭白菜5块钱一斤。 2229. 这些50坐标上海浦东¡评论配图 2230. 遵义连夜送了50吨蔬菜过去 2231. 还行松江这里¡评论配图 2232. 是不是可以用蔬菜换玲娜贝儿了 2233. 疫情真的是好多人发国难财的利器，良心呢？是不是很多人还巴不得赶紧疫情狠赚一笔呢？不义之财不可取啊 2234. 两斤刀豆50 2235. 现在不是有全国各地的援助嘛？ 2236. 我昨天买了个西瓜和一小盒蓝莓一百多，大冤种这个节骨眼还敢吃西瓜¡评论配图 2237. 发国难财 2238. 罚死他们这些不要脸的！！！ 2239. 就这价还抢不到 2240. #上海疫情#很多街边水果店、超市等等，这两天都忽然改为重点在门口街边摆卖蔬菜。今天很多店的肉昨天就卖完了。今天去某折扣超市，基本上都被抢空。货补不上？ 2241. 虽然但是，图三是卷心菜（恶意提高菜价可耻我先骂了） 2242. @第五晨曦 2243. 昨天买了五颗菜102块 2244. 前天刚买的土豆，1.1元一斤，坐标山东 2245. 涨价原因是什么 2246. 是真的涨了很多，看到那么多地方都有给上海捐物资蔬菜，可是到我们买的时候还是价格飞涨。大一些的超市和网上买菜价格还算正常，可是一些菜市场和小菜店价格简直离谱，更可笑的是这么贵你还可能买不到 2247. 话说春季其实也是菜价比较贵的时候哦，多雨天的蔬菜不好保存，损耗大，价格本来就是贵的，疫情当前贵点还能理解，但是这个白菜一颗几十块有点夸张 2248. 图片评论¡评论配图 2249. 这些挣国难财的无良商家一定要追查到底，一群强盗 2250. 发国难财？ 2251. 马云说的楼房白菜价实现了 2252. //@中新经纬:亲历上海疫情：重新认识“白菜价”O网页链接 2253. 50万发烧了的 2254. #上海疫情下被重新认识的白菜价#如今的白菜价，已不是当初你认识的，现在的白菜价，你“高攀”不起。白菜：感谢疫情提升我的“身价” 2255. 白菜价不是白菜价了 2256. 三根黄瓜三十二元 2257. 凤梨50，一颗西芹20多 2258. 现在应该说：天呐，居然涨成了“白菜价” 2259. 浦东菜价O网页链接 2260. 坐标宁波，今天市场菜肉价格全部上涨，昨天才8块的今天就15了，，明天去超市看看，商超应该不敢涨吧 2261. 不知道上海人会蒸馒头不，作为西安人，疫情期间在家基本吃面条，馒头，菜省着点吃 2262. 我昨天买了两颗小白菜，真的不大，19块钱一斤，花了将近40块 2263. 四个杭白菜+荷兰豆95 2264. 昨天买了8两小米椒，65.8元。今天买基围虾一斤75元也不觉得它贵了 2265. 两根莴菜4个生菜2把菠菜63.9 2266. 帮我妹买到了正常的菜价O网页链接 2267. 昨天买一个菠萝57块钱O网页链接 2268. 七个番茄70多块钱 2269. 我以为我这边10元一斤的西红柿已经很离谱了。。。。。。我当时都想拿他的西红柿砸他 2270. 楼下小店贵的离谱，压根没买 2271. 昨天买了两个梨，居然称完了，要42块 2272. 学校里食堂旁边的水果耙耙柑都卖二十多一盒一盒两个 2273. 江西的80吨援沪蔬菜30号已经出发了¡评论配图 2274. 生菜一颗10块 2275. 那还行我们这一颗40多我好很久没买了 2276. 大部分蔬菜贵是因为本地蔬菜供应不上，得从外地调运，因为疫情全国现在蔬菜价格本来就高，在加上从没有疫情地方，去一趟疫情区，司机回来就得隔离一段时间，这样也就增加了运输成本要不然没人去，还有前段时间乌俄打仗，油价也涨，今年是啥都贵，就钱不值钱了 2277. 我买的卷心菜24黄瓜17.5一斤 2278. 青菜都十五元一斤，还得手快有手慢无 2279. 考虑在家小阳台养点简单的菜 2280. 我还买到了18块一斤的青椒，可敢相信，一个疫情搞得吃不起菜了快 2281. 真的无语，昨天晚上卖菜小贩说刚刚是15.8一斤辣椒，现在涨价16.8块一斤，越晚买还有再涨价 2282. 我家附近的菜场蔬菜是翻了3-4倍的价格，超市倒是涨幅不大但早已售空 2283. 烽火连三月，家蔬抵万金 2284. 现在物价就是高很多。我们三线一颗白菜也3块多一斤 2285. 自从上海这波疫情后，再也不敢说操着卖白粉的心，挣着卖白菜的钱了 2286. 我前几天还没感觉说白菜难求，我在杭州，昨天买青菜没买到，刚才我在盒马买，加了购物车，一会儿结账就没有了，然后去叮咚，也是这样的情况，之前晚上都可以买得到的 2287. 要不咱们囤点种子自己种叭 2288. 西红柿13.5一斤 2289. 昨天买的芜湖椒25一斤 2290. 昨天深圳捐了500吨蔬菜去上海，但朋友圈儿的上海人说了，他们不缺蔬菜，要多少有多少，只是人多哄抢加没物流造成的这些问题 2291. 虽然但是老家菜农的依旧便宜甚至卖不出去 2292. 保留好小票后期清算这帮发国难财的孙子 2293. 才十块钱一斤这算啥长宁区这边某小区门口水果店卖的黄瓜18.8元500克 2294. 不仅贵，还要抢，还不新鲜有烂的。。。 2295. 嗯上海的铁子们等等我，我要过去卖个烤红薯烤茄子看看能不能卖到这白菜价呢 2296. 深圳封城第二天我在app下单的几样蔬菜价格截图，价格没变，后面怕吃不完也不敢多买。¡查看图片 2297. 几天前40一颗的白菜已经吃完了 2298. 我5个苹果，5个橙子，一盒青提，卖我150。。。 2299. 现在机票真的是白菜价，噢，可能比白菜还要便宜 2300. 杭州的菜也涨了很多 2301. 买了十五元一斤的青菜，能买到已经很感恩了 2302. 买了2根丝瓜。3根黄瓜，两个洋葱，4个番茄，70块钱 2303. 西红柿12块一斤，香菇20块一斤 2304. 昨天在家楼下小摊买的:番茄8个，卷心菜2只，土豆6个，西葫芦4个，小油菜一把，两个花菜，花了200多 2305. 就真的贵得离谱，平时我买菜也很少问价格，但是很明显能感觉到平时十几二十元份量的菜，这次花了我一百多 2306. 以后谁还敢说便宜的东西是白菜价了 2307. 买菜哪有选择的权利，有啥买啥，闭眼入 2308. 哪些店在哄抬物价，到时候秋后算账 2309. 我还耿耿于怀，昨天上海郊区菜场买的一斤青菜，一斤茄子，2斤土豆，100块钱 2310. 虽然但是坐标上海隔壁，我们平时西红柿也在8-10块，豆角类基本都是10+，蒜苔10+ 2311. 坐标浦西，菜价正常O网页链接 2312. 我买的8块一斤，相对良心了 2313. 看图吧O网页链接 2314. 一整条蔬菜链，不光光是超市涨价，疫情管控之下，农户，运输，批发商都会涨价 2315. 附近有个小菜场30块一斤的宁夏小青菜早上还卖25，下午就变30了 2316. 138这些菜O网页链接 2317. 这点菜，115，坐标宝山O网页链接 2318. 菜场都不止卖10块一斤，前天都有人卖15/斤了，就这个价格了十点不到蔬菜摊都抢空了 2319. 我这没一个荤菜，都是超市平时便宜的菜，现在花了我一百O网页链接 2320. 图片评论O网页链接 2321. 杭州也涨价了 2322. 8块钱买了根葱 2323. 不仅是白菜呢O网页链接 2324. 我买了七个苹果36还选的最便宜的 2325. 10块一斤真便宜，我昨天买的18上海青17一斤，前天买更贵 2326. 白菜18一颗西蓝花十块一颗 2327. 客观说两句，从18号被封抢到现在，菜价还好，主要是运力难抢。电商平台和超市价格跟平时差不多，菜场和小区门口的摊贩涨幅比平时高了25%。至于高岛屋那个77的白菜被罚了50万，其实高岛屋这种地方它也不是今天才贵，一直就这么贵，联华超市土豆107一公斤，小数点错了一位，卖了一盒就贴召回通知了。 2328. 两根黄瓜17,两颗莴苣30，这还是前几天，这两天更离谱 2329. 今天买了两斤西红柿38 2330. 上海都这样，其他小城市要是沦陷了更不敢想象。大家一起努力努力，争取过正常的生活！🙏🙏加油加油。 2331. 无良商家，我们着土豆几毛钱一斤还是新土豆， 2332. 上海周边城市，白菜也已经7元一斤了 2333. 我们小区门口可以买，商贩在外面，我们在里面…拿到手的菜好多都是烂的软的O网页链接 2334. 昨晚刚拍的，合肥菜价，突然感觉很幸福O网页链接 2335. 还好我家有菜园自己种的有各种蔬菜在超市里逛了一圈想买新鲜辣椒炒的以前一块多现在八九块一斤算了还是回家吃酸辣椒吧现在卖的菜是真贵 2336. 80O网页链接 2337. 求求管管吧，都是一群发国难财的O网页链接 2338. 买了4个土豆，4个黄瓜50块钱真是贵的离谱 2339. 疫情期间恶意哄抬物价可以构成非法经营罪哦 2340. 我们前几天白菜一颗110～然后被罚了好多钱……… 2341. 两斤馄饨80，也不知道是不是包了龙肉 2342. 同事买的两根黄瓜85 2343. 10元一斤在我这都垫底了昨天走了三个摊位才买到10块一斤的，还有卖20一斤的 2344. 78不是白菜是卷心菜 2345. 海南白菜平时都是八九块一斤啊 2346. 可是现在都是这个价啊…… 2347. 这么敢吗。我刚刚从美团优选上买完菜。80元买一堆 2348. 上海现在不管市区郊区蔬菜价格已经普遍10元以上了，都赶上肉的价格了。小区封闭的这半个月里伙食费比平时一个月还高。因为都已经是“普遍”现象了，所以大家都只能默认“算了，疫情没办法”“你不要有别人要”“不怕你不要，就怕买不到” 2349. 我爷爷自己种的莴笋上周卖2元1斤还卖不掉。现在小区封闭刚好留着自己吃 2350. 我买了一颗白菜51 2351. 看完评论我感觉前两天买的两根黄瓜+两颗小娃娃菜36块好像挺划算的 2352. 昨天去一个生鲜店土豆六块一斤，白菜八块就离谱，原来土豆一块钱一斤，赤裸裸的发国难财，菜价确实贵，买不起菜确实真实 2353. 我买了7个粑粑柑，68块钱，真的是天价 2354. 有东西就不错了，，还能看价格买东西呢？¡评论配图 2355. 昨天买了这些菜130坐标长宁¡评论配图 2356. 重新定义“白菜价”//@四叶草NNZZ:“烽火连三月，家蔬抵万金”果然段子来源于生活 2357. 就说，我感觉我没有白菜值钱 2358. 小白菜12元一斤 2359. 我买了4个番茄24块钱就这还是排队好久买到的 2360. 转发微博 2361. 看来昨天我买三颗娃娃菜25不算贵 2362. 这是在干什么？发国难财？ 2363. 不想错过草莓季 2364. 我们是小城市……O网页链接 2365. 这不是国难财，这是经济学规律。 2366. 两串葡萄180，还坏了一半O网页链接 2367. 19一斤的鸡蛋O网页链接 2368. 盒马146O网页链接 2369. 就这些160块，平时也就三四十O网页链接 2370. 呃，我们小区刚刚发了免费的蔬菜，肉，和大米，我去超市买菜价位也不高坐标上海浦西 2371. @Jessica小盆友 2372. 买菜配货真不是开玩笑的，就这样还抢不到呢，土豆黄瓜的均价10元一斤！这是什么神仙物件！O网页链接 2373. 不止高岛屋一家……昨天在百联联华下单，生鸡蛋一个两三块……无奈换了叮咚，结果是全部涨价……资本主义还是厉害，在金融中心的上海就是吃得开。//@科技刚子:高岛屋百货发国难财呢O网页链接 2374. 烽火连三月，家蔬抵万金 2375. 一根白菜🥬➕6个小土豆🥔总价：63.6元已经买到家并且供起来了 2376. 两根黄瓜500g两个西红柿500g,三个土豆500g豆芽500g一颗花菜900g一颗白菜1000g8个鸡蛋150的组合菜贵就算了，还很难抢到菜，质量也没保障没售后两个西红柿烂了一个鸡蛋烂了一般，花菜压烂了一半白菜也压烂，整个觉得买的很费但是舍不得扔。睡醒就发现被封没一点准备 2377. 130O网页链接 2378. 发这种国难财，良心不痛吗 2379. 冰箱里还有棵白菜薅的还剩菜心赶快转移到花盆供起来 2380. 确实贵，长春的我们还在各个平台抢菜虽然一个菜叶子也没见到 2381. 10元一斤不贵啊，我买的都15一斤了 2382. 被重新认识的又何止是白菜价 2383. 虽然但是，图三是包菜 2384. 美团买菜还是比较人性的，没怎么涨价，就是比较难抢O网页链接 2385. 一说封控，抢购物资，物价暴涨，哪都一样 2386. 我的卷心菜都才7-8块钱一颗不能以偏概全 2387. 全国都在给上海送菜 2388. 机票价都比白菜价便宜，以后不能说白菜价了 2389. 昨天绿叶菜20一斤了 2390. 现在浦东小区群里发出来的蔬菜团购，差不多都是10元一斤 2391. 我有10斤土豆，突然觉得好富有 2392. 图片评论¡评论配图 2393. 昨天我们小区发了蔬菜盲盒，里边有两颗大娃娃菜，四个土豆，五个番茄，还有一斤芹菜 2394. 大润发永远的神！昨天去买了一堆，正常价格¡评论配图 2395. @文化有限y真的白菜价哈哈哈 2396. @hh-路人甲 2397. 图片评论¡评论配图 2398. 深有同感套餐价119！！！青莱3斤莴笋2.8斤洋葱2.7斤芹菜2.4斤茄子1.6斤西兰花1.5斤黄瓜1.6斤包菜3斤共计18.6斤¡查看图片 2399. 只买了两天的量蔬菜只买了两种，就这还是抢购来的。¡评论配图 2400. 坐标普陀。帮我朋友买的。闪送过去。真心贵¡评论配图 2401. 今天在园区抢到2颗白菜70，确实体验了一把抢着买贵的 2402. 这些400多¡评论配图 2403. 哦O网页链接 2404. 控制一下啊。去年年底在西安买菜都没这么离谱的，两个人基本上一百多出头就可以买好大袋子菜 2405. 发国难财的罚他倾家荡产 2406. 买菜问价都没人搭理你！爱买不买…… 2407. 看着看着我就笑了，浦东的¡评论配图 2408. 我们这四线小城市一棵白菜都要20左右上海这个价格还不是正常 2409. 深圳居家7天的时候蔬菜价格的确还算是平稳的，据说是在决定封城前提前做好了蔬菜供应的准备 2410. 发国难财的，拉出来游街，纳入企业个人征信，太可恶了，这么严重的疫情，大家都在积极配合，一线的人那么辛苦，你们居然还这发横财 2411. 应该是疫情连三月，家蔬抵万金，现在的菜真的好贵 2412. 我们这三线小城市一棵白菜也二十多了 2413. 首先，这波疫情，蔬菜涨价应该是有的，但也并不是全部像网上吐槽的那样夸张，不要只看总价，看看重量啊。图2有6斤多重的白菜，32元，5块多一斤。事实上，过完年到这波疫情之前，蔬菜已经大面积涨价了啊，你们在这波疫情之前没发现吗？ 2414. 现在蔬菜供应不求，进价高了，自然涨了，能吃上就不错了，嫌贵可以选择不买或少买 2415. 政府还给发菜，米，面，油呢 2416. 现在是高价都买不到菜了 2417. 这课白菜32元¡评论配图 2418. 还是想不懂，上海现在七万多例了，才累计七个人死亡，死亡率1/10000，我们却动态清零，到底在怕什么 2419. 昨天我们隔壁楼上门做核酸，做完也是大家冲着楼下大白喊，谢谢大白！正常人都会这么做，拿了菜在网上嫌东嫌西的是坏，转来转去引流量的大概既坏且坏。他们不是义愤填膺，而是知道这样会让更多人一起骂上海。 2420. 有些人吧，人家不表达感谢，你们说看不起人；人家表达感谢了，你们又说是形式主义。你们到底想咋样嘛？ 2421. 什么叫做负面新闻，新闻没有正面负面，只有事实 2422. 很客观。从来没地域攻击过全上海人不过到处投毒的真的很欠 2423. 上海的城市名片已经被撕碎，想要重新粘回去。。需要时间 2424. 但是上海人排外是真的排啊 2425. 打人视频呢？ 2426. 这个不是排练好的吗到现在了还大搞特搞形式主义 2427. 这次疫情对上海人本地人好感全无，太没有人情味了 2428. 今天还看到有一个上海的暗讽广东只去了300人，唉！又要支援香港，又要支援吉林，还要顾及被上海波及的那几个城市，真的服了，做好人真难，不知恩情者比比皆是，愿上海早日战胜疫情，活捉吴凡 2429. 上海做为一个国际大都市，这次的疫情怎么乱成这样？还有人提出与病毒共存，不知道中央政策？而且昨天方舱医院附近莫名其妙发生火灾，越来越他妈离谱！难不成有人诚心搞事？你他妈封城能咋地，上个月从上海输入我们这小县城一例，回来不隔离不报备，就为了看小三传染了一大圈，市政府还下令援建方舱 2430. 微博上有多少上海人，能有个两三百万就很不错了，你你这ip归属地是上海就是上海人了？上海有三千万人的话，只有一千两百万人有上海户口，试问，有哪个城市本地人口比例比上海低的 2431. 这才是真实的上海 2432. 能出现那么多负面消息，真的会是无中生有编出来的吗？其他有疫情城市负面消息大多趋同，为啥上海的负面消息这么特殊 2433. 我一开始看到骂上海的新闻也很生气，甚至很多人装都不装，就是说的“我早看上海不顺眼了，没疫情我也骂”，后来我想通了，上海人的生活不会因为他们的辱骂而变差，这些人也不会因为骂了上海生活质量会变高，如果骂上海能让他们得到些许成就感，那就让他们骂吧，反正现实中和这些人没交集 2434. 感觉SH有人故意掩盖人民的真实声音，再故意放大负面的、抵抗的声音，想以“民意”挟持政策。 2435. 章子，这里面水太深，你把握不住，听叔一句劝，退网吧 2436. 感觉这个时候好多行走的50w在拼命就着上海的风口制造舆论的，希望别被利用了，归根结底国家是我们的，我们应该相信国家🙏 2437. 某地不是带着特有的傲慢，吹着最响的牛皮，爆炸之后，一地鸡毛，还死鸭子嘴硬 2438. 真的少吗？上海共存派可不少 2439. 转发微博 2440. 新闻没有负面正面，只有事实，把事实报导出来没有错。 2441. 我朋友在上海都他妈快断粮了，力气都没有了，还跟你谢谢？谢谢医护和志愿者，不谢谢你们所谓的“防疫政策” 2442. 上海疫情本来可以像深圳一样避免结果这样了……被骂是应该的，毕竟是一线城市啊 2443. 为什么不先想想为什么这次防疫一面倒被骂？ 2444. 怎么不跳一段儿了 2445. 你发国外和港澳台的负面新闻的时候，可不是这么说的……双标很正常，但是你本来就双标，又来反对双标，真的令人无语 2446. #上海高风险地区查询 2447. 江苏真给力了，一下派出一万多人 2448. 积极解决出现的问题才最重要 2449. 上海现在就像一个西装笔挺的爷叔拉了一裤裆，他不想着去赶紧把稀洗了把裤子换了，反而捏着其他兄弟姐妹的嘴不让说臭，还说这是咖啡印红酒香，好了现在把自己搞瘫痪了，还要全国各地给他擦屁股换裤子，还一点都不反思，真的是 2450. 这就要点名两个博主：ID名叫是个小姬零鬼，罗森店员和泉子了 2451. 章老师不容易啊 2452. 共度难关 2453. 愤怒的新闻是少，毕竟微博审核员不是吃白饭的 2454. 上海加油 2455. 整栋楼的老百姓说谢谢，不至于要说人家是作秀。什么地方都有好人坏人，好人居多。 2456. 上海人真的是会会说 2457. 谢谢方块头传递正能量，谢谢来支援上海的同胞们 2458. 网上的视频，那些活生生的人，那些骂人的话，都是假的？都在演戏？疫情结束以后外地人会和本地人一样吗？ 2459. 家里长辈看了很多负面新闻，就开始各种担心，每天发很多信息给我，我还是坚定的安慰他们：我们会扛过去的！就算所有物质最后都会归于虚无，但至少不是这次。人类是渺小又伟大的存在啊。 2460. 每一个人都是中华好儿女，每一寸土地都是九百六十万平方之一 2461. 能全国骂肯定有原因，难不成全国其他地方一起商量来的？ 2462. 负面新闻就不是新闻了？重点不是正面负面，重点是这些正面负面的新闻都是事实！别光能承受住赞美，却不认可自己的不足！ 2463. 上海人上人装 2464. 永远不要离什么都不做的喷子，把我们每个人该做的事做好，中国加油啊 2465. 我只想说既然知道防疫人员辛苦大家就自觉点配合点管理到位点不然我们就白关了这么久也对不起人家千里迢迢来帮我们收拾烂摊子 2466. 毫无表演痕迹 2467. 负面的事件还少吗？ 2468. 说这句话的，是在沪华人，本地人不会的。 2469. 你这评论区真是精彩难怪上海人还有一堆人想共存了 2470. 还真是，巨量转载评论，而且一件小事能迅速上升到国家政体上面 2471. 英勇抗疫的上海人民万岁，躺平装死的文邬双废遗臭万年 2472. 地图炮陕西人河南人上海人，说小了那也是一个个活生生的中国人，可能昨天还和你在网上一起哈哈哈，或者跟你打了一局游戏，有问题就说问题，不要地图炮 2473. 很奇怪的是今年疫情那么多无症状已治愈的没有看见有人发声呢？无症状感染到底是什么样，有没有后遗症患者？是后遗症不愿发声还是其他原因？ 2474. 支持 2475. 喜欢看到这样的画面 2476. 一个到处控评的地方，哪里有什么负面新闻啊 2477. 上海这一系列的国际化操作，不管放在中国任何一个省市绝对会比现在骂得更凶！ 2478. 方脑壳有颗温情的心。 2479. 不能因为部分行为就对某些地方地图炮，我始终相信大部分民众都是善良知礼节的，话说上海这方面宣传能不能支愣起来啊 2480. 都2022了，上海疫情还能媲美到20年武汉的水平，就离谱 2481. 传递正能量 2482. 加个俄罗斯乌克兰再看看IP 2483. 这7列应该是20年年初的吧 2484. 好事不出村，坏事传千里，但坏事也是工作中的不足，也应该予以重视改进 2485. 上海人排外是一直以来的事情，这个洗不掉的 2486. 我只是想到好几万阳性了，没有高风险地区吗，我只是不懂而已，谁能解释一下呢，不得不好让人捂嘴吗？ 2487. 心 2488. 顾村要物资！！！！ 2489. 弘扬正能量，邪不胜正 2490. 管控失利不是本地外地互掐能解决的。大家理智点别互掐。管理层面造成的后果都是基层医护人员基层志愿者和基层人民承受。如果没有管控失利，就不会造成大面积风控，没有大面积封控就不会缺少生活物资，不缺少物资就不会导致捐赠区别对待。我们要的是解决问题：国家已经正视问题并在解决问题了，加油 2491. 七例死亡病例是新冠患者还是因为别的疾病无法就医造成的 2492. 正面新闻真没见多少，只记得最开始把咖啡当好事 2493. 您说的没错，对于志愿者我们心里万分感激，不敢有任何语言亵渎。很多人都是本着，如果帮不上什么忙至少不添乱的原则做人的。民怨如果小，不至于如此吧。一开始个例少以为是传言但事情在身边发生了，还是个例吗。当然这代表不了一个城市，但请正视直面问题。不要偷换概念，把问题引到志愿者身上 2494. 事实只有真假//@直到遇见最好的你:什么叫做负面新闻，新闻没有正面负面，只有事实 2495. 怼的好！//@不是东西的南北2015:是因为有清零政策，所以我们死亡率低，对比你看看美国英国韩国香港的。你们倒好，拿我们政策的结果来攻击我们的政策。//@理性战胜盲目:还是想不懂，上海现在七万多例了，才累计七个人死亡，死亡率1/10000，我们却动态清零，到底在怕什么 2496. 本来新闻基于事实，应该反应社会全貌，但问题是现在正规军的“新闻”全是宣传，想看全面的新闻，必须要通过网络乱军补充。这时候有很多人怪网络信息真假难辨，但有没有想过，人们为什么如此需要网络信息？//@直到遇见最好的你:什么叫做负面新闻，新闻没有正面负面，只有事实 2497. 2020:时代的沙子落到每一个人身上都是沉重的负担，那些不是数字，而是活生生的生命2022:不就死几个人吗？这都接受不了呐 2498. 一切都会好起来的，万众一心，相信国家🇨🇳 2499. 负面是为了引起重视不是为了骂而骂是人民对公信力的监督 2500. “正因为少所以你觉得不可思议，否则你早麻木了”，话虽然是正确的，但用到这里合适么？真等到面对负面新闻麻木的那一天，社会成什么样了？ 2501. 别吹了赶紧治吧 2502. 朋友圈里都在发国家队来了，上海站直了别趴下！这其实就是普通老百姓的心声！没人愿意共存 2503. 说得对，谢谢你。看了平时几个爱国大V的博，这个时候还在挑拨地域，吃上海流量，有考虑过处在疫区的人的情绪吗，有考虑过全国来支援的人的情绪吗，这些人平时表现的爱国，会不知道越是这个时候越需要团结？今天是上海，明天也会是别的地方，它们永远有吃不完的流量… 2504. @地域黑bot来看 2505. 否定嘛？（这是打错字还是我理解错了） 2506. 希望今年是疫情的最后一年🙏 2507. 我小区的群里大家也是满满的感激确实有人不怀好意在分化，但我相信大家能分辨。上海加油！ 2508. 吉林市市长被免职，而上海至今无一人对疫情防控不力负责，这公平吗？不能因为极端的少部分的地图炮否定所有人，这对看不惯上海防疫现状的普通人不公平，而这些人才是大多数。 2509. 吉林的志愿者不是志愿者吗我看那些人也没少骂过一句论地域黑对象被攻击黑吉辽才是首当其冲 2510. 我们对待优秀的人事一但成了惯性常态以致迷信麻木如果失去了发现问题的能力，失去了批评的勇气不敢再拿起批评的武器那社会就要倒退，当然批评是需要真诚的假批评之手笔实恶意所为这跟批评没关系这跟恶劣的品性有关系，所以是有严格区别与划分的，真正的批评和表扬是同性质的，这些都是推动社会的力量 2511. 疫情后会有一波离沪潮吗？ 2512. 如果我没记错，吉林陕西都被免职了不少哦sh动得了吗？ 2513. 对摆烂的政府，已经词穷了¡评论配图 2514. 哪里都有老鼠屎，上海盘大，老鼠屎更多，历史又造就了那么一些本地人的优越调调，这不是一天两天了，不是这次疫情这种调调也一直在。 2515. 所有的成见，先放下；所有的争论，先暂停，先把上海救出来，先让上海活起来。//@胜利主义章北海:有些人吧，人家不表达感谢，你们说看不起人；人家表达感谢了，你们又说是形式主义。你们到底想咋样嘛？ 2516. 骂上海不是骂那些大白，那些基础，骂谁你们懂得 2517. 上海加油，天佑上海。在这种危急时刻，我们需要的不是仇恨对立和互相埋怨，而是爱与团结一起努力撑过难关。 2518. 顾村都要没物资了 2519. 上海真的太自大了，去年底期间爆发疫情，很多人反映上海进地铁啥的根本不看健康码。然后一群人还有政府在那吹牛逼，搞得他们管控多厉害，技术多牛逼，说不用看，因为红码黄码都被管控隔离了……然后奶茶店有感染，就封一个奶茶店，说是最小风险区。 2520. 不能因为大多数人觉得这日子还过得去，就否定了少部分人的水生火热 2521. 上海加油我爱上海 2522. 除非己莫为 2523. 你怎么看这样的求助？或者说“负面新闻”？¡评论配图 2524. 少但是存在，存在必有争议，存在就是事实，敲门被骂的志愿者，被报道怎么了？这部分人，就和大家不一样 2525. 一群疲惫不堪的医护把一堆濒临死亡的病人拦在医院外，守着病房生龙活虎的无症状感染者刷手机。 2526. 别喊了注意飞沫传染，拿出行动就行了… 2527. 新闻只有真假，没有正负 2528. 上海现在负面新闻是少数？你来实地看看 2529. 虚假的形式主义🤢 2530. 说的真好 2531. 身为小区业主道谢纯属自发行为，并且也是真心感谢人家从外地来不容易待客之道 2532. 加油啊上海 2533. 哈哈哈，一万三，大家都要夸上海！做得好才一万三呢 2534. 上海是真废了 2535. 别说负面了，上海这个地方我就说一个事实：排外。自负引起的排外，排外引起的你说的那些负面新闻 2536. 新闻媒体讲究客观事实，实事求是，暴露出来的问题是为了解决问题，如果一直宣扬全盘正面，不觉得很可怕吗 2537. 希望本地外地一视同仁吧！🙏🙏🙏 2538. 理解包容！ 2539. 这次上海可真是出名了 2540. 非常在理上海很糟糕但大部分人都没有网上说的那么糟糕 2541. 有人说“求求你不要再发了，因为国内发这些东西，现在上海已经出现反华情绪了。我们留在上海还有好多中国人，为了同胞的安危，求你们别再发攻击上海的内容了！”这是啥意思，反华？ 2542. 这是松江九亭的九亭家园吗 2543. 营销号，你是个P 2544. 能写出这么些东西。我真不知道你良心何在？ 2545. 现在上海的感染病人，往各地送，浙江的几个大城市，每个城市都接受了上万上海人来隔离，地方政府没有发表任何声明，是什么意思？ 2546. 新闻：新近发生的事实 2547. 垃圾垃圾垃圾说的高大上的城市防疫工作做的烂透了还排外几个芍水婆娘把成都整的遍体开花或者说不只成都事情发生了这么久也没见咋个处理这些上海无作为的官员喃打着大家都一样其实不一样吧只有上海户籍才一样哇？ 2548. 在上海的四川人河北人山西人辽宁人吉林人黑龙江人江苏人浙江人安徽人福建人江西人山东人、河南人湖北人湖南人广东人海南人贵州人云南人陕西人甘肃人青海人台湾人；以及内蒙人广西人西藏人宁夏人新疆人；最后还有北京人、天津人和重庆人，加油！你们定将战胜疫情！加油！ 2549. 我现在只后悔当年没考上tz内，不然隔离也可以领取工资，我们现在老百姓要生意没生意要钱没钱，隔离一天内心慌得一逼，对未来好迷茫，各种信用卡逾期，房贷车贷想都不敢想，还要生孩子，呵呵呵！ 2550. 基层累死累活，上层粉饰太平，老百姓面临食物匮乏的风险。如果这些都算是极端的、少部分的负面新闻的话，那索性就变成1984得了。 2551. 只允许你们骂别的城市，就不允许别人说事实，你们上海得有多么好贵， 2552. 我爸妈确诊且有症状，在家待了五天了没人管没人问，没有物资没有药物，到底什么时候给带走治疗啊？坐标浦东新区北蔡镇 2553. 大家能不能理性的评论和发表文章。这明明就是事实存在的东西。根本就不是非黑即白的事情。 2554. 上海加油！！ 2555. 新闻还能有正负，，，，你这也好意思发出来，，， 2556. 怎么说呢四川山东都被连累了你不让人家出出气你们受了帮助说声谢谢别人被连累了骂几句怎么啦山东大妈救助，上海母婴分离常人看了说句不可思议也是常态吧 2557. 封了就好？难不成以后一直封闭？饿死你的时候你就知道了 2558. 做错事了还不给骂，这和国足有什么区别？ 2559. 我就想问一下，支援上海的菜是供给所有隔离的人嘛，除了上海本地人，在上海打工的外地人也可以有免费的菜，吃吗？ 2560. 他们能这么叫嚣一个是已经损害自己的利益了，二是仗着自己身边人多群起而攻之，三是不分青红皂白是风就是雨。体现出资本主义精神，和没有独立思考能力。 2561. 学英国免疫？美狗最多的城市 2562. 国内报喜不报忧，国外报忧不报喜？ 2563. ？？？差不多德勒允许你发正面的不允许别人发负面的，允许你赞美不允许别人批评，好好在家呆着配合防疫就完事了，越解释越掩饰难不成别人视频是假的就你是真的是吧 2564. 新闻还分正面负面？就问你真实不真实 2565. 整这些没用的，你不要乱跑啊，祸害其他地方 2566. 有没有一种可能说谢谢的是外地人呢？ 2567. 章北海大约是说不出这么没水准的话 2568. 真是东方小孟买亚洲小巴黎每天新增一万多有脸在这说什么正面新闻西安疫情的时候你们不还是地图炮喷的贼欢双标成啥样子了真的把西方媒体的精髓都学会了真牛掰不是网络上的话语权掌握在你们手里你们就可以左右真相 2569. 江苏大白我家邻居也去上海做“江苏大白”了，她们好辛苦 2570. 对，大家不要给境外势力递刀子呀！ 2571. 会好起来的，这种时候更要怀揣着信心和希望 2572. 也看看#南汇方舱吧#也转发扩散一下吧好的不好的都应该看到这才是公正的自媒体🙋 2573. 这才是大部分的上海啊 2574. 现在很多人都有了HWB的气质 2575. 这也是某些人某些平台吸引流量的套路，更甚之，就是挑唆制造矛盾 2576. 倒霉的永远是普通百姓，希望你们能挺过去！！！ 2577. 这样一看，青岛防控速度不错啊 2578. 元月份全国骂西安，我觉得除了说“乡下偏远地区西北佬”之外，就事论事的评论我作为一个西安人还都会虚心接受的，而且我们也收到了全国的支援，整个疫情期间家里没缺过啥。看到今天的上海深知其中不易，希望真心祝福上海早日恢复 2579. 全國各地的大白和為支援上海付出辛勤勞動和表達祝福和關心的人們，謝謝你們！❤️ 2580. 安徽人在上海受到歧视的时候没见你出来沪犊子 2581. 乌克兰 2582. 上海伤害了全国人民，疫情疯狂外溢，现在搞得我跨市区都不行了，过年到现在都没回过家。上海=垃圾 2583. 我看到的实际情况是，小区物业特意通知每一户让他们配合演出， 2584. 不要在乎那些说怪话的人！当初西安疫情的时候说怪话的人多了。有很多本地人因为后勤一时没跟上，发发牢骚也要注意表达方式。因为在网上任何反面的话都会被有意放大，其实我们中国的老百姓虽然嘴上发牢骚，行动上是很遵守领导的。所以请不要在网上口无遮拦，被别有用心的人恶意引导！ 2585. 本文意思因为少就不必较真了，不用报道了。这种现象本来就是少，也说明存在这种心理 2586. 上海能给公寓和群租房发食物吗 2587. 中国人不骂中国人 2588. 我本来对上海人没啥想法，但是看到这个上海人疫情期间来我们大广西到处耍无视疫情管控还开地图炮，我想这就是你们疫情为啥这么严重的原因了吧@幻影丶奈 2589. 呵呵那个是真的负面? 2590. 长宁区玉屏南路203弄居委通知二楼有两户确诊，现在都过去几天了也没人拉走，整栋楼卫生间都是在外面的，里面很多老人，出去洗手间风险很大，没人来消毒，核酸也没人来做，现在都不知道有多少人感染了，一直这样恶性循环 2591. 上海不考虑上门先给长者把疫苗打了吗 2592. 还是很动容的 2593. 毕竟不懂这个道理的红卫兵太多 2594. 新闻只有真假，没有好坏 2595. 每个地方都有好的事情和坏的事情，好的事情大家就积极宣传，让大家更加往好的来，出了不好的事情也不能一味的找理由，而是积极的将事情处理好，避免这样事情再次发生，这样才是政府个人民应该积极去做的，暴露问题解决问题才是最棒的，坚决不能隐瞒问题！ 2596. 就是能不能把物质安排到位啊我男朋友就是外地去上海工作的现在被封在小区里面一天就吃一包泡面目前已经断粮政府能不能给安排啊 2597. 就不明白物资还分本地人外地人吗？听说外地人来做核酸打扮的那么漂亮展现自己的高贵吗？我什么也不懂，但是物资还要区分本地人外地人就是不行，送来物资也瞧不上，哪个省活该给上海送物资啊，给吉林哪里随便一个地方送去都比送上海强 2598. 最新的数据上海已经7万3千例新冠感染，死亡人数为零，这就是现实情况，病毒已经发生了很大的变化，目前没有因为新冠肺炎的死亡但是因为封城造成的医疗资源紧张，很多其他危急重症的疾病引发的死亡反而出现了，是时候考虑优化防疫的政策了 2599. 日增一万三，还不封城，非要害人，上海牛逼到要全国给他擦屁股。 2600. 下班了？别走啊，还有二百多小朋友等着跳舞呢，一个个来，预备~听我说谢谢你 2601. 呵呵，继续动态清零吧，把仅有的一点家底也清了，就再也没有可操心的了，0000🈳️🈳️🈳️🈳️，四大皆空，好 2602. 到底是不是少数不是你说了算的，至少外溢省份这方面算是大多数了，全国34个省级行政区，被上海外溢20多个 2603. 我只听过分国籍，这次第一回听到地区籍！我只听过救死扶伤，这次第一回听到护士无人救治！我只听过一起攻坚克难的，这次第一回听说见外地支援的采样人要去奢侈品走秀的！反正我看见的新闻报道真是让我大开眼界！ 2604. 上海现在有多少小区阳性没转走的，是留着等社区交叉感染吗？这是共存派的阴谋的吗？ 2605. 上海加油！一个这么多人口的城市，肯定异常艰辛。不管过程中有多少不合理事情发生，都应该中国人帮中国人，共渡难关！等疫情过后再合法追责。 2606. 对于一个见多了上海金银卡的人来讲这个世界终于能看清上海到底是什么样子了…… 2607. 一句负面新闻，人家发的人命关天，不是你被关，不是你没菜吃饭，你正义的化身在这说这些，上海政府从上到下管理的混乱不堪，不止全国人民要骂上海人自己也在骂，一个全国经济大都市，爆发疫情期间，暴露多少管理问题和漏洞，不认真改正，还阳奉阴违的糊弄上面，让在上海的老百姓水深火热。 2608. 这个怎么说呢，还是要看人的，每个地方都有好人坏人，但他们代表不了所有人 2609. 额，别喷我。。。我在上海也工作过几年。有好的上海朋友，也遇到过部分背地里一口一口外地人的上海人。不得不说，这个确实是事实。而且一直以来都有的部分情况。这次疫情，有个朋友那还不分给外地人。要物资优先本地人，这也是事实。后来报警了才消停。有些上海人确实脑袋有点意思。不是说全部 2610. 上海曾经是国内防控最好的城市，现在发展这么快可能有不为人知的原因，国家在努力尽快清零，作为普通百姓应该配合国家从我做起，可悲的是如今很多人却落井下石的不断嘲讽，国人的悲哀！ 2611. 不要被敌对势力的负面宣传带偏了，要感恩知足 2612. 一帮原始社会的骂现代人 2613. 5号了，在家快待了一个月了，没吃的了今天下楼去居委会讨饭。告诉我在等消息.也不知道能等到啥时候。 2614. 为什么上海还没高风险地区？搞不懂 2615. 个人经历：不同区和街道存在差异，但从通知浦东封城以来共收到两次政府大礼包（各式蔬菜、咸肉和一桶油）加上疫情伊始的储备以及小区居民自发组织的团购，物资肯定不如过去丰富，但可以维持健康生活，小区居民也自发给物业捐款和物资，同样真心感谢所有医护、志愿者，这是困境中必须保有的善念和同理心 2616. 请问小区的阳性什么时候转走？5天了 2617. 上海昌硕电子厂，有确诊病例，还一直开工是怎么想的 2618. #上海新增本土确诊268例无症状13086例#仿佛看到2年前的武汉 2619. 哪个地方都有歪瓜裂枣，我们从不否认为疫情付出的人。上海人排外，也许是个别现象。但每每网上有说上海人排外高傲的言论，很多本地人就会站出来一边说我们没有排外，一边又嘲讽你个外地老没见过世面，活生生一副外地人都该死的嘴脸。如果想反驳，就做出来。不要一边为自己辩护，一边搬起石头砸自己的脚 2620. 都喜欢摆拍，啥是真实？ 2621. 我靠我他妈真的无语了天天看微博一堆说上海人装13，作秀恶心，没有人情味，刷个视频也一堆骂的，我在家封了那么多天每天都要上网课写作业准备考试，一边关注疫情情况，在严重的情况之下看见的却是一堆人的指责与阴阳怪气，心都寒了。 2622. 作为外地人来看，你们上海疫情管控这次就是很烂。倒不是上海人的错，是你们的政府部门，领导的错！因为，我们小破成都，居然也能受到上海疫情的影响。想让我不去关注上海的负面新闻？不好意思，你们上海的疫情在我看来，只有负面。你应该庆幸外省乡下都骂你们的疫情，不然你们政府还得沉迷于自我满足中 2623. 不排除有些负面是坏人所为但没有负面一片祥和水深火热的人民怎么办 2624. 好事不出门，丑事传千里。 2625. 今天石家庄新增一例3.30上海来的大货车司机先不说司机是哪里人就想问问为啥那么严重了上海还不出台相关政策尽量不外溢影响其他城市呢 2626. 新闻有真假，各持意见也没问题，但是上海疫情外溢到全国好几个省份，上海政府的不管控也都是事实啊!这个普通市民没有错，很多人都在积极抗疫，但是也请上海人不要到处跑了，给本来要解封的省份带去麻烦! 2627. 其实已经不是上海一个城市的问题了，回顾整个中国历史，有多少次这样规模的疫情，为啥我们和米国关系不好了，疫情接二连三的发生，且卫健委采取的方式没有很好防控反而疫情持续不断。值得深思 2628. 今天我被派去支援帮忙医生复检小阳楼，居民都很客气说谢谢辛苦了，心里很感动 2629. 你们为啥不唱歌感谢志愿者？ 2630. 说来也奇怪，怎么全国人都不喜欢你呢？一个两个是特例，全班人都讨厌你，不考虑考虑自身原因吗？ 2631. 还是好好建设自己的家乡吧 2632. 所有的成都人节日三天都原地不动，不出成都不出川，从老到小无条件配合。这三天他们本来可以出去玩儿，现在到小区楼下都会权衡再三，就是为了不给成都添乱，不给中国添乱！ 2633. 你不配带章北海的名字，他也不是胜利主义 2634. 害，只能说是一部分上海人搞臭的名声却让整个上海背锅。 2635. 上海人真的完美的诠释了什么叫矫情，呵呵。 2636. 俞忠华把菜还我❗️俞忠华把菜还我❗️俞忠华把菜还我❗️俞忠华把菜还我❗️俞忠华把菜还我❗️俞忠华把菜还我❗️俞忠华把菜还我❗️俞忠华把菜还我❗️ 2637. 加油 2638. 他们能渐渐从心底里改过自大，排外的毛病也行吧 2639. 新闻只分真假，不分正负，所谓的正负还不是由利益相关者定的？ 2640. 感恩 2641. 被感动到 2642. 这次的上海疫情外地人和本地人就分的很清楚，以往别的省份不管哪里人你在那你就代表那个省份 2643. 对，无论如何，这种时候是应该多一些正能量。 2644. 希望部分上海人能够改正，不要辜负外地人的好心帮助 2645. 这小区住的都是外地人 2646. 为支援的医护人员表示尊重。但上海真的。。。。作为一个平时很冷静的人就想当一下键盘侠。在上海的人们别乱跑，不要祸害全国各地的人们。真的是麻了。少给全国医护人员添麻烦。好好治病为上 2647. 是的，理性评价，就事论事，不煽风点火，方为正道。 2648. 我就想说四个字，定向援助 2649. 想要不被骂上海zf还是好好做好防疫吧小阳人别到处跑了 2650. 借楼借楼，关注一下嘉定区新源路1227号，宾馆吧，一直有阳性，但今天又不核酸了 2651. 其实就是因为有这些少数的新闻才知道有这样的弊端，才能让中央管理一下，不然都闷声的话，情况说不定越来越坏，中国是个整体，虽然存在这样小部分人的问题，但是也不能否定大多数人感恩的心和各地支援者的付出，还是希望中国每个地方的疫情早点结束🙏🙏🙏 2652. 矫枉必须过正！这个时候给上海开脱只会让上海更差。 2653. @守护天使hg：学深圳，不要学香港，加油大上海 2654. 精准防控去哪儿了啊？ 2655. 感谢的是为人民付出的每一个值得尊敬的人，骂的是不作为或者没做好的zf及其他。不冲突啊我寻思 2656. 其实大部分人还是很好的，少数不理智的人夺走了大众的视线，很容易让人忽视大多数理智的人。总之，上海加油啊！之前看到说上海一天要接收很多香港的飞机，也不知道是不是真的，如果是真的，希望还是能多多体谅，市民是最惨的，重要的还是督促上海政府做事 2657. 2020那么严峻上海都有外卖可叫，2022却啥也没有 2658. 第三段逻辑真叫我尴尬 2659. 不怀好意影响防疫的人有，但更多的还是那些心存感激的人啊，虽不是上海人，但还是想对上海说：上海加油！！愿所有人都能平安！！！#上海新增268例确诊13086例无症状# 2660. 下一个武汉 2661. 上海不像广州北京经历过非典，知道严重性，所以一直都还比较严防，上海没经历过大传播，防疫松懈是自然的，全国城市都应该值得深思。有人说上海也应该享受武汉被全国支援的待遇，但也要拿出武汉封城的魄力。经济固然重要，但人命关天。 2662. 在说什么都错的时候，闭嘴就行了。不是只有你们委屈。只是事不关己你不知道而已。你去看看，哪个有疫情的城市没被骂。扬州去年封城一个多月，足不出户。一个没有外溢，一样被骂的一塌糊涂。好像我们的领导，我们的居民就是落后的代名词。我们也一度怀疑自己的城市。结果呢？现在明白了，到哪都一样。 2663. 上海的城市形象因为这次疫情给国人展示了另一面咯啊！ 2664. 现在就开始控评了，骂东北的时候就不见控评，真是经济基础决定上层建筑 2665. 也希望护城宝清醒一点，别和上面的人共情了，上面烂了第一个害的就是你们啊。 2666. 新闻没有正面负面，只有真或假 2667. 安静点吧 2668. 加油上海 2669. 为什么这次那么严重？ 2670. 赶紧控制住疫情好吗，控制住外溢才是本事，人家浙江一有不对劲，立马反应，只进不出，然后控制住了，学学吧，学那些西方的傲慢找骂吗，明明一个喝豆浆的胃，非要喝咖啡，能消化吗🙉 2671. 你在不要说废话了，如果管控到位怎么可能成现在这样子，你们上海和国际接轨搞特殊化，疫情管控也搞特殊化，那些大白真是白费力。 2672. 我湖北的，感觉没必要这么针对，当时武汉还有湖北其他城市被骂的要死也过来了，多一份宽松吧，加油 2673. 值得表扬的是上海人民，挨骂的是上海zf，有问题么？ 2674. 为什么不是上海宁也知道小赤佬这个词 2675. 以前我们只要制作宣传片就把上海当成重点内容强烈建议以后再搞宣传片淡化上海什么东方明珠的镜头给个一秒就行了。英雄的武汉包容的南京秀美的杭州活力的广州沉稳的西安浪漫的成都不值得宣传？非要惯出一个傲慢的上海？ 2676. yes，外地人不给菜吃 2677. 他们不跑毒，我同学能封家里上不了学？ 2678. 武汉疫情时也是被骂，郑州疫情西安疫情全被骂，我认为这是同一群人，而且是现实生活中过得相当不如意之人！ 2679. 不是因为少而震惊，是因为这种阴暗面的事情媒体终于报道出来了，没有人压才觉得震惊 2680. 在上海的外地人表示没有多余的存粮人已经饿得不行，菜也抢不到，封城可以，别把外卖停了啊 2681. 上海的班子真硬啊，就这样也没问责 2682. 😔😔😔😔 2683. “之所以让人愤怒，是因为少”这逻辑不对，让人愤怒是因为不正常，不应该，不符合中华民族传统美德。上海一直站在国家前沿，较其他省市有着方方面面的优越性，享受了其他省市没有的待遇和资源，这也意味着对这个城市有更高的要求。这不是地图炮，而是大家太失望了 2684. 一些人吧，就是想在祖国危难时刻搞分裂我不说是谁 2685. 老摊老摊 2686. 我相信大部分上海人是这样的 2687. #从上海20平米中风险区看精准防疫##上海为什么有底气划20平米风险区#去看看当时上海某些人和当地媒体是怎么自夸和嘲笑其他城市防疫政策的。 2688. 为什么不多报道支援上海医护人员，忙得连喝口水的时间都没有。 2689. 滚吧 2690. 我觉得你发的这个视频，才是所谓的“少部分人” 2691. 吵死了 2692. 图片评论¡评论配图 2693. 只要是事实就是好新闻，啥时候新闻还分正负 2694. 厉害了，我的国 2695. 🐴上海的，很多都是同样的话术，还有收钱的¡评论配图 2696. 我笑了，天天发菜歧视外地人，这特么人家捐给上海的，不是上海本地人的，天天特么还不让封城，还特么乡下人的三瓜两枣 2697. 好恶心，怎么不集体唱听我说谢谢你 2698. 说事实，4月2日，上海已经有多少例了？没有高风险地区。从上海还有坐高铁来广州，又去珠海。上海的管理怎么了？哪个城市的管理象上海这么不负责任？上海的封城就很辛苦，别的城市因为上海封小区就不辛苦？将心比心，换位思考，很难吗？ 2699. 国际化大都市封城使不得 2700. @ItsWangyl@Ur-ConfessionK17看点正能量 2701. 虽然但是有的负面新闻确实让人很下头啊 2702. 半封城能管住本地人与外地人只进不出。全国多地早在援助上海，而上海始终拒绝半封城持续荼毒全国经济与民生，且上海市民曝光的流调录音指向核酸结果批量造假，核酸阴性就能出沪，这让流入地如何自处❗不是地域黑[嫌弃]上海防疫相关负责人份属为美国病毒美国特效药开路的内鬼，遭到审判都不解恨 2703. 部分人有问题可以去指着他们鼻子骂人，而不是把所有上海人归为一谈，还不让还嘴，谁让你是上海人。照这些地域黑的逻辑，那这种丢人的上海人不也就是中国人吗？那遇到外国媒体把地域黑变成中国黑的时候，中国人能还嘴吗？ 2704. ？ 2705. 喜欢哗众取宠？以偏概全？？是你们对上海长期的偏见还是，真的愤愤不平，一边骂着上海，一边吃着上海带给的利益.尖酸刻薄真令人心寒…. 2706. 少部分？极端？宝山区一整个区的人民叫少数？封闭29天没物资依旧严格遵守足不出户规定，仅仅只是为了活命而在网上求助，在社区疾呼叫极端？ 2707. 这个最起码的说声谢谢，仅此而已 2708. 这是wb，骂人才是主流 2709. 洗脑了哦 2710. 看了评论，我只想说。对于支援者，滴水恩当涌泉报。冷嘲热讽者，虽远必诛！ 2711. 窝里斗要不得啊 2712. 建议上海在核酸检测点把党旗都挂上，看着就安心 2713. 上海为什么不封城？长痛不如短痛的道理不懂吗？疫情外溢导致其他省变成高风险地区，离谱的是上海还是底风险地区，这样下去疫情蔓延，那全国人民三年防疫疫情是为什么，国家为得新冠肺炎的人免费治疗是为了什么？医护工作者、志愿者…昼夜不分的工作又是为什么？全国人民打疫苗居家自我隔离又是为什么？ 2714. 哈哈哈哈，小区业主集体骂居委会的视频怎么没挂上来呢 2715. 求你们别出门一个人搞烂昆明 2716. 你问志愿者怎么想？你亲自问过没有？他们这么辛苦是因为哪些人？你有什么资格代表他们？ 2717. 如果思想不统一，在共存和清零间左右摇摆，认为不过是流感等等，全国支援也难回天。 2718. 说得对啊，三次元比二次元可爱多了，就是因为三次元看不见网络喷子 2719. 负面都是上海外地人本地人很有素质捏 2720. 最好上海永远封城封下去，把病毒熬死，这样其他地方就安全了，我觉得现在还不够严厉，一定要死死的封住不动摇，之前的官员都思想觉悟有问题 2721. 负面确实搏眼球，同样正面也只是一部分而已 2722. @英特纳雄耐尔什么时候实现强国有你，但也要做好防护，新增一万多 2723. 这回上海该放下他们的自大和媚外好好反省下到底是谁在就他们的命了吧 2724. 上海人歧视外地人，真厉害阿。 2725. 啧……这次浦东和宝山的物资发放到底在搞什么鬼¡查看图片 2726. 我是不理解，你这个红v怎么每次rs都有你在，一直在搅风向 2727. 没办法我就是被恶心到，我们山东去驰援，上海给我们送小阳人呢，还叫我们山东大妈流落街头呢真尼玛让我恶心呢上海 2728. 你说想咋样，那你说上海想咋样嘛？为什么他们还是没有高风险?为什么一开始坚持不封城现在又封了?为什么明明是阳性的人还能到处跑到处跑到处跑!!!为什么啊？更重要的是为什么到现在还没有人为上海现在的乱象负责啊？ 2729. 不能因为一点恶就全盘否定善的存在但我相信还是有大批地狱骑士的 2730. 我们也想感谢，但是被封20多天没人理，核酸到现在也没给做，大礼包被派给了别的区，这边菜叶子都没有，让大家怎么夸？ 2731. 这种截图不知真假不予置评，承认有的上海人排外，素质低，有的政府不作为，但是其余水深火热中的上海群众是无辜的啊，疫情面前就不要内讧了团结一致抗疫吧¡评论配图 2732. 博主真能洗坏的直接选择性忽略了呗 2733. 上海加油赶快好起来 2734. 大家看看孩子吧八十个抗原异常实在没办法了没人来给异常的做核酸一直和的一起住现在全宿舍估计都已经感染了主要还怕传染给别人 2735. 武汉疫情的时候，上海是第一个支援武汉的，真心希望这座城市好起来～ 2736. 什么叫否定，没有人否定他们的辛苦 2737. 别的城市疫情：这政府做的啥事啊，防疫做的那么差，怎么办的事上海疫情：哎哟上海人真是高贵，阿拉桑海宁真是人上人，不愧是“国际大都市”看不起乡下人，现在跑到乡下来祸害别人了？上海看不起外地这思想都是20年前某个人写的书了，到现在还有人信的吗，制造人民矛盾开地图炮很爽是吗，带点脑子吧 2738. 我們已經被罵過了……上海你們也要堅強挺住 2739. 只是不希望有物资烂掉没人管，但又有人没饭吃，没物资，只是希望有关部门可以做好，新闻没有正面负面，只有事实！难道表面风平浪静，内里波涛汹涌才是好的吗？你能想象被封在家里没东西吃的无助吗？？？？？？？？笑死人了真是 2740. 东佰胎君吃💩 2741. 负面报道在哪？你给我去热搜上找个上海负面的词条看看啊让我见识一下。年初西安疫情鲜明对比，经常热搜前十近半是西安负面词条，上海出了那么多魔幻事件，到现在一个爆的标签都没有….负面的上刚热搜没多久也会迅速灭火消失。被“保护”的这么好就赶紧感恩吧、别得了便宜还卖乖。 2742. 别洗了吧，其他城市爆发疫情，全国都是声援支持，唯独魔都被骂，有些人就不思考原因，总把问题归结于正负面，少数人 2743. 党的诞生地～上海加油！ 2744. 希望多报道一些这样一正能量。你知道嘛？全国人民都很关心你们，都在注视着你们的一举一动，上海人民是最可怜的，这些大家心里都有数。 2745. 负面是最需要关注的，才是解决问题的。而且也也不少！ 2746. 求你们别出门，三个人搞烂一个成都。 2747. 没去过上海，最近看到很多人在骂上海，也觉得不太公平。大多数人都在尽守本份，该隔离隔离该做事做事。很多问题都是管理层的原因吧，比如疫情扩散出去，外地人没有菜分。这都不是普通百姓能左右的 2748. 我们同事的小区，业主群自发组织的 2749. 有什么好洗的，上海ex 2750. 当时西安疫情，一共两千多例，被全国骂的半死，从西安zf上升到西安人民，西安人没素质乡下人，西安人都是护城蛆，真的很无奈我们没有外溢更没有骂别人…..同一个中国我还是希望上海能早点回归正常（地域歧视那些人除外） 2751. 居委会的视频呢 2752. 感恩感恩 2753. 谁在骂志愿者？不都在骂某些人防疫不力吗 2754. 当舆论开始一边倒的时候，有脑子的人会思考，无脑的在跟风。 2755. 彩排就彩排，说得那么好听干嘛！ 2756. 我们这有一个从上海回来的，全市全省都在为他的行为买单 2757. 经济学家吴女士怎么不出来说话了 2758. 还有无脑喷子说这是外地人在感谢，因为讲普通话 2759. 宝山区大场镇到现在没有发过一根菜叶子！ 2760. 有道理 2761. 我不是不能接受负面消息，但是别人骂我们，我很气（我有在个人调适） 2762. 你发个微博不加咒语是想炸鱼吗 2763. 听我说谢谢你 2764. 这世界不是好人多，是正常人多。拒做核酸被执法人员带走的人有，但绝对是个案，绝大多数人配合医护，还发自内心的表示感谢。#上海新增本土确诊268例无症状13086例# 2765. 好假 2766. 听我说，谢谢你 2767. 唱歌、谢谢。。上新闻。。确定不是作秀？现在有些人很着急要扭转舆论吧？ 2768. 哇哦真下头//@shawblueChelsea:你说的投毒的那些人，人家都是回老家，回户籍所在地，和上海有什么关系？难道他们出上海是全上海人同意的？//@笑盈盈哇:很客观。从来没地域攻击过全上海人不过到处投毒的真的很欠 2769. 少部分？你这是少部分吧！你快来看看大部分什么样吧，令人作呕！恶心！ 2770. 现在评论里不还是老乡老乡外地外地的，上海现在被骂的这么厉害除了防控不到位不就是这种高高在上的优越感，我建议正常上海人应该比其他地方的人更狠地先骂这种人而不是一边叫屈一边暗戳戳附和 2771. 有则改之，无则加勉，学过么？ 2772. 表达谢意都要被说成是作秀，某些人为了黑而黑的心态昭然若揭 2773. 你瞎了 2774. 一切看似末日的，终将被证明是过程 2775. 想起广西百色疫情的时候那些骂声，现在只想呵呵… 2776. 没否定所有人，但是你负面的信息比正面信息不成正比，抛除带节奏的嫌疑以外，试问本身真的有反思过吗？都这么多天了 2777. 新闻就是要说实话 2778. 有些人不用特意说风凉话，不好的该骂就骂，好的该夸就夸。实事求是 2779. 防疫政策确实很拉垮，我男朋友的室友核酸异常但是不通知和他合租的人，只通知本人，也不拉他去隔离，还让他们一起住着，直到他那个室友出现反应，自己说自己核酸异常，真的不懂这个防疫是在干嘛 2780. 少？呵呵哒，你看到的只是被允许看到的，还有大堆不被允许看到听到的吧。 2781. 因为去了几万医护支援才好的，因为不好才去了几万人支援，你懂吧 2782. 多一份理解，疫情面前大家都是受害者，没必要上升到地域问题上，大家都是中国人，互相包容、共克时艰！ 2783. 看哭了我们也在家隔离，上海加油 2784. 又内卷了激化内部矛盾现在是团结一致的时刻！ 2785. 上海人也在骂上海政府啊。做官为民吧谢谢 2786. 我同学很多都是志愿者，能体会大白们的辛苦和承担的感染风险。由衷的敬佩和感激所有尽心尽力的防疫人员。 2787. 上海东郊仁恒，是真实的，我朋友住那个小区 2788. 谁先开炮的啊？ 2789. 都来骂吧，我无所谓了，麻木了，前几天还和人对线，现在我只会对骂我们的人说南波湾你说的都对。 2790. 一天检测2500万人，本身就是艰巨的任务，也是巨大的成绩。世界上有别国能做到吗？为人民生命安全倾尽全力，只有中国真能做到。咖啡很好，要有命才能喝到！ 2791. 其实疫情爆发，各地都有负面新闻，各种问题都会暴露出来，都会被骂。比如西安疫情时大家也骂，吉林疫情现在大家也骂。但说实话，虽然我认为是个例，但真的还是第一次听说大家骂这个点：发菜还区别对待本地人外地人的。O网页链接 2792. 同行四五个人在一个房间隔离，其中有一个确诊了！两天了都没有医护人员来转移！现在四五个人都感染了，120打了，急救中心电话打了，市长热线打了，都无济于事啊！求求救救我哥哥吧[泣不成声][泣不成声] 2793. 少部分，但是竟然能铺天盖地，所以说，你说的这少部分其实就是大部分，措施没做好，连措施方案都没具体针对性，那么多案例可以去参考，上海政府也可以咨询之前的省份做一些参考，但是给人的感觉就是没有具体步步落实到位，别怪对上海人心寒，但的确很多没做好 2794. 这篇文章发的，怎么了，上海抗疫管理不到位，居民反应，就是负面新闻了？天天报道那些有的没的，有意义吗？ 2795. 再探再报 2796. 别叭叭了，人家只想早点回家 2797. 说得好 2798. 博主挺住，已经有两个被删了微博 2799. 只有正面的才叫新闻吗 2800. 那你天天沉浸在“正面新闻”里作秀没人管你，你也管不着别人 2801. 呵呵。坐标宝山顾村馨家园二街坊。楼栋里好几个阳发现几天了也没人管。没有被拉走。也没有物资。并不是所有人都能得到帮助。请大家看看我们吧。 2802. 当这些负面问题层出不穷的上新闻上热搜的时候，其实是因为脓包太大了 2803. 上海检测三天出不了结果，昨天武汉医护给测的，江苏给检的当天出的结果，听说江苏援护整完连夜回去了，做自己的事情了！！这效率真是牛逼 2804. 一句谢谢，就把所有不好的给掩盖了？ 2805. 蟑螂效应 2806. 利己主义 2807. 想起了我们西安封控的场景上海加油 2808. 上海两个问题，1傲慢，封晚了一个月，2偏见，本土人十分排外，援助物资从来先分配本土人再送到外来从业者手上。我表姐上海工作10年了，隔离的那片小区全是外来从业者，一星期发一把挂面，两个西红柿，一瓶奶，两个鸡蛋。让吃一个星期。有钱不让自己买，别人送吃的物资也不让进。 2809. 你本来就做得差啊哪来的负面新闻 2810. 有几个是本地人百分之八十都是外地的 2811. 现在这个政策就等于脱裤子放屁多此一举说什么不封城两边封这不是搞笑吗小区封14天过几天又说潜伏期又封说什么浦西封5天物资也没有我们小区毛都没见到也不知道那些物资资源的菜去哪了我真的是无奈了 2812. 有种你家人孩子永远别来上海读书工作做生意，网络喷子们，生活中的失败者，戾气都很重。 2813. 坐标上海宝山区，救救孩子吧，我已经关在家里10天了，吃的马上都吃完了，救援物资迟迟不到，难道真的要饿死吗？ 2814. 哈哈哈哈只有这样的微博才能留下来 2815. 我表弟的同学现在是一名志愿者，忙了回家，就是休息，没有时间看微博，看抖快，觉得智能机就是个老年机来用。 2816. 这不就是摆拍 2817. 为什么别的地方没有这种负面新闻单单上海有 2818. 昨天我们小区做核酸的时候志愿者跟我嘱咐到“大家做完记得说谢谢今天是外省市来的医护人员”，当然不只是这次其他每次做完我都会记得说谢谢，所以看到评论里说的什么形式主义真的很伤心不管怎样都是大家真心想要感谢所有医护人员，上海市本地人每天战战兢兢地隔离上网又看到铺天盖地的骂声 2819. 都这种时候还有那么多人骂，有的号一看就是专门骂，感觉你们是美国派来离间中国内部的？ 2820. 那每天确诊病历数也不要报道了，多负面啊！天天报道志愿者辛苦就好了，感动中国 2821. 求求上海人别到处乱跑！ 2822. 三张官方讲话的图片，无论是转发微博还是微信，都会被夹，可见他们ds内心是多么恐惧大众的察觉和觉醒，是多么的没有安全感。不过媒体这一块，还没有收回阵地，目前还在ds手中，无论是国内还是国外。​ 2823. 不需要谢谢，需要你们唱歌 2824. 我们希望看到更多上海好的处置，好的办法，好的事情，但是好像已经没有什么公信力了。希望上海挺过来，也希望上海能除掉蛀虫垃圾！上海加油！ 2825. //@直到遇见最好的你:什么叫做负面新闻，新闻没有正面负面，只有事实 2826. 大家都做听话的乖宝宝，社会就变和谐了吗？ 2827. 民众越来越像国外了果然国际化 2828. 我觉得ta可能想以后不玩微博了 2829. 狗咬人不新鲜，人咬狗才会有人看，这就是现在的自媒体思路 2830. 现在人的思维都被舆论控制了，风往哪吹就往哪跑，唉。 2831. 假象 2832. 没有否定所有人啊，只是并不是你所说的那样，真实情况是极端的大部分负面情况。有问题肯定得解决啊，但好像并没有解决。所以，请你不要睁眼说瞎话，自我麻痹有意思吗？ 2833. 这次疫情可以看出上海人素质全国最低 2834. 这一次的疫情，上海应该要撤掉一批领到了吧，真的，太失职了！ 2835. 也转发点正能量的 2836. 我买不到菜…… 2837. 感人，上海加油。 2838. 中国加油，上海加油 2839. 感谢说出了我们的心声，上海人最近给骂成了狗，实际上上海除了本地人还汇聚来自全国各地的人，上海人大多和善友好，根本不是负面新闻报道出来的那样，那只是极少数！ 2840. 我真的谢谢你们啊 2841. 但是他们的政策是针对外地人的 2842. 吉林加油 2843. 这种安排好的正能量拍摄，何必了¡评论配图 2844. 为什么这样了不封城？既然不封城那就全国放开啊？整天封校算个屁，zf不觉得自己做法双标吗？？ 2845. 笑死上海做的差是事实咋就被你叫成负面新闻了 2846. 别的地方稍微出一点问题就看看人上海怎么怎么厉害，现在出事了咋不让说了，接着吹精准防控啊，真就亲儿子其他地方都是后妈养的呗 2847. 等好了，就翻脸不认人了 2848. 真不想替上海说半句好话，牛鬼神蛇一堆摆烂叭 2849. 这个新增是有多滞后，我们这一片那么多问题（🐑不拉走，物资没有，老人小孩、孕妇都处于恐慌中），都没问没人管，长宁区虹桥街道。 2850. 事实就是负面？我的天拿了多少钱 2851. 不管好的坏的，只要是事实就可以报道，好得表扬，坏的批评 2852. 非常感谢医护人员，感谢外省市的支援 2853. 太可爱了 2854. 依然重症率为0，死亡率为0，一些人老说中国爆发疫情死亡率会多少多少的人是何居心，巴不得中国多死人是吗？ 2855. 粉饰太平不是解决问题的态度 2856. 中国人的浪漫，是刻在骨子里的 2857. 为什么还不封城？ 2858. 这条多少钱？发出来兄弟有钱大家一起赚 2859. 微博全限了 2860. 喊的不是阿拉上海宁最看不起你们这帮农村人？我不是很认可 2861. 全小区要求这样做的 2862. 上海人是自私鬼，外地援助物资竟然没有外地人的份。领取物资还要看户口，这应该是从疫情爆发到现在第一次看到封控区这种做法。 2863. 摆拍的 2864. 我只想问问到底是什么人做的防疫政策？居民一直买不到菜，到底怎么生活？如果一点粮都没有了，谁来管我们？ 2865. 政府管控不力才导致从几百到几千现在上万，更让志愿者伤心的不是你们自己吗？外溢严重，动辄就说是外地返乡？只有上海有外地人，北京没有深圳没有广州没有？没有一个在外溢的时候打出外地人的旗号！被地域炮轰委屈吗？被赶出小区的大姐什么心情？临沂自己接回去什么心情。自己反思一下吧。还有脸问？ 2866. 上海肯定还是会有很多普通善良的民众在的，但是一部分人还有上海政府真的很败好感所作所为让人不免产生有色眼镜。就拿知乎来说，一月份有营销号夸赞上海精准防疫拉踩其他城市，评论区里是上海人的狂欢，有人表示担忧都会被阴阳怪气。现在失控了那些人依旧傲慢且死鸭子嘴硬 2867. 大可不必 2868. 因为微博网友解决一切问题的方式是：骂人，拉黑，投诉，举报 2869. 这种事情多了还了得？ 2870. 都服了，什么都要喷一下，实在不信去看直播好吗？总是被别人牵着鼻子走… 2871. 过滤过了新闻，过滤过的信息，有意思吗？ 2872. 我看到我看见的事实，而不是胡编乱造的视频，谢谢 2873. 这不是居委会发通知的那种作秀？ 2874. 政府的行为百姓买单 2875. 学学新闻的定义再来说话，还负面新闻，可笑！ 2876. 上海公务员只招上海本地人以及非上海户籍的硕士研究生及以上。 2877. 好家伙还特意整个无人机拍一下多少有点刻意了🙏 2878. 回复@Dawn7777777777的赞:握手 2879. 不是吧，不会有人不知道媒体是被把控的吧 2880. 浦东现在还有没发菜的地方，一点消息都没有，人家不上网说饿死啊？ 2881. 不是因为负面新闻少才吸引眼球是因为吸引眼球了才能有人解决走头无路的人只能靠这种方式获得帮助想想也挺可悲的没有人说一线人员不辛苦如果一切都井然有序的话我想一线人员和患者也都能轻松不少吧 2882. 为啥视频的配音变了还是不是一个小区 2883. 其他地方让人愤怒的负面新闻都是官方抗议不利，只有上海的负面新闻是官方民间两开花。 2884. 菜都买不到也不想管负面正面了！ 2885. 现在就是做不到及时隔离阳性患者 2886. 说这么简单……即可这两个字轻描淡写哈哈哈我服了 2887. 方舱都来不及建，为了完成整治任务，把老百姓就往工地一样的方舱扔，上海真是三线城市都不如了，真是太让人失望了 2888. 你不是切断150人或者1500人传播源，这种数量级的想切断，没这点人手来办事儿，再拖一个月岗位上的至少五分之一扛不住疲劳工作要退下来 2889. 我们小区阳性还出来遛狗 2890. 求求别发这种往自己脸上贴金的消息了，是不是要让上海人民为你们辟谣一下？？？ 2891. 老实卖你的茶 2892. 一天天的，就会捅词 2893. 小区很多阳性都运送不及时，也不消杀。导致不停核酸交叉感染。 2894. 所以究其根本，是老百姓的错？上海对待民生的态度可远远不及二三线城市，没有一点人情味 2895. 我们处于上海市宝山区大场镇场中路4098弄23号城家公寓，4月2日核酸检测已确诊一名阳性，截止4月10号一直没有转运，目前我们公寓已经出现了所有管家保洁人员全部核酸异常，3例阳性6例待复核，公寓排风系统是相连的并不具备安全隔离条件，已经在公寓发生交叉感染，如疾控中心再放置不管后果不堪设想！ 2896. 好好好，上一次说转折点好像还没几天吧？大概一个礼拜前说的？ 2897. 问题就是很多确诊的都没拉走，就怕这样错过一个可能进入拐点的时期，然后接着爆炸。。。 2898. 我看了一个视频，毫无人性的措施，婴儿集中隔离，母亲打疾控没人接，都快要崩溃了。还有方舱真的是脏乱差，哪个城市这么整的！ 2899. 我不明白，为什么我们低风险区因为上海的外溢哪也去不了，封快递，封学校，封外卖，上海的人却想去哪就去哪，我们的自由就不是自由了吗 2900. 上等城市就是不一样 2901. 建议把阴性运走吧TM的小区一天阳性1不消杀2不运走 2902. 小区还有住户🐑，不肯走 2903. 切断传播源，把阳性患者隔离进行治疗，这招真高明为深圳医生点赞听起来简单用好这招难！ 2904. 楼里的人一个星期前开始、陆陆续续的变成阳性，却都无法转移！感觉大家都被封在楼了养蛊！不见转移！又看很多网友曝光被转移的人被转移到未完工的方舱，都不知道自己该不该希望阳性患者被转移，还是让自己继续被养蛊，阳性患者貌似在家养着更舒适，但还没阳的还是有被传染的风险。真的真的太难了 2905. 还有做核酸感染上的 2906. 我们小区到昨天还在新增。。。越增越多。。。 2907. 这是要在公布昨天新增数量前先给大众打个预防针么我能理解成昨天确诊很多么 2908. 不过华师大party开的倒是不错昂 2909. 能不能别再说废话了让大家看看真实情况 2910. 切断传染源，及时隔离感染人员简直就是个笑话。6号核酸检测结果一直不出，昨天才打电话告诉我结果异常，我又不知道我异常，7号我又下去做了核酸，上下电梯一堆人。到现在也没出复核结果，就这速度怎么切断？？？ 2911. 还有彻底消杀，求求了 2912. 小区29号的阳性还没有接走，我们这边天天抗原，核酸不停，解封无期限 2913. 呵呵。全城封闭，麦德龙还那么多人？说不定就是这样子传开的。那么多天还2万多例 2914. 四月四号说好的全员核酸36小时全部完成。结果连续三天两万多这是做了个寂寞啊 2915. 楼里阳不及时拉走，还要强制下楼核酸，不把老百姓的命当命 2916. 阳性病例拉出小区上海一日游，没接收资源，又送回小区，您说这个怎么办 2917. 认真早做隔离不就没这事了 2918. 话都会说，道理大家都懂，然后呢 2919. 都拉去深圳吧 2920. 现在已经来不及了估计会逐步开放了一个国家是整不过大自然的 2921. 说倒是好说，你倒是下基层看看啊，端着做样子谁不会 2922. 疫情在不结束，词都不够用了 2923. 这些发命令的来社区做做志愿者体验一下 2924. 说好的群体免疫呢 2925. 说的都是什么废话…… 2926. 请问因为清零而产生的次生灾害有多少？？？你们统计过吗？现在的病毒和武汉时期的不一样，请实事求是好不好！！！ 2927. 苦不堪言这日子一天天的无作为的人霸凌条款历历在目吓死人人民都在哭泣电话都是推诿和忙音我们怎么办 2928. 现在大家怕的不是阳了，而是阳了之后，政府对阳性人员的管控，就像垃圾一样被丢到不知情况的方舱 2929. 要么你就赶紧共存，要么你就隔离到位。啥也不是，散了吧 2930. 拉倒吧，全员同一天做完再说平台期 2931. 上海市闵行区梅陇镇蔷薇二居居委5个人不戴口罩不戴手套，全程飞沫直接接触抗原，再发放给我们！3.10被封至今最近3天连续确诊阳性上榜！求求救救我们，我们想活着！ 2932. 放狗屁今天我没小区新增一半，居委连个屁不放，都是患者在群里自爆的，还有很多人没爆+拒绝抗原检测 2933. 上海的现状可能是，不想共存，人多已经共存了，来不及隔离 2934. 平台期，那距离高潮很快了 2935. 工作汇报请带上可执行的解决方案1234....谢谢 2936. 集中隔离！只是隔离，不收治对吗？上海方舱情况大伙能关注一下吗？ 2937. 我同事住的那栋楼，有个阳的好几天了都没有被带走 2938. 上海真的不知道说什么好了老百姓水深火热之中了 2939. 快封城吧好吧非得上海搞例外是吧明明自己连防控基本规划没有还自以为是要钱不要命是吧 2940. ？？？ 2941. 深圳政府能不能给上海抄一下答案，再这样下去，我不知道什么才能见到相亲对象，什么才能嫁出去，可怜一个29岁的未婚女吧 2942. 上海清零：把所有小阳送出上海 2943. 全部隔离到家几天了，这数据怎么出来的？ 2944. 你在乱发什么，咋个就到十五万了？累计不是十万吗，不要乱发 2945. 上海出来的已经遍地开花了，一整个大无语，搞到别人也不得安宁 2946. 方舱来不及建，居家隔离已经阴性的强制拉走，挤兑医疗资源救救我们吧，我们被扔到还没建成的垃圾场，至今没有医务人员来看看我们没有人做核酸，没有药，甚至连测体温都没有 2947. 哦 2948. 加油 2949. 我又懂了 2950. 哎别海淘了给国家添麻烦 2951. 听🧱一席话犹如听🧱一席话啊怎么切断传播源？这个应该是外省市该研究的课题，怎么杜绝来自上海的传播源 2952. 家里后面的那栋，不愿意去外地隔离，现在好像还在家里，周围也没人看着，出来了谁知道？ 2953. 浦东莱阳路881弄，小区的阳性拒绝隔离，居委110都是是自愿隔离，除非找疾控强制！人家还晒被子呢！根本接不走，传染源就在小区里，其他几栋也没接走！ 2954. 早干嘛呢，早干嘛呢，早干嘛呢，早干嘛呢 2955. 我的妈呀 2956. 我相信还有很多，只是没爆出来 2957. 平台？怎么确定不是上坡 2958. 我们小区还有🐑的不肯走等着祸害一整栋的居民呢 2959. 高位平台期，但不是高风险 2960. 也许上海有自己的想法。毕竟是天龙人 2961. 醉了央视新闻发布数据瞬间把说数据的评论删除都不敢说上海咯吓死人了 2962. 哦对了，上海都这样了还是中风险耶 2963. 讲的都是废话 2964. 为什么不出病理分级管理啊？轻症可以留在家里自行隔离，腾出医疗资源给重症，也不至于劳财伤民。这个防疫政策要顺应病毒发展呀，新冠不会灭绝，现在不管做什么都是周而复始而已，什么时候上岸？百姓能扛住吗？经济能扛住吗？其它非新冠重症者能扛住吗？ 2965. 集中隔离后呢？弃之任之不管不顾？这就是你说的防疫？可笑可悲至极 2966. 希望疫情赶紧过去吧，同时还是想说一句：感觉还不如禹州市政府给力 2967. 天天叫嚣躺平，同时又怪政府不拉走🐑性 2968. 我们小区从封开始查出来的阳性和无症状到现在都没拉走 2969. 求求大家救救菏泽疫情严重仍要开学，想要变成第二个上海 2970. 🙏🙏早日清零。我不要闭环住宿到高考！ 2971. 砖家又出来了 2972. 可是为什么还在不断外溢呢 2973. 又在那造词了 2974. 说的为时尚早 2975. 又整个新名词，早日清零吧 2976. 河南加油 2977. 这种词有意义吗 2978. 阳的不拉，拉阴的。这是清阴计划吧 2979. 说了个废话，道理都懂，可你们上海人还是往外放人，不封城，不封路，危害陕西西安，商洛周边城市，能不能把上海的人都变成红码 2980. 说实话我这里浙江四线，只是只知道我爸去过密接地点，并且核酸绿码，都在我家门口按了监控。居家隔离。住5楼，每天上门测核酸 2981. 哪那么多新词汇 2982. 你说是就是 2983. 很多居委和物业不作为，管理不好小区，乱哄哄 2984. 现在是人手严重不足，至少要25万人(一个医护服务100个人) 2985. 啥意思？继续高位？ 2986. 3万很快就来了 2987. 如果这么简单轻松早干嘛去了？？b 2988. 卧龙凤雏也是这么认为的 2989. 哈喽？刚刚从上海坐火车来了两位阳性还是平台期呢，你们倒是平台了，别的省份呢？ 2990. 好意思？ 2991. 哈哈哈 2992. 希望我的评论不被精品掉 2993. 鬼扯也信 2994. 说我也会说 2995. 可拉倒吧方仓的铁皮房弄好没啊厕所就一个都没有通水还把人集中在一个地方还集中在一个地方即可控制疫情我真的会谢没能力做好就让人自己在家隔离去里面还不是遭罪服了 2996. 江桥小区，从无到有，直到楼栋被阳人的包围，刚刚下楼竟然还和阳人楼栋一起核酸，让我们死吗 2997. 上海加油！ 2998. 新冠疫情都这么多年了还能这么严重，对比广深的疫情防控，超一线亦有差距 2999. 又创造新的词汇 3000. 为什么上海疫情总是上不了热搜 3001. 哪门子平台期有这么夸张的增幅 3002. 扯淡一天天，张吴国之重器多一点张吴，……国不久矣 3003. 根本就管不过来。太复杂了。这个体系。 3004. 即可？你ta知道上海现在什么状况吗老爷 3005. 上海现在还是中风险呢 3006. 只要切断传播途径，只要保护好易感人群就可以控制，张口就来谁不会 3007. 第一次听说高位平台期。哪个高位，万位吗？那可真是牛逼啊 3008. 还绿？ 3009. 吹牛谁不会， 3010. 每天屁没少放 3011. 上海这波伤害了上海人 3012. 你问问政府官员同不同意 3013. 🤗 3014. 明显的偷换概念把每天高局不下换成平台期当百姓都是沙子 3015. 绝大多数阳都是在封控期间发现的，真的不用反思自己的防疫政策吗？朋友的小区单元楼是3月15号左右封的，除了核酸没出过门，4月5号确诊！我都惊呆了，是怎么感染上的呢？ 3016. 高位平台？意思后面几天差不多也每天这个数？ 3017. 家人们别再相信阳的有饭吃，阴的没饭吃啦，事实是阳的也没饭吃朋友在闵行颛桥中学隔离点，今天的中午饭是17:08发的，拉走5天了，现在仍没有热水，喝的热水。其他的更不用提啦，什么洗澡换衣服，不存在。 3018. 两年都没回过家了，shzf能不能做个人！我只想回家！ 3019. 天天喊着早上清零，也就是个念想了吧，遥遥无期等着吧 3020. 平台期吗我怎么看着还是上升期来着 3021. 来上海看病被传染，目前阳性，医院不管！让我联系自己当地社区！ 3022. 别骗自己了，这不跟美国类似，只会越来越多，高危平台期？笑死冠冕堂皇，哪个城市不是控制住传染源就好了？问题是你们大上海多少人被感染了？怎么控制的住？你要是从一开始就限制出行还能有这事?经济中心？你那隔离的治疗的不会给国家造成负担？ 3023. 摆烂呗 3024. 我默默说一句，他妈的，已经没词可用了 3025. 我他妈迷惑了，这明明还是高速增长期 3026. 只要做到 3027. 上海到现在还没一个高风险地区，完全不慌 3028. 现在看似中央和上海政府出台很多措施来清零和保民生，但是下面的这些🐷没一个知道怎么执行，连转去哪个方仓医院估计都没想好，特别是一些区的管理简直令人发指。说实话要是放在美丽国，估计都已经被散弹枪喷死了 3029. 这又是什么词 3030. 何其有幸生于中华 3031. 这又是什么新词？ 3032. 废话 3033. 听上去，像是说了，感觉说了又等于没说呢 3034. 中文博大精深 3035. 你说话就好像放屁。 3036. 到拐点再说，文字游戏一套套的 3037. 全把人拉到建筑工地去隔离，也没医生治病。还不如自己在家自愈，真搞不懂shzc这么装是在演给谁看 3038. 我这小区天天新增，你说这话可信吗 3039. 現在陽性不能及時轉，最重要的消毒都不做，怎麼好轉 3040. 志愿者真的很辛苦很辛苦的！有些志愿者带200张医保卡来帮邻居开药。 3041. 所以就不能每个小区出一个有医师经验的买药员，非得把时间浪费在不同小区之间奔走？ 3042. 不容易啊，辛苦了 3043. 厉厉我发个贴我的邻居上海抗疫求助超话坐标西康路989弄邻居家一个老人一对新手父母出生几天的新生儿，月嫂昨晚进小区到他们家，今天抗原阳性，打电话求居委会疾控中心尽快复核转运走就让等着。他们家只有一个卫生间。大人不怕但是婴儿刚出生几天太脆弱了，求尽快复核如果确阳转运走～ 3044. 唉好辛苦啊，希望能快点好起来 3045. 图二推荐'22最佳图片 3046. 志愿者太辛苦了开车一定要慢慢的 3047. 不容易 3048. 我帮我朋友在京东上买的药真的也快，其余的快递都因为疫情拖延了只有药从拍到收到货3天 3049. 看到了。岁月静好。 3050. 辛苦了，谢谢你们 3051. 每天高喊“此生无悔入华夏来世还做中国人” 3052. 辛苦了，在上海奋战的志愿者和上海人民一样，都是中华民族的英雄! 3053. 感谢为疫情辛劳的每一人 3054. 失望至极，我的生活全乱了 3055. 一些人在感动上海，一些人在敢动（伤害）上海 3056. 那些城市管理者情何以堪，造成这样难道不应该负责吗？ 3057. 辛苦了 3058. 辛苦啦 3059. 辛苦了，愿疫情早日散去🙏 3060. 配送员真的太辛苦了 3061. 转发微博 3062. 这是软广？ 3063. 不容易，了不起的配送任务🙏保佑疫情尽快过去🙏 3064. 好难，大家加油！ 3065. 辛苦了！ 3066. 辛苦了🙏 3067. 希望多给他们发奖金多给他们发补贴，等疫情过后他们的福利待遇能跟上 3068. 药品当然是上海人民所需要的 3069. 现在的物流卡控的是蛮紧的 3070. 我觉得那真的是非常的伟大 3071. 那就多招志愿者啊，多派人手啊，好多毕业生也找不到工作，给个机会锻炼啊 3072. 还有很多买不到药的老人小孩怎么办呢 3073. 辛苦了辛苦了辛苦了 3074. 苦的苦，日进斗金的进斗金 3075. 平时养尊处优这时候别抱怨。真正辛苦的只有真正的志愿者。 3076. 志愿者辛苦了！ 3077. 那一刻，内心已经被击中了 3078. 这样的行程，他们居然坚持了这么多天 3079. 我们公司几家上海的药房也是线下一直所在区可以派送但是线上发不了好多人来问催发货一动不动就投诉说实话作为客服我都怕上海的客人动不动就投诉 3080. 让那群做个人就能早结束了 3081. 辛苦啦 3082. 生活中还是有很多美好！ 3083. 上海加油 3084. 上海加油，每一个妈妈加油！ 3085. 谢谢你们的平凡而伟大希望上海疫情早日结束 3086. 太不容易了 3087. 上海，一切都会好起来的🙏，加油💪 3088. 看到视频最后好心痛一切快快好起来 3089. 上海棒棒哒🎉🎊 3090. 辛苦了注意安全 3091. 上海加油 3092. 生活中还是有很多美好！ 3093. 上海加油 3094. 上海加油，每一个妈妈加油！ 3095. 谢谢你们的平凡而伟大希望上海疫情早日结束 3096. 太不容易了 3097. 上海，一切都会好起来的🙏，加油💪 3098. 看到视频最后好心痛一切快快好起来 3099. 上海棒棒哒🎉🎊 3100. 辛苦了注意安全 3101. 上海加油 3102. 6月1日起，上海有序恢复全市正常生产生活秩序 3103. 复工啦！ 3104. 最美上海 3105. 上海 3106. 坚持清零，复工有望 3107. 恢复生产啦 3108. 上海恢复生产 3109. 中国加油，全域清零，指日可待。 3110. 恢复生产，拉动经济，上海最棒 3111. 万众一心，共抗疫情 3112. 坚决遏制疫情蔓延，从源头控制住 3113. 守护好来之不易的胜利果实 3114. 各行各业积极复工复产的同时，上海也从政策层面上下功夫，为统筹好常态化疫情防控与经济社会发展做引导、疏堵点。 3115. 随着上海疫情防控形势稳定向好，市民复工复产的出行需求不断增加，上海城市交通动态调整，适应各方的需要。 3116. 自此轮上海疫情社会面清零以来，城市交通进入快速恢复阶段。 3117. 上海市有关部门聚焦“快”“准”“管”，采取多举措，争取在第一时间控制疫情传播和扩散。 3118. 上海加油 3119. 坚持清零不动摇 3120. 本轮疫情发生以来，上海市毫不动摇坚持“动态清零”总方针，坚决遏制疫情扩散蔓延，确保人民群众生命安全和身体健康。 3121. 早日恢复正常，加油上海！ 3122. 确保人民群众生命安全和身体健康。 3123. 强调个人防护措施到位，确保阻断疫情的传播。 3124. 坚持“动态清零”总方针，坚决遏制疫情扩散蔓延，确保人民群众生命安全和身体健康。 3125. 上海加油！中国加油！ 3126. 疫情有效控制形势持续向好 3127. 希望早日痊愈，全面清零，复工复产 3128. 上海加油！ 3129. #上海加油#【6月1日起，上海有序恢复全市正常生产生活秩序】 3130. 共同抗疫，加油！ 3131. 加油 3132. 加油！ 3133. 祝上海快速走出阴影，稳住经济大盘。 3134. 大爱会被永远铭记，祝他们返程顺风！ 3135. 希望疫情能够早点控制，尽快复工复产 3136. 一切美好如期而至，如你所愿！ 3137. 我真想开学 3138. 不能上班太难了，尽早清零，恢复正常 3139. 加油，想回家了 3140. 希望疫情早点结束 3141. 早日清零 3142. #疫情退退退！ 3143. 恢复原有生活！愿疫情之后 3144. 这波…终于快熬过去了。好好过个端午和暑假吧 3145. 希望疫情能够早点控制，尽快复工复产，恢复原有生活！愿疫情之后，一切美好如期而至，如你所愿！ 3146. 希望疫情快点退散 3147. 坚持“动态清零”高效统筹疫情防控和经济社会发展 3148. 应该要解封了，两个月的抗疫终于告一段落。祝上海快速走出阴影，稳住经济大盘。 3149. 这就是中国，我们中国人好团结 3150. 感谢所有大白的付出，他们的大爱会被永远铭记，祝他们返程顺风！ 3151. 总有人在负重前行，感恩！ 3152. 不聚集、不扎堆，外出务必做好个人防护 3153. 生活还是要有仪式感的，这是美好生活的动力 3154. 仪式一定要有，这是灵魂 3155. 好消息~医护人员辛苦啦 3156. 大白辛苦了。感恩有你们 3157. 辛苦了，大白们 3158. 平安归来，上海加油 3159. 人要懂得感恩，所有人都辛苦了 3160. 祝贺关门大吉，感谢人民支持，医务人员辛苦啦，好好休息！愿你们一生平安！疫情过后，请来上海做客！！！ 3161. 各地援沪医疗队陆续离沪，上海社区传播风险得到有效遏制 3162. 我们生在红旗下，长在春风里 3163. 人间净土啊，全靠正确的政策和大白们的付出 3164. 这还是我们热爱的那片土体 3165. 不放弃希望是克敌制胜的法宝 3166. 我们的岁月静好是他人的负重前行 3167. 加强新时代爱国主义教育，需要把握好这一时代主题，增强“四个意识”、坚定“四个自信”、做到“两个维护” 3168. 爱国主义是中华儿女最自然、最朴素的情感，是中华民族精神的核心。弘扬爱国主义精神，必须把爱国主义教育作为永恒主题。 3169. 上海本轮疫情防控取得了阶段性的成效，当前上海市的疫情形势稳中向好 3170. 按照“有序放开、有限流动、有效管控、分类管理”的原则，实行分区分级差异化管控对防范区，严格实施“个人防护，严禁聚集”的防控要求 3171. 坚决遏制疫情蔓延 3172. 坚持终可以战胜疫情 3173. 上海继续加油，中国加油！ 3174. 为上海恢复生产 3175. #上海本轮疫情的首个方舱医院休舱#祝贺关门大吉，感谢福建人民支持，医务人员辛苦啦，好好休息！愿你们一生平安！疫情过后，请来上海做客！！！ 3176. 坚决遏制疫情扩散蔓延，确保人民群众生命安全和身体健康。加油 3177. 抗击疫情效果显著 3178. 市将进入第三阶段 3179. 加油加油加油 3180. 一切越来越好 3181. 确保人民群众生命安全和身体健康， 3182. 坚持“动态清零” 3183. 确保人民群众生命安全和身体健康 3184. 加油上海市毫不动摇坚持“动态清零 3185. 只要我们坚定信心，就一定能打赢这场防疫狙击战。 3186. 恢复正常秩序，防疫措施还得继续！ 3187. 全域清零，指日可待。 3188. 6月1日起，上海有序恢复全市正常生产生活秩序l 3189. 特殊日子，值得铭记 3190. 人民之期盼，太好了 3191. 云南人民发贺电 3192. 绝不躺平，不会放弃任何一个人 3193. 人民有信仰，国家有力量。 3194. 可喜可贺 3195. 上海有序恢复全市正常生产生活秩序，加油上海！ 3196. 中国加油，上海加油 3197. 好事好事，上海加油 3198. 没有一个春天不会到来，愿花枝春满，山河无恙，人间皆安! 3199. 疫情防控见成效 3200. 疫情防控，人人有责 3201. 疫情快速清零 3202. 上海加油！！ 3203. 中国加油，全域清零，指日可待 3204. 人民有信仰，国家有力量 3205. 感谢所有大白的付出 3206. 疫情防控清零 3207. 同心抗疫 3208. 中国加油 3209. 中国担当！ 3210. 共筑多重抗疫防线 3211. 我希望在下一次的时候，没有疫情 3212. 坚决打赢疫情防控攻坚战。 3213. 加油上海 3214. 坚决遏制疫情扩散蔓延 3215. 共同抗疫 3216. 打好疫情防控持久战 3217. 疫情防控见效果 3218. 做好疫情防控工作 3219. 祝上海快速走出阴影，稳住经济大盘。 3220. 大爱会被永远铭记，祝他们返程顺风！ 3221. 希望疫情能够早点控制，尽快复工复产 3222. 恢复原有生活！愿疫情之后 3223. 一切美好如期而至，如你所愿！ 3224. 我真想开学 3225. 不能上班太难了，尽早清零，恢复正常 3226. 加油，想回家了 3227. 希望疫情早点结束 3228. 早日清零 3229. 疫情退退退！ 3230. 希望疫情能够早点控制，尽快复工复产，恢复原有生活！愿疫情之后，一切美好如期而至，如你所愿！ 3231. 希望疫情快点退散 3232. 坚持“动态清零”高效统筹疫情防控和经济社会发展 3233. 应该要解封了，两个月的抗疫终于告一段落。祝上海快速走出阴影，稳住经济大盘。 3234. 这就是中国，我们中国人好团结 3235. 感谢所有大白的付出，他们的大爱会被永远铭记，祝他们返程顺风！ 3236. 总有人在负重前行，感恩！ 3237. 不聚集、不扎堆，外出务必做好个人防护 3238. 生活还是要有仪式感的，这是美好生活的动力 3239. 仪式一定要有，这是灵魂 3240. 好消息~ 3241. 大白辛苦了。感恩有你们 3242. 辛苦了，大白们 3243. 平安归来，上海加油 3244. 人要懂得感恩，所有人都辛苦了 3245. 祝贺关门大吉，感谢福建人民支持，医务人员辛苦啦，好好休息！愿你们一生平安！疫情过后，请来上海做客！！！ 3246. 各地援沪医疗队陆续离沪，上海社区传播风险得到有效遏制 3247. 疫情面前，我们无论来自何地、无论身处何方，都是休戚与共的命运共同体。 3248. 面对疫情，积极乐观的情绪、健康向上的心态，也是一种强大的免疫力。 3249. 中国人民一定能！中国一定行！ 3250. 坚定信心，同心同德，风雨无阻向前进。 3251. 同时间赛跑、与病魔较量，这是一场必须打赢的人民战争! 3252. 守望相助抗疫情，关心关爱见真情。 3253. 没有理由退缩，没有理由懈怠。 3254. 疫情终会过去，静候春暖花开。 3255. 与时间赛跑，同病毒较量。 3256. 病毒无情，人间有爱；勇毅笃定，战无不胜。 3257. 安全距离可以有，人间温暖不能无。 3258. 病毒可以被隔离，但温暖不会被隔离。 3259. 多难兴邦精诚志，同心同德显担当。 3260. 信心，是战胜病魔的锐利武器。 3261. 做到群策群力、群防群控，确保坚决打赢这场疫情防控狙击战。 3262. 经历疫情洗礼，我们开始反思人生，管他什么爱恨情仇，活着最重要。 3263. 不聚餐是为了以后还能吃饭，不串门是为了以后还有亲人。 3264. 政治优势，组织优势，群众优势，人民至上，生命至上。 3265. 关键时刻显担当，危急时刻见境界。 3266. 每个人做好力所能及的事，理性认识，认真防护，主动减少聚会扎堆，就是与前方“战士”比肩同行。 3267. #上海疫情防控##上海加油#【6月1日起，上海有序恢复全市正常生产生活秩序】 3268. #上海战疫##上海疫情防控##上海加油#【6月1日起，上海有序恢复全市正常生产生活秩序】 3269. #上海疫情防控##上海战疫##上海疫情防控##上海加油#【6月1日起，上海有序恢复全市正常生产生活秩序】 3270. 你在我生命中太多的感动 3271. 送给你小心心送你花一朵 3272. 不能上班太难了，尽早清零，恢复正常🙏 3273. 疫情退退退！🤺🤺🤺🤺 3274. 希望疫情早点结束😷 3275. 这波…终于快熬过去了。好好过个端午和暑假吧 3276. 感谢所有大白的付出，他们的大爱会被永远铭记 3277. 我真的好想出去旅游求求快点好吧 3278. 医护人员辛苦了！！ 3279. 中华民族抗击疫情迎来伟大胜利 3280. 转运老人网上一片骂声！不转运出问题了还是一片骂声！反正上海这次怎么都是被骂！以一个支援过疫区的人来讲，超过70岁的老人都应该被转运至方舱观察！一旦有问题从方舱到医院更快！至少至少方舱有监护设备！ 3281. 都养老院了，先想想自己为啥不孝吧 3282. 这个四月之声看到朋友圈有人发十几分钟就封掉了，想看看不到视频 3283. 没躺平都这样了，如果当时真的躺平，上海同胞岂不是要处于更为坎坷的境地。躺平派 3284. 不清零，奶奶起码能马上送医院，家人起码能见最后一面 3285. 新冠只是导火索，上海管理层才是炸弹。换任何一个需要抢救的急诊患者，要等2个小时的救护车！9条命都不够等吧？ 3286. 目前看对上海人这个omicron真的真的不是大号流感 3287. 博主节哀这操蛋的防疫政策 3288. 还是认为上海管理层用绑架百姓的方法来软抵抗中央动态清零的政策，表面上是更严格的隔离，而一切支持和支援隔离的方法和举措都人为设置障碍，比如抽空医院，拒收病人，阻碍京东等进城恢复供应，不当使用友省援助...从市政府到基层，干不了的赶紧让位，从中获利的干部会被清算的 3289. 博主节哀奶奶还是要多注意多保重 3290. 我外公昨天晚上也在上海去世了没能等到120没机会看病明明不是大病硬拖成这样我们全家守在养老院楼下不给上去连最后一面都没见到直接拉走火化没有追悼会解封后领骨灰如果防疫真的是为了保护老人那为什么现在为了防疫在草菅人命呢 3291. 问题不是清零是腐败的政府在草菅人命 3292. 看这条因为没能及时就诊老人去世的微博下还有这么多争论躺平还是清零的我就知道这个社会魔怔了楼主节哀这个倒退的时代太不幸了 3293. 帮转，宝贝节哀养老院真的求助无门，离我家不远的一家养老院，浦东新区市南养老院，附近的小区都能听到半夜老人的求救声 3294. 所以那个始作俑者还在论坛高谈阔论 3295. 上海市虹口区虹德养老院。节哀@上海发布@上海虹口 3296. 打过疫苗的话，就是死于基础疾病。没打过疫苗，就是死于新冠。 3297. 抱抱 3298. 120在卖货……O网页链接 3299. 出现大规模医疗挤兑，这都是不可避免的。唯一避免的方法就是初期控制，可惜上海没这么做啊 3300. 然后接下去都没有追悼会直接火化，别问我怎么知道的 3301. //@宝树:不排除一开始放水的可能，后面也不用太阴谋论，就是失控了神仙难救，有些黑心的想趁机发财是另一个问题//@蘑菇目前不改名啦:我真怀疑上海就是故意的，目的就是诱导更多人去攻击现有的防疫政策。毕竟三年了啊，三年了，是头猪都该知道一线城市正常的水平是啥样的啊//@敦煌郡公 3302. 电车难题：学欧美放任，受难五个人与政府无关，主动封控管理，受难的一人这与政府有关。前者可以说无可厚非，后者责任明确，民主议会制国家为了选票肯定选择前者。而主张“王无罪岁”的中国不一样 3303. 必须清零，不清零更多惨剧 3304. 唉奇葩操作看不懂节哀顺变O网页链接 3305. 节哀，照顾好奶奶 3306. 基础病不是推卸责任的理由！慢性病人，慢性病老人为什么就成为这次事件的牺牲品！太难过！难道只有年轻人才配在世间上吗？🙏🙏🙏 3307. 玩精准玩砸了 3308. 节哀 3309. 我有录屏 3310. 节哀，太伤心了。 3311. 博主请节哀 3312. 抱抱博主，节哀 3313. 真的搞笑了，我外公外婆两个人住在长兴岛，从从封城到现在，没人管过他们，没送过物资，做了一次核酸，没有问题后就没管过了，政府没有人了吗？ 3314. 博主节哀 3315. 节哀，奶奶保重肿瘤病人也很危险 3316. 疫情是美国的人类清除计划 3317. 随申办-互动-领导信箱-信访投诉养老院12345没用，养老院归民政局管，医院归卫健委管，我就是这么投诉的 3318. 配药我们都说买药 3319. 做试点我信，但是做之前不需要做好准备工作？？？把老人急诊病人都随便扔进垃圾桶就开始试点了？？？//@兴风作浪的大叔777:给全国人民做试点是上海这座城市的历史使命，你们现在可以骂日后心里会明白//@肥yaya的Freya:换任何一个需要抢救的急诊患者，要等2个小时的救护车！9条命都不够等吧？ 3320. 🙏南无阿弥陀佛！ 3321. 市长的责任最大不仅要撤职还要她赔偿上海的损失 3322. 博主节哀。 3323. 节哀，人在做，天在看。 3324. 这笔血债我看他们怎么还 3325. 要么就老老实实的清零，要么就躺平，上海以为自己很屌，结果全国最辣鸡 3326. 节哀🙏🙏🙏 3327. 真的是这样，根本没人管 3328. 真几把6啊都21世纪了，防控出了这么多乱子，好像还有人饿死，真是魔都 3329. 用软政策兑付zy 3330. 这图也夹？？？？？ 3331. 按最近的新冠死亡统计来看这个计入新冠死亡因为需要一些死亡病例来引起足够的重视但会强调高龄和基础病 3332. 图怎么又看不见 3333. 总之，都是错！//@狗狗156:转运老人网上一片骂声！不转运出问题了还是一片骂声！反正上海这次怎么都是被骂！以一个支援过疫区的人来讲，超过70岁的老人都应该被转运至方舱观察！一旦有问题从方舱到医院更快！至少至少方舱有监护设备！ 3334. 参考香港数据，躺平后养老院就是重灾区 3335. 老龄化严重，干掉领养老金的人 3336. @人民日报 3337. 博主节哀！另外！博主提出这些质疑是希望医疗救治流程相关机构管理更加合理高效，并没有质疑清零政策。清零政策和合理高效的病人转运救治并不冲突！别一上来就清零不清零的，清零不该以这些无辜的生命为代价，不是任何对现有流程的质疑都是在质疑清零政策本身！可真是刀没砍在你身上你不疼，善良点吧。 3338. 上海市政府有多少官员的子女可都在国外，美国躺平可以有免费特效药，所以上海市委受西方国家的影响特别大，上海人民特别有钱，为辉瑞贡献2300简直就是小case。我们西部地区的穷，买不起辉瑞，所以不敢躺平 3339. 有基础病的，得了新冠的，去世按基础病算，不按新冠算，为了控制新冠死亡数字。 3340. 深圳现在新增不多都要求72小时核酸，没有核酸哪里都去不了 3341. 抱抱你。 3342. 上海的官到底什么背景，这都没给他们换了 3343. 上海的朋友们还是认真的配合政府做好封闭防控吧。 3344. 把老人放养老院是怎么想的，钱多差口饭？确实是轻松不用照顾！ 3345. 如果全国到时候每天死几十万人，不知道bbc会如何报到 3346. 很担心养老院的亲人，我们尿片也快要断档了~每天张眼就京东美团~完全不敢想现在什么状况。 3347. 🙏🙏🙏 3348. 急诊等不到120，进不了医院。生活在这样的城市，要多绝望啊 3349. 还是那句话，疫情期间，有能力的都接回家养老吧。经历过深有体会。 3350. 评论区没有带脑子上网的吗？ 3351. 广州两周清零，上海整个管理层烂透了！ 3352. 真的是达尔文主义吗？ 3353. 不知道说什么，节哀 3354. 疫情都三年了、怎么还能乱过最初的武汉状况 3355. 节哀🙏 3356. //@鲁镇的柜台:我心目中中国最美的城市，加油//@直播上海: 3357. 烂透了，烂透了 3358. 我外婆98也是因为先阳了不给送医院最后在家咽下最后一口气，一个亲人都没见到 3359. 都是因新冠去世的，这些人那些人。 3360. 节哀，不知道情况这么严重 3361. 魔都真成魔鬼之都了 3362. 太难过了，博主你要小心，昨天又有临观的工勤阳了，你知道我说的是哪里的临观。 3363. 没进医院去世的都不算新冠死亡 3364. 大城市对于老年人养老不友好，好的中小医疗条件的城市才适合 3365. 真的难受了 3366. 这是回到文革了吗？ 3367. 直接不找中央有毛用哦 3368. 难过也要照顾好奶奶呀 3369. 投诉他们 3370. 我好难受啊 3371. 节哀，不知道应该说什么，只希望一切快点过去 3372. @今天也要喝冰阔落 3373. 心难受节哀愿您奶奶安好 3374. 告死他们 3375. 节哀。 3376. 真的看哭了 3377. 节哀。诶…好心疼 3378. 唉！🙏 3379. 抱抱博主 3380. 还要折磨多久 3381. 🙏🙏 3382. 🙏 3383. 节哀节哀节哪门子哀啊！？该追责的要追责，绝不可轻饶 3384. 无能的天朝 3385. 节哀，希望那些不负责任的人能受到惩罚 3386. 节哀，保重 3387. 节哀希望奶奶身体健康平安 3388. 🙏节哀也保护好自己 3389. 节哀顺变。 3390. 原博主节哀 3391. 抱抱你 3392. 我的天… 3393. 博主节哀… 3394. 这就是上海人选择躺平的后果 3395. 关键是上海这种还不算是新冠死亡，瞒着国家 3396. 都看不到图片的 3397. 节哀🕯️ 3398. 这是还没完全躺平医疗资源就挤兑的这么严重，共存了还不知是什么样了。 3399. 怎么好多被封了？ 3400. 哎...🙏 3401. 香港向中央请求支援医护的新闻你真的看不见吗？我们大广东派了上千的医护去香港，连张忠德都去了//@白羊座辰辰:回复@Paynard:香港医疗资源挤兑了吗？我看到的数据是他们感染数里8%的没接种人群，其中死亡16%。前段时间算了算他们的死亡率超过2%，不过我一看甲流死亡率超过7%，就不担心了 3402. 缴纳了医保，叫不到救护车他们违约了 3403. 其实很多人是不是把“动态清零”政策和目前上海的“防疫措施”混淆了，作为一个生活在上海的人，支持动态清零，但是目前的这种“防疫措施”尤其是前期的一刀切带来的次生伤害已经超越了病毒本身，很多需要看病求生的人，那可是一条条鲜活的生命就这么因为耽误治疗而逝去了！ 3404. @宣克炅@上海电视台案件聚焦@北京青年报@人民日报@新华社@北京晚报@澎湃新闻@上海发布 3405. 上海的医疗挤兑比武汉还厉害，武汉60岁的老人方舱都不收，直接进医院。//@狗狗156:转运老人网上一片骂声！不转运出问题了还是一片骂声！反正上海这次怎么都是被骂！以一个支援过疫区的人来讲，超过70岁的老人都应该被转运至方舱观察！一旦有问题从方舱到医院更快！至少至少方舱有监护设备！ 3406. @kx装睡了吗 3407. 血的教训，不能躺平，保佑家人平安🙏 3408. 虹口一塌糊涂，别来虹口 3409. 太多的老人在疫情期间离去，不能办追悼会，甚至有些没穿上寿衣，非常无奈。 3410. 四月之声没有办法被听到的人就此湮没 3411. 不知道怎么安慰你，抱抱 3412. 我们这边即使是很早之前就已转为低风险地区，但是依旧严控市外来员，甚至很多村镇都在劝回家参与丧事的人离开，不让入村。严防死守至此，我不能理解。 3413. 上海再不嘚瑟了，之前还一直嘲笑别的地方的防控措施，现在直接被点名了 3414. 21年，上海承接14137架次国际航班，占入境总量35.6%。今年前两个月1972架次占41.2%。一线、隔离酒店、公卫中心满负荷运转，这样的日子，上海过了三年，上海疏忽了、也累了，但我们坚信他会历劫归来，而所有失去的，都会以另一种方式归来 3415. 奥密克戎真的狡猾，潜伏期又长，为了自己还是老老实实核酸吧。 3416. 上海认为其他城市“过度防疫”“防疫一刀切”不对，作为国际化大都市要做个表率，要“精准防疫”“科学防疫”完全没问题，但不能以把风险转嫁给其他省市作为代价。其他省市老老实实“从严防疫”，多少轮全市核酸，好不容易稳定下来熬过来了。因为“精准防疫”的漏网之鱼，努力付诸东流，谁心态不崩？ 3417. 面对疫情防控，多些理解，少些埋怨，疫情防控需要每个人的配合。 3418. 这数据真实吗#上海六院# 3419. 没人想吐槽上海疫情，但是上海疫情外溢到外省是不争的事实。而现在上海公布的感染人数，让人觉得不符合常理，实实在在的合理质疑。希望上海和全国各地的疫情得到控制，大家越来越好。不要把自己架在那，搞得自己现在下不来台！ 3420. 上海的无症状感染是真多啊，都不是确诊吗？ 3421. 上海六院什么情况？没有一家大v出来发声？ 3422. 上海的确诊和无症状比例永远与其他省份与众不同 3423. 每天都看到奋战在一线的大白们和120，这个时候就听话，出门戴口罩，少去人多密集的场所。上海，会好起来的！ 3424. 精准防控的理念是没问题的，只是管理没有达到，提前嘚瑟打脸了。 3425. 无症状，全国就上海无症状多，怎么上海人民打的疫苗是上海政府研发的 3426. 上海六院到底有没有瞒报，护士到底有没有受委屈。请给人民个交代@上海发布 3427. 江西已经三例全关联上海，可想而知当地有多严重 3428. 说上海境外输入压力的，可以考虑一下深圳的感受，另外，第一入境点可不只是上海一个城市，除非有2021年全年入境排名数据，所以就不要拿这个洗了¡评论配图 3429. 看看六院救救妈妈#上海六院# 3430. 听到街上的救护车，真感觉这次感觉疫情真的很近，决不能掉以轻心啊！ 3431. 一直这样管控经济毁了，没钱纳税，而且很多人失业，公司关门，生意难做。所以说，慢慢还是要阶段性的疫情管控打开，对国外也打开，一直这样管控也不行。欧美早就全面打开恢复正常了。（不是亲美，只是理性去说，有些人不要听到建议疫情放开就一直喷，除非你家里几个亿） 3432. 上海六院呢？ 3433. 现在小区封锁程序太乱了，居民都跟惶恐 3434. 上海也从来没说过自己是模范生啊，嘲讽啥呢？HK的同胞们应该给其它地方平摊一点，上海也就没这么大压力了 3435. 这个数据是可以相信的嘛？？？ 3436. 无症状转的，也算意料之中 3437. 上海赶紧封城吧，全部人员禁止出门，居家隔离知道全部清零为止 3438. 惊雷哪去了？ 3439. 医生打人的事情说一下？ 3440. 这个数字包括六院了吗？他们怎么样了？ 3441. 上海新闻北京头条报道。。。 3442. 应该这样报道，新增本土阳性病例61例（其中确诊病例6例，无症状感染者55例。） 3443. 尼马当大家是傻子吗，海上玩什么文字游戏，有就是有，希望你们全员核酸检测 3444. 上海发布就是个笑话 3445. 上海：我不查就没有，你非要让我查就是无症状 3446. 在看发布会很喜欢吴凡每次都像妈妈一样虽然很能说但是说的有血有肉，苦口婆心，把事情说清楚，能感受到是真的希望大家一起努力早点好起来 3447. 疫情不可怕，瞒报才可怕，某些领导为了自己的官帽真是丧尽天良 3448. 疫苗接种率提上去，轻症无症状没必要占用医疗资源，自行在家隔离就好，恢复阴性就自由活动，最后还是会从动态清零走到共存这一步不信走着瞧，防不住的（奥密克戎传播率高，但毒性小，得新冠就像感冒一样而已了，别说什么后遗症，除非你没打疫苗）杠就是你对 3449. 看到六院的信息，请关注 3450. 关注一下这个事情吧O网页链接 3451. 看来六院的情况不能一直这样遮掩下去，如果是院内感染，将是严重的问题。 3452. 最不要脸的就是上海市，精准它奶奶个腿，不知道外溢多少个省市了，还天天牛逼哄哄的不行，呸！ 3453. 总结一下沪城蛆的抗疫表现1；2次拒诊孕妇，攻击孕妇没有去对医院。2；一码通崩溃，狡辩那不是一码通是云健康。3；外溢是好事，打破精准防疫神话，探寻新模式。4；赌运气赌赢了就是优等生，赌输了也是学霸只是这次没考好，外溢啦。5；控评，删评，上海yyds。6；今夜我们都是上海人。 3454. 这波来势汹汹，大家做好防护 3455. 直接说61例不就行了 3456. 我们这昨天就因为一个上海回来的，直接整个市都进入了紧急状态。上海应该自我反省，吉林省长都撸了，上海撸谁？ 3457. 这几天最大感受：当初真别骂武汉了，按那个时候的情形搁哪个地方都是烂或者更烂。没有净土。 3458. 要求跟香港全面封关，内地人的命也是命，生而为人凭什么香港人就要比内地人高人一等？香港自己不自救，而且还不接受内地的帮助，还歧视我们的医护和护工，甚至连杀猪匠都被歧视，这是要把整个中国都祸害了吗？你们这样做，经过内地的老百姓同意吗？香港是个什么东西，值得你们这么下跪？ 3459. 今天去做了核酸，防控从我做起 3460. 顶上去//@神之近卫军:21年，上海承接14137架次国际航班，占入境总量35.6%。今年前两个月1972架次占41.2%。一线、隔离酒店、公卫中心满负荷运转，这样的日子，上海过了三年，上海疏忽了、也累了，但我们坚信他会历劫归来，而所有失去的，都会以另一种方式归来 3461. 建议上海来次全民核酸 3462. 不要瞒报谢谢！ 3463. 但凡在上海的人就不会喷精准防控 3464. 朋友中招了一个学校确诊几粒都没报发烧26小时才有救护车来拉他到医院没有医护人员管他…他隔壁同事确诊了太吓人 3465. 不要再打肿脸充胖子了，捂不住了，赶紧好好做核酸吧 3466. 上海是精准防控还是精修防控？ 3467. 数据漂亮！不管你信不信，反正我是不信！ 3468. 这么漂亮的数字 3469. 定位徐汇靠近闵行周围真的蛮严重肉眼可见的比疫情第一年严重 3470. 坚持防疫，人人有责 3471. 所以为啥大学做核酸不让拍照，为啥不让社区发全员核酸字眼的通知，解释一下，没人会觉得推翻错的理论丢人，大难临头大家都是中国人 3472. 上海能不能居家办公啊？？起码一些可以居家的职业能不能让居家啊少点人口密度也好啊？！！！！ 3473. 上海孕妇流产，六院，上海红楼，上海地铁夹死人，疫情一直外溢，热搜撤的快当别人不知道呢？还给外地买热搜。钱是真多。 3474. 精准防控 3475. 大夫打护士的事不解释解释？？ 3476. 其实就是61，没必要分开的报的。上海加油啊，希望我们中国的每个省都能控制住，每个人都能平安！ 3477. 华政的到底什么情况，为什么还没通报 3478. 遏制源头，严防死守。早日清“零”，上海加油！！ 3479. 上海是不是独立啦？上海不是中国的吗有些人嘴巴干净点吧小心孽力回馈 3480. 心疼医护人员，我相信只要积极配合做核酸，就会好起来的 3481. @北京头条好大胆子，本来人上海捂得严严实实的 3482. 护士有没有被打？？！！？？？ 3483. 上海精准防控上海精准舆论防控上海微博会员大户 3484. 配合工作做好核酸，在家不乱跑，一线工作人员太辛苦了 3485. 想知道上海六院的具体情况，这个时候还瞒报要出大事的 3486. 我寻思这难道不是当时被吹精准防控，然后一帮沪吹和沪黑就开始吵，然后把zf驾到那个高度上去了出了问题又下不来。我就没见过我身边的上海人吹防控，我只想骂不给说话捂我的嘴，上海的领导什么时候能把骂的评论放上来，而不是只回复夸奖的话？有点本事行不行？别连累老老实实的上海人给你买单 3487. 我算是看明白了，哪个地方有疫情那个地方被骂🙉 3488. 这里没有控评，看到的都是真实的声音 3489. 为什么咱们总是学着千年不变的传统《装体面》，却总是埋没事实的真相！ 3490. 上海“精准防控”（怕不是“政治防控”吧）不要吹，低调一点。自己的城很放松，搞得别人的城负担很大。 3491. 建议以后别分有无症状了，有啥意思，无症状还不是会传染人。 3492. 求求别瞒报了 3493. 笑死每次都搞这种没满24小时偏少的数据上热搜，第二天完整数据是几倍，有意思吗？粉饰太平？ 3494. #上海新增本土确诊6例无症状55例#为啥无症状比确诊的多的多呢？ 3495. @超级多余余 3496. 还是这里的评论比较正常，其他很多媒体都精准控制舆论了 3497. 都这样了还不通知居家办公！ 3498. 希望新冠患者和其他患者早日康复！ 3499. 上海对于疫情的态度真的是下头。光说外地这不行那不行。这次西安疫情就是上海来的。还我们平安健康和自由。 3500. 只希望给医护人员的保护做到位，物资各种！ 3501. 远不止这点吧 3502. 加油🙏 3503. 作为社会人，自然要聚集，长期的隔离，生病态心理...... 3504. 上海防控疫情做的还是好的！ 3505. 江苏自媒体联盟超话 3506. 可怕 3507. 应该远远不止 3508. 上海加油鸭，共同抗疫，一起战胜疫情！ 3509. 上海加油！ 3510. Hk这些不孝子回来干嘛 3511. 俄乌战争之际，中国多地出现疫情？事出反常必有妖，很明显是有人在搞事。一些媒体唯恐天下不乱。......疫情是人为的，存在严重数据造假，无中生有。希望早日将那些搞事的人绳之以法，不能一直让它们兴风作浪！ 3512. 疫情是人为的，存在严重数据造假，无中生有！ 3513. 笑死，疫情严重开始比谁更烂，内撕了，好幽默的评论区！ 3514. 俄乌战争之际，中国多地出现疫情？事出反常必有妖，很明显是有人在搞事。一些媒体唯恐天下不乱。疫情是人为的，存在严重数据造假。希望早日将那些搞事的人绳之以法，不能一直让它们兴风作浪！ 3515. 谁都不想看到疫情，两年了，病毒在进化，防不胜防。希望大家都平平安安的。现在的网络上戾气太重了。 3516. 上海精准吹牛逼哪次没给北京送除了会舆论 3517. #上海新增本土确诊6例无症状55例# 3518. 严防。 3519. 强烈要求境外航班放到外地，上海真的累了，都是中国城市凭什么要上海承担 3520. 无症状感染多说明症状轻微。 3521. 这数据………真实吗？ 3522. 徐汇可以居家办公吗 3523. 脸呢？ 3524. 我们要知道真相 3525. 没有惊雷心好慌 3526. 不明白为什么哪个地方一有疫情就要被骂！长春人在上海，有疫情我也没慌过！境外飞机多少落在上海？上海前几次疫情总人数多少？多少天结束的？被隔离的人生活有没有得到保障？！只要有密接，第一时间找到，立刻全楼核酸！我之前广州出差，回上海后广州有疫情了，社区立刻打电话做登记，这效率要被骂？！ 3527. 前两年上海疫情控制还算不错，估计产生了麻痹思想，被病毒找到漏洞，这下玩了个大的，居然传到了大学校园，这下是不是该启动问责机制了，吉林广东官员够处理了一大堆，上海呢？ 3528. 病毒会变异，人性也会变异。 3529. 我有个想法：何不研发一种能重复使用的快筛试剂，发给每个家庭或者每一个人一只，每人每天自己在家快筛一次，阳性的就自己上报。 3530. 讲真，外地人真的要感谢上海帮你们挡住了疫情，而不是忘恩负义，攻击阿拉上海 3531. 上海春考面试怎么办春考也是高考啊几千学生的命运和时间在上海市教委眼里都不重要是吗 3532. 别的不说但是上海最早最自主人性化让宠物和主人一起隔离开始就比其他地方强，特别是你北京当互联网没记忆么 3533. 这破疫情什么时候是个头啊！赶紧结束吧！！ 3534. 一个用嘴防疫的城市，现在全国疫情爆发，必须追根溯源。 3535. 建议上海全民做核酸检测 3536. 上海疫情上了几个热搜，紧张ing，希望赶紧控制住。 3537. 真的有这功夫做好防疫不比啥强，成辈子就爱搞地域黑//@妖女YY:上海再不嘚瑟了，之前还一直嘲笑别的地方的防控措施，现在直接被点名了 3538. 上海，可以啊，管的不错 3539. 这都吵啥吵呀，这种时候要静心，少折腾，交给专业人士，管好自己，在家没事打打八段锦适当锻炼适当吃饭保持自身身心健康 3540. 一直删帖压热度当网友眼瞎是吗 3541. 上海加油！！ 3542. 感觉这样也不是办法，还是放开吧 3543. 哎 3544. 北京头条报道你北京吧啊，上海你管不着 3545. 我们没有逞能我们也不想逞能我们只是做了全国想做的事情防疫两年精准防控都是做的很好如果一刀切两年可想而知这次上海的疫情上海发布也承认了境外输入的防控管理疏忽我们勇于承担错误及时止损学校停课公司停业为现在站在防疫一线的医护人员警卫人员说一声你们辛苦了 3546. 经历疫情两年多了，医院起码有必要的储备物资来应对突发情况吧，而不是慌乱抓瞎，医护人员都不容易，疫情当前，首先保护好自己才能帮助更多的人。 3547. 上海的表现毋庸置疑，已经做得很好了，希望疫情早日结束！ 3548. 上海这回真的一团糟了，不知道咋精准的，精准到千家万户？ 3549. 隔离真难受，快点过去吧 3550. 我们国家突然的疫情爆发到底是咋引起的？ 3551. 上海最近辟谣多，无症状感染者多，确诊病例少。 3552. 好 3553. 路过 3554. 啊 3555. 上海精准防控，精准投放吧 3556. 防疫长备不懈！ 3557. 我感觉美国又给我们投毒了 3558. 自己注意比啥都强 3559. 为了一个逆子害了两个最强的城市群 3560. 南京的就是上海过来的不要杠，已经通报了 3561. 疫情啥时候才是头啊，真是揪心 3562. 评论真是诠释了什么叫孽力回馈 3563. 你多久 3564. 已经点不了“赞”了，太神奇了 3565. 下面由院领导发表下岗前的最后陈述。 3566. 把上海封了吧真的 3567. 战胜疫情 3568. 全国一盘棋，不要地域黑 3569. #上海六院疫情#，这是徐汇区有些人决策的问题，六院的医生护士已经很辛苦了。是谁决定的人口密度高的居民区附近的老旧宾馆可以作为隔离点的？因为不止华亭。田林宾馆也是老旧宾馆，宾馆后面后门连着田林三四村人口密度极高小区,做这样决策的人到底是谁？这到底是天灾还是人祸呀？不要转移问题的本质，O网页链接 3570. 如果单纯是感染对人体伤害不大的话，非常想知道现在的重症率和死亡率 3571. 加强防控 3572. 真相呢！ 3573. 好的 3574. 上海人民，在这高高在上没啥用，拿出真本事呀，做个防疫标兵呀，让全国人民也瞧一瞧么。光在这卖嘴有啥用呢 3575. 疫情防控守土有责人人有责！！ 3576. 全国任何地区有疫情都是我们不想看到的，为什么会有这么多人在这阴阳怪气的幸灾乐祸呢？素质可见。 3577. 评论真的是一塌糊涂，一有疫情就开始怪罪从去年西安疫情及冬奥会前后上海很多时候境外输入病例比整个广东都要多，最近一两个月大部分都是香港来的，但其他省市的人可不会这么讲逻辑，他们从不看上家是谁，溯源到底又是哪，一提到上海就喜欢集体高潮属实很无奈 3578. 6例怎么算的 3579. 防疫人员最辛苦 3580. 评论里一帮戆乱趁机地域黑天天盼着上海出事这下高潮了以后别说什么上海是全国人民的上海滚了远点 3581. 真奇葩。无症状不也是确诊感染吗？数据这么拆分有啥意思，自欺欺人？ 3582. 真累人啊 3583. 上海医疗资源充足，每天新增比杭州还少，没问题 3584. 每个地方情况不一样，人密集度，管理措施等情况也不同，大家还是冷静 3585. 好烦，这一生都要与病毒作斗争了…… 3586. 相信上海防疫！那些口出恶言的人真的是。。。。无语！！！ 3587. 挨着海外，依仗强大的流掉团队，以及强大的反应机制，就喜欢玩精准布控，玩大了，补救回来也没事，可怜的就是那些受难者，希望上海政府能脚踏实地点，别太骄傲 3588. 一出事就可以看到很多有心理病的人来指点江山，有时间还是多去关心吉林怡情吧，上海这点数量还不会垮掉 3589. 回复@布布曰:无症状那些应该是6Y的吧 3590. 给全国人民演示一下低成本高效率的精准防控 3591. 这确诊和无症状的比例不得不说还是上海🐮🍺 3592. 建议全国居家隔离，学生不上课，工人不上班，商人不营业，政府每日提供生活必须品，每月每人补助3000元，暂停一切房贷车贷，直到清零。 3593. 众所周知无症状不算确诊…… 3594. 今天有东川路800号吗？ 3595. 我信了 3596. 为什么别的几十条几百条的能上热搜，上海这么多还一次一次被刷下来 3597. 热搜一上就下去了 3598. 不明白上海现在难道还不算严重吗？为什么还不停止流动活动进行全民核酸筛查，就这样疫情怎么才是个头。天天在上海人心惶惶的 3599. 防疫还是看广州吧，都是埋头苦干，从来不敢说别人，被别人说也不会回应。结果只有自己知道，2例外市来的确诊，全民核酸了三次。感谢政府的默默努力，保障着我们！ 3600. 作为一个上海人，说句心里话，这次确实有关部门做得不够好，但作为一个居住在上海的民众，天天出门上班，路上看到的民众基本都是规范防护的，也许会在别人看不到的私密空间喘息一下也是无可厚非，但仍然请不要一棒子打死所有的上海人。上海地方不大，但人口密集，也是一线城市。。。 3601. 疫情不停工，为了gdp，脸都不要了 3602. 魔都这么牛逼，怎么上海人不开口了，当时喷别人的劲去哪了 3603. 上海个别领导的无能不足掩盖上海的整体成绩和能力，几倍香港人口，2者比较下，高下立判，相信疫情会很快控制，做到及时透明，有错必改，全国都会支持上海，保护好医生，这些都是我们的战士 3604. 南京就是上海输入的 3605. 上海碰到非常多骑着电瓶车还往后吐痰的，整个后面的人全都跑不掉！希望这种没素质的人早点回家呆着 3606. 常州今天确诊的从上海回来的。江苏省内其他几个城市确诊的也都上海回去的。不懂上海这么多天了都没全排查完而且每天才这几个， 3607. 老沪屁股摸不得 3608. 萧山机场你的福气来了哟，球球了，把境外航班境外人员都接过去呗，右边这位你们浙江的乡亲父老说不怕国外输入呢//@红社会组织:球球了，上海以后别装逼了。现在不怕国外输入，就怕上海不认真输出到省外啊 3609. 为什么压六院热搜 3610. 新上海人觉得上海做的以后很好。 3611. 上海加油，有些人不要地域黑，这些天医务工作者和防疫人员，到学生等老百姓都不容易，谁想这样的！动不动就上升到地域黑，有事就怪这怪那，同理心都没有，疫情当下，应该管好自身！国内已经很好了，不像国外疫情严重，还打仗！ 3612. //@舌头小舅妈:每天看微博都能看到一堆人在嘲上海什么精准防控了精准外溢精准投毒了不是优等生吗之类的话疫情那么久以来上海也许不是做的那么完美但是也不至于很差吧？那些一线防疫人员真的是很辛苦疫情当前没必要互相责怪大家都命运共同体我们的敌人是新冠而不是中国某个地区 3613. 删帖快哦 3614. 为什么评论要阴阳怪气的互相攻击？什么智商啊 3615. 一天天的，咱能不吵了吗 3616. 看了通报，感觉上海疫情有个特点，无症状感染者特别多。 3617. //@游天地寻龙鳞:回复@吴小呆www:要是北方随便一些城市。要被南方系媒体黑成翔了。//@像花泽香一样菜:上海六院什么情况？没有一家大v出来发声？ 3618. 精准控评 3619. 不真实的数据会让医护人员心寒，也会让百姓恐慌，希望这真的是真实数据！ 3620. 文字游戏，这些数字用得着这样？不要自欺欺人 3621. 求求了，封锁上海市吧！我们常州被霍霍了。。 3622. 消灭疫情是大家共同的目标，不关乎哪个城市哪个区域 3623. 指不定自己的城市就爆发疫情了。大家谁也别笑谁。谁也别骂谁。共同加油吧。（这b疫情真的累了） 3624. 西安人民谢谢你嫩 3625. 六院的都算进去了吗 3626. 搞不完了还！！！！！ 3627. 西安这次疫情就是上海航班过来的唉😮‍💨 3628. 这个数据里面感染者的溯源、轨迹、时间、请真实公布！ 3629. 黑社会化的人就要用黑社会的作风去治他们，反正后面如果我摸清真的是她幕后搞鬼的话，那我真的不会对她客气了。你也不需要心软，但是前提是要保证她不用坐牢，不是为了她，是为了你以后能出来。我是巴不得她牢底坐穿的 3630. 张文宏大师您怎么看？哦，现在是确认了不是临时工干的！ 3631. 你们这些评论有外国人逛微博的话可能给人家看乐了.. 3632. 我寻思不是让全国抄作业？朋友圈各种精准防控大v文章？怎么原来是驴粪蛋表面光啊 3633. 震惊😱 3634. 看了有位护士的老公发文如果是真的那简直太可怕了 3635. 全报出来了吗？（纯疑问） 3636. 为什么西安人跟上海人能吵起来啊，这又不是什么可以讽刺的点，之前骂西安的也不止上海的吧，想想去年那会上的热搜没几个好的肯定给人印象就不好0.0真是无语 3637. 还记得2020年造谣被抓的人吗？键盘侠们造谣之前想想清楚～ 3638. 有援助嘛？ 3639. 奥米克隆传播快，除非你永远封国别和地球上其他人来往，不然这是迟早是的事情，这么简单的事情为什么10亿网民就无法理解呢 3640. 就这次来看这大部分都没啥症状。有症状也大多数轻症。这比流感、感冒要好吧。流感也具备传染性。但是抵抗力好的人，在周围都感冒的情况下，就那一个始终坚挺着也不在少数。所以增强抵抗力。 3641. 任何人任何地方可以接受善意的提醒和建议，但不能忍受无理的谩骂和颐指气使的瞎指挥。都是同胞何必如此。这个时候有能力的可以提供专业知识、做志愿者，普通人就做好防护，不乱跑不添乱。这才是我们中国人在困难面前应该做的 3642. @茶世界-辉:福建疫情虽然减少增加，但昨天来看还是新增100例这样下去咋整 3643. 看来各地都放松疫情警戒了不然也不会一个省多点开花全国也是逐步开花呀 3644. 听说需要隔离风控的小区家里的宠物需要无害化处理是真的吗？？？🙏~ 3645. 漂亮数据，打脸充胖子没脑子。日本重要城市海关早就关闭境外输入。上海既然还能允许香港，东南亚，欧美疫情灾区进入。这是疫情泛滥的最大原因。境外输入病毒。 3646. 公子，啊哈，为什么，把我小护士打。医生，啊哈，为什么，穿防护服，不敢接触病人。护士啊哈，为什么，这么可怜，医生随意奴役。病人，啊哈，为什么，为什么？我要医生，偏只有护士。为什么，为什么？因为没有防护服。啊哈，为什么，医生，有防护服不敢来看我，护士，啊哈，可怜，没有防护服。 3647. 无语了😓评论里一些狗借着上海户籍又开始吠了 3648. 球球疫情快过去吧🙏🙏 3649. 明天封闭管理今天中午把文职赶回家办公，让一线员工直接在工厂住不要回去（这么热的天没有洗澡啥也没有）还想尽各种办法和zf去沟通开工要封闭式生产，还去搞个通行证可以往返什么三观简直了。疫情第一年也是，偷偷开工被举报了晚上开夜班！丧心病狂。 3650. 医生打护士这事怎么处理? 3651. 转发 3652. 无症状这么多，有什么办法，疫情太狡猾，不要遇到什么事情都落井下石 3653. 事实证明上海的防疫政策还是领先的尽管出现了点小问题相信上海政府的能力加油。 3654. 香港人都是大傻杯 3655. 玖富爆雷一年多来，一直不好好兑付出借人本息，一直暗箱操作，从未按要求披露资金兑付进展，不公布真实逾期率，不公布老赖借款人名单信息，不公布催收进度，不公布每月归集多少钱回款多少钱，不公布具体回款比例、人为制造紧张气氛，欺骗甚至恐吓出借人以极低折扣转让债权。 3656. 大上海也不过如此，贪污，腐败，欺下瞒上 3657. 说实话很难过上海瞒报疫情我们也受了很大罪如果要骂请骂那些瞒报的领导和那些素质极差的人，不是所有上海人都很排外那些自以为是的人多了去了要骂请直接骂人而不要带上上海上海不仅仅只有这些差的东西也有很多好的东西包括这些医生护士他们都在一线努力这关系到他们是哪里人嘛？ 3658. 这他妈也能地域黑，我真是笑了，微博还真是厕所 3659. 丶 3660. 这个时候还在吹精准防控的人真的智商捉急，从德尔塔开始就是物传人非常严重了，精准防控只能防人传人，上海这些当官的会不懂吗？继续拿精准防控欺骗无知百姓，为自己的玩忽职守开脱？全员核酸有多难，一千多万人的城市有不少都做了n轮全员核酸。上海就是当官的怕打脸呗！ 3661. 没必要吧都是一个国家，都是中国人，不要说一些让同胞伤心的话了，某些人看笑话呢，疫情这种天灾谁也不想的，大家戴好口罩注意防范，不要被某些别有用心的人当枪使就行了，团结一致才是炎黄子孙应该做的事 3662. 这都没上热搜，媒体一点风都没有。资本和权利的乐园。不愧是魔都。真魔幻现实主义 3663. 有些人就巴不得上海疫情防控不好，还是中国人吗？在阴暗的角落里键盘敲比谁都响。 3664. 赶紧好起来呀！！！ 3665. 上海人出门有好多囗罩都不戴，到处人群聚集，挖控不给力！ 3666. 癌症化疗不让去，数据是好看，小区全封闭。只有新冠是病吗 3667. 建议上海目前入境人员加以限制入境@健康上海12320@央视新闻@北京晚报@新知视频@封面新闻@国务院@国家卫健委@上海卫健委 3668. 评论给我看迷惑了，管好自己得了。 3669. 不止 3670. 我觉得在疫情防控期间，最重要的是全国人民一起互相加油、共克难关，而不是搞地区质疑，或者当网络喷子破坏团结 3671. 一直撤热搜，不就坐实了吗？ 3672. 上海六院到底什么情况啊 3673. 回复@凌老佛爷不吃素:酸吧。 3674. 6例？这是除了多少剩下的数字 3675. 哦？我还以为是西安呢（后仰 3676. 蹭一下热度，看看华东政法吧，要穷死了，今天校长打饭，如果吃不完的话，我压力很大（虽然我吃的挺多的，可能还不够） 3677. 三年了还在瞒报，政绩仕途就这么重要吗？ 3678. 看其他报道好像不止看看#上海六院疫情# 3679. 瘟疫最可怕的地方是得了瘟疫的人被病痛折磨死，没得瘟疫的人被恐惧失心疯。明明应该一起携手共渡难关的朋友同胞，却互相撕咬、彼此谩骂、底层互害。 3680. 主要上海的房子贵，怎么做都对 3681. 玩的一手好双标！当事人写的文章看了吗？上海发生这样的事难道不是管理混乱？难道没有瞒报情节？怎么西安当初就被骂混乱，无能，追责，到了东南沿海所谓强省市就是鼓励，加油！舆论都掌握在南方媒体手里，自己出事就各种隐瞒安慰和bbc,cnn一丘之貉 3682. 我就在上海，这个数据我都不信，隔壁小区就有确诊的 3683. 每个入境人员好像都是祖宗对他们太好了吧！全国十几亿人都要为他们买单。但他们是回来放毒的，两年多了病毒都变异了好几代了，隔离措施还是两年前的哪能不出事才怪呢？还在市中心，就像在人群中放了颗定时炸弹随时爆炸。 3684. 群众监督，舆论发酵，哪一个是压热搜能捂得住的？ 3685. 有这嘲讽的劲，为啥不去当志愿者，把劲都使出来转化成服务！全国抗疫一盘棋，都好才是好！ 3686. 现在全国各个医院领导基本都把抗疫当作捞功绩的好手段对于一线医护人员不管不顾 3687. 我真笑了大众合理质疑不行吗？防疫出纰漏了这不是事实吗？上海确实在发展中承担了很多，同时也不要忘记它本身也赚的盆满钵满，不应该将这一点拉出来作为疫情外溢、疫情爆发的托辞。我们不满的点从不在于人民，而在于政府作为。最后，南方系媒体控制舆论的手段真的很有一套 3688. 热搜没有了，厉害 3689. 说到底窝里斗的还是太闲了，事情都已经出了，还能怎样呢，政府自有安排，过好自己再说吧，要喷干嘛不喷美国 3690. 各位防疫人员很辛苦，我们人民群众很心疼。但是，这一次华亭宾馆管理疏漏，是哪些领导拍板叫香港同胞过来而没做好后续的隔离准备工作和监督工作的，我觉得这点需要给民众一个交代，华亭宾馆，锦江集团需要负责。商铺，小工厂的经济损失，怎么办？有的人举全部家当创业，政府又该怎么解决？ 3691. 上海市六院还是临港六院？ 3692. 不能因噎废食，也不应以偏概全，现在上海需要每个在沪人士共同努力、共渡难关，也需要不在沪的大家的精神支持。不要实现人生价值时要上海海纳百川，有事就骂上海装逼，上海也会累，也会疼，也会难过。 3693. 六院热搜下的挺快啊 3694. 我爸爸还在六院求求保佑 3695. 这次就好好接受网友的批评吧 3696. 上海六院，能不能别敷衍了 3697. 身为领导能不能发现问题及时想出方案解决问题，瞒报到底是处于一个什么心态，能不能将心比心，每个家庭都有老人跟小孩，第一时间发现的时候，紧急处理比什么都重要。每个地方都不缺缺心眼的领导，也不缺一心为民的领导，疫情真的是能照亮人心的一面镜子🪞 3698. 上海发布你人呢 3699. 热度降的真快 3700. 住在上海的🐶都有优越感 3701. 这是在撤热搜啊？？？？ 3702. 疫情当下，各地方由上到下都应该团结抗疫，而且一方有难，八方支援，咱们泱泱大国，别说八方支援了，十八方都能来支援。为何会敢瞒报，为何要瞒报，先是吉林某高校，现在是上海某医院，纸包不住火这么简单的道理都不懂的吗？团结一致抗疫，疫情终将过去，加油！ 3703. 精准泄疫的沪人 3704. 上海加油 3705. 唉 3706. 吉林呢？ 3707. 《精准防疫》《抄作业都不会抄吗？》 3708. 那上海最开始的又是哪里跑回来的呢，北上广深是中国的门户，境外输入的压力都顶了几年了，咋滴就活该啊，出了事，都在怪上海，是不对是房价对调一下就没有这些阴阳怪气的声音了啊？ 3709. 111 3710. 这是怎么啦， 3711. 噢 3712. 奥密克戎病毒传播力太强了，新冠病毒对所有人类社会都是挑战，这场攻防战要群策群力，打嘴仗就能赢吗？互相借鉴经验，有则改之，无则加勉，有问题就说问题，该落实责任就落实责任，我们后面的路还是摸着石头过河，现在互相攻击真没必要。 3713. 要做内部调查 3714. 疫情也要做好个人防护和配合 3715. 什么国家都这样管控了，疫情还是如此反反复复 3716. 医生隐瞒疫情，逼迫护士无防护工作，上海可真棒👍 3717. 哪里都不保险 3718. 注意防护 3719. 上海太差了，再也不去上海！！！！！ 3720. ！ 3721. 也就深圳狠，说封就封，是个狼人 3722. 3年了，疫情怎么就遏制不了 3723. 快用UVC病毒消杀技术 3724. 满了 3725. 咱就是说好好做防控，有些时候很难做到万无一失，咱就是管好自己，祈祷同胞们安稳度过，如何防控也不是我们网上敲键盘能做好的。 3726. 冲 3727. 都怕撤职，都怕担责 3728. 这也破防咯 3729. 愿太平无事 3730. 太难了 3731. 为了自己的仕途，领导不当人啊 3732. 妈的，上海招谁惹谁了？各地疫情严重的时候，上海支援哪一次不是冲在前面？全国各地，哪里没有得到过上海的支持？上海有疫情居然还被骂？也行啊，受过上海好处还骂上海的，祝你倒霉一百年。 3733. 啦啦啦 3734. 六远的医生竟然打护士，我也是醉了，我以为是家属打护士，结果自己人打自己人，我也是醉了 3735. 一定要防控好啊 3736. 疫情赶快过去吧，还我们一个正常的生活环境 3737. 防疫一盘散沙，各区各自为政，上海的没落看来不远了！ 3738. 开 3739. 笑死了评论里竟然还有阴谋论三个字几年过去了还论着那奏嘛呀 3740. 我就很纳闷了，外溢的城市每天确诊那么多，上海每天新增没几个，那么多无症状感染者？ 3741. 在中国官方处理问题的特色：隐瞒 3742. 谁再吹公立医院管理好啊？不也就这样？还打架呢？真是丢人！ 3743. 大家谁都别嘲讽谁，天道好轮回 3744. 浙江也深受其害，还特么的不承认 3745. 疫情应该很快会控制。但是六院的男医生竟然会打护士！这就不能忍了，他还是人吗？ 3746. 不夸大不隐瞒，做到一直很难吗？ 3747. 可以接香港的人过去 3748. 哎，可恨的疫情 3749. 整天不给市民做核酸，把疫情放给兄弟省市，不负责，什么精准防控，屁！ 3750. 上海为了保证给大家继续做ATM，在这样疫情环境下，宁愿停饭店都不给停工！动不动就停摆，我们也想啊，上面不愿意啊！再说了，你倒是接掉境外的飞机呀，别动不动就飞上海 3751. 让网红出来走几步…… 3752. d 3753. 我现在都还记得沪吹全网贬低其他城市防疫，吹嘘上海是精准防疫先进典范，并以上海不搞全员核酸检测为荣的模样 3754. 请问动态清零和清零的区别？ 3755. 上海疫情北京头条来报 3756. 烦死了 3757. 啥时候居家办公？ 3758. 哦 3759. 上海防疫已经信用破产，累计传到七八个省，每天只爆出几例确诊病例，周边城市纷纷对上海来的实施3+11新政，昆山直接封了去上海的路，可见真实情况……。天天吹精准防控，阴阳怪气踩别的城市，这次爆出这么多大漏洞，直到现在才要求出城48小时核酸，可见防疫责任感之差…… 3760. 为了祖国那一个逆子，所有兄弟姐们都遭殃 3761. 所谓的一陪一护针对的老百姓，里面的清洁工都是横的 3762. 又有了 3763. 数据好假 3764. @来去之间你可劲儿压热搜吧 3765. 晕 3766. 源头香港为什么还不管控 3767. 都是中国人，疫情难道是他国的吗？为什么会是嘲笑，表示不理解 3768. 呵呵 3769. 加油，上海！ 3770. 加油加油！ 3771. 持续关注疫情，愿国泰民安 3772. 西安被传染都不敢直说只能说省外还要被骂 3773. 还是北京头条有态度 3774. 某些上海人民真是玻璃心，谁闲的没事追着你们这种无名氏骂？不就是因为六院爆的一些事吗？真就娇生惯养只能夸上海好呗 3775. 愿早日好。 3776. 看看上海六院吧，不要装聋作哑了 3777. 所以六院怎么了 3778. 啊为什么不一样¡评论配图 3779. 为什么上海无症状这么多，确诊这么少？ 3780. @空纸又严重了什么时候能见面 3781. 没错 3782. 天呐这这这 3783. 说防疫做得好都怪上海外溢的城市？有的城市境外航班都没有凭什么骂上海，北京那么大的机场他们接多少，上海接多少。苏州防疫好，江苏一直封控快，那境外航班直接转弯去苏州吧，都是疫情的祸上海也在尽力 3784. 香港不封内地全封 3785. 不是笑上海，是说上海六院，不严查说不过去 3786. 上海？不是吹精准防控中国第一吗？翻车了这是 3787. 疫情快过去 3788. 哈 3789. 我感觉绝对不止这么少人，这么大一个城市，人流量这么复杂。怎么可能只有这么点呢？我没有诽谤的意思，只是怀疑。 3790. 上海装逼，要不然也不会主动接收香港的同胞，接收倒没问题，酒店消毒不到位 3791. 大家注意，踩和捧有很大一部分是同一批人 3792. 说一千，道一万上海疫情就是香港宗桑害的！就是香港宗桑把病毒带来上海的！香港宗桑滚回去！ 3793. 什么红楼地铁夹人孕妇六院，到底咋回事啊，一觉睡醒就这么多词 3794. 奇了个大怪上海什么时候吹自己精准防疫，踩别的城市了？而且上海这次是因为华亭出了问题，华亭住了什么人不知道吗🤷🏻‍♀️怎么搞的上海恶意传播病毒一样如果没华亭那帮人上海今天也不会出事好吧 3795. 怎么又严重了，疫情快点结束吧 3796. 请查明上海六院疫情真实情况，保护好一线工作的医生护士们！ 3797. 加油中国 3798. 这不是传染病啊 3799. 国外输入不断！国内疫情就会断断续续的出现！ 3800. 关注了 3801. 中国🇨🇳加油💪 3802. 祈祷上海平安 3803. 🙏🙏🙏🙏🙏🙏上海快快好起来呀 3804. 早说了，抓起把沙子，手指送一送，漏下的沙子总得有人接着 3805. 确定没有人投毒吗？ 3806. 66666666666666666666666666666666666666666666 3807. 每个人都要做好防护 3808. 啥时候是个头啊 3809. 精准防疫原来是精准瞒报。 3810. 人家北京广州深圳也没得瑟过。就上海是说不得娘娘 3811. 怕 3812. 疫情早日结束 3813. 上海加油💪 3814. 中国加油 3815. 。。。 3816. 真的还有蠢人不知道这次上海是为了救香港才沦陷的吗？ 3817. 上海阴间新闻是真的多，上海孕妇，上海地铁，上海监管不力上海政治好差，人民好辛苦 3818. 只求平安 3819. 防控 3820. 上海疫情 3821. 大家不要理那些在评论区的阴阳怪气的人。这些人没安好心，在这里故意引战，挑拨是非，分裂祖国。不要让他们得逞 3822. 不要被黑子带了节奏，所有城市的疫情都希望尽快控制。最近反中势力肯定会浑水摸鱼，希望无论哪个地方的人民都不要攻击自己的同胞 3823. 上海精准防疫就是最大的笑话封了一个奶茶店一片叫好但是背后却是管控不力导致疫情外溢出了问题就是瞒报粉饰太平 3824. 了解 3825. 来自大城市的“危险” 3826. 藏着掖着只能消费我党的公信力，要赢得群众的信任才能战胜任任何困难 3827. 压力山大，更须加油。 3828. 从没看见过无症状报道是怎么回事 3829. 就这还瞧不起外地人呢，本地人就先倒下！ 3830. 加油↖(^ω^)↗ 3831. 🙏加油 3832. 我家小区被三个上海来的祸害了，又突然紧急核酸。 3833. Bbb 3834. 现在看来网传都是真的，说不停课，结果两天后就停课了，说不封城，结果现在变相封城，还有哪些网传？ 3835. 不能 3836. 精准 3837. 可怕上海真可怕 3838. 加油！加油 3839. 国家抗疫一直不容易，上海这次就是某些人太自信了导致的，那些还在装x的说上海现在还是很松的，这种人怕真的🧠不正常要不是我也在上海，我真的信了 3840. 沪舒宝最让人反感的就是我网暴你是为了你们好，你们网暴我，就是你们的不对！ 3841. 真搞笑，素质见高低，有些人真的唯恐天下不乱 3842. HK来沪航班每天几十例确诊但却可以不熔断，谁拍板的？ 3843. 👌 3844. 吗 3845. 2022都不容易 3846. 精准防疫真准 3847. 怎么会这样，啥时候是个头 3848. ！！！ 3849. 6 3850. 西安几次疫情都和上海有关，这次也不例外。 3851. 大家别骂了，希望全国都快点好起来。唉，可太烦了。 3852. vv 3853. hh 3854. 我妹妹在上海，这下不知道什么时候才能回来，希望这波疫情早点结束吧，没完没了的 3855. 是 3856. 悲剧 3857. 挺好的，上海本来就超负荷了，入境航班赶紧分散到其他城市吧，比如防空很牛的西安 3858. 你永远可以相信上海 3859. 疫情防控要严防死守！ 3860. 早日安好 3861. 加油吧 3862. 坚持党的领导。 3863. 愿世界无灾无难 3864. 真厉害，这么快榜单上就没有了，咱家微博真厉害 3865. 弄不好了，没完了，开学不开哪哪不能去，两年多了，搞死了 3866. 这就是疫情一直拖了三年的原因！一直在遮掩，全国有很多像上海六院这样，只不过这次事态严重实在瞒不住了！ 3867. 什么 3868. 看看 3869. 上海还有民乐幼儿园感染了多名儿童呢 3870. 陈j和滚滚你呢 3871. 问一句，无症状感染者和确诊的区别？ 3872. 曱甴真多 3873. 这次香港祸害了不少地方啊！你们就惯着这个反骨仔吧！ 3874. 怎么没有人嘲笑上海呢？ 3875. 发布会有什么好看的呢，让我们老百姓花时间看他们撒谎。 3876. 上海继续吹继续吹 3877. 我们可以永远相信上海，我在武汉，我是抗疫志愿者，上海有脚踏实地的官，爱心满满的民，扎实的专家（张文宏为代表），为上海加油：以最小的代价，争取抗疫胜利 3878. 昨天登记个上海回来的，我问他疫苗打了几针了，他说没打，我说为什么不打，他说不想打。。感觉上海确实心态好。。。可我心态不好啊 3879. 病毒厉害 3880. 现在为什么这么多的无症状啊 3881. 不管怎么说，加油吧 3882. 你们微博这些人真的好闲，除了冷嘲热讽当键盘侠对国家一点用都没有 3883. 一向以防疫措施和应急处理反应最快为傲的上海，竟然以这种方式上热搜，希望尽快说明情况给大家一个安心 3884. 西安比上海做得好太多了！西安迅速封城，没有造成像上海这么严重的外泄，更没有吹牛皮说自己精准防疫！23-28轮核酸1566万人！上海呢？牛皮都吹爆照样孕妇拒诊，孕妇流产、小红楼、地铁夹死人、宾馆医院大感染然后控评撤热搜！至少西安的信息是公开透明的！西安接受所有人的监督和批评！ 3885. 上海怎么还不要求居家办公？ 3886. 做好自我防护 3887. 好吧 3888. 作为中国人大家这么落井下石真的好吗可以吐槽当地政府防疫不当为什么要吐槽这个城市上海一直作为标杆压力也很大航空熔断也不是上海人说了算的中国作为大国一直在包容国外疫情上海作为境外输入点也在不断接受新的挑战中国几个城市接收境外输入掰指头也数得出这种时候别再阴阳怪气了 3889. 六院六院六院 3890. 丨 3891. 大上海快点清零 3892. 已阅 3893. 加油祖国！ 3894. 打护士的医生傅一牧，本科毕业进上海三甲医院工作6年不断进央视纪录片澎湃新闻​……啧啧啧，好奇家庭背景 3895. 严格管控 3896. 怎么突然就多了 3897. 无奈 3898. 基本都是无症状了，病毒威力减轻了？ 3899. 上海真的是个装逼王。赶紧好好管管这届各路神仙领导吧。自己没做好还找顶锅的。这是高高在上的上海人的做法吗？ 3900. 上海人的排外在微博上算是体现了，一句让不得外人说 3901. 可以强制办公嘛，公司让我们带核酸上班，公交地铁停了好了，大家减少流动！ 3902. 心累 3903. 上海--干啥啥不行，甩锅黑别人第一名 3904. 首先先学会别瞒报吧！ 3905. 网络就是键盘侠战斗的地方 3906. 🙏🙏🙏🙏 3907. 这我是很不能理解上海嘲笑浙江全民检测？还有嘲笑北京？然后现在说话还要带西安这两字，是自己不行吗？人家怎么不说别的地方就说上海呢？也是要自身找找原因。还有说西安自己给自己买热搜停课和飞机我们很闲吗？在我们来说不过是正常的事情而已根本就没有必要还是先把你们防护措施做好再说 3908. 遇到问题撤热搜，遇到困难就瞒报。看来哪里都一样 3909. 为什么这么说上海 3910. 早点结束 3911. 上海真的没必要去喷，要不是上海愿意接国际航班，国外留学和务工人员回国就更加艰难，而且疫情本身就防不胜防，很难做到清零 3912. 、 3913. 疫情二年了，管理还这么差 3914. 我周围的上海市都在吐槽瞒报确实 3915. 小心，1450及境外组织挑唆制造内陆地区间敌对心态，时局动荡一致对外，搁置争议。少些抱怨，那样没有任何用处，共同努力为国家巨轮增添一份动力能源，再小也是一份力。各自做好自己该做的事，赚该赚的钱。有大家，小家才会更幸福。 3916. 现在已经开始拼“无症状”了吗？不确诊不担责？ 3917. 打脸总是太快。之前在网上高呼“抄上海作业，看怎么精准防控”的那一波人去哪里了？ 3918. 早日战胜 3919. 在有些人眼里，“上海”两个字就是原罪。不知道是谁嘲笑了外地，确定是上海人吗？如果是，那个上海人可以代表我们所有人吗？这次的疫情不管是天灾还是人祸，总之都是上海的错。 3920. 这次好几省疫情又是上海输入，长点心吧，谎言会戳破的，非得要到为全国人民低头认错的时候，搞实验田利己主义折腾外省 3921. 差 3922. 境外输入不是闭环管理吗？之前还有人科普境外的都是统一隔离核酸检测，是不会传播到社会面的，那这次是哪个环节出了问题啊？ 3923. 就觉得如果是真的为什么要隐瞒下来呀，我们又不是“外人”，不怕你生什么病，只怕你有“病”不说，为什么呀，我们不是一家人，不能一起解决吗？ 3924. 不是护着上海，咱也不是上海人，防疫这一块做的真没得挑 3925. 不要再给外省输出了，几个省就被你们这种装b的政策霍霍了，还在吹牛逼 3926. 上海加油！中国加油！我们一起扛~ 3927. 上海之前因为精准防控麻痹大意了，导致疫情外溢江苏陕西等省份，好好的认错不行吗？非得找回面子？什么境外航班多接的人多隔离任务重。那广州，北京，深圳，四川等等地方也有境外航班啊，广州白云国际机场还是一度成为全球第一多航班的机场呢，深圳每天都要接几百个香港过境的呢，那是不是要吹破天？ 3928. #上海六院疫情##上海六院疫情##上海疫情##上海交大疫情#城市一换，评论过千万（西安：又来黑我？） 3929. 骗子 3930. 我不理解你批评一个人批判一件事都很合理但有些人否定一个城市是想干嘛？想搞分裂吗？ 3931. @央视财经@光明日报@人民日报@人民网@央视新闻@央视网可以调查一下关于#上海市第六人民医院#这个事情，如果是真的，可以管控吗？ 3932. 瞒报太多了我们小区也确诊了附近的也封了很多天，到现在都没有黄浦 3933. 上海什么时候嘲笑过别人了，一些骂上海的是受过什么挫折了？这么多疫情城市就怼着上海骂是因为上海是明星城市吗😊😊😊可以理解，明星都是要被人说三道四的 3934. 魔都魔鬼多。 3935. 也就是今天一共新增61 3936. 吵勾8⃣️吵了疫情能好吗？不管南方北方都有自己做的好与不好的地方嘴下积德一起度过难关不好吗？被微博搞地域黑的人带着跑冷静点吗？ 3937. 表彰都领导受着，这时候倒没影了，推一帮护士上，给真正的好医生丢脸！这么多护士和病人家属的命不是命吗？！危害这么多生命免职都不足以解决！打人的医生和杀人的未遂的区别在哪里？！ 3938. / 3939. 我觉得喷这次上海防疫失控也能接受，确实做得不好，但我怎么也想不通做了两天多志愿者，总共没睡几个钟头，临睡了还要被嘲笑，被嘲笑到睡不着。敲键盘地域黑的人哪里都有，但是精准防控的概念本身是没错的吧？对老百姓影响尽量小点不好吗？ 3940. 无语 3941. 此病毒不是上海制造，上海承担了大部分的境外帮那些没能力承担的省市包揽了业务好来人无完人一旦出事那些没有能力一直躲在背后外省人心里千百个恨啊你怎么没守住门你怎么还传给我们了你是怎么做的你们上海太没能力了你知道吗就是因为你们的过失让我们不能过像以前躲在你背后的安稳日子了 3942. sh？卧槽 3943. 上海控制的挺好的，希望继续加强严控，加油！ 3944. 看不下去了，我就问一件没有这些一线工作人员，谁去检测核酸，防控新冠，医治病人，是你们穿着防护服的领导吗？！ 3945. 为了保证精准防疫，脸都不要了 3946. 大上海人的优越感从来没有缺过 3947. 内讧有意思吗？心理太阴暗！ 3948. 就说外溢了这么多个地方，而且我相信不止我们这儿在重点排查有上海旅居史的人了。就这样还压。前两天官方通报还说就几个新增，你们这一波节奏着实让人民不相信zf了。🙈 3949. 这次上海的无症状太多了吧…… 3950. 总计不就是61例吗，为啥非要把无症状分开说，显得好看嘛。。。。。 3951. 为啥全国只有上海无症状比例这么高呀 3952. 疫情这么严重，各位键盘侠除了会地域黑，又能为疫情做点什么呢 3953. 说的我很赞成，但是话说回来主要还是前面沪吹把上海吹上天了把别的城市骂成狗屎了，所以现在算是反噬，过了就好了 3954. 我一直有问题为什么到现在所有的国际航班还是不给停，现在深圳东莞全部封闭造成的损失比国际航班停运损失大的多得多吧，国际航班关三月，会少什么嘛，因私的国外的别回来，国内的别出去不行吗。。。 3955. 咋说呢，反正各地都有疫情了，摆烂呗，都待家里。 3956. 总感觉这样的政策下去，国内会开始内讧啊这是国外最希望看到的大家要清醒啊唉 3957. 我党好好想想失去群众、让群众寒心的结果是什么就该明白现在到底该怎么做了吧！ 3958. 山东这么严重都没上热搜 3959. 这里评论还没被删 3960. 这次公关做得不好，热搜榜都好几个小时了，差评 3961. 都是中国的城市，干嘛互相说不好啊，大家一起相互扶持就可以了呀，现在上海这么严重，应该关心而不是落井下石。 3962. 真的只有55例么？ 3963. 上海某些小赤佬：没把控好不准你们说，六院的事不是上海人，医院也不是上海的，用的只是上海的第，就是不准你们喷的意思，金贵的很 3964. 这倒是该说一下，毕竟这个疫情源头是万恶的那啥弄的。咱们这个时候互喷没有意义。还是希望大家能共渡难关。这个时候都不好受。咱们这儿2年多的无新增被上海这波外溢给整破防了。而且，我们也给足了上海面子，报到都是写省外输入，而没有指明是上海。青春那么短，疫情就三年。三年了啥时候是个头啊。 3965. 上海人是真的不行 3966. 捂不住了吧 3967. 积分系统三公 3968. 护佑平安 3969. 阳性病例是在临港院区发现的吗？ 3970. 法律法规现在明确规定，地域黑网暴都会被被查ip和行政拘留👋，绩点口德吧评论区无脑喷上海的 3971. 行了吧…………都是自己人，都是中国人，本来境外输入就防不胜防的，我们不是更应该团结一条心吗，再说现在变异株体本来就很狡猾，干嘛非要骂来骂去的，有意思吗……… 3972. 丧嗨宁说不得 3973. 虚报瞒报 3974. 这两天某书上的上海街拍几乎就没有看到戴口罩的…… 3975. 66 3976. 上海人biss 3977. 数字对吗？我同事周一碰到确诊的，周五才通知他隔离？4天老早跑好多地方了 3978. 没有指责的意思，但是这一次我们这里两年多的无新增被上海整破防了。不管怎样，都是同胞，都希望全国各地的疫情都快点结束。 3979. 我看大街上没一个戴口罩的 3980. l 3981. 上海学学深圳吧 3982. 上海加油！！！ 3983. 希望疫情快点好🙏 3984. 阴阳怪气讲话的人到底是在看上海笑话还是在帮助上海人反思，动机是什么？敌我不分，帮西方国家拆自家团结，唉 3985. 三年的疫情了还是这种处理方式只能说一直原地踏步或者说又要五年一波了 3986. 不是太明白确诊和无症状感染者的区别 3987. 疫情防控是大家的事，请不要网络暴力，一味埋怨是没用的。我怀疑那些骂的人都是间谍吧被收买了，来煽风点火来了，想让中国社会动荡。 3988. 怎么上不去呢？ 3989. 看完吉林林的再看完上海的我觉得还行啊上海那么大 3990. 听说上海是因为接收乡港人，所以爆发了，是不是真的？ 3991. 不可信 3992. 无语子 3993. 下台把 3994. 3.3那天在6院奔波的我有点害怕。 3995. 上海承担的已经太多太不容易了，此刻只希望赶紧太平 3996. 上海～一个鄙视全员核算，只要不测就没有的城市 3997. 看了上海那个什么电子厂的视频，感觉没有我们广州安逸哦，之前感觉大城市防控有力，希望这次不要让大家失望哦，要是一直爆爆爆群体免疫可能在路上了 3998. 希望疫情赶紧过去!!!上海挺住!!!还有就是别压自己个儿热搜了，也别买别的地方的热搜了!!大家心里都很清楚的啊 3999. 身在上海从没受到疫情影响，为上海的精准防控点赞👍也相信上海很快走出这次疫情。 4000. #上海六院情况说明# 4001. 唉热搜一半和疫情有关不是这发现就是那听课 4002. 兄弟，你这数据太假了，都已经都曝出来了。你这样消费政府的公信力。真的没道理的 4003. 目前被隔离在上海松江某小区，第三天了也没有任何物资，饿的不行。关键也不说啥时候解封，居委会也不发公告，就说等通知，向单位请假都不认可，对上海防疫彻底失望了。 4004. 过一会江苏各个市喜提热搜嗷 4005. 认真 4006. 无症状也是确诊病例吧，能不能别说的这么含蓄其他城市很多被隔的人，确诊后也都是无症状者，都是一视同仁啊，为什么上海要这么报？ 4007. 六院真丢上海人的脸！！ 4008. 人类对抗病毒哪能做到精准，非要搞政绩做宣传，拿市民的健康开玩笑，这场战役就是宁可错杀一百不能放过一个，拿经济换健康，当初二三十平米的地方，好好消毒几个小时都能给病毒冲几个澡了，非要封几天拍点照片做点宣传精准防控，真搞笑 4009. 我就好奇，这个数据通报有第三方监督不 4010. 这精准地区防控远没有网络舆情做的好。 4011. 疫情期间去了几趟上海公共交通畅通无阻地铁火车站没人检查基本的绿码行程码。今天疫情的反复一点不惊讶。 4012. 说实话，现在真的没必要清零了，因为估计这个病毒会伴随人类好几年，应该想想其他方法，现在天天让人排队做核酸有点矫枉过正的感觉。 4013. 哟！ 4014. 上海金山检测异常隔离点已经满员了，里面都是检测异常但没有确诊的病人，他们缺少足够的医生，希望这批未确诊的病人能得到国家有关部门的帮助 4015. 看了看言论，喷上海的或说被上海喷的，微博都没发言地域显示的，所以到底是哪里人说的话谁知道呢，干脆强制要求微博显示发言区域就太平了。 4016. 看了评论区，围西救上？ 4017. 等什么时候你们的谩骂、诋毁能转变成为对疫情有益的行动，我想中国的思想、素质教育应该不低于其他强国了 4018. 评论里的上海人真是让我开了眼界了 4019. 天津人搬着板凳坐这看。 4020. 上海做的不好还不让人说了 4021. 首先发现有阳性患者应该立马转移到专业医院去，该封院封院，该隔离的隔离，所有省份都是这样的，没有什么好瞒的，若是自己医院有隔离病房那就有专业隔离病房工作的医护人员，让一些没有培训的人直接上（重点还是密接）我不理解这个医院的作为，太不把自己的护士当人了吧，看到这里我真的是很气愤 4022. 防不胜防 4023. 评论区真是…都2022年了还有人在yygq地酸上海啊格局大点吧 4024. 上海的疫情什么情况我就这么说吧第一次看见家附近的小区封掉了我家在去地铁站一定要做公交车的乡下 4025. 数据是不是要捂不住了 4026. k 4027. 上海六院上海六院上海六院上海六院别的不想说，就几分钟全删评了，再过几分钟热搜都降没了。 4028. 致敬医务工作者 4029. 真心搞不懂你们现在这些人，你们能算是人吗，一个省爆发，你们就互相喷互相嘲笑，几万条人命在你们看来很好笑吗，国难当头不仅不互相加油鼓励，一天到晚互喷，真不想骂你们畜牲 4030. 加油啊 4031. 评论里好多键盘侠 4032. 这个上海政府和香港政府有得一拼 4033. 希望六院快点好起来，昨天做着核酸，今天就关门了🙊🙊🙊 4034. 上海一直疫情严重，就是不知咋地那么安静一整年都有疫情 4035. 我 4036. 上海宣传系统我是服气的 4037. 大家要团结一致去抗疫，上海加油，中国加油 4038. 为什么突然就遍地开花了？ 4039. 全国都疫情爆发，希望疫情快点过去 4040. 热搜继续啊，别压 4041. 期待一个真实有效的回应 4042. 上海六院上热搜了！ 4043. 防疫魔幻事件：1、所有去过风险地的人都要去社区居委会报备，你来我往欢聚一堂2、黄码红码人群不能去任何公共场所，却可以自由出入社区居委会还是防控办要求的3、所有确诊病例的行迹无一例外都没有提大社区居委会，是他们都没去过报备？还是故意不说？这些到底是精准还是懒政？ 4044. 仔细看看?数据和实际真的匹配么? 4045. 上海六院领导层能判刑吗 4046. 六院领导咋说不是传染病哦？ 4047. @-明明哥-：好好查查6院吧，一个医生竟然说新冠不是传染病，自己穿的防护服不上楼，让只带了口罩的护士照顾阳性病人，这就是精准防控的上海 4048. 好好查查6院吧，一个医生竟然说新冠不是传染病，自己穿的防护服不上楼，让只带了口罩的护士照顾阳性病人，这就是精准防控的上海 4049. 西安疫情就是上海传来的。哎！刚消停一个月过了年，又来 4050. 一个区失守，一个医院又失守了，混乱堪比武汉疫情初期。全国防疫，本就没有神话 4051. 开始降热搜了 4052. 上海是全国人民的上海，这次的疫情从全国的传播速度来看都是非常迅速的，大家都有些掉以轻心，上海已经在积极应对，没必要搞地域针对，你在说风凉话的同时，想想如果经济重地的崩溃，对国内其他地区的形势都是不利的，如果想自己城市好，就盼着上海早点控制局势吧 4053. 上海加油啊，控制住，疫情当下人员流动好麻烦 4054. 相信上海，在广州为上海加油 4055. 不是精准防控吗精准到哪里啦 4056. 我就不明白疫情报备为啥一定要去社区居委会不能在网络平台？人人都去社区居委会这不是更大的交叉感染风险吗？最魔幻的是黄码红码人群不能去任何公共场所甚至医院都不能进，去可以自由出入社区居委会还是防控部门要求的，这到底是精准防控还是只是防控懒政？魔幻魔幻 4057. 看评论，真是搞笑死，说什么小浙江，什么别的地方犄角旮旯……要上天么这是，个别上海人真是这嘴吐不出象牙，别给自己抹黑了 4058. 321 4059. 本人判断疫情估计与春节长假有关我所在的公司节后有大量的员工感冒姑且不论是普通流感还是新冠希望科学家能早日开发出抗新冠特效药把新冠当感冒治从而结束疫情 4060. 其实无症状也是阳性，也是具有传染性的啊，这样区别报道，有什么用呢 4061. 其实百姓心里早就有定数了，冬奥开完，疫情猛增，只不过没想道，增的这么严重 4062. 领导：听我说，这不是传染病！而自己全身上下装备齐全！可笑！ 4063. 我不装还不行嘛！我撂挑子了，你们这13个城市自己玩去吧¡查看图片//@请结合临床及其他检查:你是不怕境外输入毕竟境外的飞机都往上海落//@红社会组织:球球了，上海以后别装逼了。现在不怕国外输入，就怕上海不认真输出到省外啊 4064. 六院的具体情况什么时候能披露！！！医生打护士！！院领导穿着防护服告诉护士这不是传染病！！这些事到底是真是假，能不能解释清楚！！！ 4065. #上海六院疫情#这个时候还想拿人家西安给自己顶雷，贱不贱啊，真下作真小人！！！！​ 4066. 这轮疫情来势汹汹，大家的神经每天都绷得紧紧的。按理说出了问题就该及时上报并找方法解决，可是总有个别人为了保住自己乌纱帽，瞒报甚至强压基层工作人员执行不合理的工作。唉~希望其他地区高层引以为戒，把抗疫放在首位吧！中国加油！ 4067. 没事都打了疫苗呵呵 4068. 哦吼，好一个精准防控 4069. 感觉这一次上海有点难，希望疫情赶快结束，这样的日子真的过够了 4070. 我想知道六医院事件什么时候给个答复 4071. 热度慢慢在降... 4072. 上海第十南京第四 4073. 领导辛苦了¡评论配图 4074. 一觉醒来小区封了，外卖停，快递停，一点准备都没有，三天了，核酸结果到现在都查不到，也不说什么时候解封，好乱，还有一颗白菜… 4075. 上海六院不出来解释吗 4076. 中国人还是老样子，窝里横，出国连个屁都不敢放。挺好的，今天骂上海，就像当初骂武汉一样。同胞这个词算了吧。不同阶级的人在一个国家也不是同个世界。 4077. 问责严惩 4078. 每次报道都是上海疫情精准防控，看到网上那么多视频流出，现实估计也挺乱的。 4079. 此处艾特央视@央视新闻 4080. @梅川酷子67你注意啊 4081. 上海疫情，没有瞒报漏报吗？我周围很多朋友的小区都有确诊都封了小区，可从来没在上海发布上出现过名字！请问确诊的人去了哪里？另外，为什么要大家在公司就地隔离？这样不危险吗？上周新闻发布会每天就不停报数字，什么措施都没有，也不停课！那么多孩子感染！！ 4082. 傅一牧是怎么回事？ 4083. 不管怎么说，打人者该抓抓。院部门领导该撤就撤 4084. 六院领导是干嘛的、自己穿防护服不冲在一线缩在后面，一群护士没有防护服不把他们当人，这种领导立马下台 4085. 《热门》 4086. 几时才结束 4087. 评论真是惊呆了，上海乱套了，别的地方有什么好处吗？国人自己互相地域 4088. 我们小区跟没事一样，没封也没让做核酸，不测体温不看码都随便出入睡觉了明天还要上班呢 4089. 我们小区封了，居委和民警都不让拍照！懂了么？ 4090. 还是相信政府，至少我所在的区都有条有序，大家都很辛苦，放弃了休息日坚守在岗位上，谁都有疏忽的时候，尽力弥补吧 4091. 开任务分配（摊派）动员大会，干活高效办事靠谱的即使不吭声，领导的金手指也是会不偏不倚的呢//@CoNnIe-yyyst:我一直不懂那么多人追着骂到底是为了啥？是需要2500w人给你们下跪吗//@红社会组织:球球了，上海以后别装逼了。现在不怕国外输入，就怕上海不认真输出到省外啊 4092. 上海口碑约等于台北的口碑之于台湾吧，天龙人，自视甚高，却极端崇洋媚外，好多人有外国国籍绿卡，看不上外地省市。要不然怎么北京大妈炫耀八旗通天纹，上海大妈炫耀我是美国人。 4093. 现在疫情还有死亡病例吗？ 4094. 热搜就这么掉到第十了！ 4095. 关注一下绥芬河吧，都是命啊 4096. 感谢北京头条的关键词 4097. 这能压下来，真是对大家的不尊重啊刚刚还第八现在第十 4098. 多关心一下医务人员吧。一年到头一直在抗疫，到了新冠病房做六休一。最基本的生活物资都没有。要自己带，吃的也要自己带，八个小时工作制有时候甚至忙到八个小时不停歇，根本没时间出来换口罩。上面下令医生非必要不进病房，全是护士冲在前线，这份工作真的寒心 4099. 在北京发布下，看上海，西安，浙江等等城市pk 4100. 敢情只有新疆在认认真真地防疫 4101. 评论真有意思，黑什么的都有，这时候不是应该越团结吗？我们不是一个整体吗？现在评论撕成这样，都不要造谣就够别人笑话了 4102. 现在喷上海的必有陕西人！ 4103. @小桃子警官 4104. 到底担心的是什么，这个所谓的精准防控，是否把百姓放在第一位？明明身边有疫情，隐瞒不报。公司也只是发一纸通告，注释告诫我们上班人注意防护，少乘坐地铁公交。难道我们没车的要走路上班么？有时候也要为我们打工人多考虑呀，不要一味的想着经济发展 4105. 医护人员辛苦了！精准防控不能停留在嘴上 4106. 拿出骂西安，吉林的气势，接着手撕上海！全国骂遍了，你就是人生赢家了 4107. 在大都市时间呆得长了就以为自己水平就是大都市的水平了，见得钱多了以为自己也是有钱人了 4108. 好多人看笑话啊 4109. 上海加油💪//@神之近卫军:21年，上海承接14137架次国际航班，占入境总量35.6%。今年前两个月1972架次占41.2%。一线、隔离酒店、公卫中心满负荷运转，这样的日子，上海过了三年，上海疏忽了、也累了，但我们坚信他会历劫归来，而所有失去的，都会以另一种方式归来 4110. 有些人就恨不得上海出事，一看到上海两个字就开骂开喷！ 4111. @留的一点芸香在 4112. 回复@凯溡三:全网最佳！ 4113. 想问问具体的阳性人员信息 4114. 听说6院的护士都没防护领导硬让上 4115. 我们小区防疫人员都说是有确诊封小区的但是通报上每天都没有。也不知道是怎么回事 4116. 上海人吃什么？都是无症状 4117. 还记得那个15㎡的奶茶店，上海特色的精准防控 4118. 全国投毒 4119. 在深圳的我们也很难，同样理解上海深圳这样的一线城市防疫难度，面临的挑战太多，太难了 4120. 谁会想要疫情发生的，都是一个国家，大家还是努力共同抗疫吧 4121. 这也能搞地域歧视？有些人的脑子病得好像得过800次新冠一样，脑子萎缩成黄鱼了？//@凯溡三:一个个只会键盘喷，窝里横，就这样子，都不用外媒动手就已经可以内斗了。上海有点情况都跳出来嘲讽，幸灾乐祸，脑子里请有点命运共同体意识，上海如果真崩了，下一个就是你家门口。一帮阿缺西 4122. 我只想活着…… 4123. 各位上海的同胞们你们去理睬这些地域喷子干嘛大家做好自己的事情又不是我们的错该配合的配合防疫没事少在外面乱逛增加防疫负担到时候防疫不力的该担责的担责六院管理真的有问题该处理的处理 4124. 上海真的也很不容易，我们大家加油抗过这场疫情 4125. 一场疫情，让我们明白，活在这个世上，除了生死，其他都是小事；除了健康，一切都是浮云。好好的活着，就是幸福 4126. 有钱就是好，买热搜祸害别人 4127. 据说医生和护士都干架了吧 4128. 上海加油🙏🙏 4129. 热搜压下来了厉害啊 4130. 长三角地区所有境外基本都是上海一人扛了下来，不知道有什么资格说上海，哪个有能力的城市别bb你行你上啊。要多感恩，互相扶持。 4131. 就这么点，貌似没多严重啊 4132. 境外势力真可怕，分裂社会的手段真是一套一套的 4133. m 4134. 上海真是做色孽，承接各种境外输入还要被左边的江边样子哈讲扒讲//@红社会组织:球球了，上海以后别装逼了。现在不怕国外输入，就怕上海不认真输出到省外啊 4135. 就问能居家办公吗迟迟不通知真的怕了不能我闭麦 4136. 只求居家办公 4137. 呼呼 4138. //@红社会组织:球球了，上海以后别装逼了。现在不怕国外输入，就怕上海不认真输出到省外啊 4139. 看着评论，突然能理解抗日时候伪军那么多是有道理的 4140. 加油💪 4141. 能不能封城，打工人明天还得去公司@上海发布 4142. 上海土著觉得这次真的很严重但喷子哪哪都有冲浪不必较真都是中国人何必在哪说风凉话呢 4143. 楼里别吵了，还不如去顶贴让大家重视起来 4144. 还是深圳的领导雷厉风行，66例马上停运公交地铁，非常有前瞻性 4145. 到底是不是传染病呢？那位领导？ 4146. 瘟兵犯汉宫，经冬又历春。精准护毒宝，几度害西京。 4147. 热度不要下去啊 4148. 深圳今晚宣布已经封城，上海也同样被香港所累，为什么香港就不能封关，你们自己爱怎么样确诊是你们的事，为什么来祸害我们，上海和深圳，多么重要的两个城市，就这样栽了 4149. 差点就信了，别骗老百姓了 4150. 我觉得上海防疫整体做的确实好一点，但是大家喷可能还有一部分原因是上海部分人太傲娇了多多少少有点瞧不起别的省，才引来众怒(别喷，喷就是你对) 4151. 上海这效率，这态度。青岛，吉林，深圳都控制了，上海都控制不了！ 4152. 我只能说上海舆情控制一级棒，从迪士尼开始，外省接受上海输入病例通通不敢吭声只能说是外省不能说是上海。大儿子有爸爸罩着别的孩子都不敢吱声。 4153. 评论删的也不少啊 4154. 真想想了解一下上海到底有多少小区街道单位实行14天封闭管理的….中风险地区只划分那些个没人居住的地方有什么用？既然小区街道单位不是中风险地区那为啥要封闭管理呢？就很不理解 4155. 现在上微博就是有人评论下面就有人骂哎 4156. 🙏🙏🙏天呐 4157. 数据做的是真漂亮啊 4158. 小区居民只要做了核酸就可以自由进出，外卖一概不能进小区，出租车快递车可以进，什么鬼逻辑 4159. 唉，什么时候是个头啊 4160. 为什么有的已经被确诊了这几天网上也不见公布，更不见确诊者行踪 4161. 这是真实数字吗 4162. 祈祷台湾再次大停电，让微博安静一会 4163. @六神驱蚊花露水6688评论里在说还有假数据？ 4164. 上海怎么了。求官方说法 4165. 又不是谩骂东北的时候了，深圳上海管控不当咋没人知声呢，真是风水轮流转啊，国人都互相伤害吧，都去看笑话吧，都居家做个键盘侠埋头开骂吧！埋怨完这个吐槽下一个，活该！再出言不逊网络暴力地域黑的人，希望国家出手法律制裁！ 4166. 表面工程做那么好，有什么用。压制舆论，瞒报，算什么 4167. 上海啥时候也没嘲笑过其他地方啊真的逗做得好就是好不好就是不好上海每天接收来自全国各地和全世界各地多少游客你们有谱儿吗，这回一个不小心爆发了你们就这么迫不及待显示自己自卑的优越感吗？ 4168. 上海这波疫情可给有些人逮住了，真有意思 4169. 疫情稳定的时候，大家都大意了…放任香港等地自以为是… 4170. 你去提议让其他地方都封了某城别给进不就好了 4171. 没有全局考量，科学变成了伪科学！ 4172. 香港有只鸟进入广州，瞬间广东鸟都成惊弓之鸟！ 4173. 上海从疫情解封初期就承担着全国唯一入境筛查压力，就算后来其他城市也逐步开放了，上海还是承受着过半的入境人流。这样不搞精准防控难道还次次都全市封控？那么多无症状感染者哪个城市能提前查出来？ 4174. 如果去年听了某医生的建议，在结合香港的现状……预计到现在死亡至少几百万，“放开”没错，但要看时机，但绝不是医生提到的去年的时候…… 4175. 香港疫情目前为止数据显示，死亡率和其他国家相比名列前茅…这数据意味着什么？？死亡率为什么比别国高？如果放开，以香港死亡率计算，计算过吗？中西方抗疫不同点在于：中国希望每一人都活着；而西方“与病毒共存”是野蛮的自然淘汰制。 4176. 有些武断了。每个城市地理位置，经济发展，人口数量，医疗资源都不相同。全国各地不因地制宜，采取一刀切是以牺牲经济和生活为代价的。一次两次可以，次数多了谁也受不了。上海本身防疫压力就大，病毒在改变。只要有脑子都不能去照搬别的城市，还得因地制宜防疫才行。 4177. 以体质论实在说，中国人体质比美国要差，据最近数据，中国人75%亚健康（抵抗力差），放开？如果去年放开，那是关乎民族危亡的事情……大都市偷摸的搞，那是建立在国家零感染防控基础之上的…才没出大事，即使这样还是影响了全国……有些人要负责任的… 4178. 放开，国家早就想，但要看死亡率……香港的“与病毒同在”，目前750万人口，每天死亡近300…… 4179. 我们上海市民自己也很希望跟你们一样管控方式，我们现在不敢上班还是不得不上班，每天都在跑毒！！ 4180. 这么久都没事，作为上海人，非常肯定上海的防疫！ 4181. 让香港的都飞你们那些地方吧，让上海喘口气 4182. 全国尤其大城市恐怕真的是只有上海没做全员核酸了，周围扩散出去的外省市有一例就都做全员核酸了....上海这所谓的精准真的是蜜汁无语，公司附近基本被阳性圈包围了，街道也没让做，公司也不做...... 4183. 上海精准防控那么久都没事。是因为香港三百人来隔离。空调没有装过滤循环。病毒外泄的。 4184. 上海花了多少防疫成本！肩负了多少境外航班，居然如此内涵上海！奥密克隆本来就传染力度大。本身上海这种人口密集城市就不能，也不该担负这么大的境外输入！！！ 4185. 经典地域攻击言论不看全局，只挑有矛盾的点说事，这种不客观的言论大家看看就拉倒吧 4186. [福气虎][福气虎][福气虎] 4187. 面對疫情，做好防護 4188. 中午快到了，午餐愉快 4189. 晚安好夢💤🌙 4190. 支持博主觀點 4191. 你去点名首都啊，首都也是精准防控，只会说上海 4192. 上海的精准防控比我去的绝大多数都好好吧张口就来 4193. 价值几百万的防控和价值几十万的防控，政策怎么一样呢？你出钱？还是你把全国文化经济一致化呢？ 4194. 起码某地人去外省自觉的做核酸传染完了拍拍屁股走人裂了真的 4195. 上海也是一个香人外溢来的，他不是直接飞来上海的，是先进了别的地方，然后在来上海，没有隔离到处走造成的 4196. 坐标上海。我认同上海的防疫政策。你可以建议你所在的城市封闭起来，鸟都不进。 4197. 关键在于那个地方作为全国金融中心不能直接封，代价太大，而且封了也不一定有用，人流量太大等确诊了密接早就全国跑了 4198. 让香港飞机飞你那去，让境外飞机都飞你那去，我们上海还就太平了! 4199. 疫情每天全球都有爆发的可能，这个原因不是你一个键盘侠了解的，上海也没有完美，但如果，连上海都新增，那你们那里爆发后将不堪设想。 4200. 精准防控才是上海经济外贸高度发达地区的正确选择，动辄停摆封城对经济和正常生活会带来毁灭性打击 4201. @X车厘子小丸子倩倩子Q 4202. 说上海害了这个地方害了那个地方的键盘侠，跪求你们去和上头提，让你们觉得能接手这么多境外输入的人员，那么多隔离人员的城市或者省来接。不否认上海是做的不好，反正啥事儿都骂上海，上海就跟亲妈生后妈带似的。嗷嗷被说这说那，说上海歧视外地人，说上海害了八个城，说上海崇洋媚外，上海欠你们了？ 4203. 精准防控没有错，但是适时地调整也是必要的，不能认为精控适用每一种场景，躺在功劳簿上不动了。国际大都市的担当和能力，要拿出来！加油！我们可以的！ 4204. 一定要做好防控啊！做好居家隔离 4205. 当时划的有多小，有多精准，现在就有多尴尬 4206. 某些人站着说话不腰疼，天天夹枪带棒带节奏，有意思吗？ 4207. 都这么严重了还不封控停课，都外溢多个省份了。 4208. 直接投诉这种博眼球的吧 4209. 你又知道上海不检测了？蠢还是坏啊 4210. 微博上各种V的格局和素质实在太低 4211. 相信上海 4212. 上海的精准防控屡赌屡输？现实更加证明了，其他的二三线城市的无差别防控，除了给老百姓添麻烦之外，对疫情也没有起到什么作用。优等生一次考差了，一堆差生过来落井下石，可笑。//@大风吹奏:所谓的精准防控近乎于赌一把，还屡赌屡输。 4213. 你是官媒吗？这样说话的是想咋样🙏 4214. 啊对对对，正面消息酸上海，负面消息黑上海，反驳了就说我们上海高贵说不得，太典了，差不多得了 4215. 我记得上次我说上海一直没有全民检测很危险，下面还一群妖魔鬼怪说上海多么牛逼我们多么不懂呢！ 4216. 学好自己的专业、做好自己的本职工作多么重要。“以高调开始，常以低调收场”，现在怎么不高谈阔论了？ 4217. 不宜这么说，在总体规范的前提下的探索，在一定情况下也取得一些成效。当然还需要更努力，更加实事求是，科学态度最重要，当然对于上海昨天指出的谣言惑众，信口雌黄，瞎忽悠，胡咧咧的不学无术的不规范，如同阿飞一样就会胡扯的要批评。//@大风吹奏:所谓的精准防控近乎于赌一把，还屡赌屡输。 4218. 回复@一片海的愿望:医院和各个机构都开着，可以自行去检测啊 4219. 这也太可爱了吧 4220. 希望疫情赶紧过去吧 4221. 不要祸害四方 4222. 快扎紧你的篱笆，把门锁死，别出来，也别让其他的生物进去，那一定安安稳稳的。 4223. 上海人表示对上海领导班子很失望！追责吧！ 4224. 上海实际上很多地方都已经在全员核酸了，只是没有集中性报道而已。对于上海五条独立传播链的疫情，遍地开花式的传播，就算有再强的防控管理能力也会散拨在一定范围内。至少目前社会面确诊极少，基本都在传播链上管控了，对于一个城市管理来说，即做到范围的精控，还要尽可能减少对市民的影响确实不容易 4225. 群体免疫现在看起来还是有点道理的 4226. 沪卫兵都赶到了，小心点 4227. 图片评论¡评论配图 4228. 各地发展情况和执政能力不一样，一样的防控政策和力度付出的代价也不一样，各城市定位也不一样，在当今的国内外环境中需要多种平衡，不是能够简单一概而论的，所有统一的，如果那样子，那么就需要放弃很多东西。 4229. 试点放松清零政策不是好事么 4230. 上海这一波还没有因奥密克戎死的，但我相信封闭小区搞一刀切，肯定有人会因急病不能就诊死的。 4231. 全员核酸不可能的，2020年新冠最疯狂的时候都没实行全员核酸 4232. 现在恐慌才是最可怕的 4233. 同意政策防控需要一致，但也需要考虑民情经济和目前的疫苗普及状况，三年了病毒都进化几轮了还按照以前的一样的方式走，真的还可以吗 4234. 我觉得上海的精准防控很好，只封该封的，不封不该封的 4235. 我再次建议你去了解上海精准防疫的政策和举措，在保证城市高效运转的背后，是无数防疫工作人员在精准防控，岁月静好是有人在替你负重前行，很多城市的防疫是平时疏忽，出事封这个封那个，相比于西方确实是尽职尽责，但是在花费大量精力在平时的上海，这就是一种懒政。 4236. 上海的防疫工作你可以去了解一下，在没有疫情的时候有多少工作人员把防疫工作做在平时，才做到不仅达到精准防控，而且保证学生可以正常上学，成年人可以正常工作，这次的疫情，防疫部门已经公布是因为一个管理疏忽导致的。上海在很长一段时间的精准防疫的成效是显而易见的。 4237. 不现实，不说上海没这么多人手实现全城防控，每天的花销也得以亿做单位 4238. 上海人非常肯定上海的防疫 4239. 这次上海是管理疏漏引发的，跟精准防控有啥关系？ 4240. 兄弟，你不是上海的吧 4241. 为了一个逆子把自己搭进去 4242. 全国各地防控政策一致，那要不要境外航班也都往全国各地飞不要只飞上海广州等城市，这些城市承受着多大的压力，你真的是站着说话不腰疼得了便宜还卖乖 4243. 为什么不是全国向这两个城市看齐？ 4244. 香港什么时候与病毒共存了？乱说 4245. 短视者，你就不想想都一刀切封经济民生能承受？你去养活那些低收入者？ 4246. 精准防控做得挺好的啊，上海这么大的城市不可能动不动就全员核酸吧 4247. 没办法一致的，还有国外呢，国外没法和我们统一，以后总要打开国门， 4248. 精准防控到底怎么样你感受过吗真的张口就来上海接收境没人夸一出事就来内涵还某地方…..马后炮这种话少说 4249. 我认识一个香港来的孩子，我说你来上海，别乱跑。他说巴不得能得新冠。还说新冠也阻止不了他出去玩。没法沟通。 4250. 瞎说什么东西不检测就知道阳了吗用眼睛看的吗 4251. 上海别的不知道怎么样，甩锅水平全国第一名。六院出事，外地患者住院阳性。感染多人，大头都是无症状，确诊是个零头。难道恰巧外溢其他地区的都正好是确诊？ 4252. 哪有空着的离岛，把上海和香港一起打包送去相亲相爱完事，两地挺互有好感。只要不闹独立，随他们精准防控装逼去 4253. 我记得不举是轻后遗症来着 4254. 丫天天装逼精准防控，结果外溢把好多城市都害了 4255. 不会有事的上海和美国一样发达 4256. 难怪娃读书内卷那么厉害，防疫也卷 4257. 让境外输入飞你们那边呗，上海的防疫政策和力度科学精准，国内没有哪个城市更好了吧 4258. 某市的不检测精准防疫政策，为国内多地爆发贡献了病原，这属于疫情防控不力吗？ 4259. 不懂不要瞎说 4260. 上海从头到现在承担了多少境外你们看不到上海遭难了你们如此得意咋滴了中国人欺负中国人啊有意思吗我们不慌魔都加油 4261. 上海防疫水平没毛病，发现问题及时有效能控制 4262. 为什么现在还有人嘲笑精准防控了。为什么连上海都有黑子在网络批皮上海人吹上海。上次和这次性质不一样。上海这次都全员核酸了！医护这么累，志愿者那么辛苦，蟑螂躲在网络背后嘲笑有意义么？ 4263. 看到有人喷你，我就放心了[冰墩墩] 4264. 在上海的人都庆幸自己在上海，没有精神压力的管控，其他地方都给老百姓巨大精神压力制造恐惧感，愚蠢 4265. 你又知道不检测了，基本所有企业每周一次，有的两次，不知道不要大放厥词 4266. 死要面子的沪城河 4267. 你除了当喷子还能做什么，妖言惑众！奥密克戎这个病根本没法，高福也说了当时的预判失误，以为打了疫苗可以全民免疫屏障！现在我们要意识，她的共存，改变防疫策略 4268. 很多国家都开始和病毒共存，我们如果不死防，就实行共存政策，但是，不实行也不严防的话，浪费的就是大白们的牺牲。 4269. 这次疫情过后我们上海再也不接收输入性新冠源体，外国回来的各回各家各找各妈，谢谢你们 4270. 你的名字就取对了，来上海的出上海的都自费隔离21+21天，大家就都太平了。 4271. 一国两制……政策制度问题，做不到的，大陆也没法完全管理香港 4272. 这波疫情本质是香港政府不作为，防疫政策与大陆政策背道而驰导致的，凭什么上海人要背锅，别的省市疫情爆发的时候上海人说什么了吗 4273. 要不把国际航班都赚到北京去？看看你还骂不骂？ 4274. 放开估计是最后的选择 4275. 小心你号没了有的人为了洗白，连航班号和籍贯都改了 4276. 注意安全，注意防护 4277. 全国各地防控政策必须一致啊。 4278. 唉，希望疫情早点儿结束吧！ 4279. 真心的希望所有人都能够远离疫情 4280. 好可怕不会传播吧 4281. 疫情赶紧过去吧 4282. 太难了吧希望疫情可以早点结束 4283. 香港这是管不了吗？以后湾湾更难。。 4284. 希望你们不要来上海 4285. 香港🇭🇰一定有内鬼不作为想搞乱香港拖中国大腿！务必谨防！！！ 4286. 把香港看成和其他共存的外国一样就可以了，香港是传染源，但不是唯一的传染源。 4287. 那些想与病毒共存的都是已经感染的吧？与病毒共存的后果就是人口减少，经济下滑 4288. 墙倒众人推被你诠释的淋漓尽致 4289. 全民同心共通防疫不好吗？非要站在上帝视角阴阳怪气的去职责，是不是心理有问题？与其蹲在电脑前无脑喷，还不如为自己城市疫情防疫做出点绵薄之力！如果实在控制不住自己的言行，建议可以看看心理医生！ 4290. 为攻破中国防疫，东原乐多少水军，看下面评论就知道了 4291. 张网红医生内心向往美国新加坡抗疫模式，必然对中国模式有抵触，抗疫工作懈怠 4292. 说实话，上海承担了很多。如果可以的话，那种本地有三甲医院和隔离医院的地区帮上海分流一点就好了。像成都那些的话，其实压力也挺大的…… 4293. 建议你的当地政府启用熔断机制OK，全部自给自足，相信就没有疫情影响了 4294. 你以为为政者不比你聪明？ 4295. 一看中医粉，就懂了。 4296. 远在上千公里外的我因为受到上来疫情精准防控的快准狠的防疫政策的影响，此刻正在居家隔离呢14天，工作都不能做就整个一离谱！ 4297. 上海全员检测一次比较好 4298. 支持老铁 4299. 在苏州站进出站都要查验48小时核酸的时候，多次出入上海站，却从未查验任何内容，单从上海市市民个人体验来说，防疫确实相较其它很多城市没有那么严格。原以为是精准防控下的自信，但前几日太仓通勤的病例确实证明上海疫情外溢，希望上海能够提升大城市担当。实事求是。 4300. 无脑跟风 4301. 我最讨厌这种人，有本事你去和国家申请把浦东机场承接对外输入人员管控的任务拿到你们城市去，看你能做到什么程度？ 4302. 关键是香港继续这么随意永远都弄不好啊然后这边一直要收 4303. 傻子 4304. 中国2022年这波疫情就是香港人传开来的。害深圳年前核酸不停，封禁不断，深圳就是不肯把深港口岸给停了，禁止香港人入境！ 4305. 太可笑了，其他地方全员核酸封城，就是为了保卫上海北京这样的经济政治中心尽量过正常生活。你本末倒置，不理解国家政策和全局。 4306. 上海两年多的精准防控，取得的成绩有目共睹，风平浪静时漠视上海，上海防疫工作者一直在默默付出，从未松懈。目前的确出了问题，大家也没有想着帮助上海，而是用文字抨击上海，文字可以抗疫？不如去当地做做志愿者大白，为疫情真正的做出自己的贡献，我们相信上海防疫部门~会还我们安全的生活环境 4307. 港府豁免机组人员免隔离的时候市民专家都是反对的，有用吗，弄出个大窟窿出来港府现在就装死，破罐子破摔了，如果这群尸位素餐的官员中央不问责就搞笑了。 4308. 现在非得把确诊和无症状分开算，无症状一样是被确诊阳性有传播能力，然后统计里基本都只写确诊总人数，不写总无症状数，都jb搞不清到底有多少人真得新冠了 4309. 建议核平香港 4310. 你为啥觉得自己那么懂，去应聘一下疾控主任吧 4311. 阴阳怪气你是什么防疫专家吗 4312. 不检测？？你臆想的 4313. 太不负责任了🙏 4314. 这次决定接收大批香港人的决定的人应该出来负责人 4315. 可是马上要中考高考了，要真上网课，我们这届就完了 4316. 今天公布的五例和21例无症状还没计入 4317. 2020年的表吗？ 4318. 为什么自告奋勇要分流香港居民？出事了吧？ 4319. 适当做一做压力测试也行不能永远死守 4320. 奇怪，这回突然上海严重了 4321. 周六还要聚众教资😅 4322. 继续上班上到确诊 4323. 疫情到现在已经不能像以前一样死守了，无症状越来越多就是征兆，是时候尝试放开了，而且中央已经在提倡精准防控，这就是给放开的信号了。 4324. 哈哈作为过去三年生活在广州的表示真的没啥去年12月底刚来上海，小区就封封楼两次，有一次还是我住的楼，只能说之前上海防的太好了一些人接受不了这种落差吧，该干嘛干嘛呀，服从管理 4325. 周六的教资....又是一拨.... 4326. 市教委还在硬撑什么啊我不理解 4327. 楼主看发布会了吗这些病例都是在集中管控时期发现的不能只看数字不看实际情况啊… 4328. 我不是杠哦，因为各种观点，甚至截然相反，停课歇业又有另一批人骂防疫过度 4329. 与病毒共存，群体免疫是最终的选择，清零政策在没有“疫苗”出来之前都是苦最下面的人… 4330. 闵行已经停课两天了。谁说没停课啊 4331. 一哥你好red今天 4332. 青浦一例都没报就离谱根本不知道哪里有 4333. 实话，如果现在再全部居家，那可能，国内经济问题更大了 4334. 2020年是封城了，你要封城吗？ 4335. 为了所谓的GDP，为了政绩！ 4336. 因为国外都开放了这是才测试国内疫情抗压力，以后肯定与疫情同存 4337. 不仅不停，现在直接连周末都没有了 4338. 有没有人管管松江大学城朵拉大学城 4339. 呵呵，上海的思维可不会那么落后，学会逐渐共存，上海一定时走在其他城市的前列，现在还在坚持清零的，早晚会被时代所淘汰 4340. 就不懂为啥。我们已经被有确诊感染的封闭小区包围了，孩子们还正常上学的 4341. 上海根本就没有打算严防死守哦，想太多 4342. 崇明是净土 4343. 漏了，青浦多的是 4344. 命到底重要吗？是不是要整个上海所有人都确诊了才肯停课居家办公？打了疫苗有什么用？该中的还是中，不该中的也中了，这是在拿老百姓的命在开玩笑。这样下去上海早晚沦陷 4345. 我们公司已经要求明天开始在家办公，暂时先到3月底 4346. 想问考研复试还能正常进行吗？ 4347. 虹口这个，压根没有报道 4348. 居家办公？公司让我们准备牙膏牙刷毛巾去公司办公，我真是操了他的🐎了 4349. 我也不知道为撒学校都不停课却要求线下培训全不停课？可真牛逼 4350. 最近在忙着为两hui删微博，没空办正事儿 4351. 闵行区好多难怪周边医院学校都封了。 4352. 为了能继续吹，上海速度，精准防控 4353. 真的无语，附近小区就有几个都封了，隔壁小区全员核酸，就是不停课停工，疫情刚开始的时候都没这样过 4354. 感觉是个放开的试点，不然干嘛不停工停学 4355. 继续上班刚做完核酸周六聚众考教资 4356. 学校门口就有个确诊的小区。。明天还要继续上学。。出了门都怕回不去。。😭 4357. 真的不知道在想什么普通百姓的命就不是命了就离谱 4358. 应该到别的城市就被查出来的的也算上 4359. 不知道哪些煞比瞎传造谣的。一边不相信政府，一边又相信公布的数字？？ 4360. 从公司有确诊爆出到现在30➕h过去了从昨晚就在说的第一轮核酸安排到现在还没给安排上大几百号人被封在楼里、基本都是密接、次密接想问这样真的安全吗？还有、这就是所谓的上海速度、精准防疫么？30+h过去了啊！！！O网页链接 4361. 整得到处是密接，次密接，就上下班的功夫，就接了 4362. 精准防控么。上海不一直是好学生交作业大家跟着抄 4363. 呵呵，上海真是个好地方，不敢说，说了就有护城宝出来喷 4364. 浦东惠南的教育机构出现确诊！！教育机构不是明令禁止了吗？还出现这样的失误？彻查！！ 4365. 我们学校停课了 4366. 还不停课。上到确诊也不停课真是大城市啊 4367. 我在摆烂上海也在摆烂 4368. 没事的，热搜能压就压，压不住等过了这个风头，领导买两条宣传热搜就好了，依旧还是国际大都市 4369. 最可笑的是很多公司非但不让居家办公，还要求三天做一次核酸，凭证上班，这是怕员工去不了人群密度大的地方交叉感染么？…… 4370. 我是真怕，家里有小孩，哪里也不敢去 4371. 6月要中考家被封了学校上课一分钟都不能少啊现在不让我上课了能不能都别上真的对我公平吗我要上高中的啊我要听课的啊别人可以上课我就只能在家坐牢，再不停课等封更多感染更多吗 4372. 我们明天幼儿园师生一起核酸检测了 4373. 学校被包围了 4374. 绝了，天天在家都还是觉得不安全，门都不敢出 4375. 闵行我真的会谢 4376. 是不是在试点 4377. 快点让本地大学生回家网课吧我真的求求了学校明明没有任何人确诊但就是吊着不上网课 4378. 上海都是些来镀金的领导、弄的上来越来越不像样、就知道数据好看 4379. 我不敢发截图，教育局发布的通知：关注学生的出勤率，出勤率低的个别沟通 4380. 崇明还没有是吗 4381. 我们公司倒是周日就通知居家办公了。这波接收香港人算是坑苦了上海 4382. 明天会更新不？加上3.10的估计要超400了 4383. 去年静安寺奶茶铺爆出疫情的时候就和同事说了，上海真叫没搞全员核酸检测，要搞的话感觉会有很多，现在不幸，被我言中 4384. 今天早上4+76，晚上又5+21… 4385. 为什么我开始怀疑这个数字…（现在很严重，那时候大爆发为什么只有349……单纯疑惑……别骂我） 4386. 真的烦！香港的滚哇 4387. 321例是现在的？ 4388. 这个病毒这辈子肯定结束不了了，还是学会和它共存吧 4389. 真无语了还不停课有病吧 4390. 全军覆没. 4391. 不能松懈，带好口罩！ 4392. 上海在想啥 4393. 听专家的做好自己 4394. 周六周日还要继续上课，连上十一天的课 4395. 我们刚才还在讨论这个问题，第一次的时候感觉真的没几个，这次这么严重居然还不居家 4396. 停了，只是部分，我就是停的一个 4397. 嘉定人多的我都害怕 4398. 救救 4399. 上海这几年挡了这么多境外，看来这回是真的累了～。大白们辛苦了！ 4400. 你搞笑吧！只是你那地方没停而已，这就要检讨了，领导你来当，我现在住老师家蹭吃蹭喝就等着回去呢 4401. 因为产能刚提上来吧，不舍得停摆，停摆太影响经济了 4402. 回复@人生是一个茶几:安徽浙江江苏都有回沪的感染病例了上海还是一点点的挤也是神了外地人跑上海一趟回来就有了所以上海到底是个什么情况了？ 4403. 兢兢业业守护了上海两年兢兢业业，出一次事情就被骂，唉，上海政府真难 4404. 因为是奥密克戎了，要开放的前奏了。 4405. 能不能居家办公现在完全看老板了 4406. 我拒绝。 4407. 说的很对 4408. 到现在还不居家办公就很离谱了。。 4409. 去崇明岛吧 4410. 瞎搞精准 4411. 是的呀烦死了明天还要上班都不敢坐公共交通 4412. 为什么上海没有人被免职？ 4413. 停课了啊！ 4414. 很多单位已经在家办公了 4415. 上海确实不严，正常下了高铁就该核酸检测。然后疫苗也是，我加强针都打了大半年了，上海的朋友还不知道去打。个人感觉各方面都很宽容 4416. 放心上海发布，已经通知网课了 4417. 上层建筑你不懂… 4418. :看上海教育发布新通知 4419. 毕竟还要接受香港过来的呢香港才是国家儿子 4420. 一个政府如果不是为自己人民而只是想做出政绩来，那么早晚会失去市民的心！ 4421. @希望永远睡懒觉 4422. 你这条居然没被压下去 4423. 虽然感恩感谢医护人员们🙏但是也请真实面对这次上海突发的疫情，请求上面不要再瞒报了！拜托🙏现在哪哪都是人心惶惶的 4424. 呦呦！怎么觉得你有点幸灾乐祸呢？ 4425. 脑子里都是💩 4426. 上海不愿意承认精准防疫失败。 4427. 疫情最厉害的时候都没这样，坐标闵行，昨晚突然小区就被封了，说是普通筛查，却要14天，也没爆出有病例 4428. 现在官方发布都是留言精选，一片和谐，两年前可都是接受群众的批评的 4429. 格局打开看问题的角度自然不同 4430. 还有就是既然有的地方做全员检测，也不好好配套做，乱糟糟的 4431. 事实就是以后这种情况就是常态，不可能让城市停摆的，国外做群体免疫情决定也不是全无道理，再基于过去两年的抗疫经验，上面肯定觉得目前还是在可控之中的，咱们配合就是了 4432. 你什么都懂，去抗疫啊 4433. 是为了钱吗 4434. 上海这次一塌糊涂，管控地方，120车也没，进了医院，出不了医院，没有120接送，不可以自己走，一天了大家都回不去，上海到底有多少人在叫120，简直是想骂人了 4435. 因为不敢，一旦停工停学等于打自己脸。而且现在是什么时候，在干什么 4436. 生病事小，饿死事大 4437. 同感 4438. Gdp5.5的目标 4439. 是需要好好检讨 4440. 看看这次撤职哪位 4441. 坐标上海闵行一小区，昨天开始要封14天，他封小区我要疯了 4442. 说句你们都不信的话，我们厂到昨天为止进门都不需要测体温的 4443. 到处在封，既防不了扩散，又折损了经济，邻居上班也是跑客户，防不胜防。呼吁封城。 4444. 这次明显厉害多了 4445. 上海真的被坑惨了，境外输入隔离酒店根本不够住，人家不管接着硬压指标，很多酒店打不到隔离要求，很多人员还不具备足够的防疫素质 4446. 小区里隔壁楼都被封了还得正常去上班，只能说心是真的大 4447. 身边很多朋友的学校都有同学家长确诊 4448. 这波疫情真的影响挺大的关键学校居然不像以前一样停课搞的那么多学校需要检测还是带着家人一起的 4449. 我靠我靠我靠我又不敢睡了 4450. 我们公司都发内裤了，意思是工作到确诊，然后老外本周五还要去开40个人的派对。牛批，顶风作案！服了！ 4451. 海纳百垢去他的吧 4452. 其实上海的防疫工作全靠上海老百姓怕死的心态，防护自觉的不得了 4453. 不明白为什么香港那么多严重还要开放航班出来？ 4454. 我不信西安来两个上海的都30个了上海怎么可能这么少 4455. 跟香港情况还是不一样吧，这里没有出现大量重症挤兑资源啊，不筛查都不知道附近小区有隐形感染者。 4456. 确实很精准，遍地开花罢了 4457. 教资都头铁还要考 4458. 深圳高校一个都没有还都封校了，教职工全部居家办公 4459. 害人 4460. 公司通知明天居家办公，但是办公室正常开放，我们领导特意强调：要居家办公的跟他打招呼，这意思就是不让居家办公了吧，我没理解错的话 4461. 20年上网课，中考高考结束上海多少学生跳楼？！希望领导们珍惜孩子们的命！ 4462. 这就是防控精准到人，不影响别人的生活啊，有问题吗？只要没被通知，该干嘛干嘛 4463. 之前全名打疫苗搞了鸡飞狗跳！现在学校又要强制孩子做核酸了！tm卫健委几人开发布会没有一个带口罩的。要求人家不要聚众。出门带口罩。自己都没有以身作则。 4464. 过几天学校要核酸检测还不停课不过中考快来了不好好学不行呀 4465. 几十万餐饮、娱乐、旅游服务行业就业人员怎么办？都奄奄一息了，还能封城不成，涉及百万家庭 4466. 上海会精准定位防护，可以控制管控区在几十平米內，大家不要慌 4467. 真的太可怕了我们学校都是被叫回家的同学还不听课而且在学校也不戴口罩甚至就在前一天早操时候校领导和大家说下楼做操时不用戴口罩太闷了 4468. 无语了还你妈不停课 4469. 如果重复感染的几率很低的话，还是有序开放共存吧。给感染过新冠的通行证，可以出入自由，随意跨省出国。 4470. 上海真的头铁 4471. 抗疫都抗魔怔了，动不动就封城，动不动就管制，寸步难移，这样的日子没完没了了吗？逐步放开不行?非得天天关禁闭? 4472. 这样的格式很一目了然呀，以后发布能不能用表格的形式 4473. 精准防控不行了？ 4474. 不停课反而通知我们上班时随身带被褥折叠床洗漱用品去他妈的宁可抱着集中隔离的决心也不停课头铁的哟吐了 4475. 公司都倒闭了上哪打工 4476. 正常上班，不过大家都把口罩戴起来呀，在公司也应该带。外面来访公司谈话也都没带口罩 4477. 求求让小学生在家上网课好吗 4478. 我上海客户说了居家隔离成本太高了 4479. 看到的还是被压过的 4480. 对于学校通知带好洗漱用品，保暖用品和睡袋这个骚操作我不理解。把老师就在学校镇压病毒吗？熊孩子怕老师病毒又不怕 4481. 已经完全不是一种病毒了。 4482. 有没有可能病毒可控 4483. 别站着说话不腰疼了，作为家长、不想要停课，想停课的可以直接请假在家空中课堂 4484. 把官方平台骂到N次方的，肯定忘不了//@水笔小欣:要政绩拍马屁啊，2020年疫情刚开始骂扑天雕骂到封词条都忘记了？//@人生是一个茶几:这次决定接收大批香港人的决定的人应该出来负责人 4485. 好可怕 4486. 这么多了救命🆘 4487. 怎么说呢，停工停产，社会咋办， 4488. 已经有不少小学停课了 4489. #上海疫情#请问小区隔离了，没有任何方案可以让人出去打狂犬疫苗吗？狂犬疫苗的致死率可是100%，上报就告诉你会继续向上报备，要报到什么时候？已经超过打针时间了 4490. 就这松江欢乐谷，上海迪士尼还开着呢 4491. 管的最严的时候只有20年头两个月而已 4492. 上海被网上自己发点新闻夸懵了吧 4493. 到底在坚持什么啊我不想戴口罩线下上课在路边吃食堂打的饭因为食堂不让堂食一进学校就担心下一秒被封所有活动都停了还坚持线下上课图啥啊我 4494. 很简单这样是对正常的生活和工作影响最小的哪里有问题封哪里也可能是未来的趋势。 4495. 现在是实验放开的前奏吧 4496. 键盘侠抗疫第一 4497. 真要没了 4498. 这是高传播性的奥密克戎，不是2020年的老株 4499. 哎～怎么肥事儿呀～ 4500. 全国疫情看上海感觉我们这次风向标是要和病毒共存的样子毕竟奥密克戎干预速度比不上传播速度清0政策不适合这代病毒了主要是清零后一旦恢复运作疫情又来了上海感觉在走一个新方向但是中央没开口地方也不能自己做主。看吧！ 4501. 上海稳住啊 4502. 停课可以居家办公是打工人的狂想曲只有老板面临压力居家了可能就裁员了还办啥工 4503. 我们早就居家办公了 4504. 搞得到处都是了，我们这也有了 4505. 精准防控厉害了 4506. 建议上海市长下台 4507. 我觉得很好啊，没有影响生活不是蛮好。 4508. 群体免疫吧累了生死由命吧 4509. 那些当初吐槽深圳表扬上海的人，知道深圳人有多难了吧 4510. 青岛也很严重 4511. 在家办公我们一家子就得饿死，唉，这疫情真的快点过去吧 4512. 继续上学上到确诊三公里内八九个小区有病例周围所有小学初中都停课就我们高中天天跑毒决赛圈 4513. 可能在下盘大的吧 4514. 相信政府 4515. 上海其实也是压着没有爆出来实际上也是有很多例 4516. 上班上到确诊封城前的西安如出一辙 4517. 也是没办法在规则下为了活着 4518. 实际数量谁知道 4519. 不过呢，2020年初当时都没打过疫苗，是应该停摆一切的！现在3针打完了，再这样封城的话，就要质疑……到底有效吗？纯属个人看法 4520. 娱乐场所今天停了，电影院从明天起也停了。 4521. 居家办公就无法压榨打工人了，就不存在内卷行为 4522. 我在嘉定继续搬砖恐慌中搬砖 4523. 我是大为震撼啊 4524. 两全马上要结束了…… 4525. 应该是奥密克戎传播性很高的一方面因素，然后用经验来看新问题没重视吧？ 4526. 检讨什么？检讨你为什么没病么？ 4527. 请不要给国外反对势力递刀子，相信政府。谢谢 4528. 我买的夏目友人帐展是不是只能退了到四月十号 4529. 没打疫苗的孕妇真的害怕 4530. 为什么要停课？ 4531. 青岛一千多了，说不定还更多 4532. 民不聊生 4533. 明天早上发布还会翻倍，崩溃！！！！！ 4534. 一直信任上海 4535. 毒圈跑酷哈哈哈哈，我爱上班 4536. 上海市民应该委托人大代表向市政府问责… 4537. 精准防控，当然停课！ 4538. 号对我来说不重要，有停Ke的但不让发，你猜他是要里子还是要表子…上海加油 4539. 我今天才入职啊 4540. 精准防控执行得很不错，说到做到，为啥要检讨。病毒感染危害流感化和国际社会逐步开放的大背景下，防疫只是生活的一部分而非全部，有疫就防无疫该干嘛干嘛才是正常的生活节奏。 4541. 是的，因为上海来了个人到南京，南京多了3例确诊病例 4542. 看了吉林农科，上海学校还不采取点措施！！ 4543. 感觉逐渐要跟病毒共存了，不如以前管得严了 4544. 然后在上网课的我，下周要去外地上学了 4545. 20年5km内有病例都全体紧张，目前大家就只希望不要被隔离在公司 4546. 这表不对吧，宝山3.8号没有这么多呀 4547. 那要快看看吉林，一天一百五，把你半年指标达到了， 4548. 我知道很多学校因为有密接，次密接都停了，学生和同住人都要做很多次核酸，我觉得上海防疫做的很到位了，能留在外面的会有多少是真的危险的？？？ 4549. 累死一线医护，一线居委，一线保安，公安人员，封这封那，就是不停学。不停工可以，为啥不停学，真是看不懂了。今天这个学校闭环，明天那个学校闭环，折腾小学生，折腾家长，折腾老师。哪个白痴领导出的政策，真是火大… 4550. 我们苏州算是挺过来了，但是都是正常上班的 4551. 无语了 4552. 上海大学 4553. 按照这个趋势，明天无症状感染者破百了 4554. 学校不停课，那就只能等着孩子直接被封在学校里了 4555. 虽然你说的有道理，但是有一说一无症状应该是不算在内的……这种连中风险和带星都不会。 4556. 要命了……… 4557. 附近5km就有疫情，还有人不戴口罩，就是因为现在大家都松懈了，才会爆发这么快。 4558. 真的无语了昨天今天还在全校核酸已经有高中的老师确诊了就不停课。真·坚持上学上到确诊 4559. 有道理～每天送娃，然后上班，提心吊胆 4560. 可能因为这次致死率低了，不按照20年那样对待？ 4561. 8号越洋广场临时封闭跑出去很多人第二天又要召回但并不是所有人回去写字楼接受隔离不服从疫情管控不接受核酸检测的大有人在也并没有看到防疫相关部门有什么管控措施防疫形势大于实际意义 4562. 上海这次很严重么？上个月因为工作跟同事一起去了趟，最近才发现带星了居然。。。 4563. 这就是精准防控 4564. 每天数据麻烦更新一下 4565. 不知道在想啥 4566. 难道是探索新模式？ 4567. 设立风险区上海说第一没有省份敢说话积极的很其他防控政策就没得堵死 4568. @培根芝士双层牛肉堡 4569. 可以停课了 4570. 还没停课🙏 4571. 上热搜让上海政府看看呀 4572. 终于有人说实话了 4573. 🙏🏻🙏🏻🙏🏻 4574. 你这表做的比卫健委疾控中心发布会清晰明了多了 4575. 能不能彻底封了香港人入境，嚯嚯广东还不够，又去嚯嚯上海，也是服了 4576. 真的搞不懂，这么严重为什么不停工停学，害的别人现在喷上海精准防控，上海人民也瑟瑟发抖好吧，上面不把我们当人，下面老百姓还排挤我们，我们的苦谁懂，继续上班上学吧，上到无症状上到确诊 4577. 传染力不是一个级别的 4578. 话说松江大学城这边都开始封校了 4579. @C阿君呐我累了 4580. 今天已经发布全面网课了 4581. 今天发布的要听课了 4582. 同事小孩，包括上大学的都回家上网课了…… 4583. 检讨啥？再来摘乌纱帽？一刀切？疫情这种情况是谁愿意的么？大家都在努力 4584. 我们学生都被拉去当志愿者了真的好痛苦 4585. 这个才字，过分了 4586. 可能在搞压力测试，总有一天要放开 4587. 唉，为了居家办公这事，看透了领导的虚伪 4588. 慢慢放开吧，别折腾了 4589. 想开点，我感觉这是在慢慢适应开放，听说这次这个病毒只是和流感差不多 4590. 这次是真的厉害了，前年疫情爆发的时候我们这里郊区都没有的，现在叫我们这里那么偏僻的地方都有病例了 4591. 你不去吆喝禁止外出，就盯着学生不放，你们大人都消停点，学生也不会被连累 4592. 其实是以前运气好，就蜜汁自信的当自己是优等生，然而现在奥密克戎太凶悍，以前那套玩法不灵了 4593. 病毒传染性不一样，有可比性么 4594. 因为都已经2022年了呀，出现病例就停课停学，那老师不疯，家长都快疯了。生活应该慢慢走向正常化，而不是倒退 4595. 真他妈。。。能不能不要影响上海人民生活啊。。。工厂又得关。。。真就乌鱼子。。。 4596. 上海人表示被香港人坑死 4597. 公卫护士表示真的很忙很累很心酸，有苦说不出 4598. 因为开放的态度 4599. 别说了，居家了！ 4600. 和20年的病毒株不一样，传染性高。目前没有危重和死亡，轻症痊愈就好了。还有人因为过度防疫投诉卫健委呢。 4601. 强烈建议中小学停课 4602. 对啊，我们学校离竹园小学只有1km多一点，20年的时候都没感觉疫情有这么近 4603. 上海这是被吹出来的‘精准防控’自乱阵脚了还是有啥别的原因？ 4604. 这次就属实不太能理解了 4605. 别说听课了，我们公司隔壁一个阳性，我们依旧上班，物业政府没任何通知 4606. 还让香港人进上海就离谱！ 4607. 就是啊，我明天还要去上班，浦东到宝山。。 4608. 呼吁停课停工吧，天天看新增的人数，瑟瑟发抖，周围也有封掉的，要是被传染了，谁还干活，暂时不干活，为了以后天天干活，留得青山在不怕没柴烧 4609. 现在的政策是动态清零，不是绝对清零，如果一有病例就停课停工还动态清零干嘛，直接绝对清零啊 4610. 两会期间他们敢报吗 4611. 我只知道外溢到其他省很多 4612. 上海人身体好！都是无症状 4613. 一把支持 4614. 都是被网友给吹出来的胆子，说啥精准抗疫 4615. 淡定点，迟早要放开的，无症状这么多根本防不住，除非中国从地球村移走，都说了政策根据情况调整，反正我听政府的，该干啥干啥 4616. 2020那是过年回家，不一样，这回是人都来了上海 4617. 精准防疫当政绩搞得高调过头了，现在多点爆发不减少流动那就是四处传播 4618. 因为要改变应对方式了，动态清零比较困难 4619. 关键很多防控在捣糨糊！尤其核酸检测，松江某小区第二轮核酸检测随便瞎叫叫，白天来核酸，到点下班，有很多人都没回来？请问这个核酸有什么意义？排队扫码登记信息是一个队伍，到了核酸的时候分三个队伍，请问信息跟人员怎么对的上？这些机构就是赚国家的钱！浪费国家资源！让老百姓受罪！ 4620. 希望上海和吉林市人民平安，要健康！ 4621. 对对对我们不停课还要集体核酸 4622. 爱上网课的就自己申请上网课呗！不要把别人拖下水行吗？ 4623. 就这周末还不延考？ 4624. 早上6点核酸现在没出结果，密接但是接的大巴等了半天没来，疫情到底怎么样了 4625. 只有崇明没有了 4626. 这么夸张？ 4627. 深圳夸张多了，不还是一样 4628. 说实话我不理解上海这次怎么会这么严重，辐射那么多省份 4629. 稀里糊涂各种松懈，进楼查码假模假式，隔离酒店各种感染，境外来的隔离不到位。 4630. 可以停课了，市教委还要害死多少孩子 4631. 都是轻症无症状的，毒性还真以为和两年前一样啊。说停就停说封就封完全不考虑老百姓的心理 4632. 居家办公说的容易，很多工作根本居家不了啊，该上班的都在上 4633. 外溢很多城市了已经 4634. 这还不算境外输入我附近有一个小区今天确诊无症状，然后这附近的小学还在上课¡评论配图 4635. 这个表在哪里看呀 4636. 我们吉林已经600多例了，人口还不到你们的十分之一啊。。。 4637. 快居家吧 4638. 我们办公室10个人已经2个人去隔离了，还不居家办公 4639. 我觉得很有道理还不停课 4640. 看市委有多大的胆量吧！ 4641. 当枪使呗失职也只是被免职 4642. 崇明挺住 4643. 我在上海目前都不知道多少例了 |
